# Bioinspired Binding and Conversion of Linear Monoterpenes by Polyaromatic Coordination Capsules

**DOI:** 10.1021/acsorginorgau.4c00013

**Published:** 2024-05-16

**Authors:** Ryuki Sumida, Lorenzo Catti, Michito Yoshizawa

**Affiliations:** Laboratory for Chemistry and Life Science, Institute of Innovative Research, Tokyo Institute of Technology, 4259 Nagatsuta, Midori-ku, Yokohama 226-8503, Japan

**Keywords:** linear monoterpene, polyaromatic capsule, cyclization-dimerization, stereoselective, solid state

## Abstract

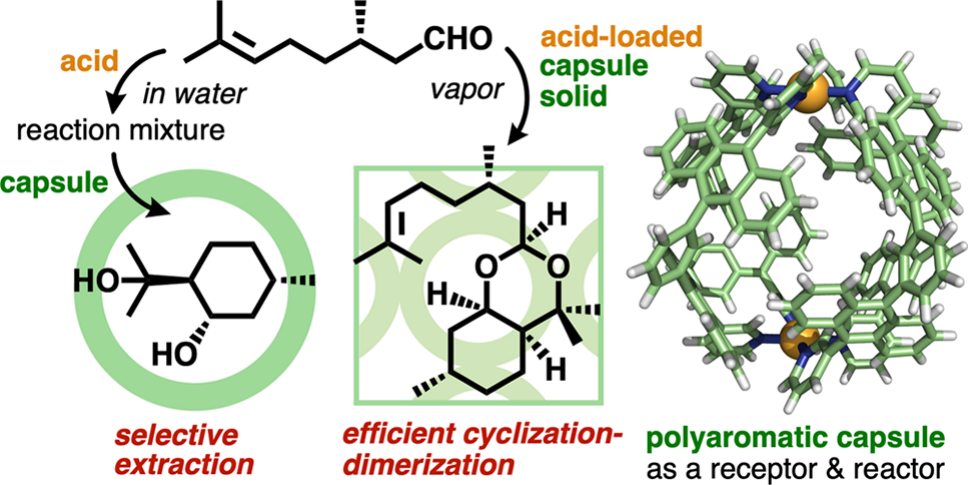

Linear monoterpenes, versatile reaction biosubstrates, are bound and subsequently converted to various cyclic monomers and oligomers with excellent selectivity and efficiency, *only* in natural enzymes. We herein report bioinspired functions of synthetic polyaromatic cavities toward linear monoterpenes in the solution and solid states. The cavities are provided by polyaromatic coordination capsules, formed by the assembly of Pt(II) ions and bent bispyridine ligands with two anthracene panels. By using the capsule cavities, the selective binding of citronellal from mixtures with other monoterpenes and its preferential vapor binding over its derivatives are demonstrated in water and in the solid state, respectively. The capsule furthermore extracts *p*-menthane-3,8-diol, with high product- and stereoselectivity, from a reaction mixture obtained by the acid-catalyzed cyclization of citronellal in water. Thanks to the inner and outer polyaromatic cavities, the catalytic cyclization-dimerization of vaporized citronellal efficiently proceeds in the acid-loaded capsule solid and product/stereoselectively affords *p*-menthane-3,8-diol citronellal acetal (∼330% yield based on the capsule) under ambient conditions. The solid capsule reactor can be reused at least 5 times with enhanced conversion. The present study opens up a new approach toward mimicking terpene biosynthesis via synthetic polyaromatic cavities.

## Introduction

Highly selective binding and conversion of biosubstrates are hallmarks of natural enzymatic reactions.^1^ These reactions occur in the isolated hydrophobic pockets of large protein assemblies under ambient aqueous conditions.^2^ Inspired by such functional biocavities, a large number of synthetic molecular tubes, cages, and capsules have been developed with the goal to mimic or even surpass such enzymatic reactions in vitro.^3,4^ However, the functions of the previous artificial systems are usually limited to *either* selective binding *or* conversion of the substrates, and generally studied in organic solvents under narrow conditions without reusability. Water-soluble synthetic cavities with dual functionality, i.e., displaying both selective binding and conversion abilities, are still rare to this date.

Linear monoterpenes are versatile reaction substrates in nature. They are bound and subsequently cyclized to various monomers and oligomers in protein pockets, with excellent selectivity and efficiency.^2^ These cyclic terpenes represent the largest class of natural products with the majority of them displaying bioactive properties.^5^ While there have been several reports on synthetic receptors capable of tightly binding linear monoterpenes, their binding selectivities are only low to moderate, owing to the flexible chain structures and scarce presence of interactive groups (i.e., −OH, C=O, and C=C).^6,7^ As for synthetic terpene reactors, the Raymond and Shionoya groups reported catalytic cyclization of citronellal using a Ga(III)-based coordination capsule and Pd(II)-based macrocycle solid, respectively, in water with high conversion (>70%) yet moderate stereoselectivity.^8^ The metal-hinges are essential to construct well-defined cavities yet inactive for the catalytic reactions. Efficient catalytic cyclization of geraniol and citronellal was achieved by the Tiefenbacher and other groups employing a hydrogen-bonded capsule in CHCl_3_ (∼100% conversion).^9,10^ Nevertheless, control over the product-selectivity of more complex cyclizations remains difficult. In addition, selective cascade *cyclization-dimerization* of linear monoterpenes has seldom been accomplished by the previous artificial systems, both in the solution and solid states.^11,12^

**Figure 1:**
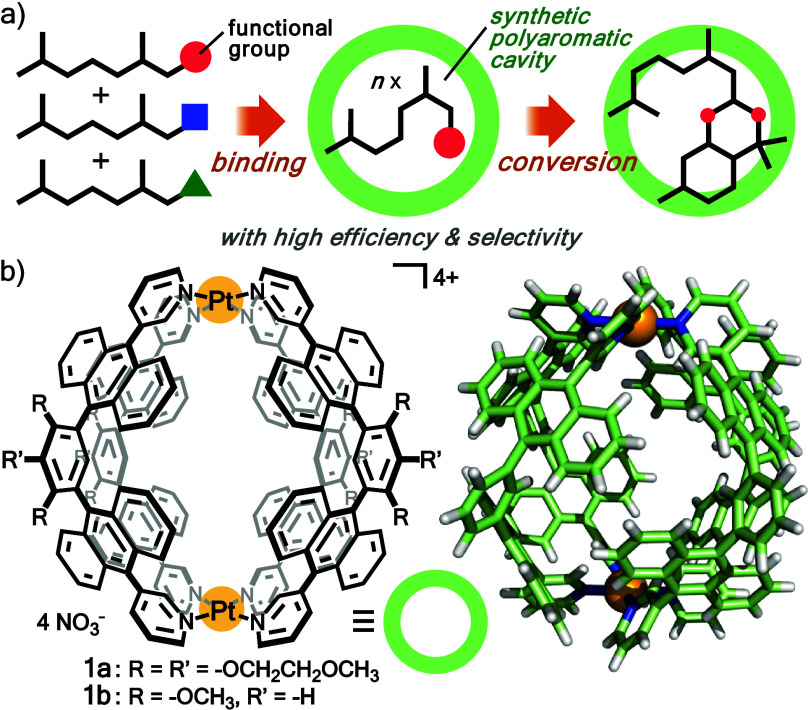
(a) Schematic representation of the binding and conversion of linear monoterpene(s) via a synthetic polyaromatic cavity with high efficiency and selectivity. (b) Polyaromatic coordination capsule **1** and its crystal structure (R = R′ = H for clarity).

To develop a biomimetic synthetic cavity with both receptor and reactor functions for linear monoterpenes, we herein used polyaromatic coordination capsules **1** (Figure 1),^13^ capable of selectively binding various biomolecules (e.g., caffeine, sucrose, and androgen) in water, through multiple CH-π, π–π, and/or hydrogen-bonding interactions.^13,14^ The capsule is prepared by the assembly of Pt(II) ions and bent bispyridine ligands with two anthracene panels. Regardless of the side chains (R = R′ = OCH_2_CH_2_OCH_3_ for **1a** and R = OCH_3_, R′ = H for **1b**), a large polyaromatic cavity (∼1.3 nm and ∼580 Å^3^) and four small windows (∼0.3 × 0.3 nm^2^) are provided both in solution and in the solid state. The assembled structures are stable enough even under acidic conditions, as compared with the isostructural derivatives (e.g., M = Pd(II), Ni(II), Cu(II), and Zn(II)).^13a,13b^ With the aid of these cavity features, here we report (i) highly selective binding (≥90%) of citronellal (**CAL**) by polyaromatic capsule **1a** from a mixture of linear/cyclic monoterpenes in water, (ii) preferential vapor binding capability (60–99% selectivity, >2 equiv. based on **1b**) of capsule solid (**1b**)_*n*_ toward **CAL** over other monoterpenes, (iii) product-selective extraction (>99% yield, 75% stereoselectivity) of *p*-menthane-3,8-diol (**PMD**) by the capsule from a product mixture derived from acid-catalyzed **CAL** cyclization in water, (iv) the efficient catalytic cyclization-dimerization of vaporized **CAL** into *p*-menthane-3,8-diol citronellal acetal, with high product/stereoselectivity (∼330% yield based on **1b**, ∼80% stereoselectivity), in the acid-loaded capsule solid under ambient conditions, and (v) the catalyst reusability (5 times) for the cyclization-dimerization reaction with gradually enhanced conversion (up to 1.4-fold). We therefore achieved both receptor and reactor functions toward linear monoterpenes utilizing a single unified synthetic design based on a polyaromatic cavity.

## Results and Discussion

### Selective Binding of Citronellal from Mixtures in Water

We initially revealed the selective binding ability of capsule **1a**, bearing hydrophilic, long side chains (R = R′ = OCH_2_CH_2_OCH_3_; Figure 1b), toward (*S*)-(−)-citronellal (**CAL**) in water from mixtures with its closely related derivatives. Unlike mono/bicyclic monoterpenes with rigid and unreactive frameworks studied previously,^15^**CAL** is a linear monoterpene, with a terminal formyl group and an internal double bond, found in plants such as citronella, lemon, and lemon grass.^2^**CAL** provides a relatively high reactivity against acid and generates a complex mixture of e.g., isopulegol and *p*-menthane-3,8-diol in solution.^16^ Synthetic receptors capable of selectively binding **CAL** and its acid-catalyzed cyclic products have been uncommon so far.^6^

**Figure 2:**
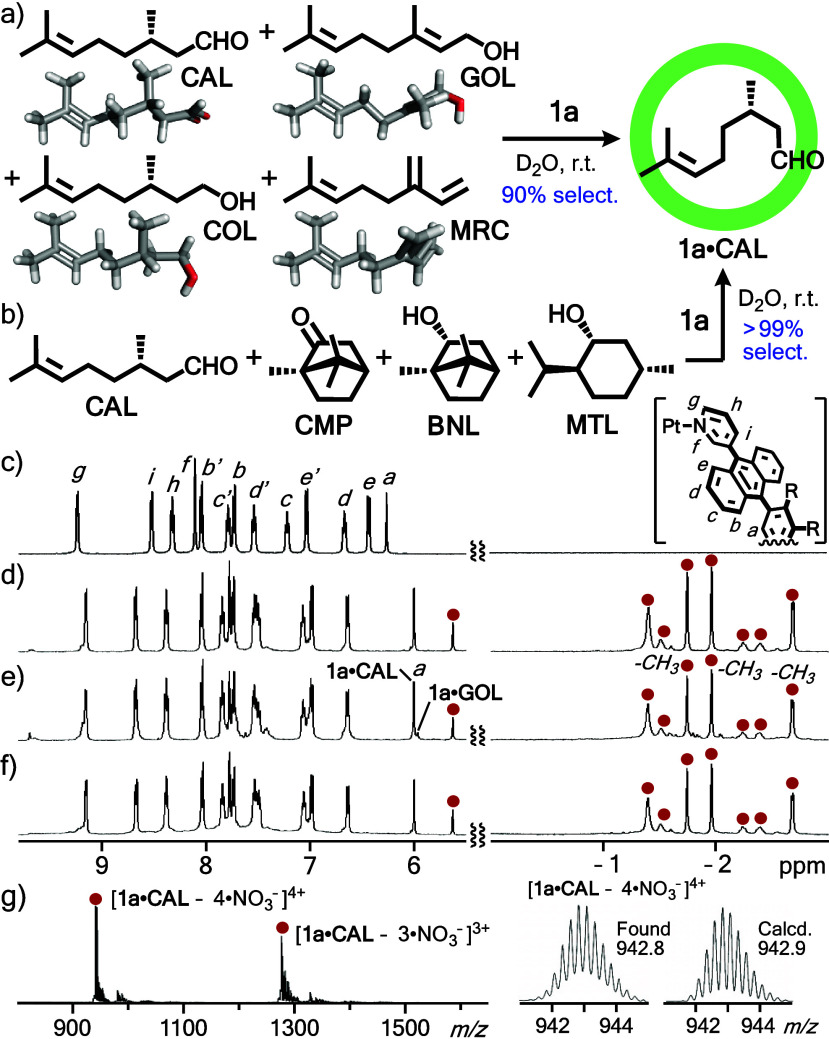
Solution-state selective binding of **CAL** by **1a** from (a) a mixture of **CAL**, **GOL**, **COL**, and **MRC**, and (b) a mixture of **CAL**, **CMP**, **BNL**, and **MTL** in water. ^1^H NMR spectra (500 MHz, D_2_O, r.t.) of (c) **1a** and (d) **1a**·**CAL**, and products from mixtures of **1a** with (e) **CAL**, **GOL**, **COL**, and **MRC** and (f) **CAL**, **CMP**, **BNL**, and **MTL** in water (red circle: proton signals derived from bound **CAL**). (g) ESI-TOF MS spectrum (H_2_O) of products from mixtures of **1a** with **CAL**, **GOL**, **COL**, and **MRC** in water.

Capsule **1a** bound one molecule of **CAL** in a quantitative fashion within 1 h through simple mixing of **1a** and **CAL** (4.0 equiv.) in water at room temperature, as confirmed by ^1^H NMR, ESI-TOF MS, and other analyses (Figures S1–S6 of the Supporting Information).^17^ The efficient capture stems from typical host–guest hydrophobic effect and CH-π interactions.^13^ When a complex mixture of **1a** (1.0 mg, 0.26 μmol), **CAL**, geraniol (**GOL**), (−)-β-citronellol (**COL**), and myrcene (**MRC**; 4.0 equiv. each based on **1a**) was stirred in D_2_O (0.5 mL) under the same conditions, host–guest complexes **1a**·**CAL** and **1a**·**GOL** were formed with 90 and 10% selectivity, respectively, with no free **1a** detectable (Figures 2a and S7–S13a). In the ^1^H NMR spectrum, the three methyl signals of **CAL** within **1a** appeared at −2.69, –1.97, and −1.75 ppm, which were highly upfield-shifted (Δ*δ*_max_ = −4.04 ppm) relative to free **CAL** in D_2_O, because of the anisotropy effect of the polyaromatic framework of **1a** (Figure 2c–e). The binding selectivity was estimated on the basis of the integral ratio of inner capsule signals *H*_a_ at ∼6.0 ppm. The ESI-TOF MS spectrum showed prominent signals corresponding to [**1a**·**CAL** – *n*·NO_3_^–^]^*n*+^ (*n* = 4 to 2) as the major product (Figures 2g and S13b).^17^ Other monoterpenes present in the mixture, like more hydrophilic **COL** and more hydrophobic **MRC**,^18^ were hardly bound by **1a** and therefore the observed high selectivity toward **CAL** is most probably derived from effective CH···O=C-based hydrogen-bonding interactions between the slightly acidic pyridyl α-hydrogens (*H*_f_) of **1a** and the carbonyl group of **CAL**. The host–guest hydrogen-bonding interactions within **1a** were supported by FT-IR analysis (Figure S5b).^17^

Binding selectivity of **1a** toward **CAL** was further clarified by a competitive experiment with bicyclic and monocyclic monoterpenes. Stirring a complex mixture of **CAL**, (−)-camphor (**CMP**), (−)-borneol (**BNL**), and (−)-menthol (**MTL**) in water under the same conditions described above led to the quantitative formation of **1a**·**CAL** (>99% selectivity and yield, Figure 2b). The selectivity was confirmed by the ^1^H NMR and ESI-TOF MS analyses of the product solution (Figures 2f and S14).^17^ These spectra are virtually identical to those of **1a**·**CAL** obtained from **1a** and **CAL** (Figure 2d). The ^1^H DOSY NMR of **1a**·**CAL** showed the host and guest signals with the same diffusion constant (Figure S2). The binding constant (*K*_a_) of **1a** toward **CAL** was estimated to be >3 × 10^5^ M^–1^, based on that toward **CMP** obtained by previously reported ITC studies.^15^ On the basis of these studies, the synthetic polyaromatic cavity of capsule **1a** was shown to provide unusual binding ability toward a monoterpene, featuring both a formyl group and a linear flexible framework, in water through effective CH···O=C hydrogen-bonding, hydrophobic effect, and CH-π interactions.

### Selective Vapor Binding of Citronellal from Mixtures

High volatility is one of the characteristic properties differentiating monoterpenes from the majority of biosubstrates. In contrast to host–guest studies in solution,^6^ those in the solid state targeting volatile linear monoterpenes have been relatively rare to this date. To assess solid-state binding selectivity toward vaporized **CAL**, capsule **1b** without long side chains (R = OCH_3_ and R′ = H; Figure 1b) was employed to increase the hydrophobicity of the space between the capsules in the solid state. Thus, the capsule solid possesses large polyaromatic surfaces (BET surface area: 172 m^2^ g^–1^) to effectively interact with substrates via not only the interior but also the exterior of the capsule framework.^21^ Competitive vapor binding experiments eventually revealed that porous capsule solid (**1b**)_*n*_ binds **CAL** (more than 2 equiv.) preferentially over other linear and cyclic monoterpenes even under ambient temperature and pressure. The binding preference is independent from the intrinsic vapor pressures of the monoterpenes (Figure S15).^18^

**Figure 3:**
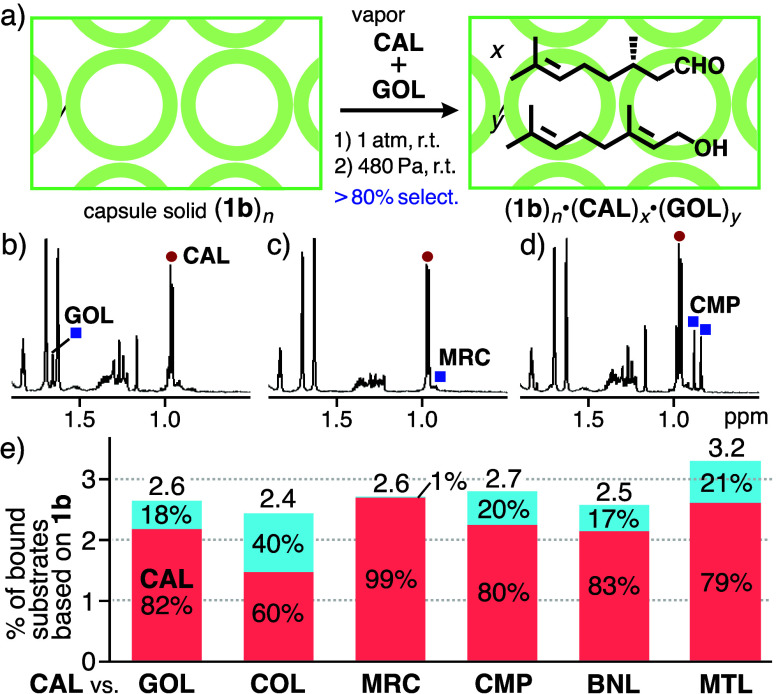
(a) Solid-state selective binding of vaporized **CAL** by solid (**1b**)_*n*_ from a vapor mixture with **GOL**. ^1^H NMR spectra (500 MHz, CD_3_CN, r.t.) of products after the competitive vapor binding of (b) **CAL** and **GOL**, (c) **CAL** and **MRC**, and (d) **CAL** and **CMP** by solid (**1b**)_*n*_ at r.t. for 2 h. (e) Host–guest ratios and guest % of the products after competitive vapor binding for **CAL** (red bar) and other monoterpenes (blue bar).

**CAL** and geraniol **(GOL**) (40 equiv. each based on **1b**) were separately put in a sealed glass vessel (50 mL) including pale yellow solid (**1b**)_*n*_ (0.6 mg, 0.19 μmol) at room temperature for 2 h (Figure 3a). After removal of the substrates *outside* the solid under vacuum (480 Pa, 1 h, room temperature), the binding capability of (**1b**)_*n*_ toward the substrate vapor was estimated by ^1^H NMR analysis. The bound **CAL** and other monoterpenes in the capsule solid remained intact even under the harsh conditions, due to effective host–guest interactions inside the solid. The proton spectrum of resultant solid (**1b**)_*n*_·(**CAL**)_*x*_·(**GOL**)_*y*_ in CD_3_CN showed sharp signals derived from dissociated **1b**, **CAL**, and **GOL** (Figure S16–21), owing to the lack of host–guest interactions in nonaqueous solution. The signal integrals indicated that a total of 2.6 molecules of the substrates based on **1b** were bound by the solid with 82% selectivity for **CAL** (Figure 3b,e). The **CAL**-binding selectivity of solid (**1b**)_*n*_ decreased against **COL** yet increased against **MRC** under the same conditions (−22% and +17% yield, respectively; Figure 3c,e and S22).^17^ These results suggest that the capsule solid can incorporate the monoterpenes into polyaromatic spaces inside and between the capsules, which slightly alters the binding selectivity relative to that of **1a** in water. Thus, it is noteworthy that the amount of the bound linear monoterpenes increased by ∼2–3-fold (based on the capsule), as compared with that of cyclic ones in our previous work,^15^ under similar conditions.

The vapor binding ability of solid (**1b**)_*n*_ toward **CAL** was higher than toward cyclic monoterpenes, i.e., **CMP**, **BNL**, and **MTL**. Solid product (**1b**)_*n*_·(**CAL**)_*x*_·(**CMP**)_*y*_ was obtained by placing (**1b**)_*n*_, **CAL**, and **CMP** in a 1:40:40 ratio in a sealed glass vessel (50 mL) at room temperature for 2 h. The ^1^H NMR spectrum of the product elucidated that 2.7 monoterpenes based on **1b** (i.e., *n* = 1.0, *x* = 2.2, *y* = 0.5) are captured by the capsule solid and the selectivity for **CAL** was estimated to be 80% (Figure 3d,e and S23). The vapor binding selectivity of (**1b**)_*n*_ toward **CAL** was virtually unchanged even from mixtures of **CAL** and **BNL** as well as **CAL** and **MTL** (≤ ± 3%; Figure 3e and S24–S25).

### Selective Extraction of Acid-Catalyzed Cyclization Products in Water

**Scheme 1:**
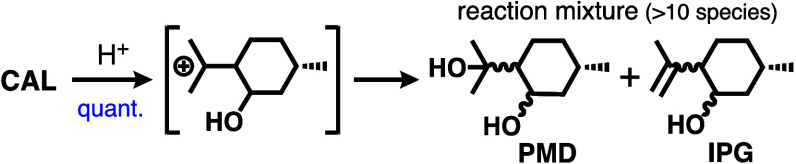
Formation of a Mixture Including Stereoisomers of **PMD** and **IPG** from **CAL** under Acidic Conditions

We next focused on the acid-catalyzed cyclization of citronellal (**CAL**) in solution and the binding properties of capsule **1a** (R = R′ = OCH_2_CH_2_OCH_3_) toward the complex reaction mixture.^15^**CAL** is readily converted to various cyclic monoterpenes, including mixed stereoisomers of isopulegol (**IPG**) and *p*-menthane-3,8-diol (**PMD**; Scheme 1), under acidic aqueous conditions.^16^ Therefore, the strict stereocontrol of the cyclization reaction remains difficult even under various conditions.^8^ We herein alternatively elucidated the product-selective as well as stereoselective extraction of **PMD** (i.e., its *equatorial* isomer *eq*-**PMD**) by **1a** from the reaction mixture in water.

**Figure 4:**
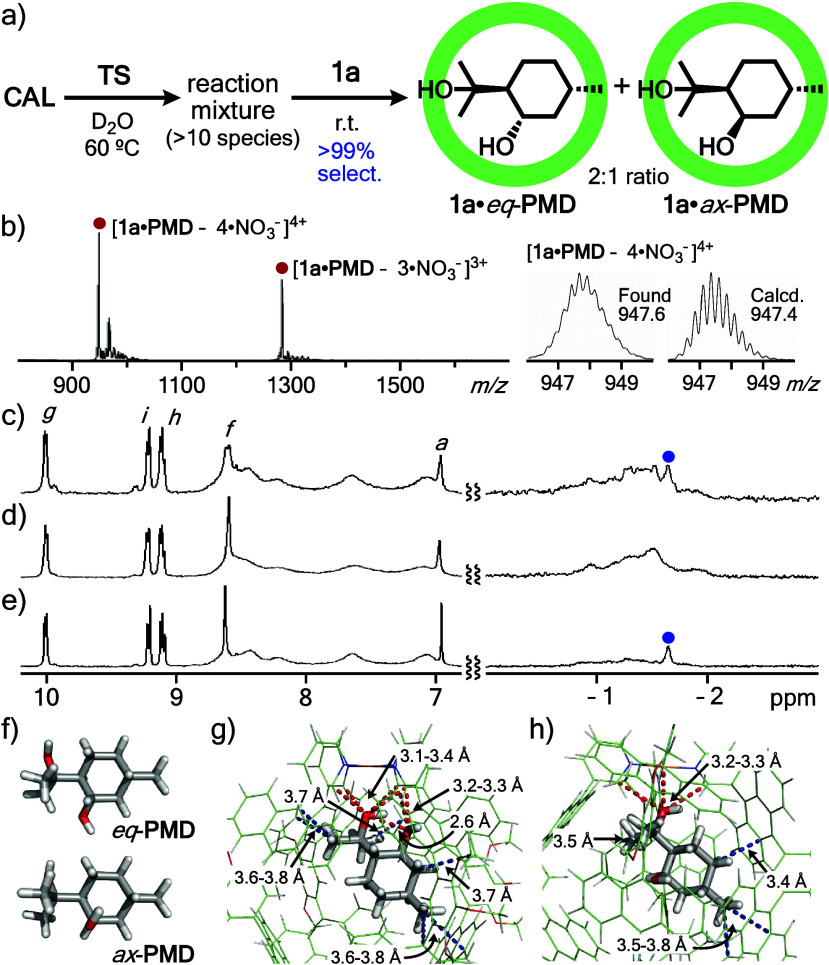
(a) Solution-state selective binding of *eq*-**PMD** by **1a** from a reaction mixture of **CAL** and *p*-toluenesulfonic acid (**TS**) in water. (b) ESI-TOF MS spectrum (H_2_O) of products from **1a** and a reaction mixture obtained from treatment of **CAL** with **TS**. ^1^H NMR spectra (400 MHz, D_2_O, 100 °C) of (c) products from **1a** and the reaction mixture, (d) **1a**·*eq*-**PMD** and (e) **1a**·*ax*-**PMD**. Optimized structures (PM6 calculation) of (f) *eq/ax*-**PMD**, and (g) **1a**·*eq*-**PMD** and (h) **1a**·*ax*-**PMD** (highlighted host–guest interactions: red dotted line: hydrogen-bonding interaction; blue dotted line: CH-π interaction).

The treatment of **CAL** (0.4 mg, 2.3 μmol) with *p*-toluenesulfonic acid (**TS**; 0.5 equiv.) in water (1.5 mL) at 60 °C (oil bath) for 1 h led to the formation of various cyclic monoterpenes and diterpenes (at least 10 species, Figure S26).^17,19^ When capsule **1a** (1.0 mg, 0.26 μmol) was added to the aqueous product mixture, the quantitative formation of host–guest complex **1a**·(**PMD**) (>99% yield) within 10 min at room temperature was revealed by NMR and MS analyses (Figure 4a). The ^1^H NMR spectrum showed relatively simple aromatic and aliphatic signals at room temperature (Figure S27a), which are fully different from those of **1a**·**CAL** (Figure 2e). The ESI-TOF MS spectrum displayed prominent molecular ion peaks at 947.6 and 1284.2, corresponding to [**1a**·**PMD** – *n*·NO_3_^–^]^*n*+^ (*n* = 4–3, Figure 4b). The stereochemistry of the bound cyclic product within **1a** was further verified by the temperature-dependent ^1^H NMR studies (e.g., 100 °C, Figure 4c-e), using **1a**·*eq*-**PMD** and **1a**·*ax*-**PMD**, prepared from *equatorial* and *axial***PMD** isomers with **1a** in water, respectively (Figures S27–S28).^20^ The proton signals and integrals indicated the product ratio being 2:1 **1a**·*eq*-**PMD/1a**·*ax*-**PMD** (63% stereoselectivity; Figure 4a).^19^ The major formation of **1a**·*eq*-**PMD** was also supported by the host signals (6.2–8.0 ppm) in the ^1^H NMR spectrum at room temperature (Figure S27b). Notably, the stereoselectivity was improved to 9:1 **1a**·*eq*-**PMD/1a**·*ax*-**PMD** (90% selectivity), when a 1:1 mixture of isolated *eq*-**PMD** and *ax*-**PMD** (3.0 equiv. each) was combined with **1a** under similar conditions (Figures S33–S35).^17^

The observed, high *equatorial* selectivity was supported by theoretical host–guest calculations (PM6, R = OCH_3_). Both *eq*-**PMD** and *ax*-**PMD** are fully accommodated in the spherical polyaromatic cavity of **1a** (Figure S36). Whereas the calculated energy of *eq*-**PMD** is 4.1 kJ mol^–1^ higher than that of *ax*-**PMD** without **1a**, the energy of **1a**·*eq*-**PMD** is 30.8 kJ mol^–1^ lower than that of **1a**·*ax*-**PMD** (Table S1; DFT, CAM-B3LYP-GD3BJ/LANL2DZ &6-31G(d,p) level). The large energy stabilization is most probably caused by multiple host–guest interactions in the cavity. In the optimized structure of **1a**·*eq*-**PMD**, the presence of six hydrogen-bonding interactions (3.1–3.4 Å) and six CH-π interactions (3.6–3.8 Å) are indicated on the basis of close interatomic distances (Figure 4g). In contrast, the model of **1a**·*ax*-**PMD** shows only four hydrogen-bonding and four CH-π interactions (Figure 4h), in spite of the small stereo difference.

### Catalytic Cyclization-Dimerization of Vaporized Citronellal

Taking advantage of the efficient and selective binding ability of capsule solid (**1b**)_*n*_ (R = OCH_3_ and R′ = H), we for the first time set out to develop a reactor function of the capsule solid toward vaporized **CAL**. Monoterpene conversion in the vaporized state was envisioned to circumvent certain problems observed in solution, particularly suppression of multistep and intermolecular reaction pathways.^16^ Solid (**1b**)_*n*_ is composed of amorphous polyaromatic assemblies bearing well-defined spherical cavities within the capsules and randomly distributed spaces between (**1b**)_*n*_.^21^ To generate reactive cation species from **CAL** in the polyaromatic cavities, acid-loaded capsule solid (**1b**)_*n*_·(**TS**)_*x*_ was used for the present studies, because the capsule framework as well as its metal-hinges provide no catalytic activity. In sharp contrast to the previous acid-catalyzed cyclization of **CAL** in solution,^8−10^ the acidic porous solid prompted catalytic cyclization-dimerization of vaporized **CAL** and yielded *p*-menthane-3,8-diol citronellal acetal (**MCA**) with high efficiency and product/stereoselectivity under ambient conditions.^22^

**Figure 5:**
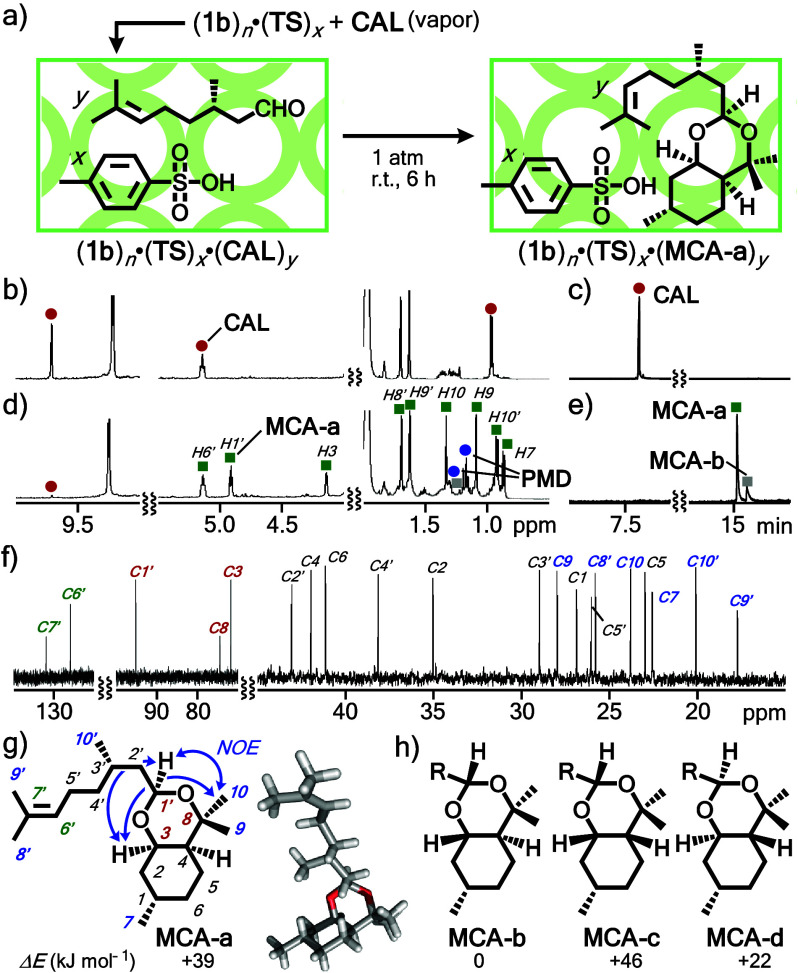
(a) Selective cyclization-dimerization of vaporized **CAL** into **MCA-a** within acid-loaded capsule solid (**1b**)_*n*_·(**TS**)_*x*_. (b) ^1^H NMR (500 MHz, CD_3_CN, r.t.) spectrum of (**1b**)_*n*_·(**CAL**)_*y*_ and (c) GC chart of **CAL**. (d) ^1^H NMR (500 MHz, CD_3_CN, r.t.) and (e) GC data of products after the cyclization-dimerization of **CAL** within solid (**1b**)_*n*_·(**TS**)_*x*_. (f) ^13^C NMR (125 MHz, CD_3_CN, r.t.) of isolated product **MCA-a**. The structures and energies of (g) **MCA-a** (with NOE correlations and the optimized structure) and (h) its conformational isomers **MCA-b**-**d**.

Solid (**1b**)_*n*_·(**TS**)_*x*_ was obtained by instant mixing (10 s) and solvent evaporation of **1b** (0.6 mg, 0.2 μmol) and *p*-toluenesulfonic acid (**TS**; 0.7 equiv.) in diethyl ether at room temperature.^17^ The incorporation of **TS** into solid (**1b**)_*n*_ was confirmed by ^1^H NMR analysis.^22^ The sequential vapor binding of **CAL** and its cyclization-dimerization occurred within (**1b**)_*n*_·(**TS**)_*x*_ at room temperature under ambient pressure (Figure 5a). Pale yellow solid (**1b**)_*n*_·(**TS**)_*x*_ (0.2 μmol based on **1b**) and **CAL** (1.5 mg, 10 μmol) were placed separately in a closed glass vessel (50 mL) for 6 h. The ^1^H NMR spectrum of resultant solid (**1b**)_*n*_·(**TS**)_*x*_·(**MCA-a**)_*y*_, dissolved in CD_3_CN, displayed new product signals in the range of 1–5 ppm, besides the methoxy signals derived from free capsule **1b** (Figures 5b,d and S37). Tiny proton signals of unreacted **CAL** (<10% based on **1b**) and *p*-menthane-3,8-diol (**PMD**) were also found in the NMR spectrum. The GC-MS spectrum of the acetonitrile solution showed a single prominent peak, derived from a monoterpene dimer, at 15.1 min GC retention time (Figure 5e), largely shifted compared to that of **CAL** at 8.1 min (Figure 5c). After isolation of main product **MCA** by gel permeation chromatography (GPC),^17^ its structure and stereochemistry were revealed by the combination of MS, NMR, and molecular modeling studies as follows (Figures S38–S46). The product mass was confirmed by ESI-TOF MS analysis and estimated to be 308.27 Da, indicating the formation of a dimeric compound (C_20_H_36_O_2_; Figure S45).^17^ The ^13^C NMR spectrum of **MCA** indicated a product containing 20 inequivalent carbon atoms, including one acetal carbon, two carbons connected to -OR groups, six methyl carbons, and nine carbons derived from a 2,6-dimethylhept-5-enyl group (Figure 5f). The ^1^H NMR and ^1^H–^1^H COSY analyses suggested the formation of two fused six-membered rings including an acetal unit (Figure S39). The combination of Nuclear Overhauser effects (NOEs) in the 1D NOESY spectra (i.e., correlation of protons H3–H10, H10–H1′, and H1′–H3; Figures 5g and S40), besides molecular modeling studies, revealed the formation of **MCA-a** as a single isomer. Capsule **1a** (as well as **1b**) provides a large enough, inner cavity to bind one molecule of **MCA-a** in a quantitative fashion (Figures S47–S49).

From the ^1^H NMR integrals of product solid (**1b**)_*n*_·(**TS**)_*x*_·(**MCA-a**)_*y*_ dissolved in CD_3_CN, the yield of cyclic dimer **MCA-a** was estimated to be 332% (TON = 3.3) based on capsule **1b**. The minor formation of isomer **MCA-b** (87%; Figure 5h) and **PMD** (60%) without **MCA-c/d** was also observed in the polyaromatic cavities of (**1b**)_*n*_·(**TS**)_*x*_. The substrate-based yields of products **MCA-a**, **MCA-b**, and **PMD** were calculated to be 14, 4, and 1% based on **CAL**, respectively, owing to the gas/solid-state reaction under ambient conditions. Weak signals for **MCA-b** and **PMD** were observed in the ^1^H NMR spectrum and GC chart (Figure 5d,e). These results clarified that the selective cyclization-dimerization of **CAL** took place within solid (**1b**)_*n*_·(**TS**)_*x*_ in a catalytic fashion, most likely triggered by the concentration effect as well as size and shape complementarity. In sharp contrast, neither cyclization nor dimerization of **GOL** and **COL** was observed within (**1b**)_*n*_·(**TS**)_*x*_ (Figure S50).^17^

**Figure 6:**
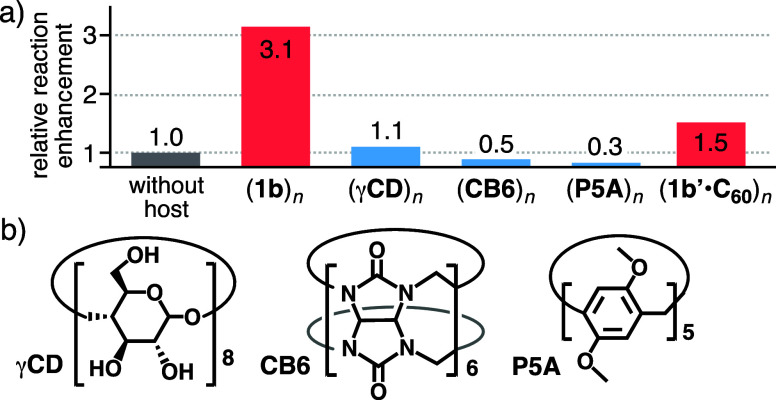
(a) The relative reaction enhancement of **TS**-loaded host and host–guest solids for the conversion of vaporized **CAL** into **MCA-a**, based on the background reaction. (b) Structures of organic hosts **γCD**, **CB6**, and **P5A**.

Both the well-defined polyaromatic cavity of solid (**1b**)_*n*_ and the loading of organic acid **TS** are essential for the present catalytic system. The metal-hinges and polyaromatic frameworks of **1b** have no catalytic activity toward **CAL**. In contrast, the treatment of vaporized **CAL** with solid **TS** without (**1b**)_*n*_ yielded **MCA-a** in 108% virtually based on **1b** under the same conditions as described above. This control experiment revealed that the reactivity of solid (**1b**)_*n*_·(**TS**)_*x*_ for the cyclization-dimerization of **CAL** is 3.1-fold higher than the background reaction (Figure 6a). Solid (**1b**)_*n*_·(**TS**)_*x*_ with a 1:0.7 **1b**/**TS** ratio acted as a superior catalyst, as compared with those with 1:0.4, 1:1.2, and 1:2 **1b**/**TS** ratios (Figure S51a). The catalytic activities of capsule solids (**1b**)_*n*_, loading benzoic, benzenesulfonic, or benzenephosphonic acids (0.7–1.2 equiv. based on **1b**), toward **CAL** were lower than that of (**1b**)_*n*_·(**TS**)_*x*_ (<0.2-fold; Figure S51b). As compared with the background reactivity, other acid-loaded solids comprising **TS** and common organic macrocycles, such as γ-cyclodextrin (**γCD**), cucurbit[6]uril (**CB6**), and a pillar[5]arene derivative (**P5A**; Figure 6b), showed only similar to lower reactivities (i.e., 0.3 to 1.1-fold; Figures 6a and S52–S53). In these reactions, **MCA-b** was yielded as a byproduct in ∼20% yield (based on the GC data; Figure S54), indicating poor reactivity on the outer surface of acid-loaded solids. The importance of the inner and outer polyaromatic cavities was further supported by using an analogous blocked capsule including one molecule of fullerene C_60_ (**C**_**60**_). Acid-loaded 1:1 host–guest solid (**1b′**·**C**_**60**_)_*n*_·(**TS**)_*x*_, prepared from capsule **1b′** (M = Pd(II)), **C**_**60**_, and **TS**, afforded **MCA-a** and **MCA-b** in 163% (TON = 1.6) and 39% yield based on **1b′**, respectively, under the same conditions (Figure 6a).^17^ It should be noted that, under neat conditions without (**1b**)_*n*_, the acid-catalyzed reaction of **CAL** using trifluoroacetic acid gave rise to a complex mixture, including a tiny amount of **MCA** (7% based on **CAL**; Figure S38). Transformation of volatilized monoterpenes was thus successfully shown to be an important strategy for efficiently accessing otherwise difficult-to-obtain product species.

### Reusability of the Capsule Solid for the Catalytic Cyclization-Dimerization Reaction

**Figure 7:**
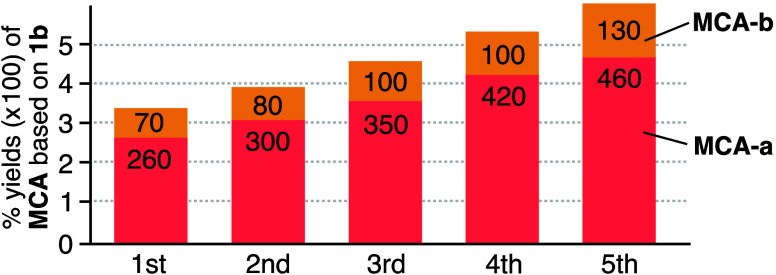
Reusability of solid (**1b**)_*n*_·(**TS**)_*x*_ for the conversion of **CAL** to **MCA**.

Porous capsule solid (**1b**)_*n*_ could be reused for the acid-catalyzed cyclization-dimerization of vaporized **CAL** at least five times with gradually enhanced conversion. After the first reaction under the same conditions (room temperature, 6 h) described above, the resultant solid (**1b**)_*n*_·(**TS**)_*x*_·(**MCA-a**)_*y*_ was washed with diethyl ether and CH_3_CN to selectively remove and isolate **MCA-a** from the cavity, recovering a mixed solid consisting of (**1b**)_*n*_·(**TS**)_*x*_ and (**1b**)_*n*_. The product structure and yield were analyzed by ^1^H NMR spectroscopy. After the reloading of **TS** into the capsule solid, regenerated solid (**1b**)_*n*_·(**TS**)_*x*_ was subjected to the next reaction cycle, to confirm the reusability of the capsule solid. The ^1^H NMR spectra of the extracted solutions displayed that the yields of cyclic dimer **MCA-a** increased gradually and reached 457% based on **1b** (TON = 4.6, 19% based on **CAL**) at the fifth reaction cycle (1.4-fold enhancement based on **1b**; Figures 7 and S55–S56). The stereoselectivity of product **MCA-a** remained intact throughout the reusability studies. The observed enhanced conversion is most probably caused by the gradual accumulation of *p*-toluenesulfonate ions in the capsule solid, through the counterion exchange with the nitrate ions (Figure S57), which could alter the space features between the capsules. In each cycle, **MCA-b** and **PMD** formed in 70–133 and 98–136% yields, respectively (based on **1b**; Figures 7 and S56). Whereas the detailed reaction mechanism is unclear,^23^ time-dependent ^1^H NMR study suggested a rapid stepwise cascade reaction from **CAL** to **MCA** in the capsule solid, due to the observation of minor **PMD** signals during the reaction (Figure S37b). No product inhibition by **MCA-a** was observed, probably due to the high volatility and flexibility of **CAL**. The high inertness and sufficient structural stability of the polyaromatic capsule framework under the repeated, acid-catalyzed reaction conditions was supported by the ^1^H NMR spectra (Figure S55), successfully preventing alkylation of the host, an undesired side reaction observed in one of the previous catalytic systems.^9^

Finally, the cyclization-dimerization reactions from a vapor mixture of **CAL** and geraniol (**GOL**) were examined using acid-loaded capsule solid (**1b**)_*n*_·(**TS**)_*x*_ and its reused species under ambient conditions. After separately putting solid (**1b**)_*n*_·(**TS**)_*x*_ (0.2 μmol based on **1b**), **CAL** (8.7 μmol), and **GOL** (8.7 μmol) in a closed vessel for 6 h, the ^1^H NMR analysis of the resultant solid revealed the formation of cyclic dimers **MCA** (165% based on **1b**) and cyclic monomers **PMD** (93%; Figure S58).^24^ The same reaction using acid-loaded, reused solid (**1b**)_*n*_·(**TS**)_*x*_ gave rise to **MCA** (210%) and **PMD** (117%). Notably, the bound **GOL** (0.5–0.6 equiv. based on **1b**) showed neither conversion nor decomposition. Accordingly, the present cyclization-dimerization of **CAL** proceeded sufficiently in the acid-loaded capsule solid, even from the mixed vapor of **CAL** and its derivative.

## Conclusions

Inspired by functional biocavities with highly selective binding and conversion of substrates, we have revealed new cavity functions of polyaromatic coordination capsules toward highly volatile and flexible, linear monoterpenes in the solution and solid states. In aqueous solution, the capsule preferentially bound one molecule of citronellal in the cavity, from complex mixtures with other linear/cyclic monoterpenes. From a reaction mixture of its acid-catalyzed cyclization, the capsule quantitatively bound *p*-menthane-3,8-diol with high *equatorial-*stereoselectivity to generate the corresponding 1:1 host–guest complex. We expect that such binding functions will lead to the facile extraction of minor yet valuable terpenes from natural plants, which contain a wide range of derivatives. In the solid state, the capsule furthermore exhibited preferential vapor binding ability toward citronellal (more than two molecules) from mixtures with other linear/cyclic monoterpenes under ambient conditions. Most importantly, the acid-loaded capsule solid, facilely prepared from the polyaromatic capsule and an organic acid, promoted the catalytic cyclization-dimerization reaction of vaporized citronellal with high efficiency, selectivity, and reusability. The herein demonstrated dual functionality of both selective binding and efficient conversion has rarely been demonstrated with other synthetic cavities reported previously. In contrast to flexible biocavities composed of large protein assemblies and rigid synthetic cavities composed of inorganic frameworks, the present polyaromatic cavities provide both flexible and rigid features as well as biological interactions, such as hydrogen-bonding, hydrophobic effect, and CH-π interactions. Therefore, we expect that the present study will promote novel approaches toward bioinspired terpene biosynthesis in both solution and vapor states using synthetic polyaromatic cavities.

## Methods

The following analytical instruments and software were used in this study. NMR: Bruker AVANCE III HD 500 (500 MHz) and AVANCE III 400 (400 MHz, TMS (δ = 0.00 ppm) in CDCl_3_ was used as an external standard for host–guest studies in D_2_O). ESI-TOF MS: Bruker micrOTOF II. GC-MS: SHIMADZU GCMS-QP2010. FT-IR (ATR): SHIMADZU IRSprit. GPC: LaboACE LC-5060 Plus II. PM6 and DFT calculations: Gaussian 16 program (Rev. C.01) package. Molecular mechanics calculation: Forcite module, BIOVIA Materials Studio 2020, version 20.1.0.5 (Dassault Systèmes Co.).

## Data Availability

The data underlying this study are available in the published article and its Supporting Information.

## Supporting Information Available

Experimental procedures, and NMR, MS, IR, GC, and calculation data (PDF)
