# Supplementary material for: Bioinspired Binding and Conversion of Linear Monoterpenes by Polyaromatic Coordination Capsules
Source: ACS Org Inorg Au. 2024 May 16;4(4):410–7. doi: 10.1021/acsorginorgau.4c00013 (PMC11311458; doi:10.1021/acsorginorgau.4c00013)
Supplement: Supplementary file 1 — gg4c00013_si_001.pdf [file gg4c00013_si_001.pdf]

Supporting Information

Bioinspired Binding and Conversion of Linear Monoterpenes by Polyaromatic Coordination Capsules

Ryuki Sumida, Lorenzo Catti, and Michito Yoshizawa*

Laboratory for Chemistry and Life Science, Institute of Innovative Research, Tokyo Institute of Technology, 4259 Nagatsuta, Midori-ku, Yokohama 226-8503, Japan
E-mail: yoshizawa.m.ac@m.titech.ac.jp

Contents

- Materials and General Methods, Calculations, References, and Abbreviation
- Synthesis of capsule **1b**
- Formation of **1a•CAL** (^{1}H NMR, ^{1}H DOSY, ^{1}H-^{1}H COSY, HSQC, ESI-TOF MS, and IR spectra)
- Formation of **1a•GOL**, **1a•COL**, and **1a•MRC** (^{1}H NMR, ^{1}H-^{1}H COSY, and/or ESI-TOF MS spectra)
- Competitive binding of **CAL**/**GOL**/**COL**/**MRC** and **CAL**/**CMP**/**BNL**/**MTL** by **1a** (^{1}H NMR and ESI-TOF MS spectra)
- Water solubility and vapor pressure of monoterpenes
- Vapor binding of **CAL**, **GOL**, **COL**, **MRC**, and **PRA** by solid (**1b**)$_n$ (^{1}H NMR spectra and optimized structures)
- Competitive vapor binding of **CAL** and **GOL**/**COL**/**MRC**/**CMP**/**BNL**/**MTL** by solid (**1b**)$_n$ (^{1}H NMR spectra)
- Formation of **1a•PMD** from **1a** and a reaction mixture of **CAL** (GC chart, ^{1}H NMR and ESI-TOF MS spectra)
- Synthesis of *eq*-**PMD** and *ax*-**PMD** (GC charts, ^{1}H NMR spectra, and optimized structures)
- Formation of **1a•***eq/ax*-**PMD** from **1a** and isolated *eq/ax*-**PMD** (^{1}H NMR and ESI-TOF MS spectra, optimized structures)
- Acid-catalyzed reaction of **CAL** within solid (**1b**)$_n$•(**TS**)$_x$ (^{1}H NMR and GC-MS spectra)

- Synthesis and structural analysis of **MCA-a** (^{1}H NMR, ^{1}H-^{1}H COSY, 1D NOESY, ^{13}C NMR, HMBC, HSQC, and ESI-TOF MS spectra, and optimized structures)
- Formation of **1a•MCA-a** (^{1}H NMR and ESI-TOF MS spectra, optimized structures)
- Acid-catalyzed reactions of **CAL** (^{1}H NMR spectra and GC charts)
- Acid-catalyzed reaction of **CAL** using **(1'•C$_{60}$)**$_n$ or other hosts (^{1}H NMR spectra and GC-MS charts)
- Repeated acid-catalyzed reaction of **CAL** within solid **(1b)**$_n$•**(TS)**$_x$ (^{1}H NMR and FT-IR spectra)
- Acid-catalyzed reaction from mixtures by solid **(1b)**$_n$•**(TS)**$_x$ (^{1}H NMR spectra)
- Theoretical calculation data of ***ax*/*eq*-PMD**, **PMD**, **MCA-a-d**, **1a**, **1a•CAL**, **1a•(CAL)**$_2$, **1a•*eq*/*ax*-PMD**, and **1a•MCA-a-d**

Materials and General Methods

NMR: Bruker AVANCE III HD 500 (500 MHz) and AVANCE III 400 (400 MHz, TMS (δ = 0.00 ppm) in $CDCl_3$ was used as an external standard for host-guest studies in D_2O), ESI-TOF MS: Bruker micrOTOF II, GC-MS: SHIMADZU GCMS-QP2010, FT-IR (ATR): SHIMADZU IRSprit, GPC: LaboACE LC-5060 Plus II.

Solvents and reagents: TCI Co., Ltd., FUJIFILM Wako Chemical Co., Kanto Chemical Co., Inc., Sigma-Aldrich Co., and Cambridge Isotope Laboratories, Inc. Compounds: Polyaromatic capsules **1a**, **1b**, and **1b'** were synthesized according to previously reported procedures.[S1,2] Structural assignments were made with additional information from COSY, HSQC, HMBC, and NOESY experiments.

Calculations

PM6 and DFT calculations: Gaussian 16 program (Rev. C.01) package, Molecular mechanics calculation: Forcite module, BIOVIA Materials Studio 2020, version 20.1.0.5 (Dassault Systèmes Co.). PM6 calculations of **PMD**, **MCA**, capsule **1a** (R = -OCH_3) and its host-guest complexes were performed for the geometry optimizations. DFT calculations of **PMD**, **MCA**, capsule **1a** (R = -OCH_3), and its host-guest complexes were performed for single point calculations (CAM-B3LYP+GD3BL/LanL2DZ (Pt), 6-31G(d,p) (others) level of theory with the PCM continuum solvent method (H_2O)). No imaginary frequency was obtained from the optimized structures.

References

[S1] a) N. Kishi, Z. Li, K. Yoza, M. Akita, M. Yoshizawa, *J. Am. Chem. Soc.* **2011**, *133*, 11438–11441; b) Z. Li, N. Kishi, K. Yoza M. Akita, M. Yoshizawa, *Chem. Eur. J.* **2012**, *18*, 8358–8365.

[S2] M. Yamashina, Y. Sei, M. Akita, M. Yoshizawa, *Nat. Commun.* **2014**, *5*, 4662.

[S3] Hazardous Substances Data Bank: a) citronellal, https://pubchem.ncbi.nlm.nih.gov/source/hsdb/594; b) citronellol, https://pubchem.ncbi.nlm.nih.gov/source/ hsdb/6805; c) geraniol, https://pubchem.ncbi.nlm.nih.gov/source/hsdb/484; d) myrcene, https://pubchem.ncbi.nlm.nih.gov/source/hsdb/1258.

[S5] a) Sigma-Aldrich, MSDS, (–)-menthone.

[S6] Tokyo Chemical Industry Co., Ltd., SDS, (–)-menthol.

[S7] TGSC Information System, (–)-borneol, http://www.thegoodscentscompany.com/data/rw1011571.html.

[S8] Y. Yuasa, H. Tsuruta, Y. Yuasa, *Org. Process Res. Dev.* **2000**, *4*, 159–161.

Abbreviation

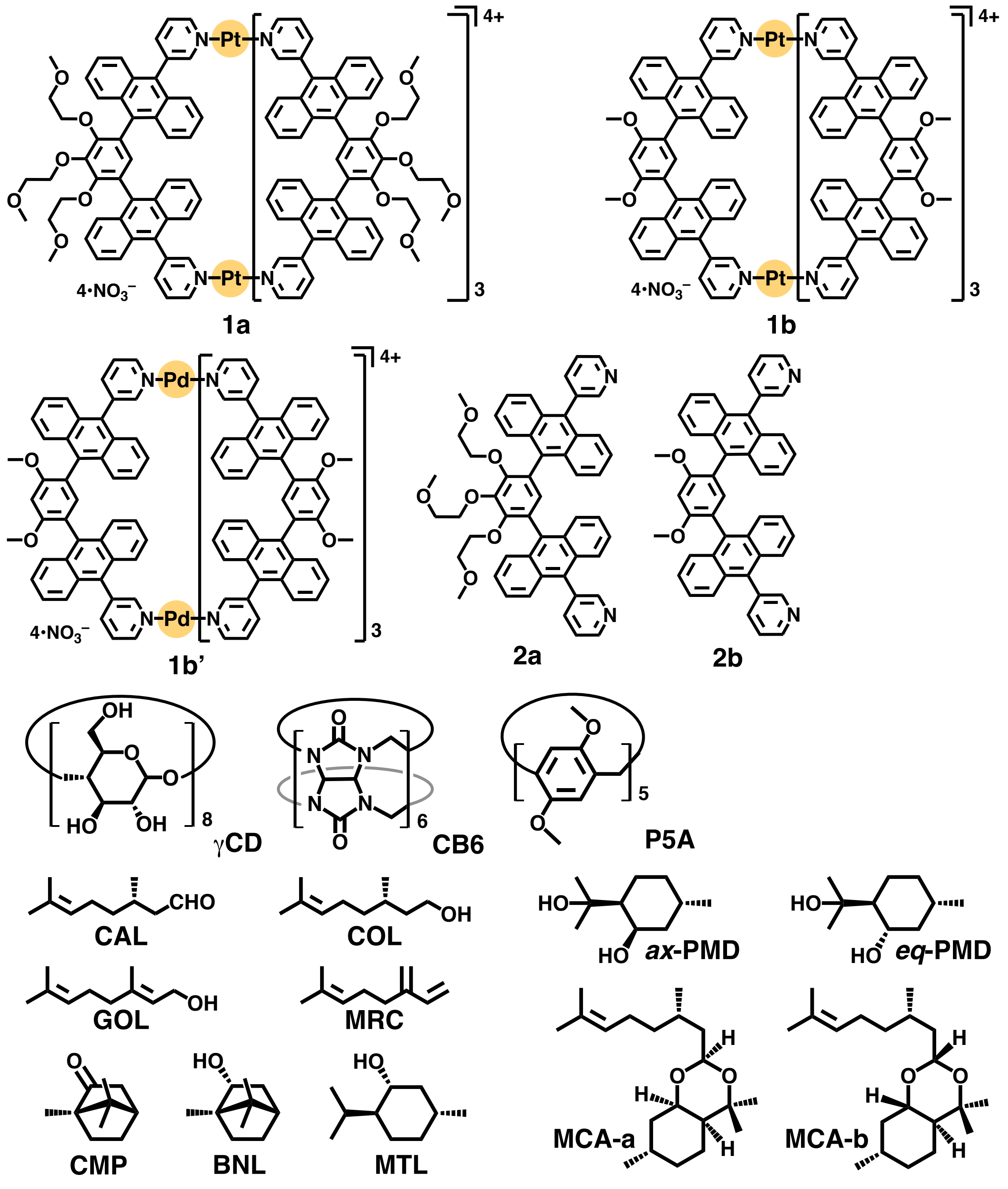

Synthesis of capsule 1b RS860

Ligand **2b** (20.1 mg, 31.1 μmol), $PtCl_2(CH_3CN)_2$ (6.2 mg, 17.8 μmol), $AgNO_3$ (6.2 mg, 36.5 μmol), and DMSO-d_6 (0.4 mL) were added to a glass test tube. When the mixture was stirred at 110 °C for 4 h, the quantitative formation of capsule **1b** was confirmed by ^{1}H NMR analysis. After filtration, resultant solution was added to an Et_2O solution. The precipitated product was collected and dissolved in CH_2Cl_2. Again, the resultant solution was added to a hexane solution to obtain pale-yellow amorphous solid **1b** (21.6 mg, 6.71 μmol; 86%).[S1,2] The product structure was confirmed by ^{1}H NMR and ESI-TOF MS analyses. Capsule **1a** was prepared in the same way.[S1,2]

Encapsulation of CAL by 1a RS935, 942, 943, 944

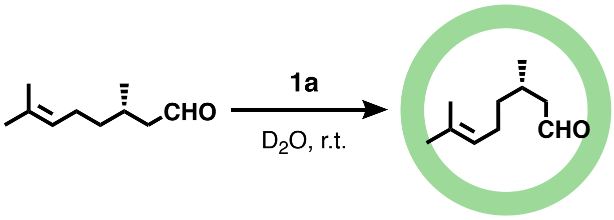

Capsule **1a** (1.0 mg, 0.26 μmol) and (S)-(–)-citronellal (**CAL**; 0.2 mg, 1.0 μmol) were added to a 2 mL test tube containing D_2O (0.5 mL). The mixture was stirred at r.t. for 1 h. The quantitative formation of pale-yellow 1:1 host-guest complex **1a•CAL** was confirmed by NMR and ESI-TOF MS analyses. In the same way, pale-yellow 1:1 host-guest complexes **1a•GOL**, **1a•COL**, and **1a•MRC** were obtained in water and the structures were confirmed by NMR and ESI-TOF MS analyses.

^{1}H NMR (500 MHz, D_2O, r.t.): δ –2.69 (d, J = 5.7 Hz, 3H, **CAL**), –2.40 (br, 1H, **CAL**), –2.25 (br, 1H, **CAL**), –1.97 (s, 3H, **CAL**), –1.75 (s, 3H, **CAL**), –1.52 (br, 1H, **CAL**), –1.41 (br, 4H, **CAL**), 2.52 (m, 24H, **1a**), 2.87 (br, 1H, **CAL**), 3.12 (m, 16H, **1a**), 3.51 (s, 12H, **1a**), 3.97 (m, 8H, **1a**), 4.06 (m, 8H, **1a**), 4.14 (m, 8H, **1a**), 4.51 (m, 4H, **1a**), 4.63 (m, 4H, **1a**), 5.62 (s, 1H, **CAL**), 5.99 (s, 4H, **1a**), 6.62 (d, J = 8.7 Hz, 8H, **1a**), 6.97 (d, J = 8.9 Hz, 8H, **1a**), 7.06 (dd, J = 7.5, 7.4 Hz, 8H, **1a**), 7.50 (m, 16H, **1a**), 7.73 (d, J = 8.9 Hz, 8H, **1a**), 7.77 (s, 8H, **1a**), 7.84 (dd, J = 7.3, 7.8 Hz, 8H, **1a**), 8.03 (d, J = 8.7 Hz, 8H, **1a**), 8.37 (dd, J = 6.8, 6.9 Hz, 8H, **1a**), 8.66 (d, J = 7.8 Hz, 8H, **1a**), 9.15 (d, J = 5.7 Hz, 8H, **1a**). ^{1}H DOSY NMR (500 MHz, D_2O, 25 °C): $D = 4.31 \times 10^{-10}$ m^2 s^{-1}. ESI-TOF MS (H_2O): m/z 1947.6 [**1a•CAL** – 2•NO_3^-]$^{2+}$, 1277.7 [**1a•CAL** – 3•NO_3^-]$^{3+}$, 942.8 [**1a•CAL** – 4•NO_3^-]$^{4+}$.

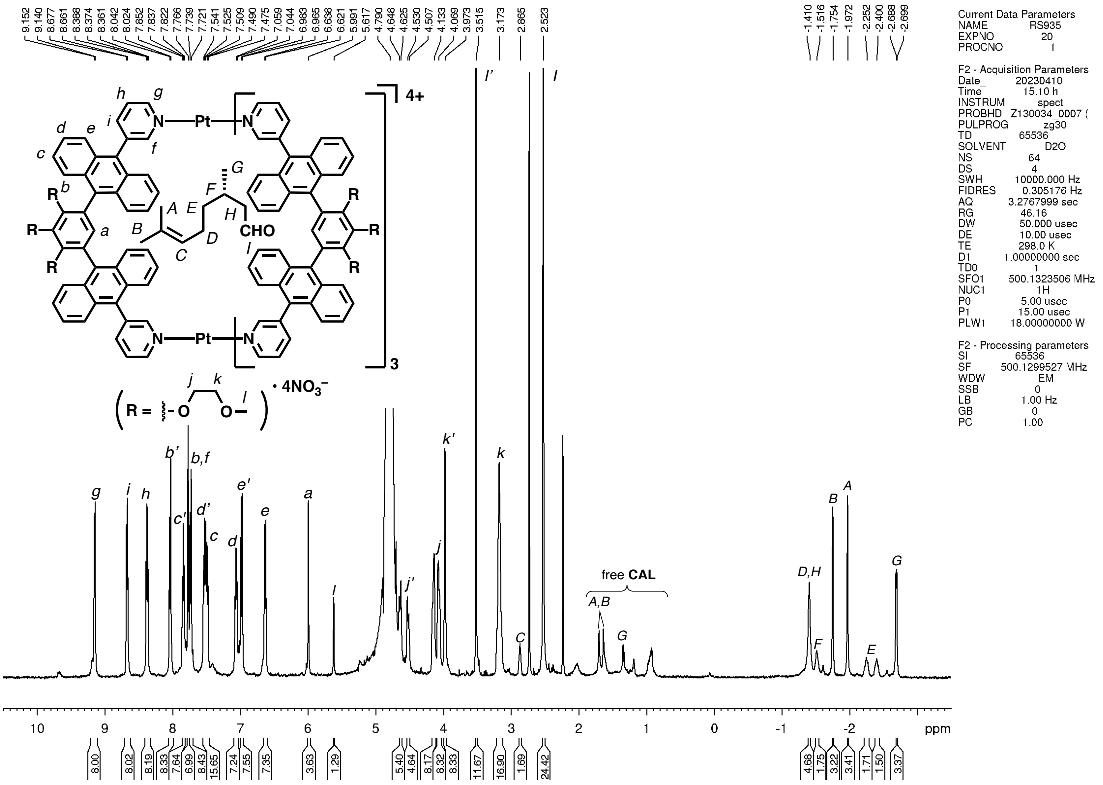

Figure S1. ^{1}H NMR spectrum (500 MHz, D_2O, r.t.) of **1a•CAL**.

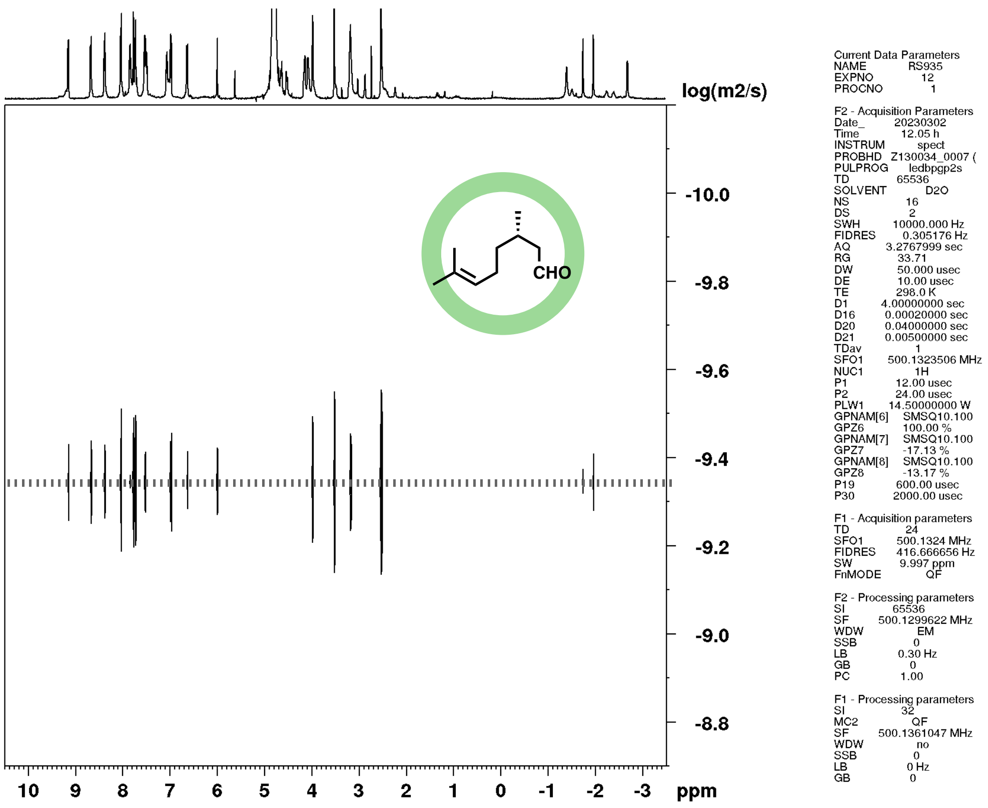

Figure S2. ^{1}H DOSY NMR spectrum (500 MHz, D_2O, 298 K) of **1a•CAL**.

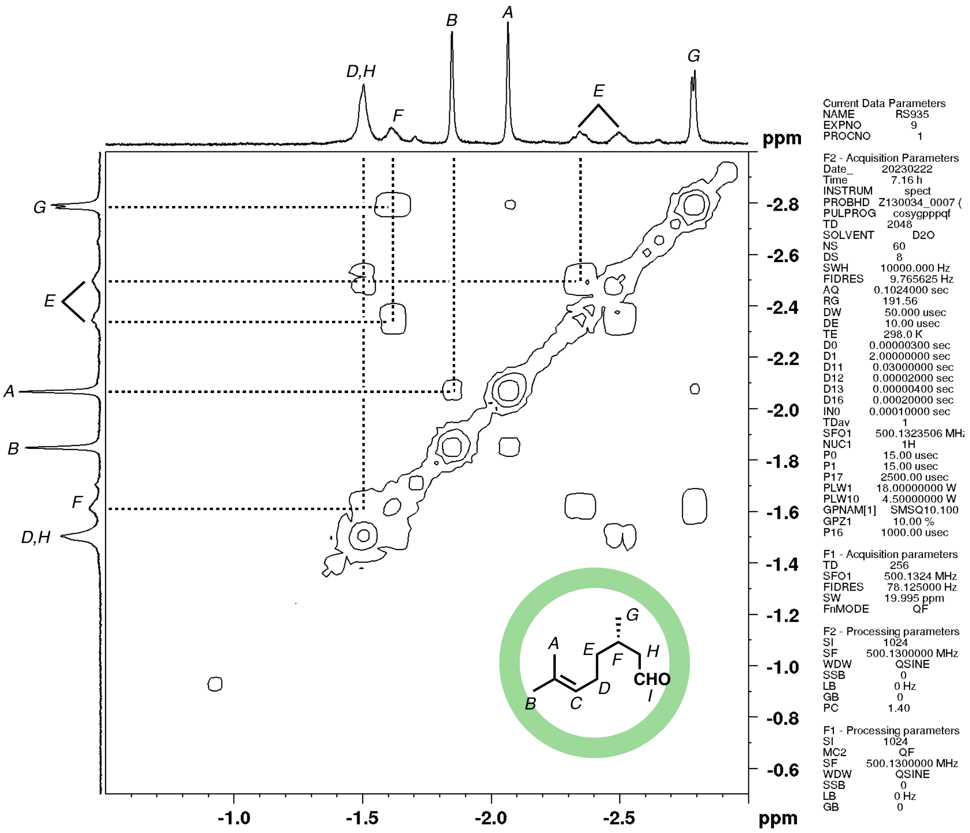

Figure S3a. ^{1}H-^{1}H COSY NMR spectrum (500 MHz, D_2O, r.t.) of **1a•CAL**.

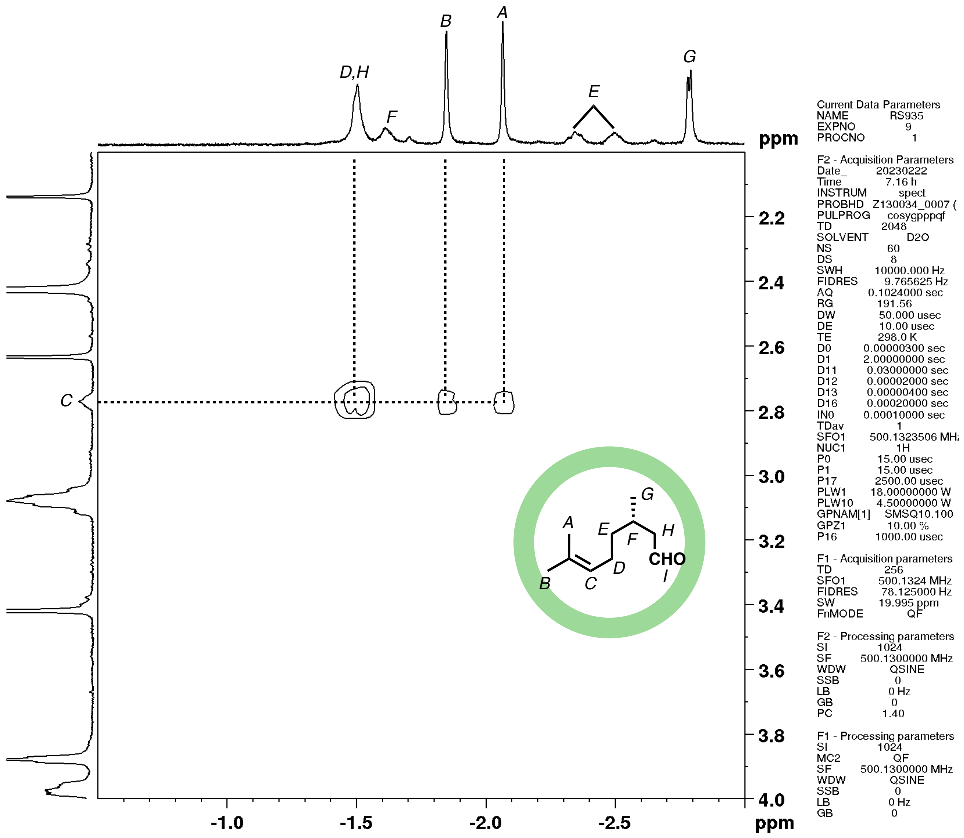

Figure S3b. ^{1}H-^{1}H COSY NMR spectrum (500 MHz, D_2O, r.t.) of **1a•CAL**.

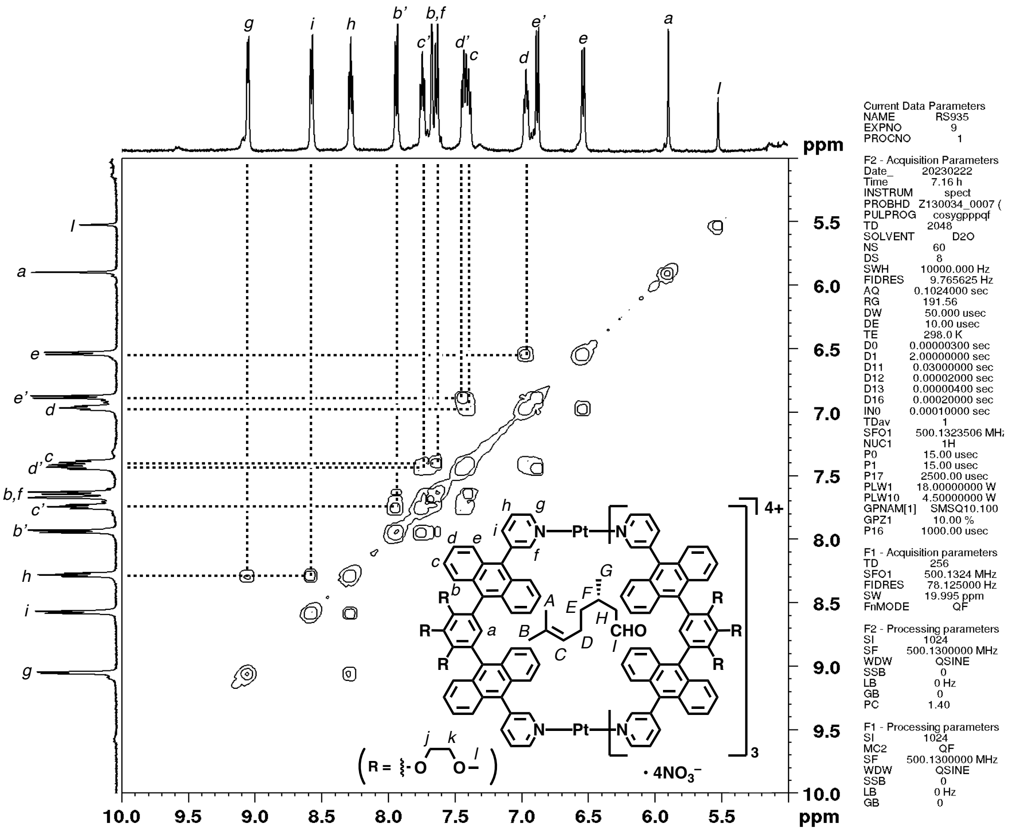

Figure S3c. ^{1}H-^{1}H COSY NMR spectrum (500 MHz, D_2O, r.t.) of **1a•CAL**.

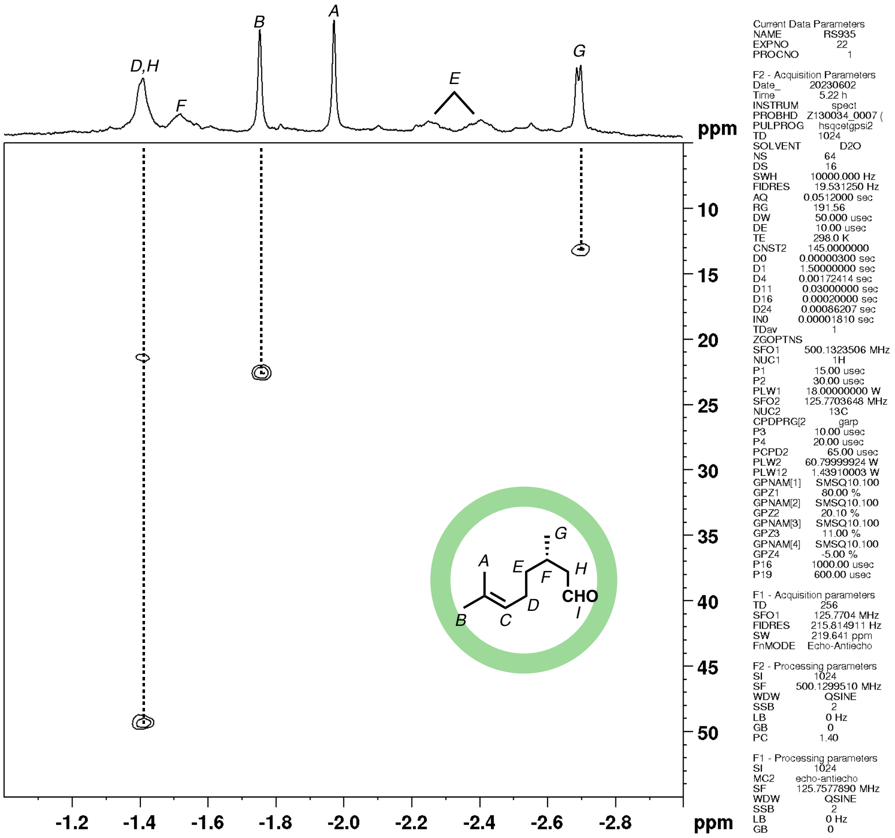

Figure S4a. HSQC NMR spectrum (500 MHz, D_2O, r.t.) of **1a•CAL**.

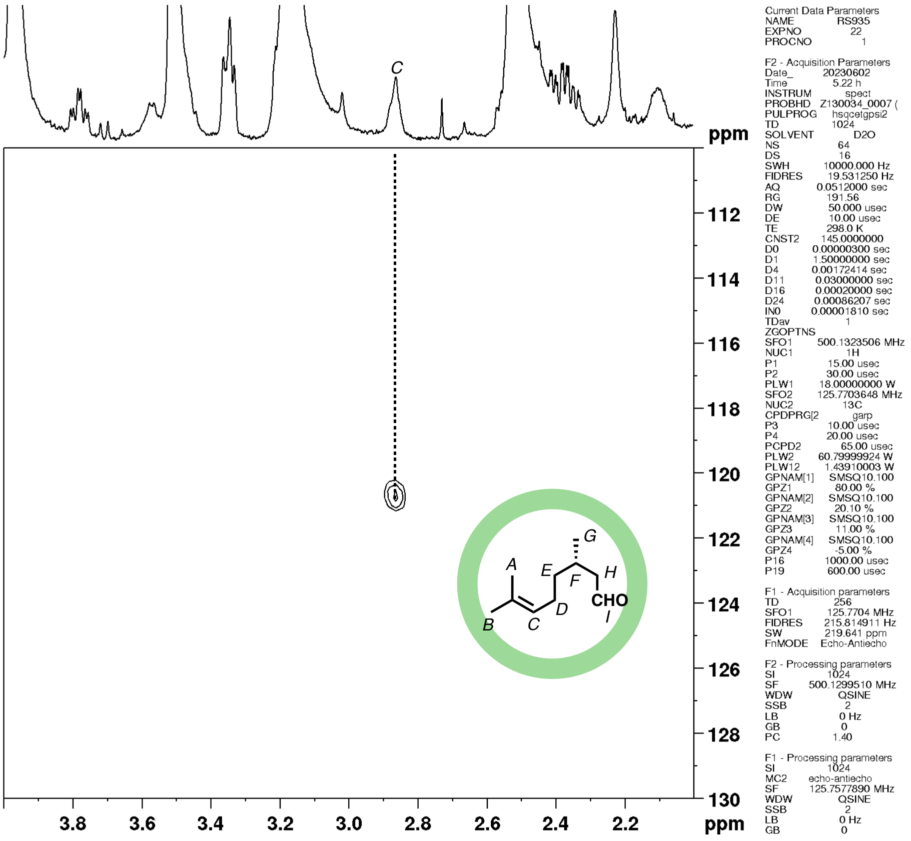

Figure S4b. HSQC NMR spectrum (500 MHz, D_2O, r.t.) of **1a•CAL**.

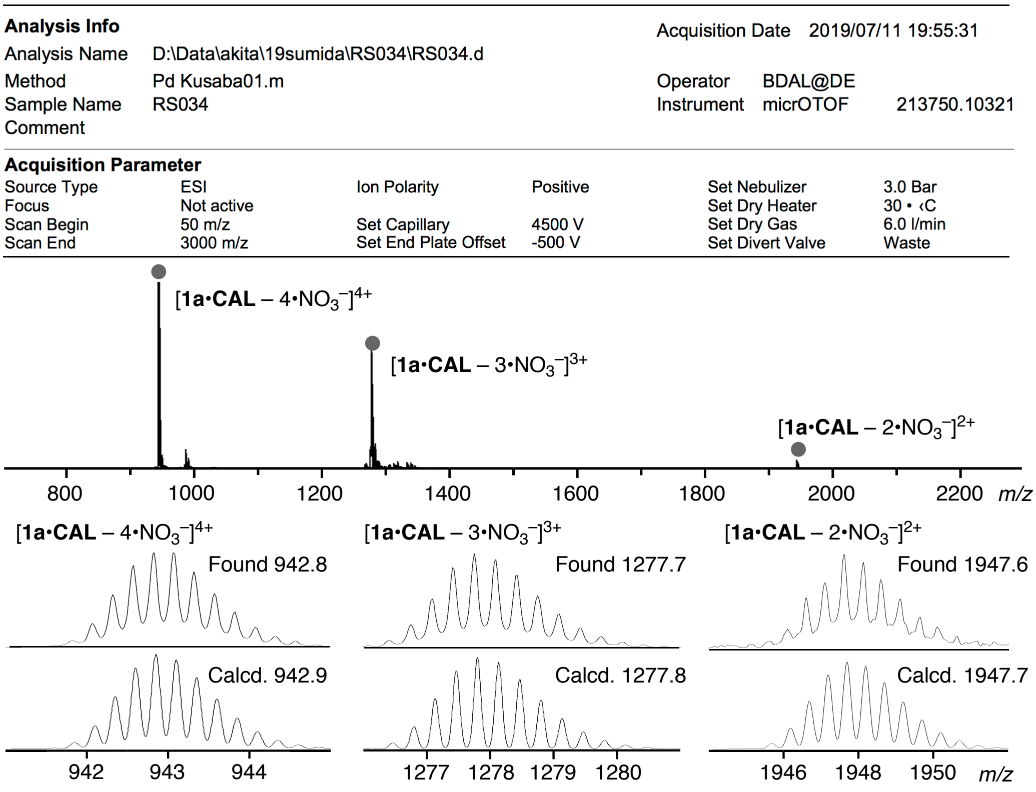

Figure S5a. ESI-TOF MS spectrum (H_2O) of **1a•CAL**.

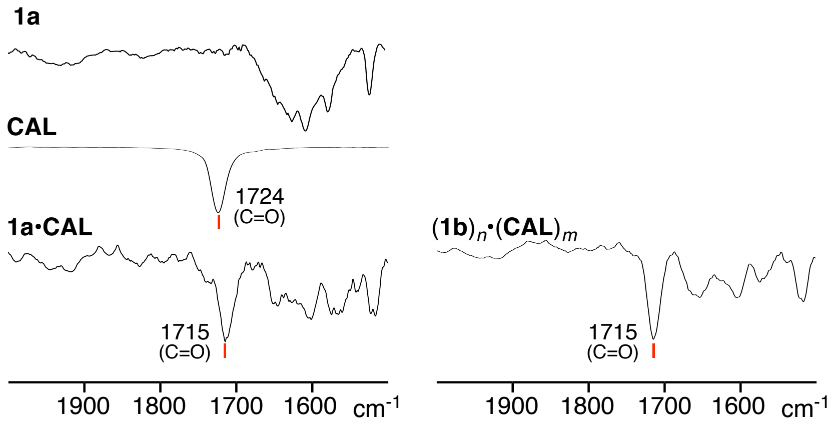

Figure S5b. FT-IR (ATR, r.t.) spectra of **1a•CAL** and $(\mathbf{1b})_n$•$(\mathbf{CAL})_m$.

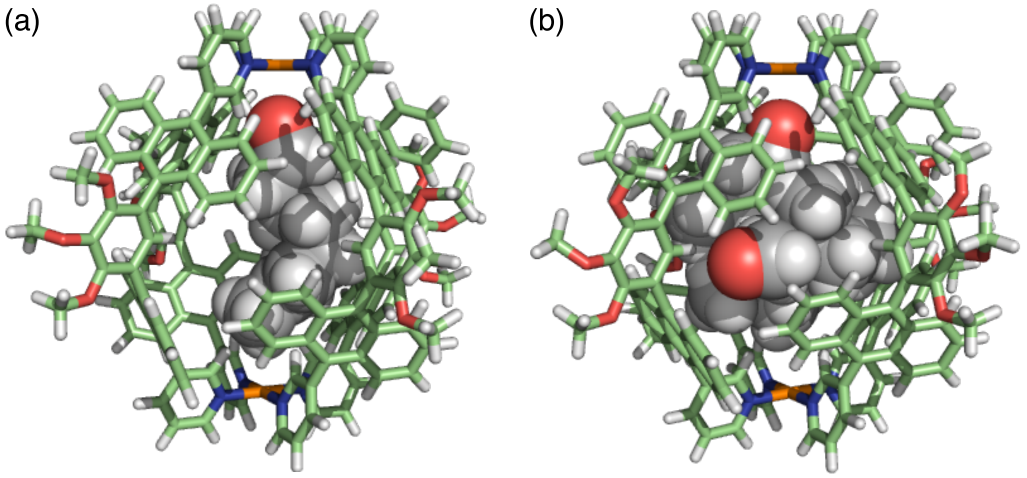

Figure S6. Optimized structures (PM6 calculations, R = $-OCH_3$) of (a) **1a•CAL** and (b) **1a•(CAL)$_2$**.

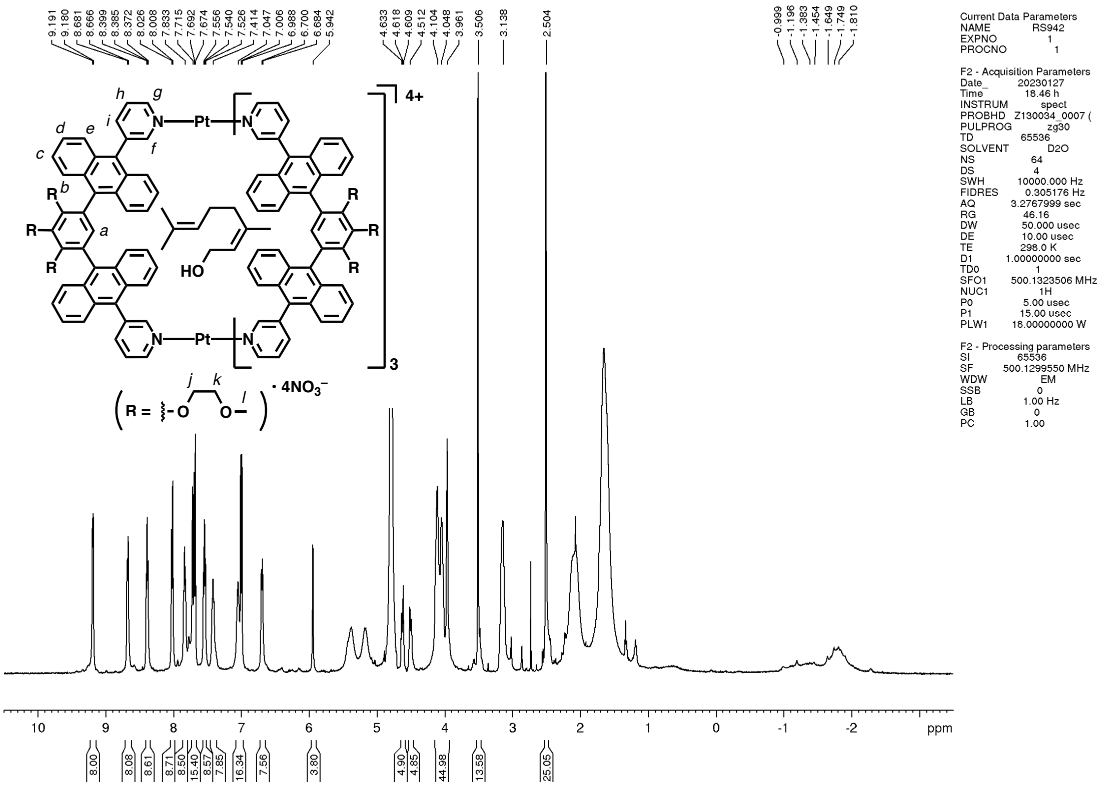

Figure S7. ^{1}H NMR spectrum (500 MHz, D_2O, r.t.) of **1a•GOL**.

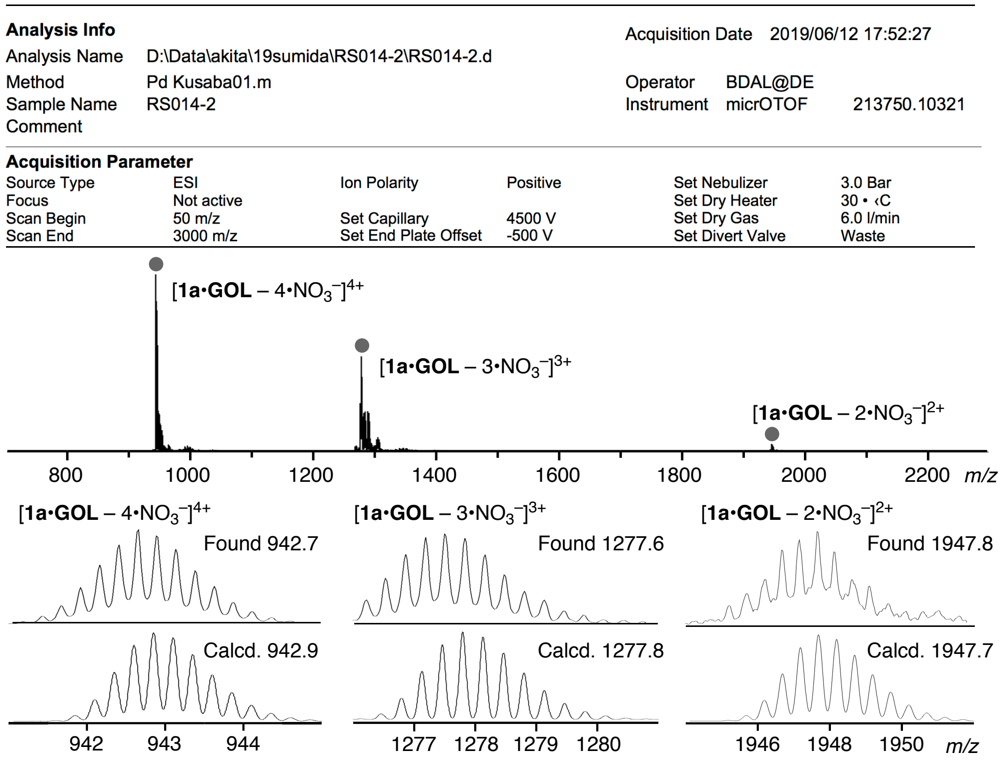

Figure S8. ESI-TOF MS spectrum (H_2O) of **1a•GOL**.

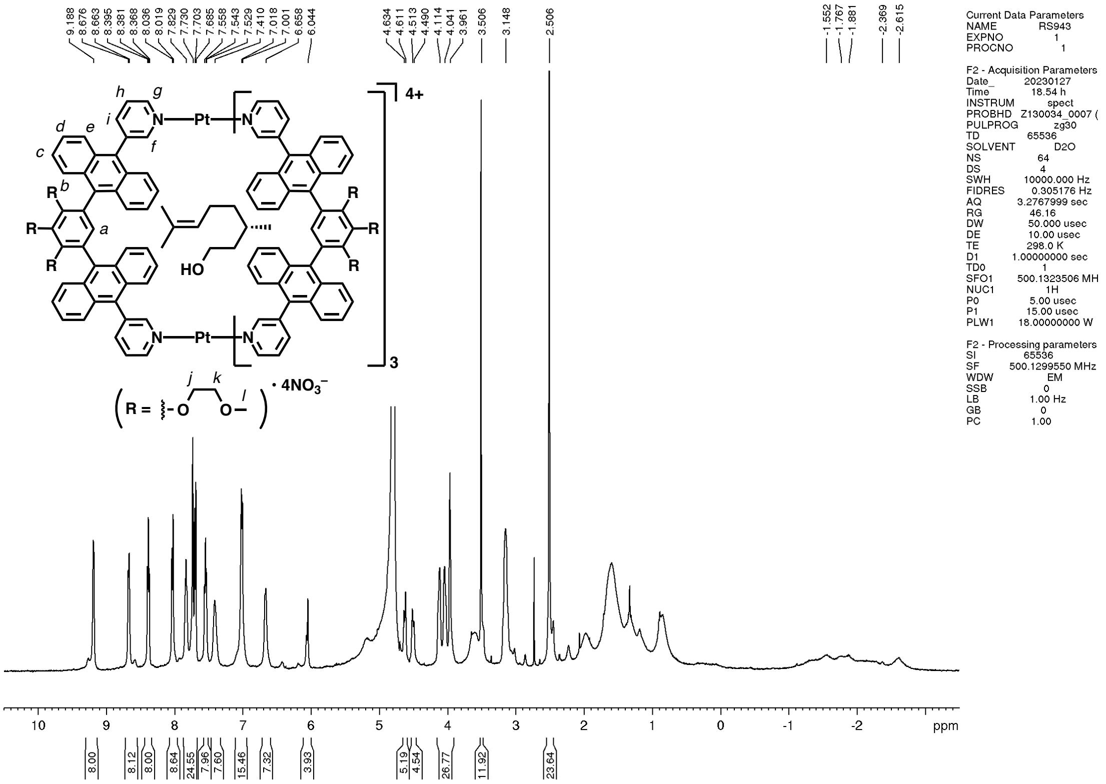

Figure S9. ^{1}H NMR spectrum (500 MHz, D_2O, r.t.) of **1a•COL**.

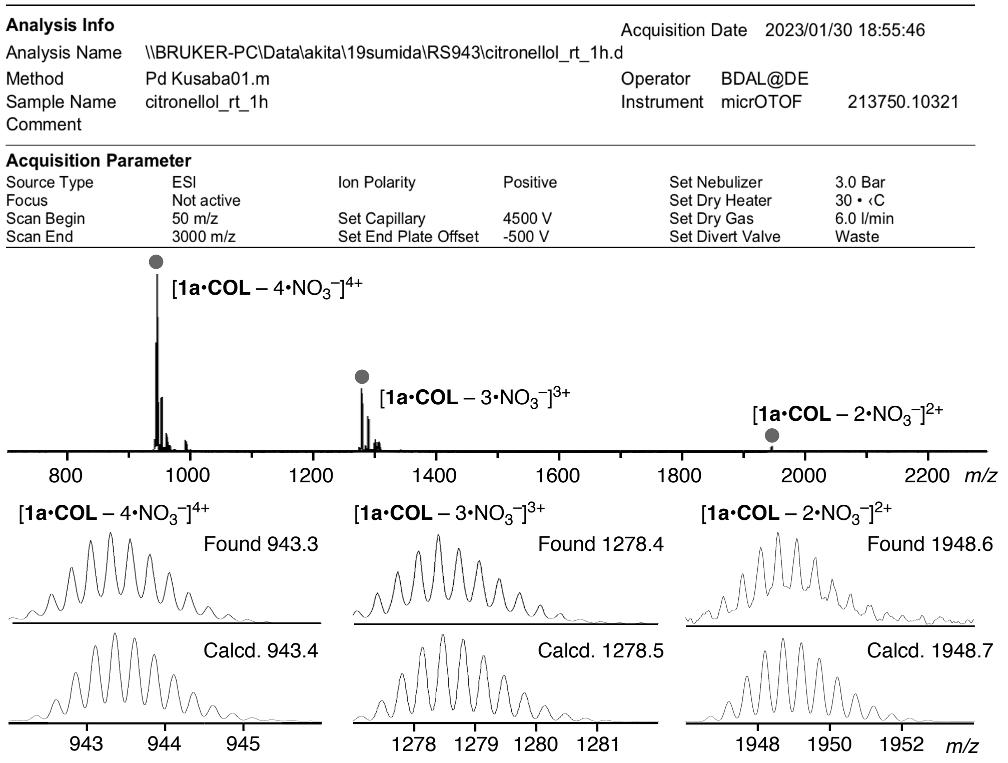

Figure S10. ESI-TOF MS spectrum (H_2O) of **1a•COL**.

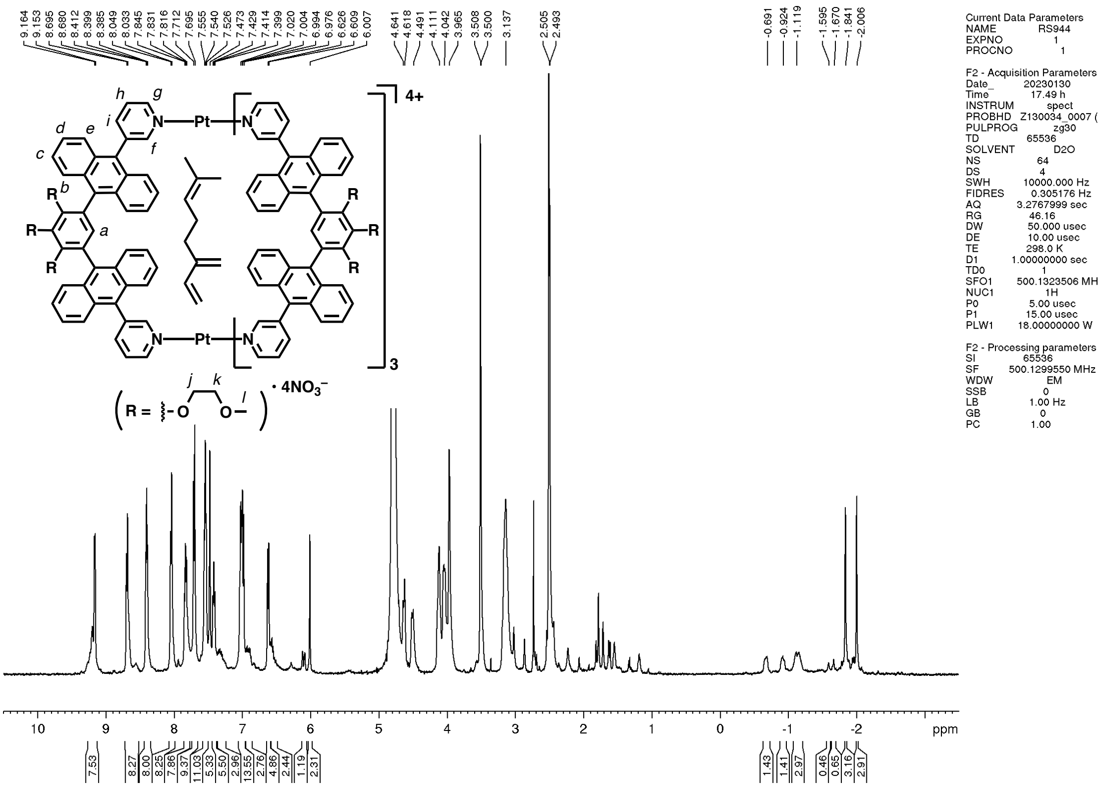

Figure S11. ^{1}H NMR spectrum (500 MHz, D_2O, r.t.) of **1a•MRC**.

**Analysis Info**		Acquisition Date	2023/01/30 19:02:38
Analysis Name	\\BRUKER-PC\Data\akita\19sumida\RS944\myrcene_rt_1h.d		
Method	Pd Kusaba01.m	Operator	BDAL@DE
Sample Name	myrcene_rt_1h	Instrument	micrOTOF 213750.10321
Comment			

Acquisition Parameter

Source Type	ESI	Ion Polarity	Positive	Set Nebulizer	3.0 Bar
Focus	Not active			Set Dry Heater	30 • ‹C
Scan Begin	50 m/z	Set Capillary	4500 V	Set Dry Gas	6.0 l/min
Scan End	3000 m/z	Set End Plate Offset	-500 V	Set Divert Valve	Waste

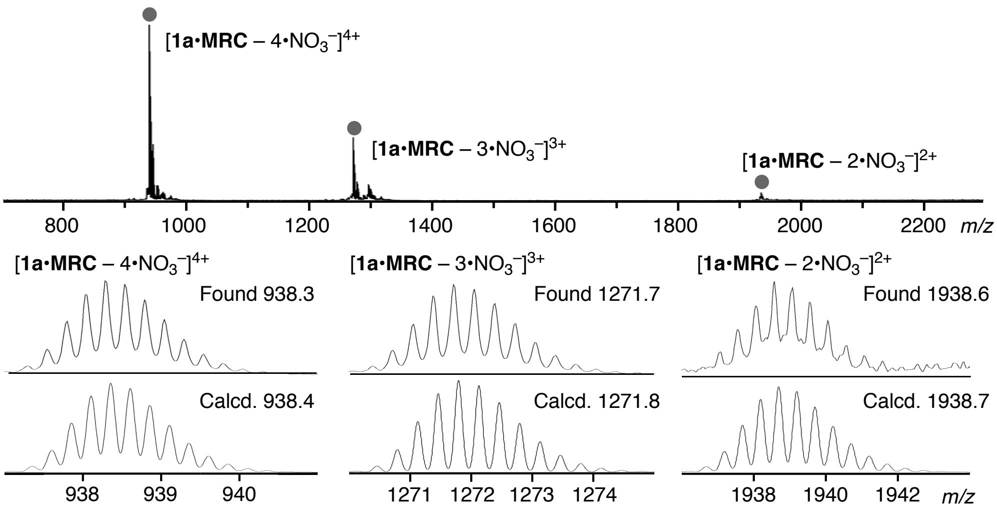

Figure S12. ESI-TOF MS spectrum (H_2O) of **1a•MRC**.

Competitive binding of CAL, GOL, COL, and MRC by 1a RS918

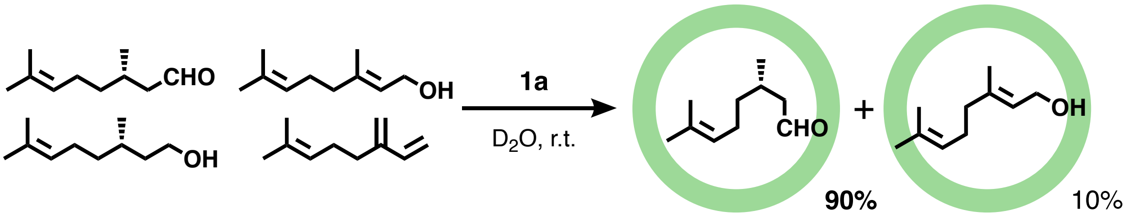

Capsule **1a** (1.0 mg, 0.26 μmol), **CAL** (0.2 mg, 1.0 μmol), **GOL** (0.2 mg, 1.0 μmol), **COL** (0.2 mg, 1.0 μmol), and **MRC** (0.1 mg, 1.0 μmol) were added to a 2 mL test tube containing D_2O (0.5 mL). The mixture was stirred at r.t. for 1 h. The formation of pale-yellow complexes **1a•CAL** and **1a•GOL** in a 90:10 ratio was confirmed by NMR and ESI-TOF MS analyses.

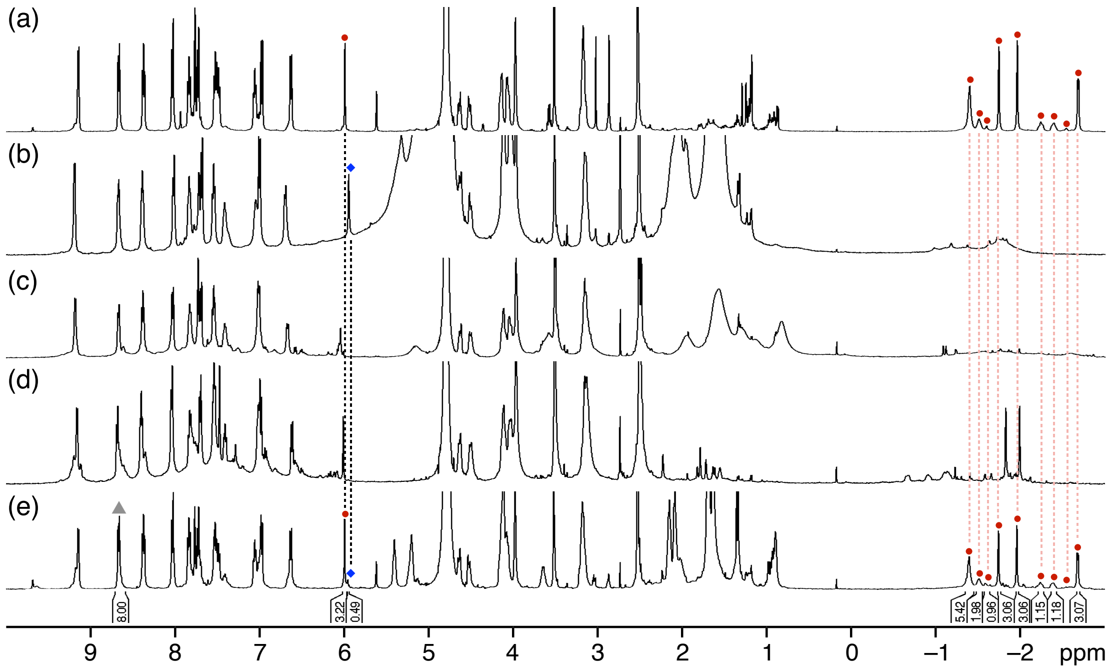

Figure S13a. ^{1}H NMR spectra (500 MHz, D_2O, r.t.) of (a) **1a•CAL**, (b) **1a•GOL**, (c) **1a•COL**, (d) **1a•MRC**, and (e) products after mixing **CAL**, **GOL**, **COL**, and **MRC** with **1a** at r.t. for 1 h (grey triangle: host reference signal (H_i)).

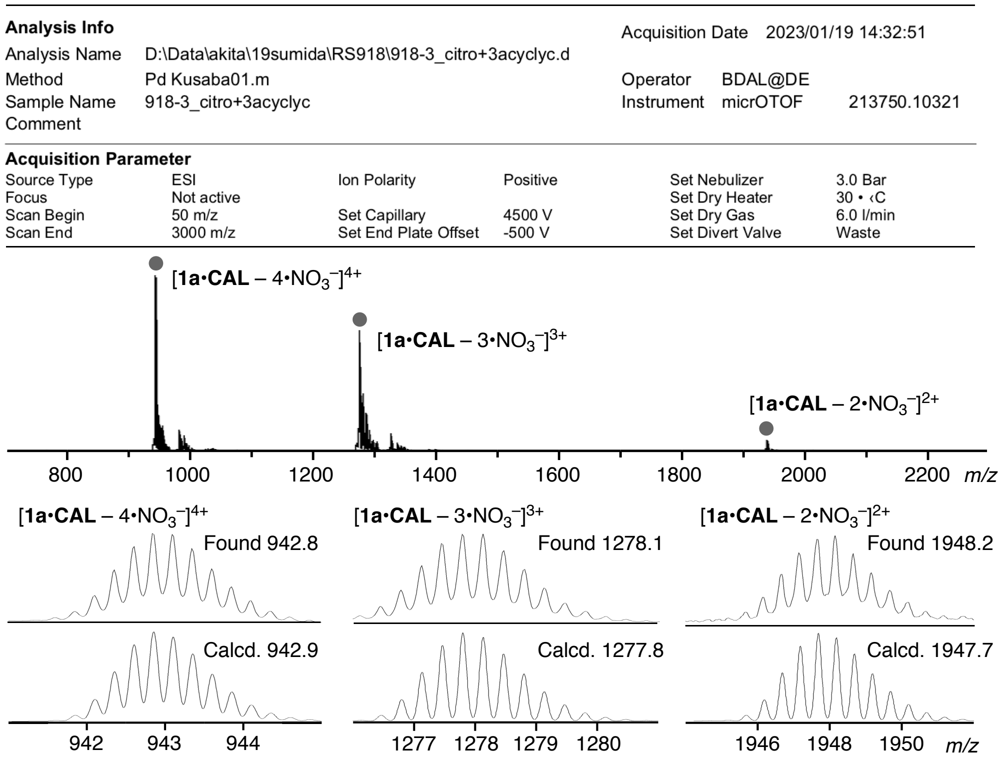

Figure S13b. ESI-TOF MS spectrum (H_2O) of products after mixing **CAL**, **GOL**, **COL**, and **MRC** with **1a** at r.t. for 1 h.

Competitive binding of CAL, CMP, BNL, and MTL by 1a RS919

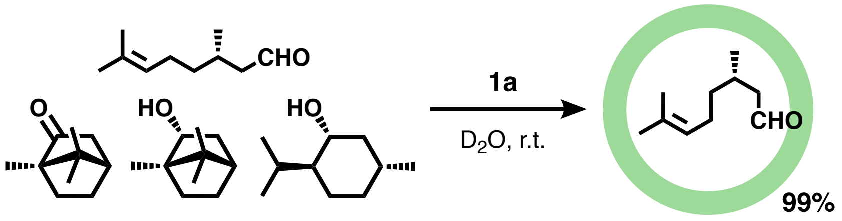

Capsule **1a** (1.0 mg, 0.26 μmol), **CAL** (0.2 mg, 1.0 μmol), **CMP** (0.2 mg, 1.0 μmol), **BNL** (0.2 mg, 1.0 μmol), and **MTL** (0.2 mg, 1.0 μmol) were added to a 2 mL test tube containing D_2O (0.5 mL). The mixture was stirred at r.t. for 1 h. The formation of pale-yellow complexes **1a•CAL** in 99% NMR yield was confirmed by NMR and ESI-TOF MS analyses.

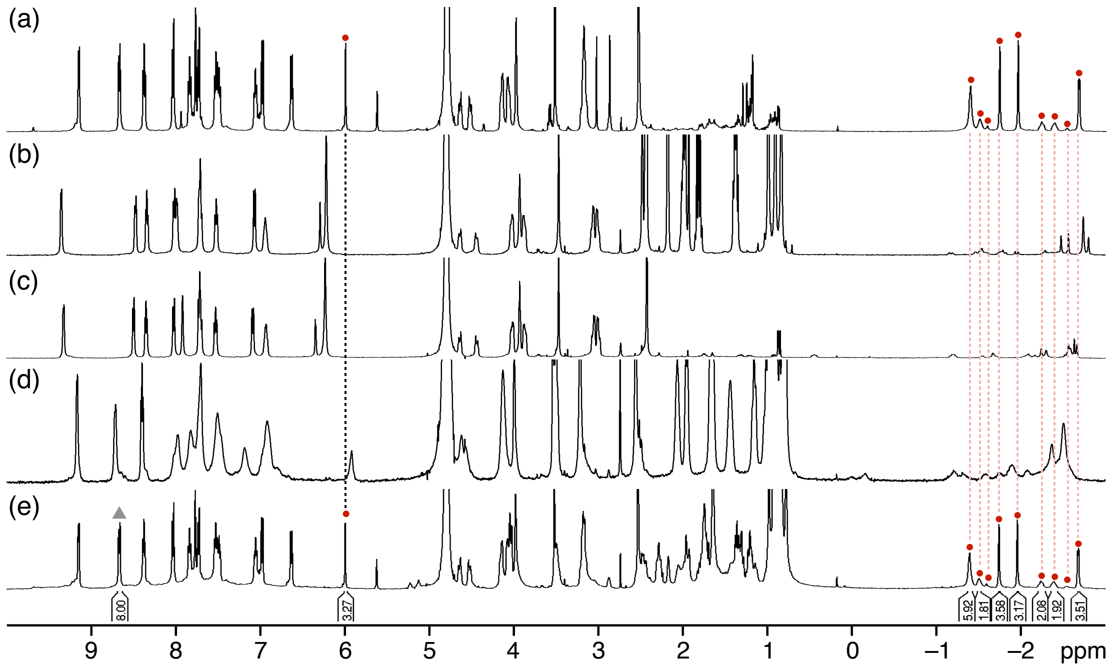

Figure S14a. ^{1}H NMR spectra (500 MHz, D_2O, r.t.) of (a) **1a•CAL**, (b) **1a•CMP**, (c) **1a•BNL**, (d) **1a•MTL**, and (e) products after mixing **CAL**, **CMP**, **BNL**, and **MTL** with **1a** at r.t. for 1 h (grey triangle: host reference signal (H_i)).

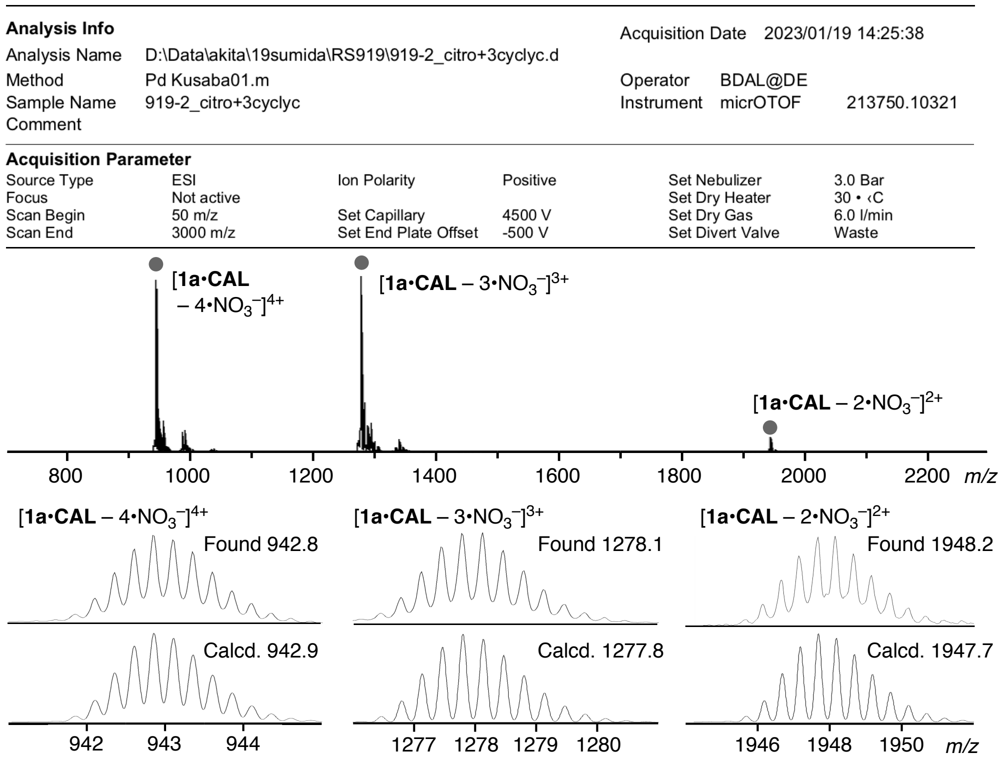

Figure S14b. ESI-TOF MS spectrum (H_2O) of products after mixing **CAL**, **GOL**, **COL**, and **MRC** with **1a** at r.t. for 1 h.

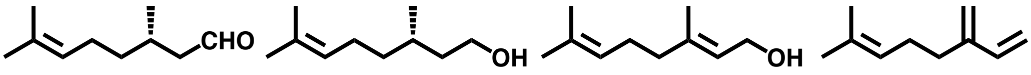

	CAL	COL	GOL	MRC
water solubility	0.46	2.0	0.65	0.03
vapor pressure	3.3	0.26	0.39	2.8
boiling point	205	225	230	167

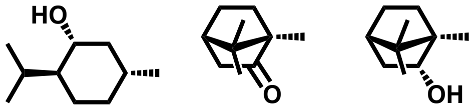

	MTL	CMP	BNL
water solubility	4.0	10.3	4.8
vapor pressure	0.85	8.6	0.53
boiling point	212	209	212

Figure S15. Water solubility (mM), vapor pressure ($\times 10^{-4}$ atm), and boiling point (ºC) of monoterpenes (25 ºC).[S2-7]

Vapor binding of CAL by solid $(\mathbf{1b})_n$ RS516, 635, 718, 908, 920

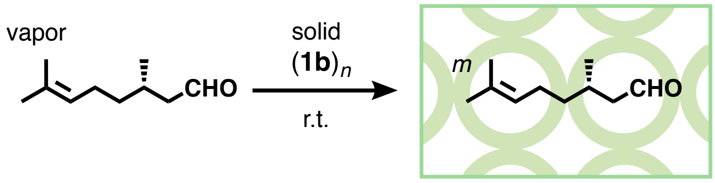

A small open vessel including solid $(\mathbf{1b})_n$ (0.6 mg, 0.2 μmol) was put in a closed glass vessel (50 mL) including **CAL** (2.9 mg, 19 μmol) without direct host-guest contact. After standing 1 h at r.t., the small vessel was taken out from the large vessel and then placed for 1 h under vacuum (480 Pa) at r.t. Resultant pale-yellow amorphous solid $(\mathbf{1b})_n$•$(\mathbf{CAL})_m$ was dissolved in CD_3CN (0.4 mL) to reveal the adsorbed guests and host-guest ratio (n : m = 1.0 : 2.4) by ^{1}H NMR analysis. In the same way, pale-yellow amorphous solid $(\mathbf{1b})_n$•$(\mathbf{GOL})_m$ was obtained from solid $(\mathbf{1b})_n$ (0.6 mg, 0.2 μmol) and **GOL** (2.1 mg, 20 μmol) and its host-guest ratio (n : m = 1.0 : 0.23) was estimated by ^{1}H NMR analysis. Pale-yellow amorphous host-guest solid $(\mathbf{1b})_n$•$(\mathbf{COL})_m$ (n : m = 1.0 : 1.0) and $(\mathbf{1b})_n$•$(\mathbf{MRC})_m$ (n : m = 1.0 : 0.6) were also obtained from solid $(\mathbf{1b})_n$ (0.6 mg, 0.2 μmol)/**COL** (3.1 mg, 19 μmol) and solid $(\mathbf{1b})_n$ (0.6 mg, 0.2 μmol)/**MRC** (2.7 mg, 19 μmol) under the same conditions. Solid $(\mathbf{1b})_n$ bound vaporized (–)-perillaldehyde (**PRA**) only in 32% based on **1b** under the same conditions, using $(\mathbf{1b})_n$ (0.6 mg, 0.2 μmol) and **PRA** (2.6 mg, 17 μmol).

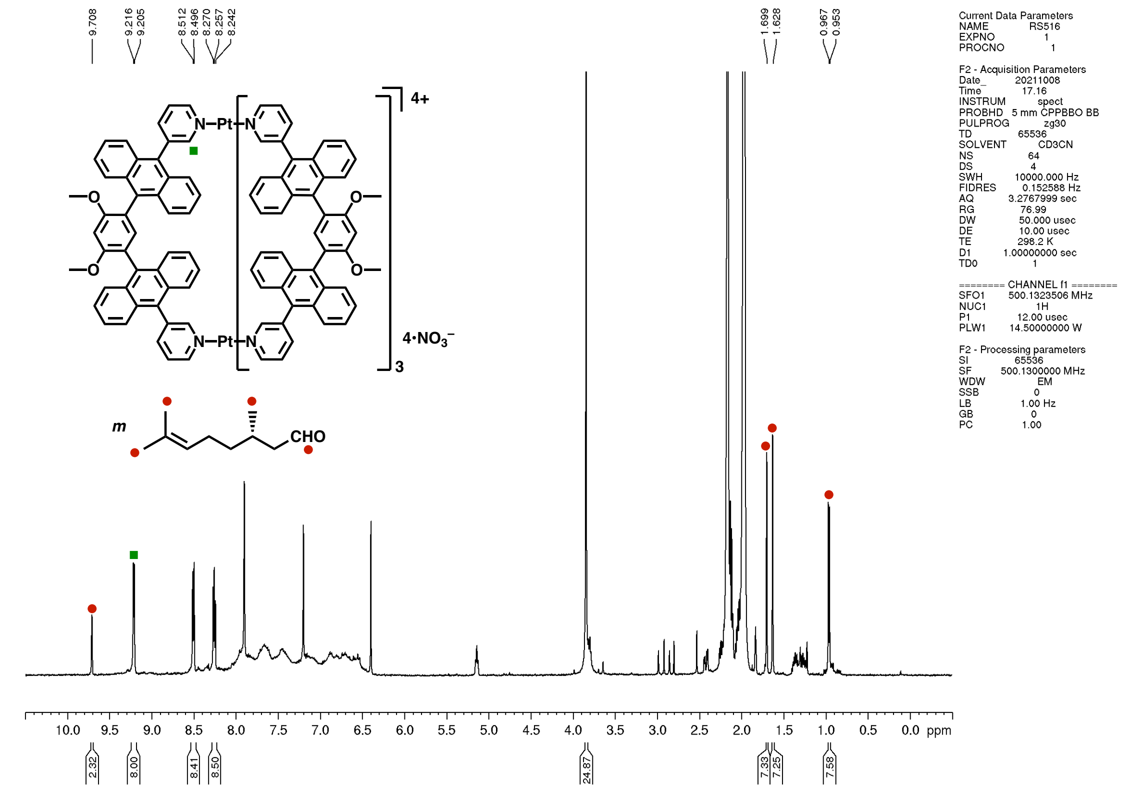

Figure S16. ^{1}H NMR spectrum (500 MHz, CD_3CN, r.t.) of products after the vapor binding of **CAL** by solid (**1b**)$_n$ at r.t. for 1 h.

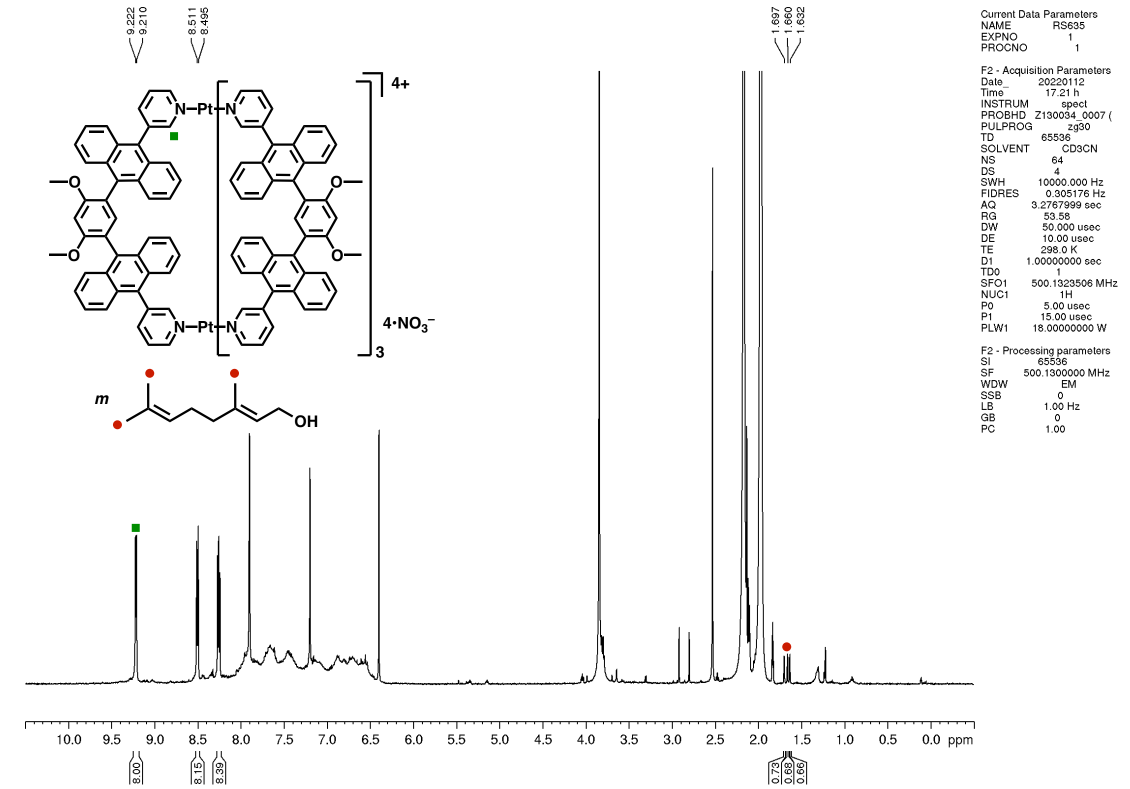

Figure S17. ^{1}H NMR spectrum (500 MHz, CD_3CN, r.t.) of products after the vapor binding of **GOL** by solid (**1b**)$_n$ at r.t. for 1 h.

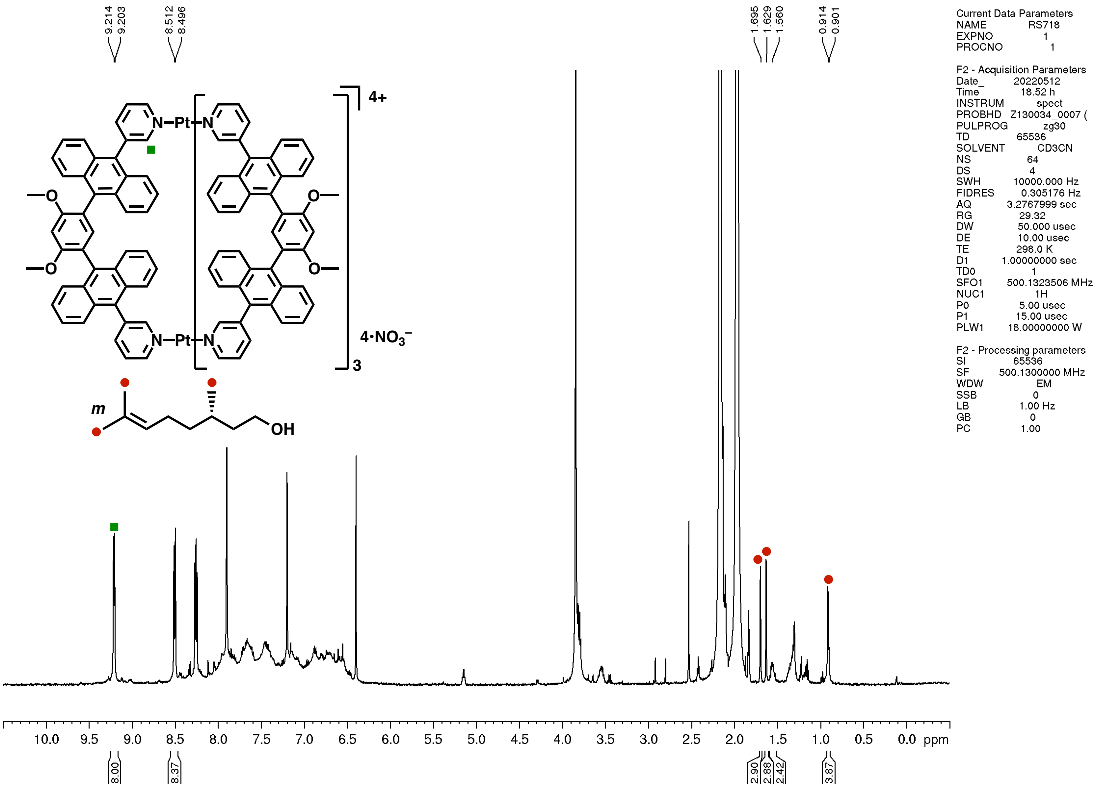

Figure S18. ^{1}H NMR spectrum (500 MHz, CD$_3$CN, r.t.) of products after the vapor binding of **COL** by solid (**1b**)$_n$ at r.t. for 1 h.

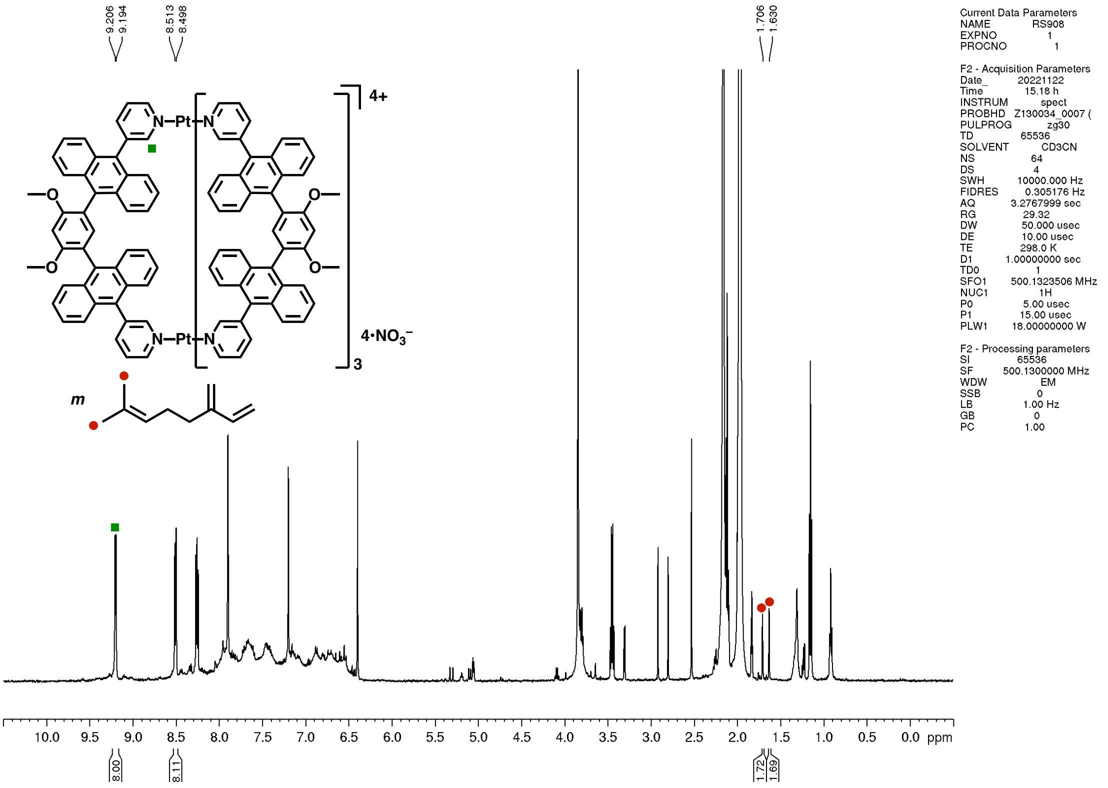

Figure S19. ^{1}H NMR spectrum (500 MHz, CD$_3$CN, r.t.) of products after the vapor binding of **MRC** by solid (**1b**)$_n$ at r.t. for 1 h.

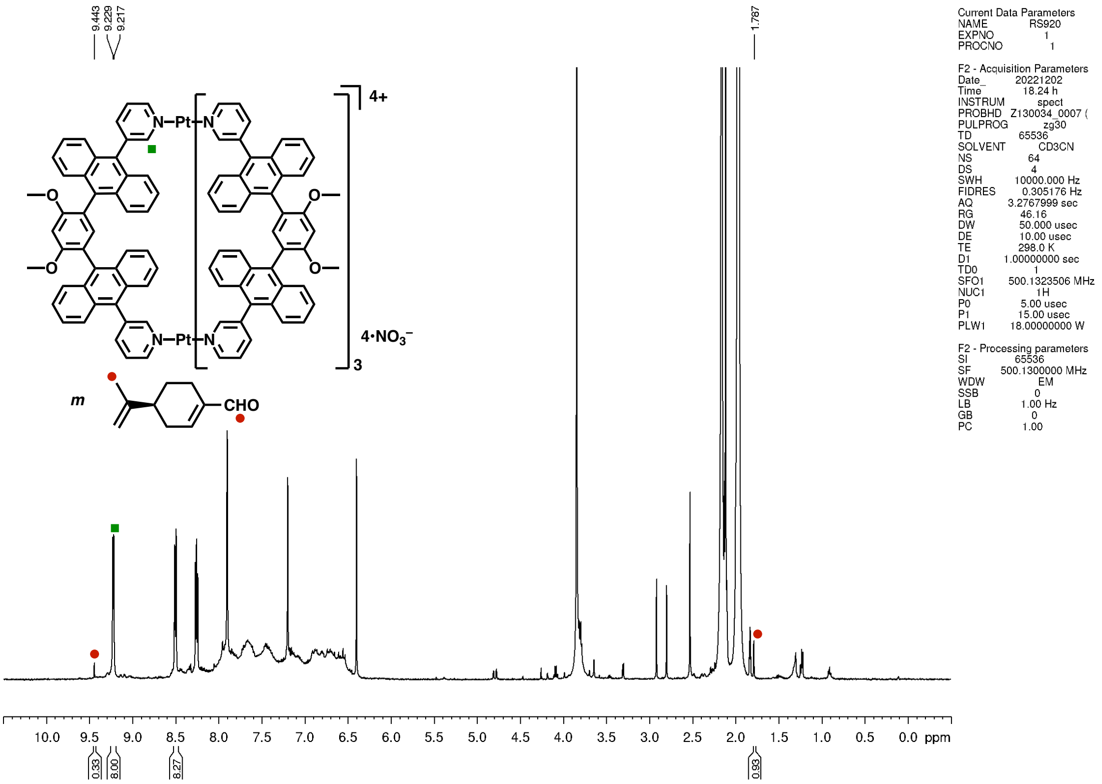

Figure S20. ^{1}H NMR spectrum (500 MHz, CD_3CN, r.t.) of products after the vapor binding of **PRA** by solid (**1b**)$_n$ at r.t. for 1 h.

Competitive vapor binding of CAL and GOL by solid (1b)$_n$ RS862, 880, 902

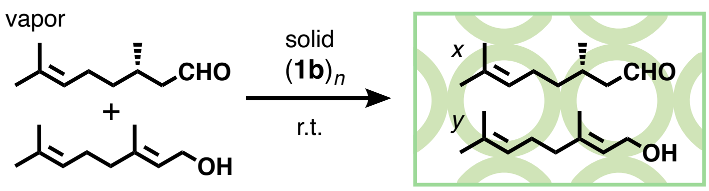

A small open vessel including solid (**1b**)$_n$ (0.6 mg, 0.2 μmol) was put in a closed glass vessel (50 mL) including **CAL** (1.2 mg, 7.7 μmol) and **GOL** (1.2 mg, 7.7 μmol), without direct host-guest contact. After standing 2 h at r.t., the small vessel was taken out from the large vessel and then placed for 1 h under vacuum (480 Pa) at r.t. Resultant pale-yellow amorphous solid (**1b**)$_n$•(**CAL**)$_x$•(**GOL**)$_y$ was dissolved in CD_3CN (0.4 mL) to reveal the adsorbed guests and host-guest ratio ($n : x : y$ = 1.0 : 2.1 : 0.5) by ^{1}H NMR analysis. In the same way, pale-yellow amorphous solid (**1b**)$_n$•(**CAL**)$_x$•(**COL**)$_y$ was obtained from solid (**1b**)$_n$ (0.6 mg, 0.2 μmol), **CAL** (1.2 mg, 7.7 μmol), and **COL** (1.2 mg, 7.9 μmol) and its host-guest ratio ($n : x : y$ = 1.0 : 1.4 : 1.0) was estimated by ^{1}H NMR analysis. Pale-yellow amorphous host-guest solid (**1b**)$_n$•(**CAL**)$_x$•(**MRC**)$_y$ ($n : x : y$ = 1.0 : 2.6 : 0.03) was obtained from solid (**1b**)$_n$ (0.6 mg, 0.2 μmol), **CAL** (1.1 mg, 7.3 μmol),

and **MRC** (1.0 mg, 7.3 μmol) under the same conditions.

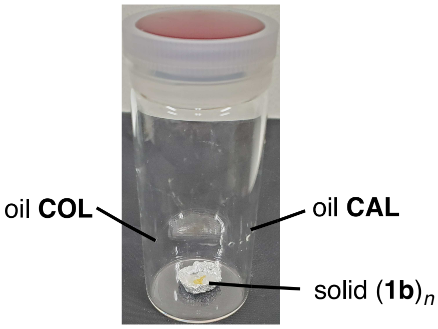

Figure S21a. Experimental setup for the binding of volatilized linear monoterpenes by solid (**1b**)$_n$.

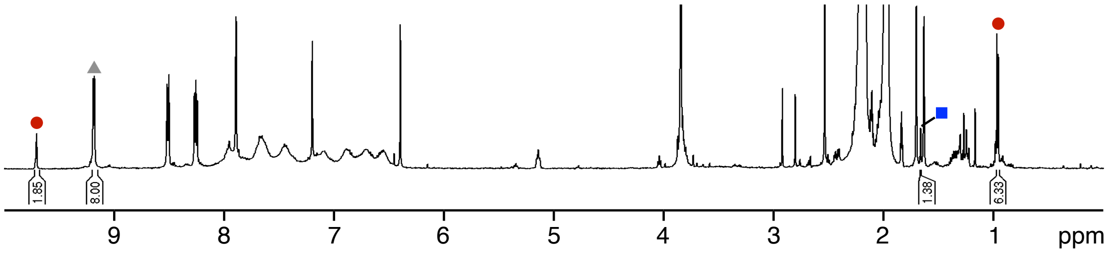

Figure S21b. ^{1}H NMR spectrum (500 MHz, CD_3CN, r.t.) of products after the competitive vapor binding of **CAL** (red circle) and **GOL** (blue square) by solid (**1b**)$_n$ (gray triangle) at r.t. for 2 h.

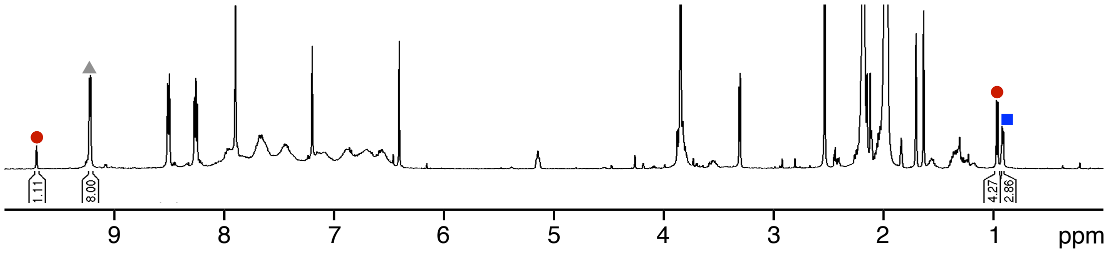

Figure S22a. ^{1}H NMR spectrum (500 MHz, CD_3CN, r.t.) of products after the competitive vapor binding of **CAL** (red circle) and **COL** (blue square) by solid (**1b**)$_n$ (gray triangle) at r.t. for 2 h.

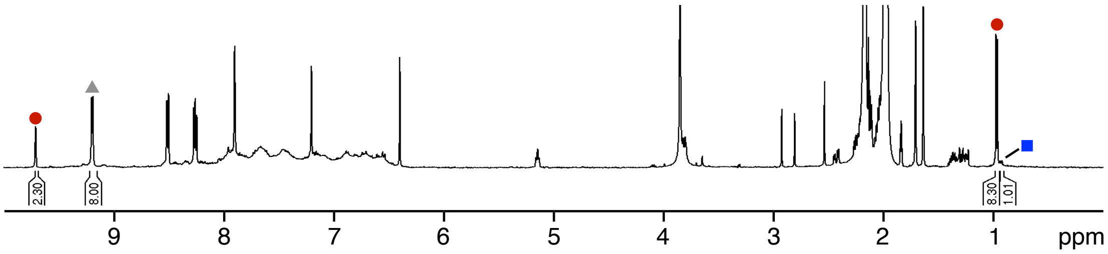

Figure S22b. ^{1}H NMR spectrum (500 MHz, CD_3CN, r.t.) of products after the competitive vapor binding of **CAL** (red circle) and **MRC** (blue square) by solid (**1b**)$_n$ (gray triangle) at r.t. for 2 h.

Competitive vapor binding of CAL and CMP by solid (1b)$_n$ RS864, 869, 903

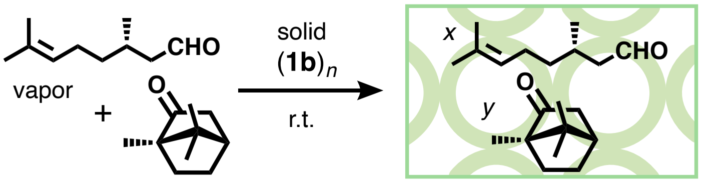

A small open vessel including solid (**1b**)$_n$ (0.6 mg, 0.2 μmol) was put in a closed glass vessel (50 mL) including **CAL** (1.1 mg, 6.9 μmol) and **CMP** (1.0 mg, 6.8 μmol), without direct host-guest contact. After standing 2 h at r.t., the small vessel was taken out from the large vessel and then placed for 1 h under vacuum (480 Pa) at r.t. Resultant pale-yellow amorphous solid (**1b**)$_n$•(**CAL**)$_x$•(**CMP**)$_y$ was dissolved in CD_3CN (0.4 mL) to reveal the adsorbed guests and host-guest ratio (n : x : y = 1.0 : 2.2 : 0.5) by ^{1}H NMR analysis. In the same way, pale-yellow amorphous solid (**1b**)$_n$•(**CAL**)$_x$•(**BNL**)$_y$ was obtained from solid (**1b**)$_n$ (0.6 mg, 0.2 μmol), **CAL** (1.1 mg, 7.1 μmol), and **BNL** (1.0 mg, 6.6 μmol) and its host-guest ratio (n : x : y = 1.0 : 2.1 : 0.4) was estimated by ^{1}H NMR analysis. Pale-yellow amorphous host-guest solid (**1b**)$_n$•(**CAL**)$_x$•(**MTL**)$_y$ (n : x : y = 1.0 : 2.5 : 0.7) was obtained from solid (**1b**)$_n$ (0.6 mg, 0.2 μmol), **CAL** (1.1 mg, 7.3 μmol), and **MTL** (1.1 mg, 7.0 μmol) under the same conditions.

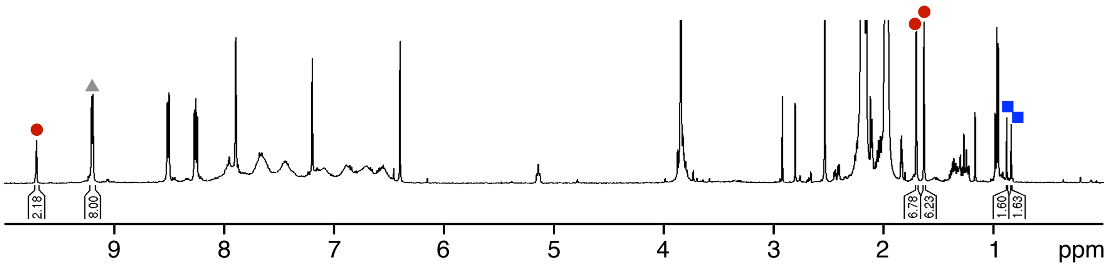

Figure S23. ^{1}H NMR spectrum (500 MHz, CD_3CN, r.t.) of products after the competitive vapor binding of **CAL** (red circle) and **CMP** (blue square) by solid (**1b**)$_n$ (gray triangle) at r.t. for 2 h.

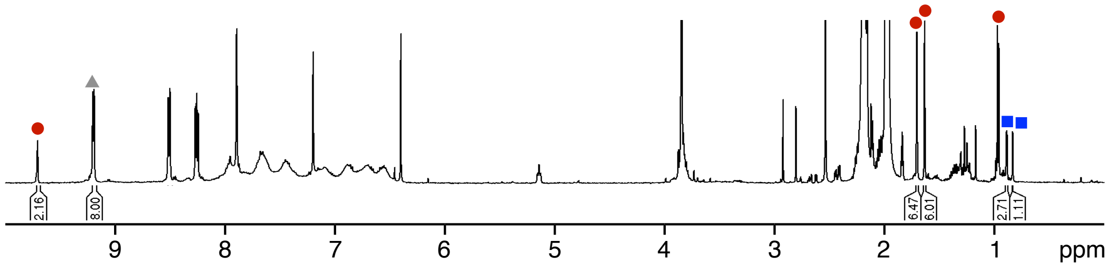

Figure S24. ^{1}H NMR spectrum (500 MHz, CD_3CN, r.t.) of products after the competitive vapor binding of **CAL** (red circle) and **BNL** (blue square) by solid (**1b**)$_n$ (gray triangle) at r.t. for 2 h.

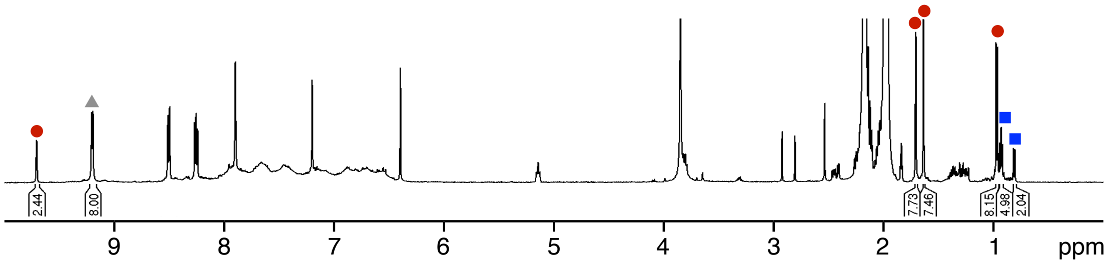

Figure S25. ^{1}H NMR spectrum (500 MHz, CD_3CN, r.t.) of products after the competitive vapor binding of **CAL** (red circle) and **MTL** (blue square) by solid $(\mathbf{1b})_n$ (gray triangle) at r.t. for 2 h.

Formation of 1a•PMD from 1a and a reaction mixture of CAL RS961, 1010, 1011, 1044, 1064

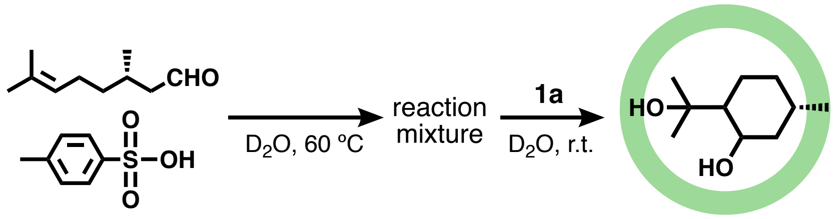

CAL (0.40 mg, 2.59 μmol) and *p*-toluenesulfonic acid (**TS**; 0.5 equiv. for **CAL**) were added to a 2 mL test tube containing D_2O (1.5 mL). The mixture was stirred at 60 °C (oil bath) for 1 h and then the product mixture was extracted with $CDCl_3$. The products were confirmed by GC analysis (Figure S26a) and the yields of the main products (*ax*-**PMD**: 52%, *eq*-**PMD**: 34%) were estimated by ^{1}H NMR analysis before the following workup (Figure S26b). The organic layer was dried over $MgSO_4$ and filtrated. The $CHCl_3$ solution (450 mL, 2.33 μmol based on **CAL**) was added to a 2 mL test tube. After removal of the solvent, a D_2O solution (0.2 mL) of **1a** (1.0 mg, 0.26 μmol, 0.1 equiv. for **CAL**) was added to the test tube and the mixture was stirred at r.t. for 10 min. The quantitative formation of **1a**•(*p*-menthane-3,8-diol (**PMD**)) was confirmed by NMR and ESI-TOF MS analyses. The ratio of **1a•***eq***-PMD** and **1a•***ax***-PMD** was estimated to be 2:1 by ^{1}H NMR analysis (Figure S27b).

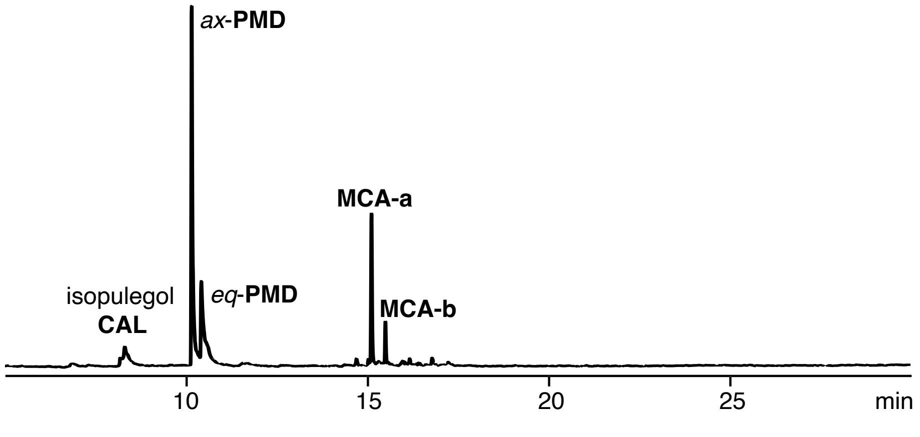

Figure S26a. GC chart of the extracted products from a mixture of **CAL** and **TS** in $CHCl_3$ (without **1a**) at 60 °C for 1 h.

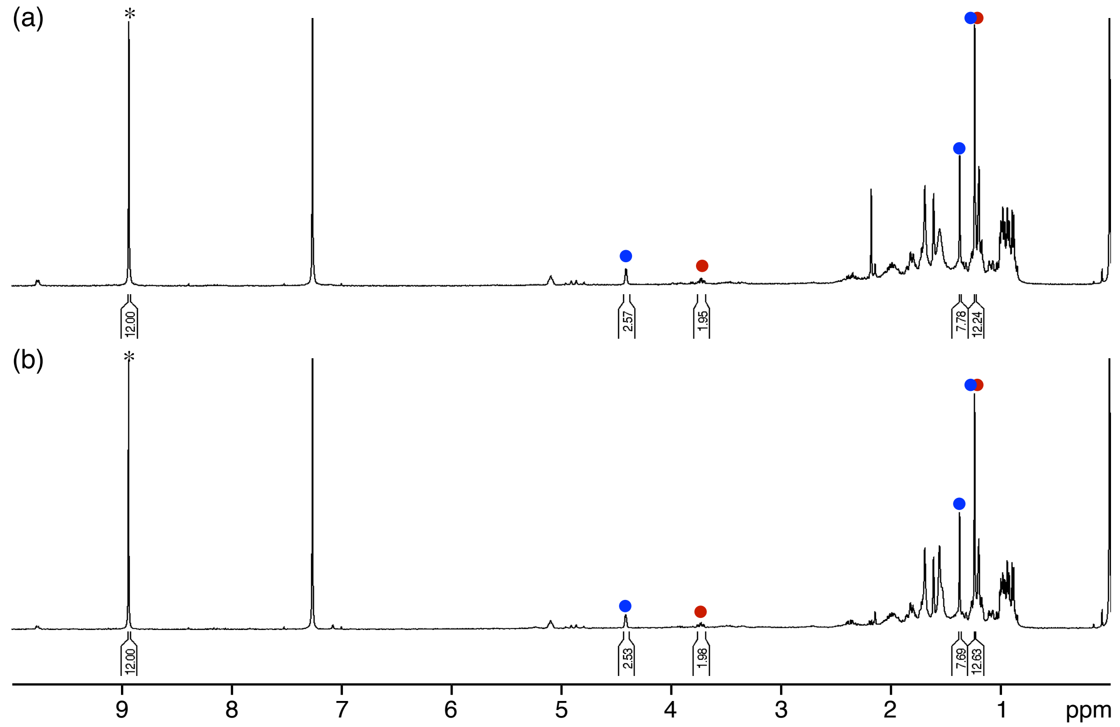

Figure S26b. ^{1}H NMR spectra (400 MHz, r.t., $CDCl_3$) of the extracted products from a reaction mixture of **CAL** and **TS** with $CDCl_3$ (a) before and (b) after workup (red circle: *eq*-**PMD**, blue circle: *ax*-**PMD**, *: coronene as an internal standard).

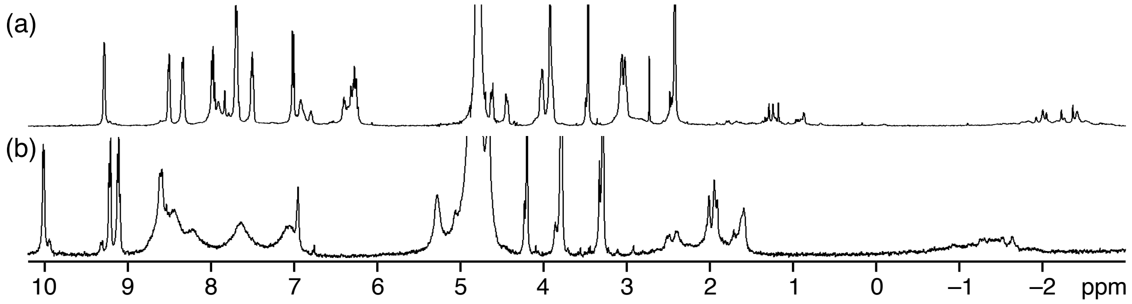

Figure S27a. ^{1}H NMR spectra (500/400 MHz, D_2O) of products after stirring **1a** and a reaction mixture of **CAL** and **TS** at r.t. for 10 min: (a) r.t. and (b) 100 ºC.

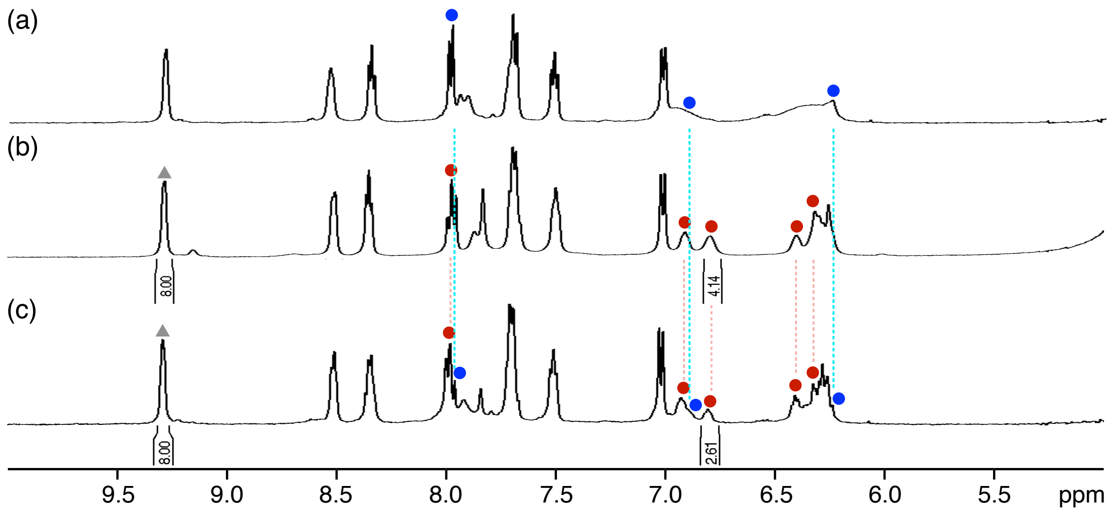

Figure S27b. Expanded ^{1}H NMR spectra (500 MHz, D_2O, r.t.) and characteristic signals of (a) **1a•***ax*-**PMD**, (b) **1a•***eq*-**PMD**, and (c) products after stirring **1a** and a reaction mixture of **CAL** and **TS** at r.t. for 10 min (grey triangle: host reference signal (H_g)).

Analysis Info

Analysis Name	D:\Data\akita\19sumida\RS1011-13\RS1011-13_citro+p-TS_PtOMOE3.d	Acquisition Date	2023/05/12 11:56:12
Method	Pd Kusaba01.m	Operator	BDAL@DE
Sample Name	RS1007-21_GPC1-3	Instrument	micrOTOF 213750.10321
Comment			

Acquisition Parameter

Source Type	ESI	Ion Polarity	Positive	Set Nebulizer	3.0 Bar
Focus	Not active			Set Dry Heater	30 • ‹C
Scan Begin	50 m/z	Set Capillary	4500 V	Set Dry Gas	6.0 l/min
Scan End	3000 m/z	Set End Plate Offset	-500 V	Set Divert Valve	Waste

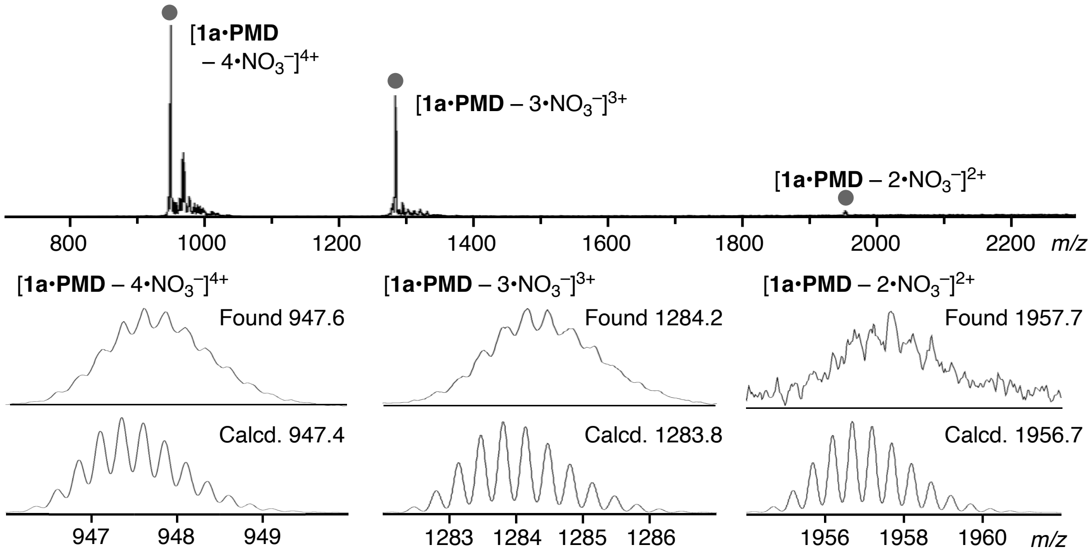

Figure S28. ESI-TOF MS spectrum (H_2O) of products after stirring **1a** and a reaction mixture obtained by mixing of **CAL** and **TS** at r.t. for 10 min.

Synthesis of *eq*-PMD and *ax*-PMD RS998

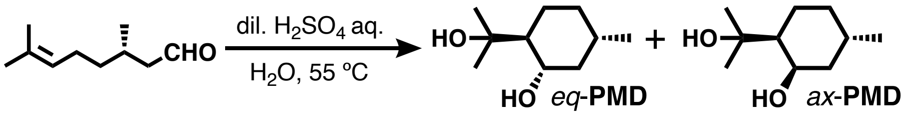

CAL (500 mg, 3.24 mmol) was added to a screw test tube containing 0.25 wt% H_2SO_4 (636 mg, 16.2 µmol).[S8] After the mixture was stirred at 55 °C (oil bath) for 17 h, a 25 wt% NaOH aqueous solution (8.0 mg, 49 µmol) and heptane (1.2 mL) were added to the mixture. The organic layer was washed with H_2O (0.5 mL), separated, dried over $MgSO_4$, filtrated, and then concentrated under reduced pressure to give the crude product as a colorless oil. *eq*-**PMD** (32.6 mg, 0.19 mmol, 6%) and *ax*-**PMD** (92.1 mg, 0.53 mmol, 17%) were isolated as white solids by recycling GPC ($CHCl_3$).[S8]

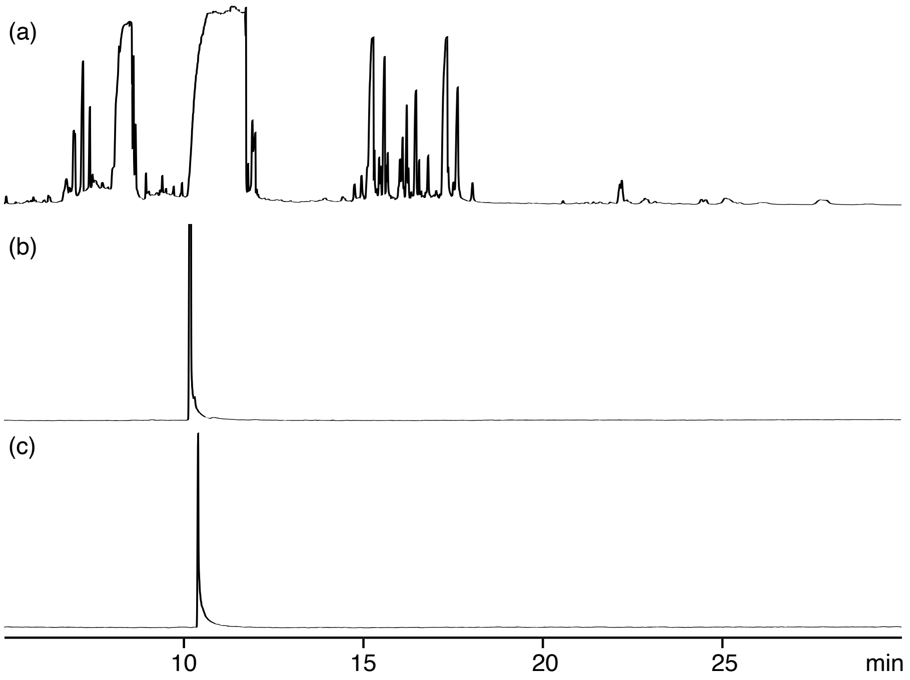

Figure S29. GC data of (a) crude products, (b) *ax*-**PMD**, and (c) *eq*-**PMD**.

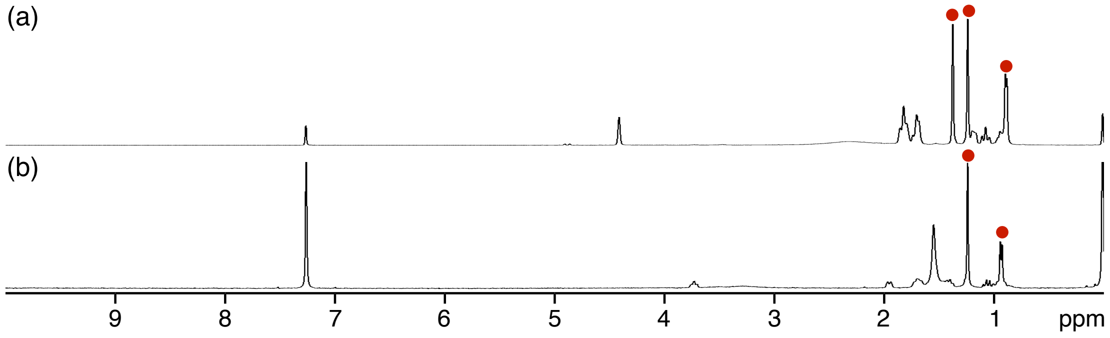

Figure S30. ^{1}H NMR spectra (400 MHz, $CDCl_3$, r.t.) of (a) *ax*-**PMD** and (b) *eq*-**PMD**.

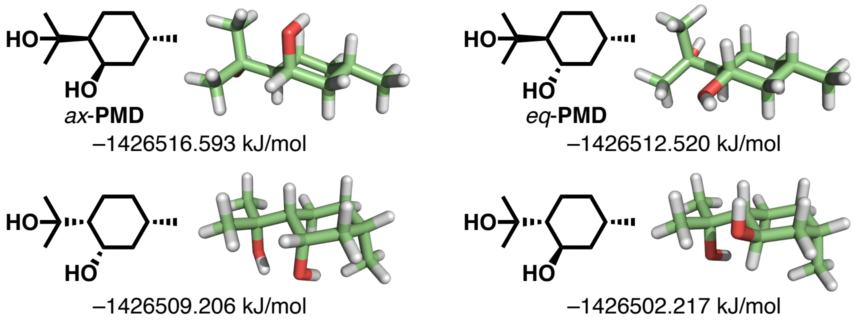

Figure S31. Optimized structures (PM6 calculations) of **PMD** isomers and their energies (DFT calculations).

Formation of 1a•*eq/ax*-PMD from 1a and isolated *eq/ax*-PMD RS1007, 1065

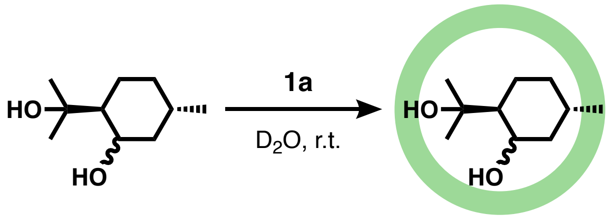

Capsule **1a** (1.0 mg, 0.26 μmol) and *ax*-**PMD** (0.41 mg, 2.4 μmol) were added to a 2 mL test tube containing D_2O (0.5 mL). The mixture was stirred at r.t. for 10 min. The quantitative formation of 1:1 host-guest complex **1a•***ax*-**PMD** was confirmed by ^{1}H NMR and ESI-TOF MS analyses. In the same way, from **1a** (1.0 mg, 0.26 μmol) and *eq*-**PMD** (0.18 mg, 1.0 μmol), the quantitative formation of **1a•***eq*-**PMD** was confirmed by ^{1}H NMR and ESI-TOF MS analyses. Under the same conditions, the treatment of a mixture of **1a** (1.0 mg, 0.26 μmol), *ax*-**PMD** and *eq*-**PMD** (0.13 mg, 0.76 μmol each) gave rise to **1a•***ax*-**PMD** and **1a•***eq*-**PMD** in a 1:9 ratio, as revealed by ^{1}H NMR analysis (100 ºC).

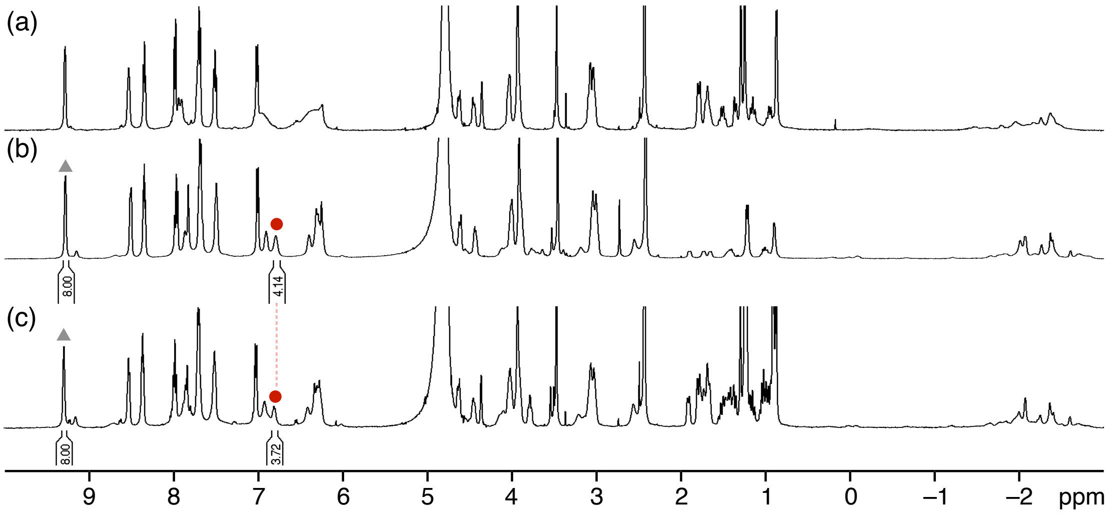

Figure S32. ^{1}H NMR spectra (500 MHz, D_2O, r.t.) of (a) **1a•***ax*-**PMD**, (b) **1a•***eq*-**PMD**, and (c) products after mixing *eq*-**PMD** and *ax*-**PMD** with **1a** at r.t. for 10 min (grey triangle: host reference signal (H_g)).

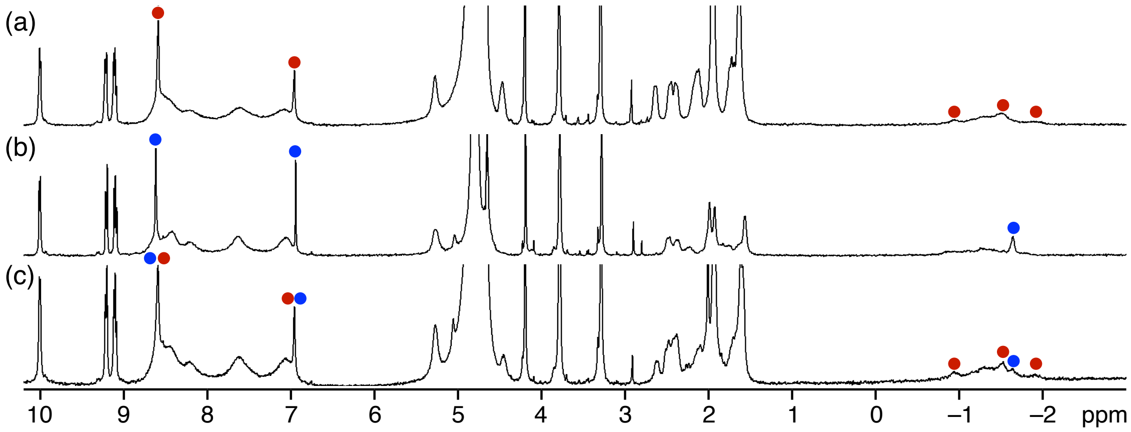

Figure S33. ^{1}H NMR spectra (400 MHz, D_2O, 100 ºC) of (a) **1a•*eq*-PMD**, (b) **1a•*ax*-PMD**, and (c) products after mixing *eq*-**PMD** and *ax*-**PMD** (3.0 equiv. each) with **1a** at r.t. for 10 min.

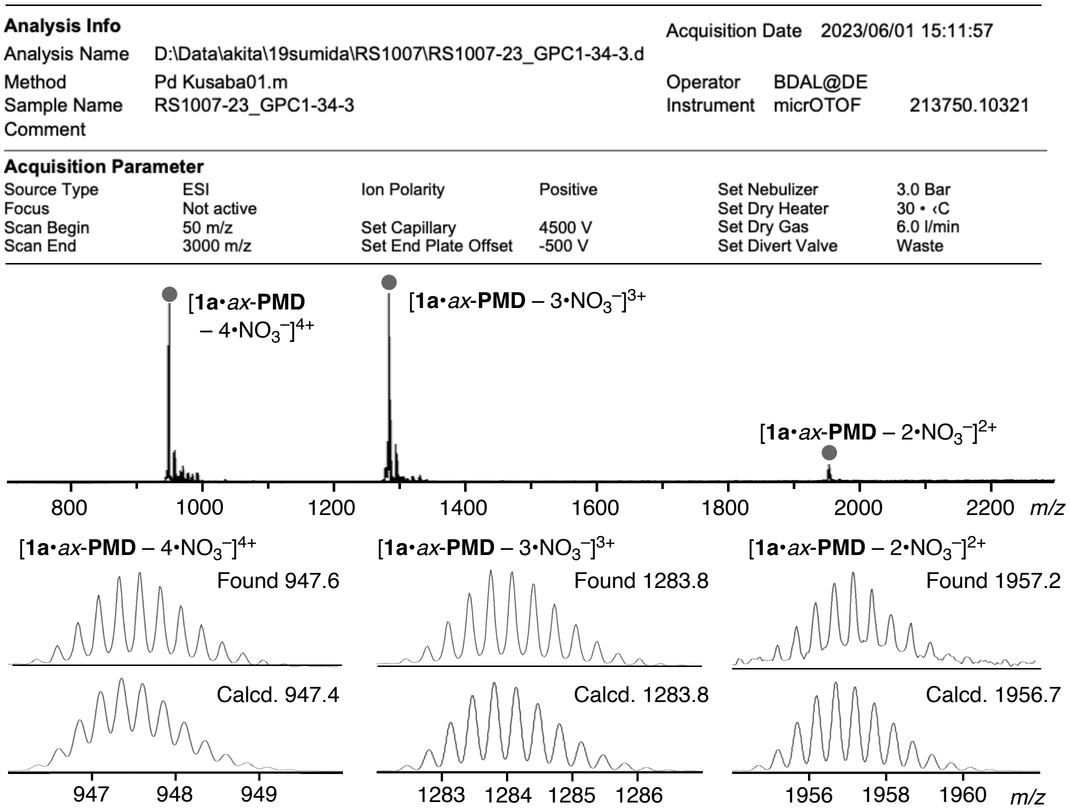

Figure S34. ESI-TOF MS spectrum (H_2O) of **1a•*ax*-PMD**.

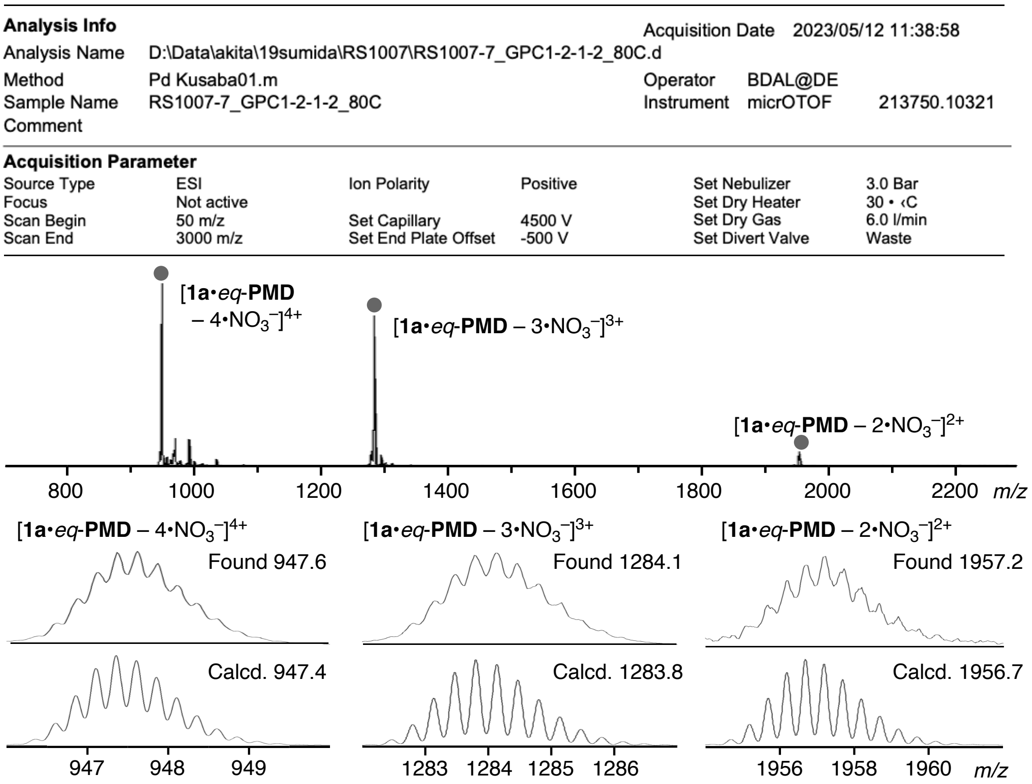

Figure S35. ESI-TOF MS spectrum (H_2O) of **1a•*eq*-PMD**.

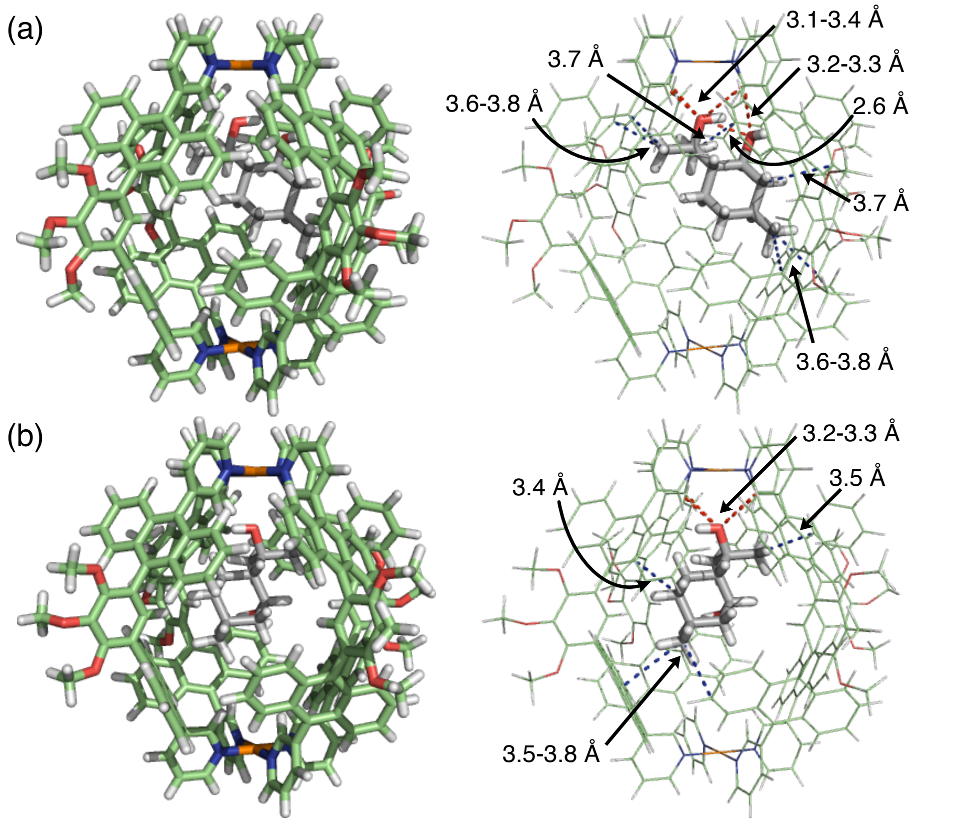

Figure S36. Optimized structures (PM6 calculations, R = -OCH_3) of (a) **1a•*eq*-PMD** and (b) **1a•*ax*-PMD**, and their host-guest interactions (blue dotted lines: CH-π interactions, red dotted lines: hydrogen-bonding interactions).

Table S1. The calculated energies (kJ/mol, DFT calculations) of *eq*-**PMD** and *ax*-**PMD** before and after encapsulation by **1a** (R = -OCH_3).

G	E_H	E_G	$E_{H \cdot G}$	$\Delta E_{H \cdot G}$
eq-PMD	–23157673.7	–1426533.0	–24584280.8	–74.2
ax-PMD	–23157673.7	–1426516.6	–24584233.6	–43.4

Acid-catalyzed reaction of CAL within solid (1b)$_n$•(TS)$_x$ RS753

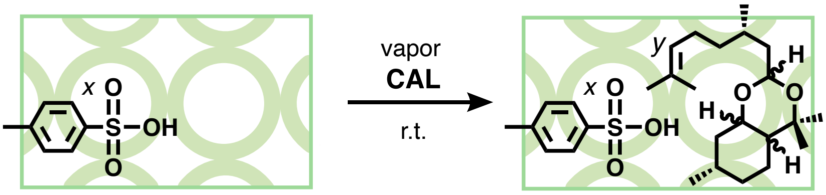

A diethyl ether solution (8.9 µL) of **TS** (0.7 equiv. for **1b**) was added to solid (**1b**)$_n$ (0.6 mg, 0.2 µmol) at r.t. and then the solvent was removed under vacuum (480 Pa, 30 min) to give solid (**1b**)$_n$•(**TS**)$_x$. A small open vessel including solid (**1b**)$_n$•(**TS**)$_x$ was put in a closed glass vessel (50 mL) including **CAL** (1.5 mg, 9.5 µmol), without direct host-guest contact. After standing 6 h at r.t., the small vessel was taken out from the large vessel. Pale-yellow amorphous resultant solid (**1b**)$_n$•(**TS**)$_x$•(**MCA**)$_y$ was dissolved in CD_3CN (0.4 mL) to reveal the formation of **MCA-a** and **MCA-b** (332% and 87% NMR yields based on **1b** (14% and 4% yields based on **CAL**), respectively) by ^{1}H NMR and CG-MS analyses. On the basis of the ^{1}H NMR signal integration, cyclic dimer **MCA-a** (0.63 µmol) was generated by the use of acid-loaded capsule solid (**1b**)$_n$•(**TS**)$_x$ (0.19 µmol based on **1b**). Thus, (0.63/0.19) × 100 gave 332 yield based on **1b**. The formation of **PMD** was also observed (60% yield based on **1b** (1% yield based on **CAL**)) by ^{1}H NMR analysis. The treatment of **GOL** (2.9 mg, 19.0 µmol) or **COL** (3.1 mg, 20.0 µmol) with solid (**1b**)$_n$•(**TS**)$_x$ was studied under the similar conditions.

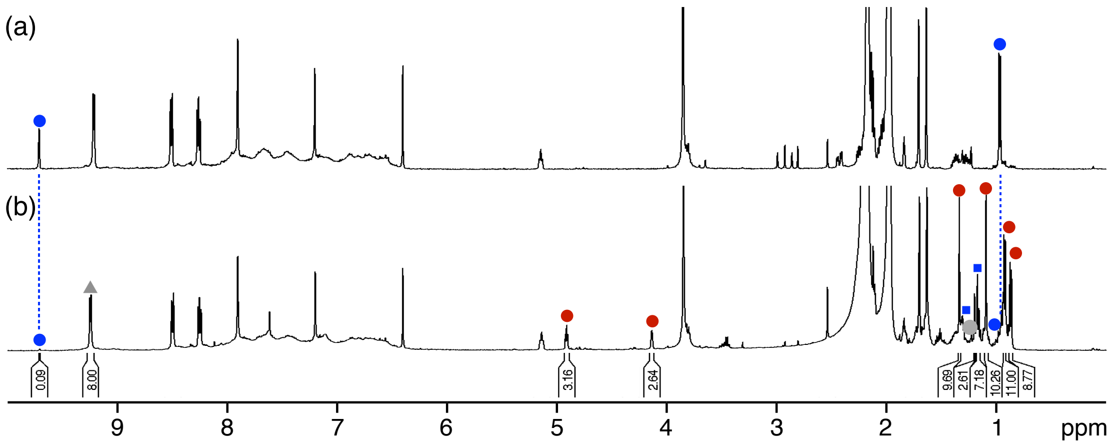

Figure S37a. ^{1}H NMR spectra (500 MHz, CD_3CN, r.t.) of (a) $(\mathbf{1b})_n$•$(\mathbf{CAL})_m$ and (b) products after reaction of **CAL** within $(\mathbf{1b})_n$•$(\mathbf{TS})_x$ at r.t. for 6 h (red circle: **MCA-a**, gray circle: **MCA-b**, blue circle: **CAL**, blue square: **PMD**, grey triangle: host reference signal (H_g)).

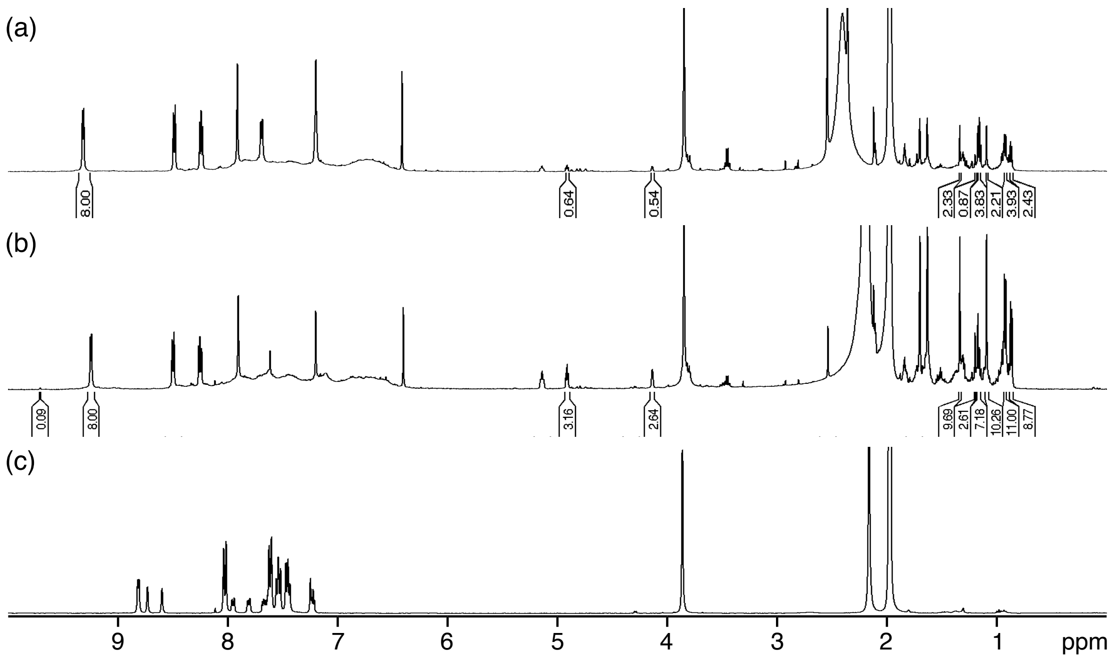

Figure S37b. Time-dependent ^{1}H NMR spectra (500 MHz, CD_3CN, r.t.) of products after reaction of **CAL** within $(\mathbf{1b})_n$•$(\mathbf{TS})_x$ at r.t. for (a) 4 h and (b) 6 h (NMR yields of **MCA-a**: 76% and 332%, respectively). (c) ^{1}H NMR spectra (400 MHz, CD_3CN, r.t.) of **2b**.

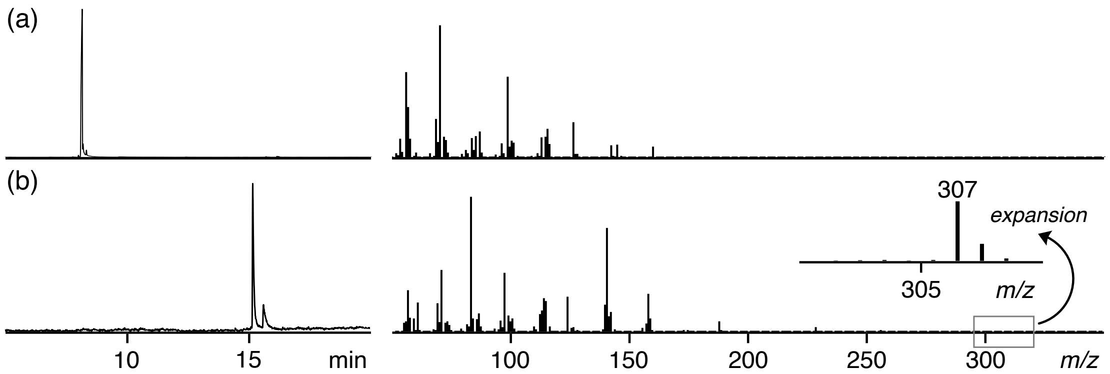

Figure S37c. GC-MS data (left: GC charts, right: MS spectra) of (a) **CAL** and (b) products after reaction of **CAL** within $(\mathbf{1b})_n$•$(\mathbf{TS})_x$ at r.t. for 6 h.

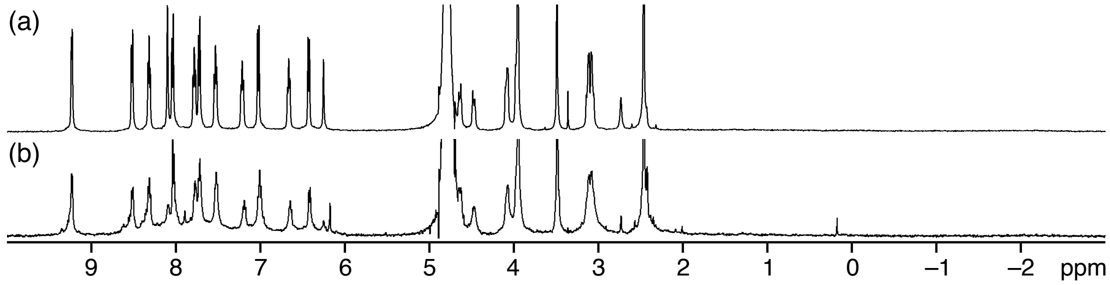

Figure S37d. ^{1}H NMR spectra (500 MHz, D_2O, r.t.) of (a) **1a** and (b) **1a** + **TS** at r.t. for 1 h.

Synthesis and structural analysis of MCA-a

RS760

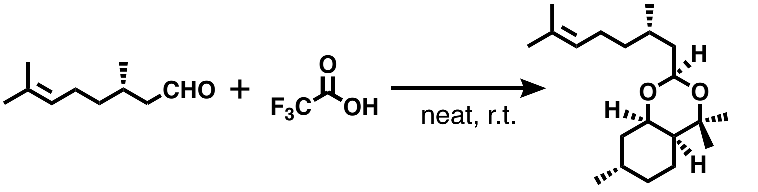

Trifluoroacetic acid (**TFA**; 148 mg, 1.28 mmol) was added to a screw test tube containing **CAL** (500 mg, 3.2 mmol). After the mixture was stirred at r.t. for 3 h, $CHCl_3$ (2 mL) and H_2O (4 mL) were added to the mixture. The organic layer was separated, dried over $MgSO_4$, filtrated, and then concentrated under reduced pressure to give a colorless oil. *p*-Menthane-3,8-diol citronellal acetal (**MCA-a**) was isolated as a colorless oil (32.7 mg, 0.11 mmol, 7%) by recycling GPC ($CHCl_3$).[S8]

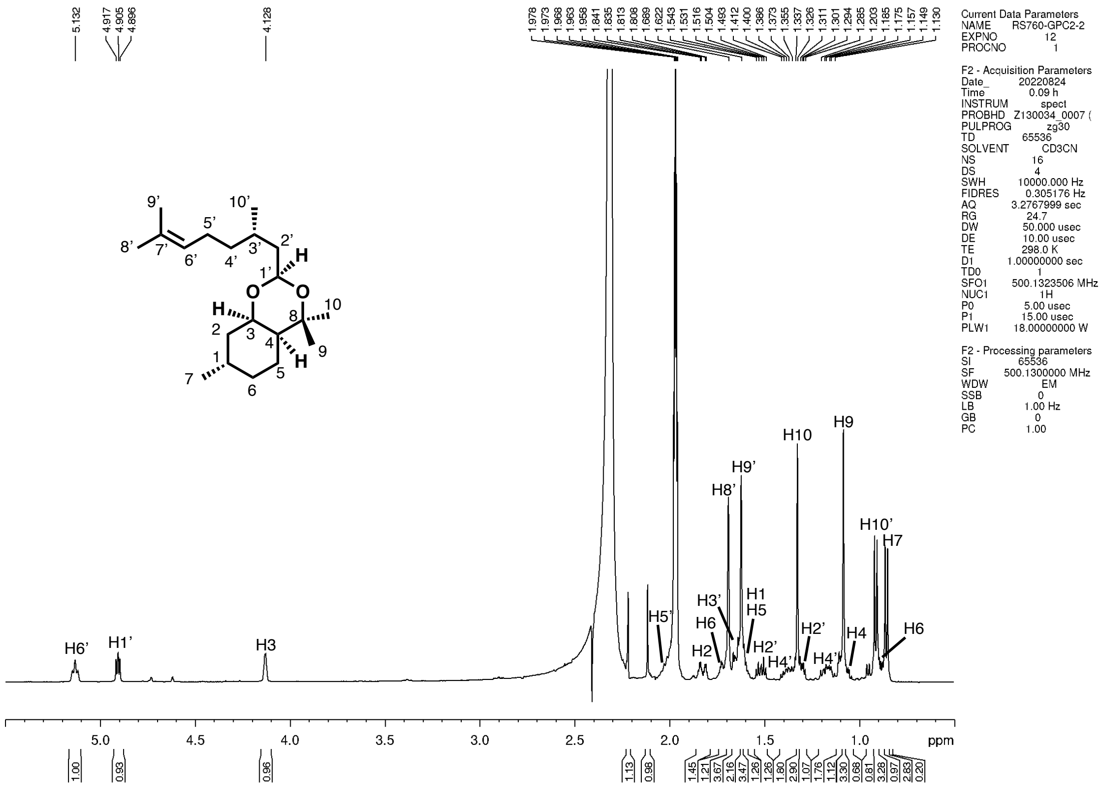

Figure S38. ^{1}H NMR spectrum (500 MHz, CD_3CN, r.t.) of **MCA-a**.

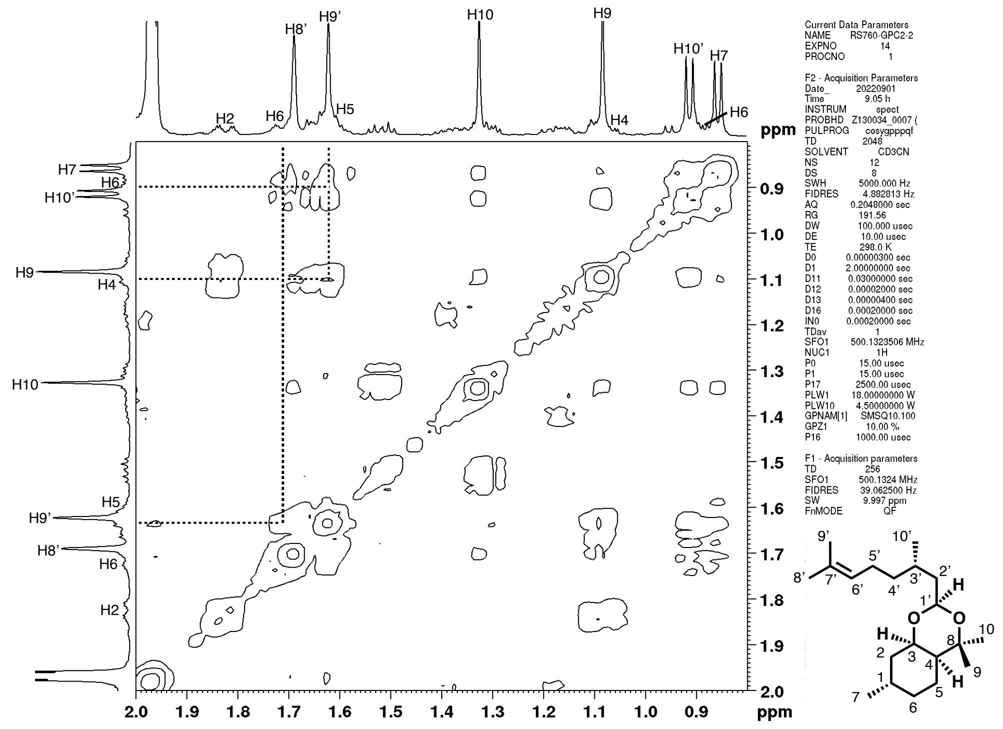

Figure S39a. ^{1}H-^{1}H COSY NMR spectrum (500 MHz, CD_3CN, r.t.) of **MCA-a**.

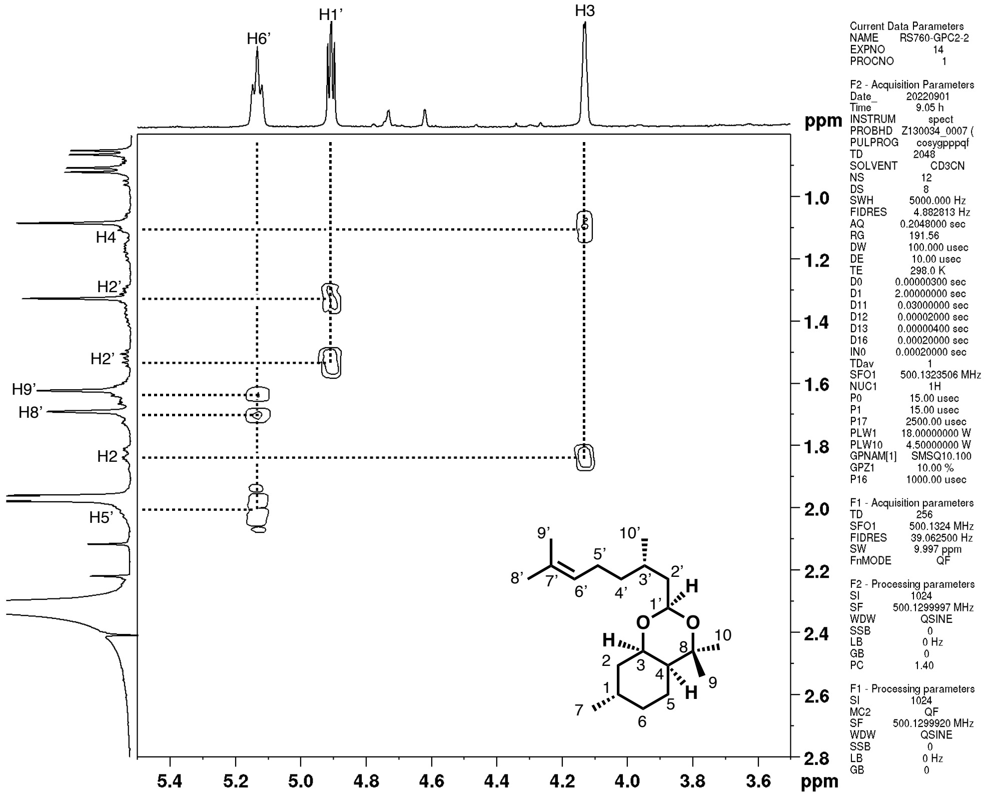

Figure S39b. ^{1}H-^{1}H COSY NMR spectrum (500 MHz, CD_3CN, r.t.) of **MCA-a**.

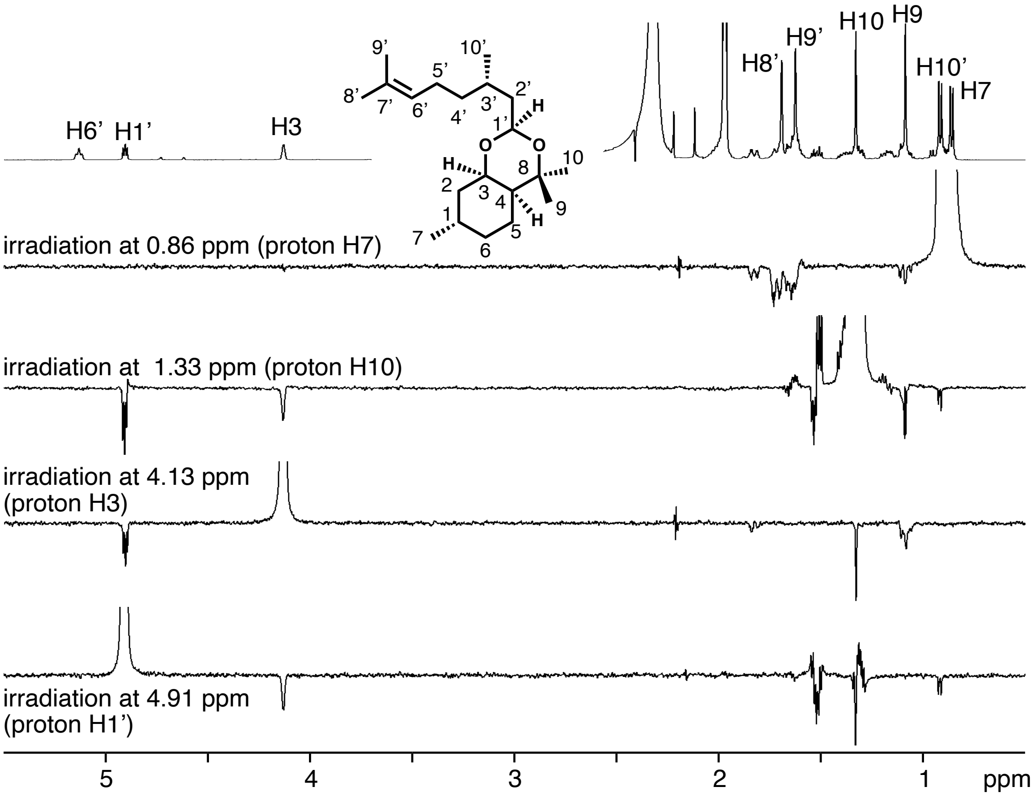

Figure S40. 1D NOESY NMR spectra (500 MHz, CD_3CN, r.t.) of **MCA-a**.

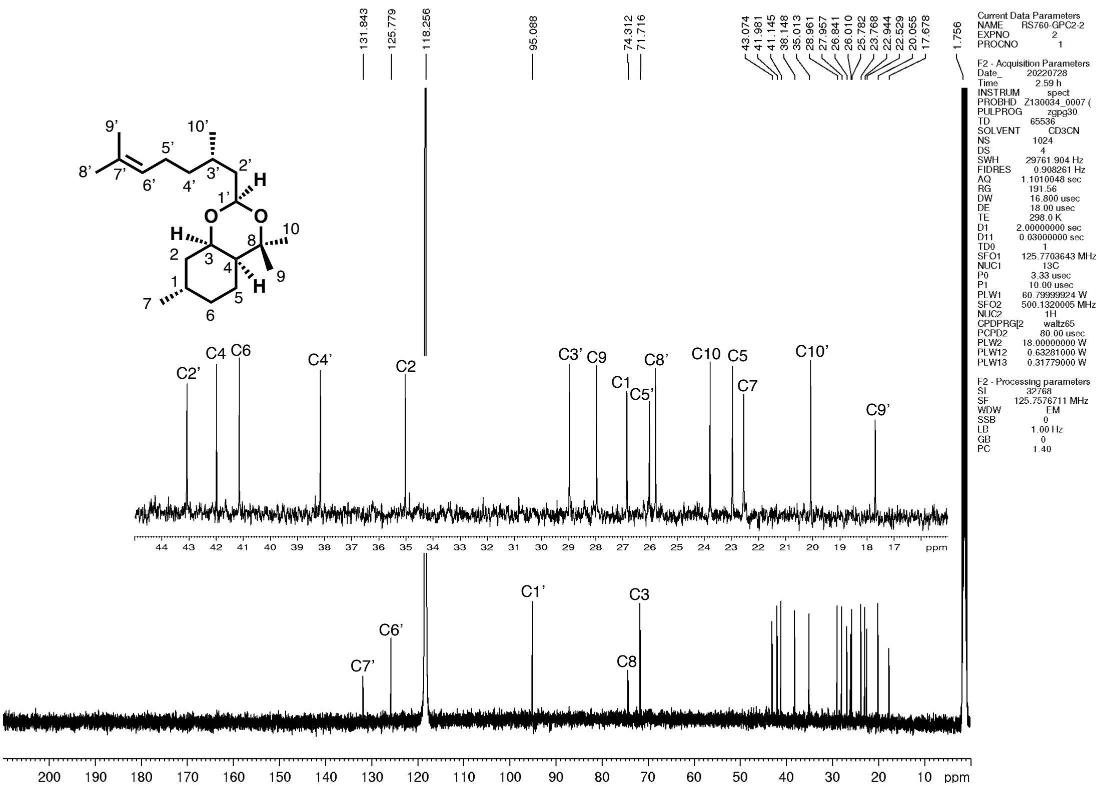

Figure S41. $^{13}C\{^1H\}$ NMR spectrum (126 MHz, CD_3CN, r.t.) of **MCA-a**.

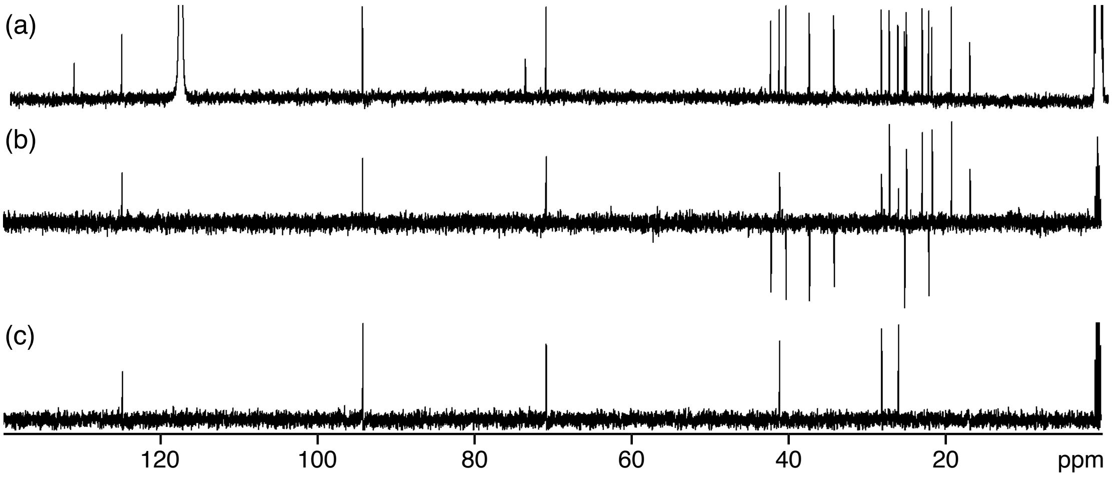

Figure S42. (a) $^{13}C\{^1H\}$ NMR, (b) DEPT135, and (c) DEPT90 spectra (126 MHz, CD_3CN, r.t.) of **MCA-a**.

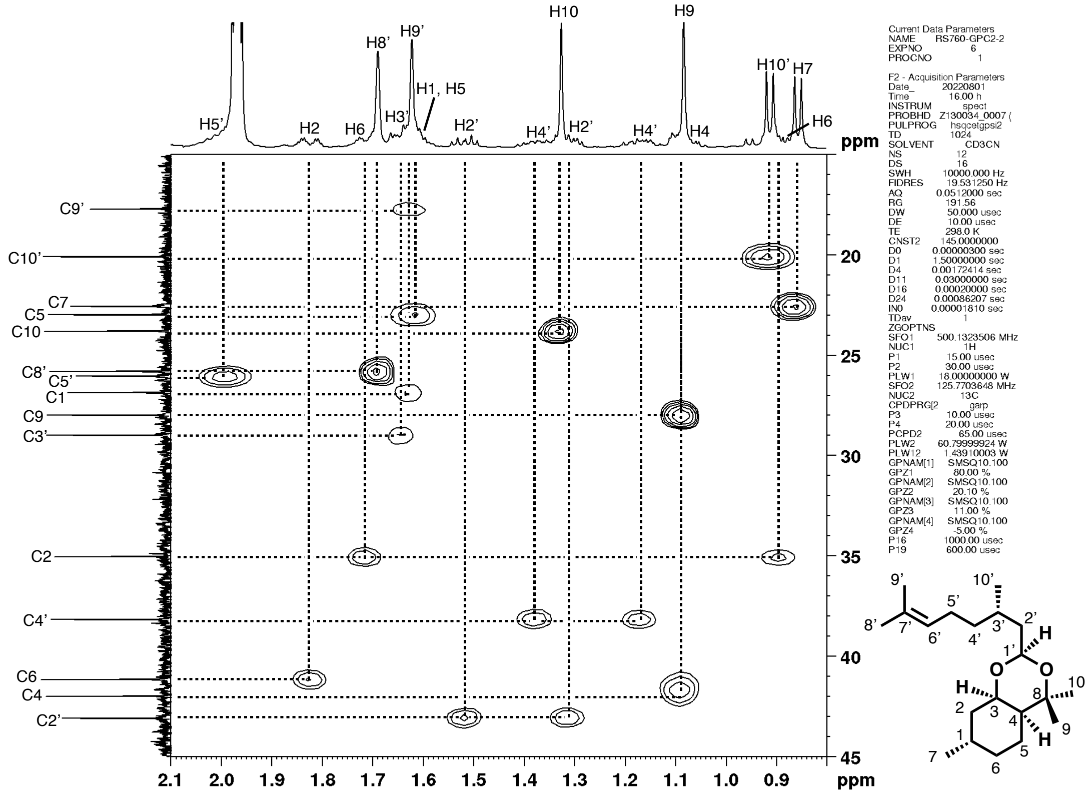

Figure S43a. HSQC NMR spectrum (500 MHz, CD_3CN, r.t.) of **MCA-a**.

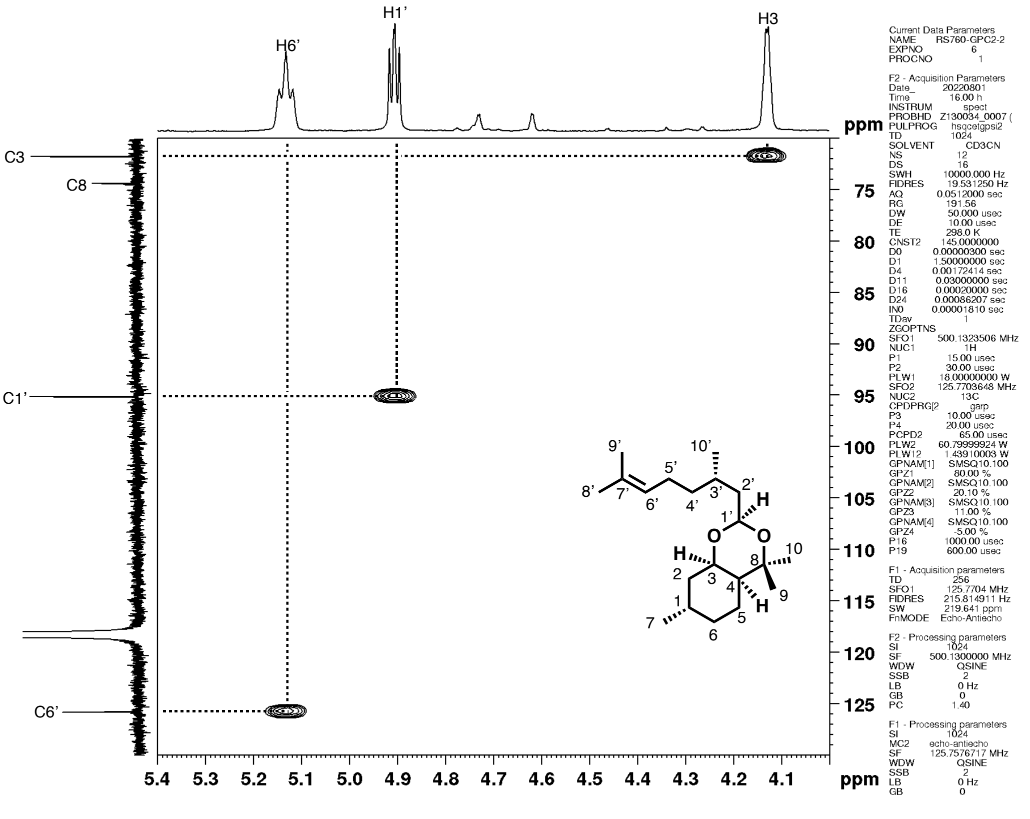

Figure S43b. HSQC NMR spectrum (500 MHz, CD_3CN, r.t.) of **MCA-a**.

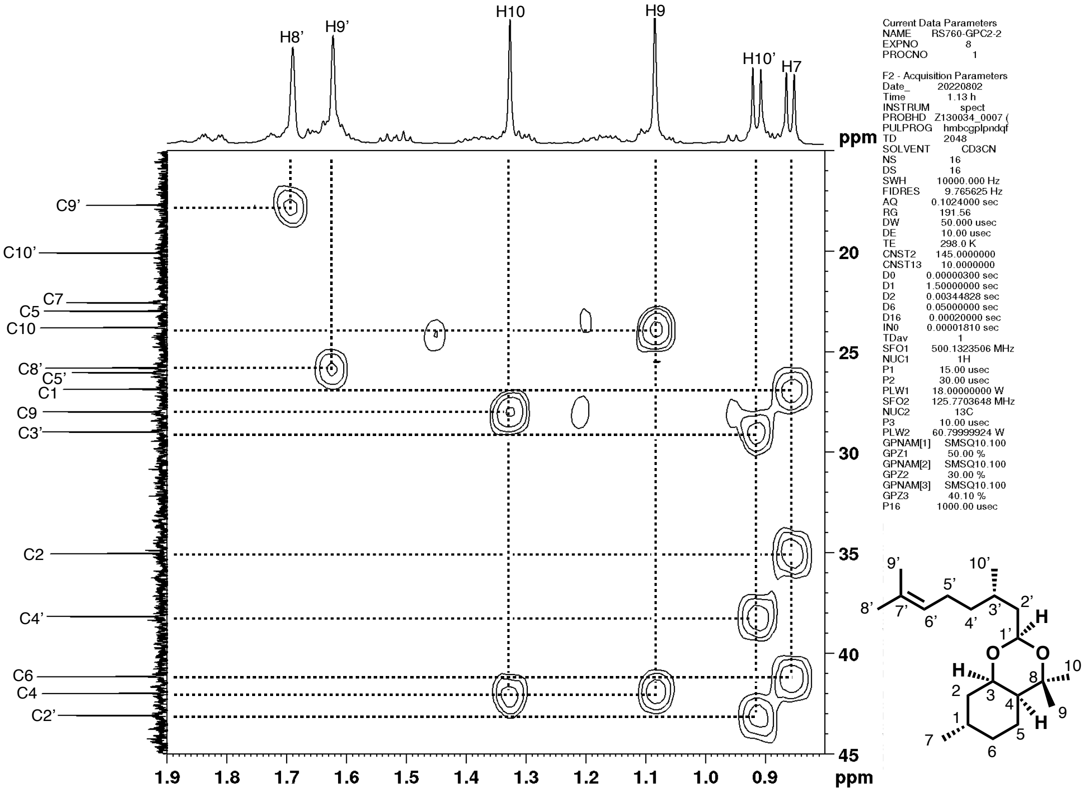

Figure S44a. HMBC NMR spectrum (126 MHz, CD_3CN, r.t.) of **MCA-a**.

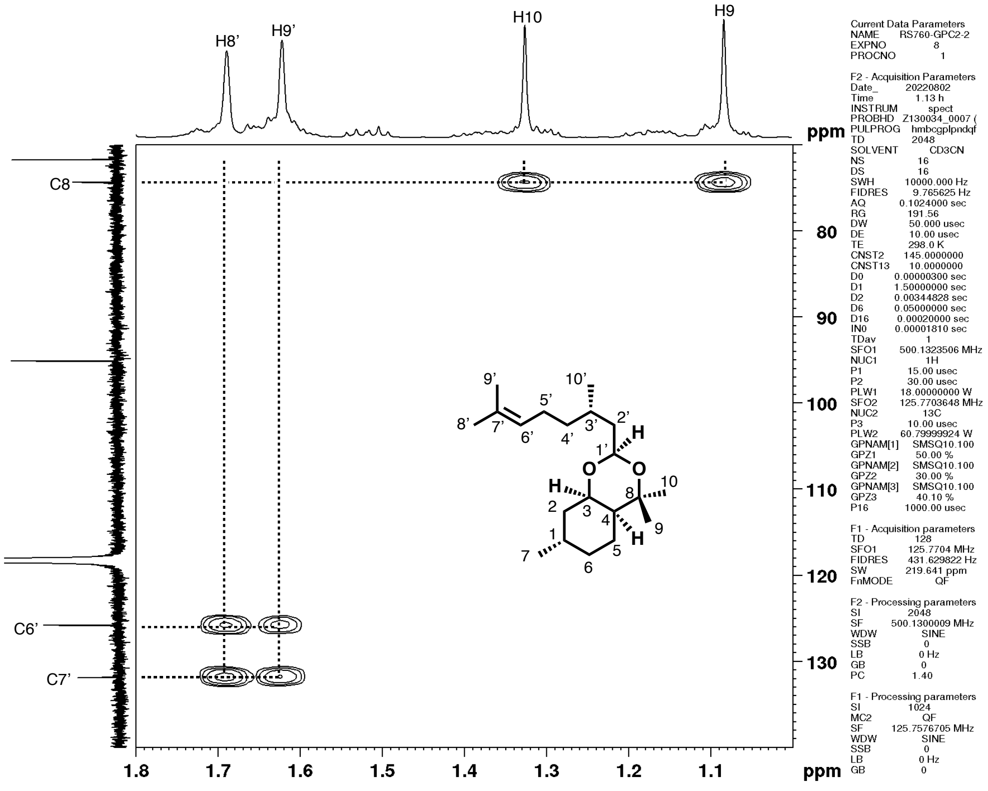

Figure S44b. HMBC NMR spectrum (126 MHz, CD_3CN, r.t.) of **MCA-a**.

Analysis Info | Acquisition Date 2023/04/26 15:18:52

Analysis Name	D:\Data\akita\19sumida\RS760GPC2-2\CH3CN_120V_300Vpp.d			
Method	esi_posi_low.m	Operator	BDAL@DE	
Sample Name	CH3CN_120V_300Vpp	Instrument	micrOTOF	213750.10321
Comment				

Acquisition Parameter

Source Type	ESI	Ion Polarity	Positive	Set Nebulizer	0.3 Bar
Focus	Not active			Set Dry Heater	180 • ‹C
Scan Begin	50 m/z	Set Capillary	4500 V	Set Dry Gas	4.0 l/min
Scan End	1000 m/z	Set End Plate Offset	-500 V	Set Divert Valve	Waste

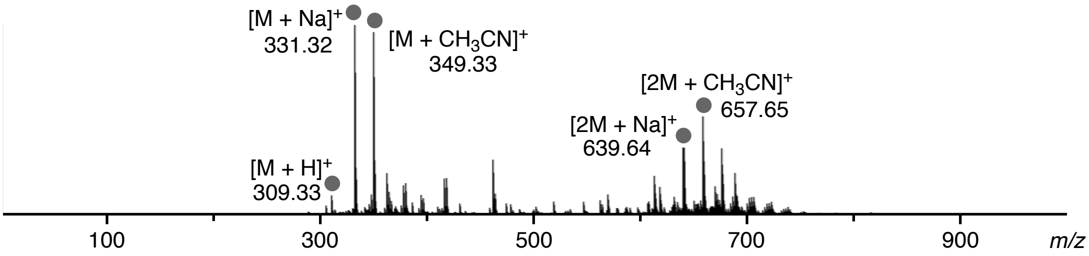

Figure S45. ESI-TOF MS spectrum (CH_3CN) of **MCA-a**.

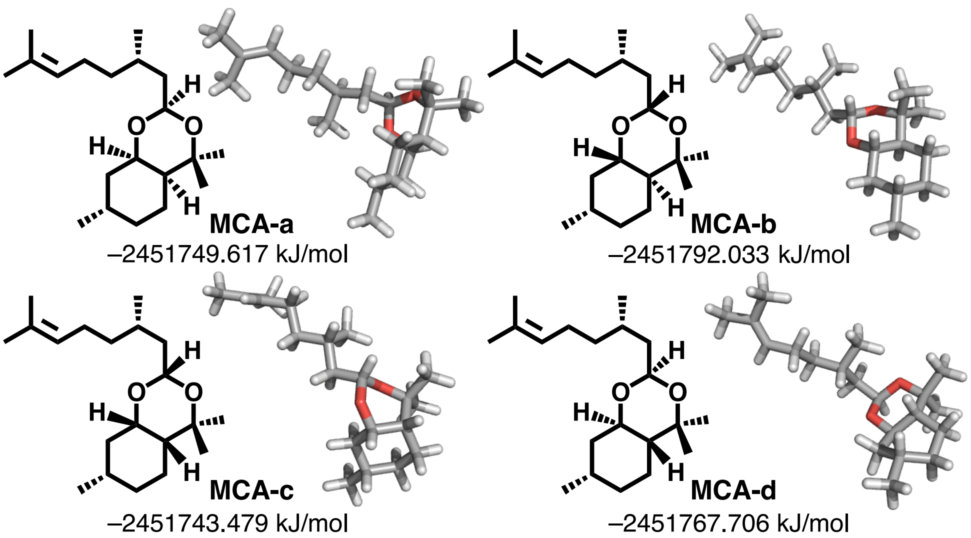

Figure S46. Optimized structures (DFT calculations) of **MCA** isomers and their energies.

Formation of 1a•MCA-a RS813, 958

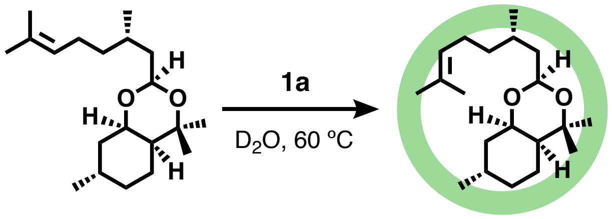

Capsule **1a** (1.0 mg, 0.26 μmol) and **MCA-a** (excess) were added to a 2 mL test tube containing D_2O (0.5 mL). The mixture was stirred at 60 ºC (oil bath) for 1 h. The quantitative formation of pale-yellow 1:1 host-guest complex **1a•MCA-a** was confirmed by NMR and ESI-TOF MS analyses. The mixing of **1a•MCA-a** (0.26 μmol) and **CAL** (0.4 mg, 2.5 μmol) at r.t. for 4 h gave rise to **1a•CAL** through guest-exchange, as confirmed by ^{1}H NMR analysis.

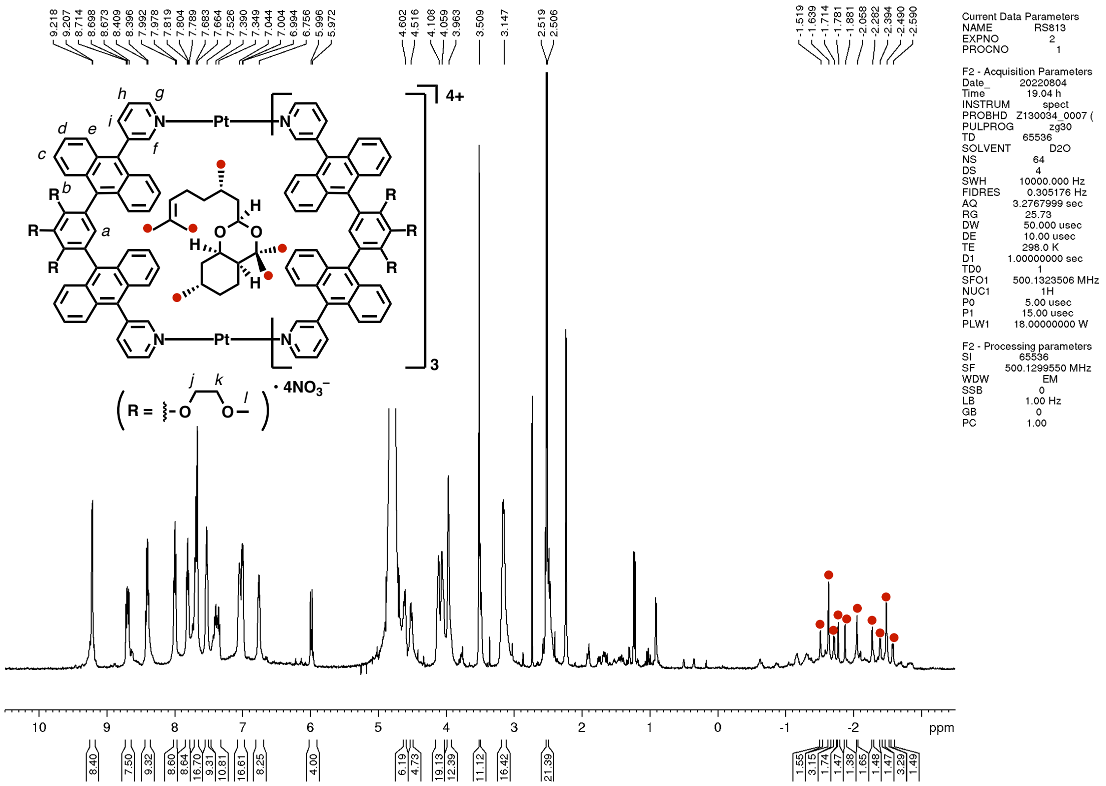

Figure S47a. ^{1}H NMR spectrum (500 MHz, D_2O, r.t.) of **1a•MCA-a**.

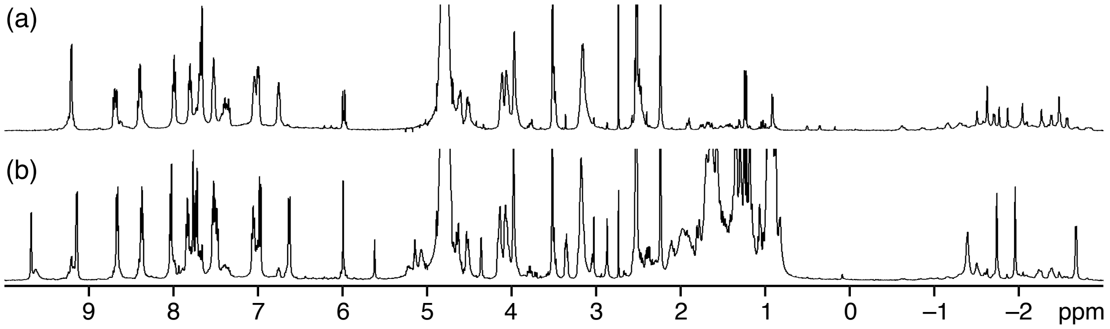

Figure S47b. ^{1}H NMR spectra (500 MHz, D_2O, r.t.) of (a) **1a•MCA-a** and (b) products after mixing **1a•MCA-a** and **CAL** at r.t. for 4 h.

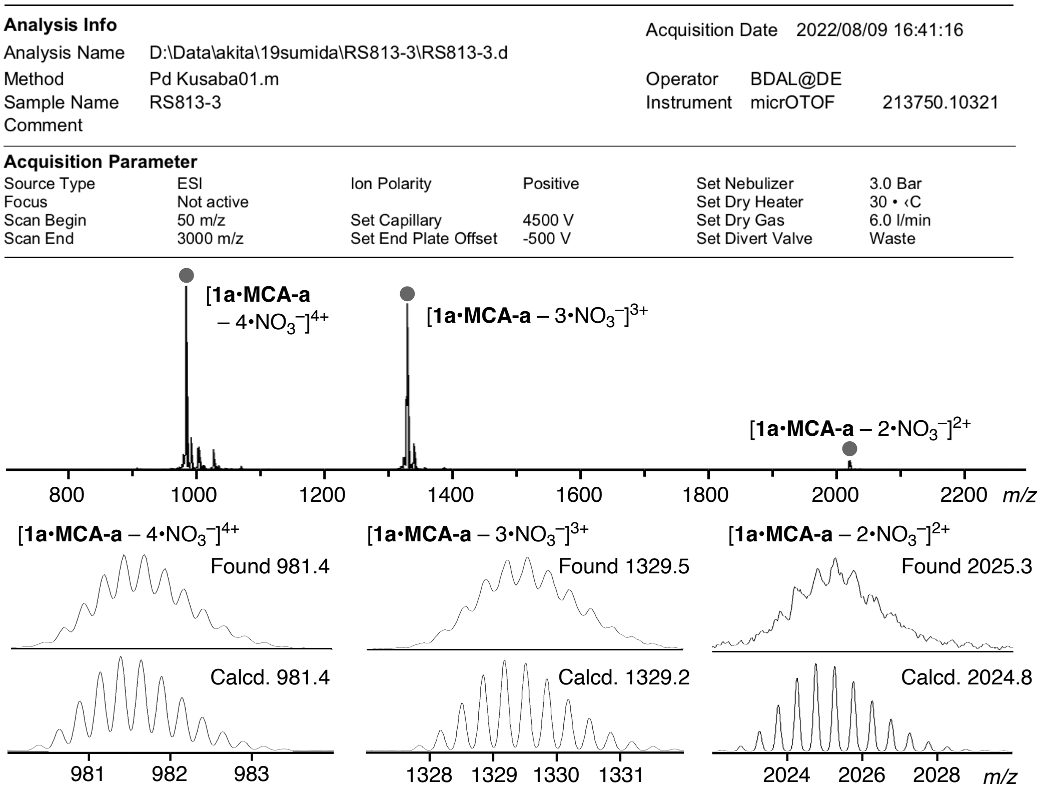

Figure S48. ESI-TOF MS spectrum (H_2O) of **1a•MCA-a**.

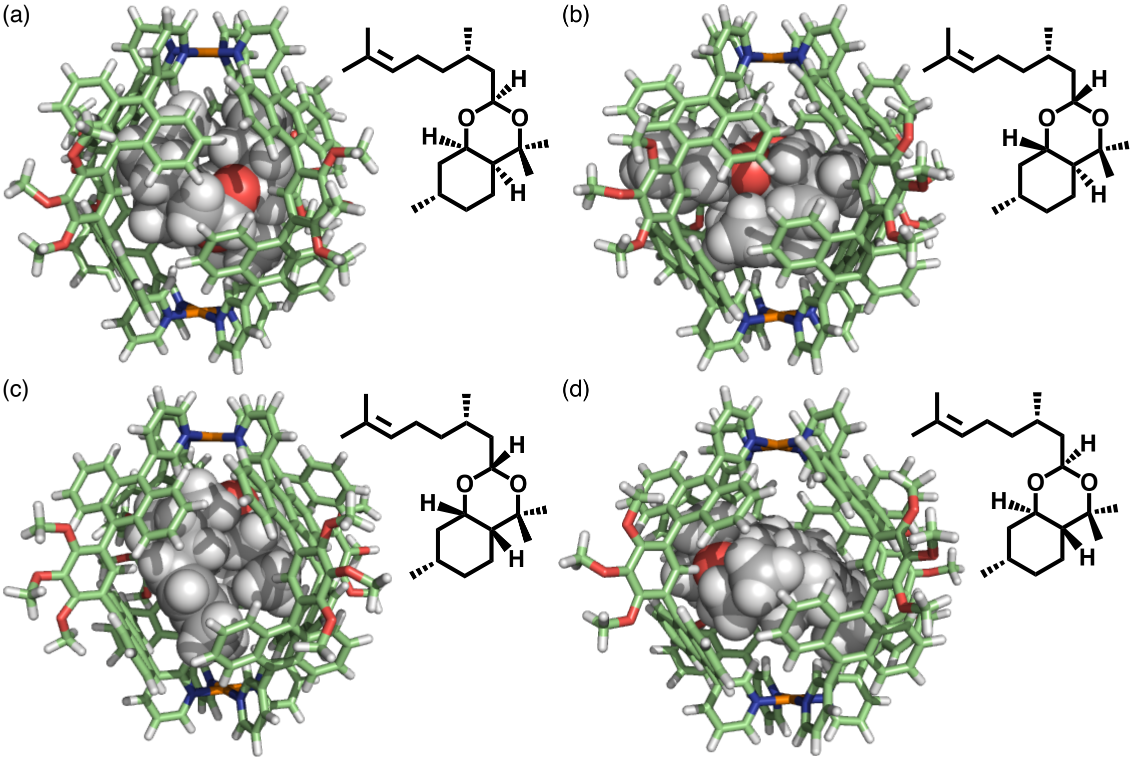

Figure S49. Optimized structures (PM6 calculations; R = -OCH_3) of (a) **1a•MCA-a**, (b) **1a•MCA-b**, (c) **1a•MCA-c**, and (d) **1a•MCA-d**.

Table S2. The calculated energies (kJ/mol, DFT calculations) of **MCA** isomers before and after encapsulation by **1a** (R = -OCH_3).

G	E_{H}	E_{G}	$E_{H\cdot G}$	$\Delta E_{H\cdot G}$
**MCA-a**	–23157673.7	–2451749.6	–25609538.2	–114.9
**MCA-b**	–23157673.7	–2451792.0	–25609527.0	–61.3
**MCA-c**	–23157673.7	–2451743.5	–25609496.9	–79.7
**MCA-d**	–23157673.7	–2451767.7	–25609511.9	–70.5

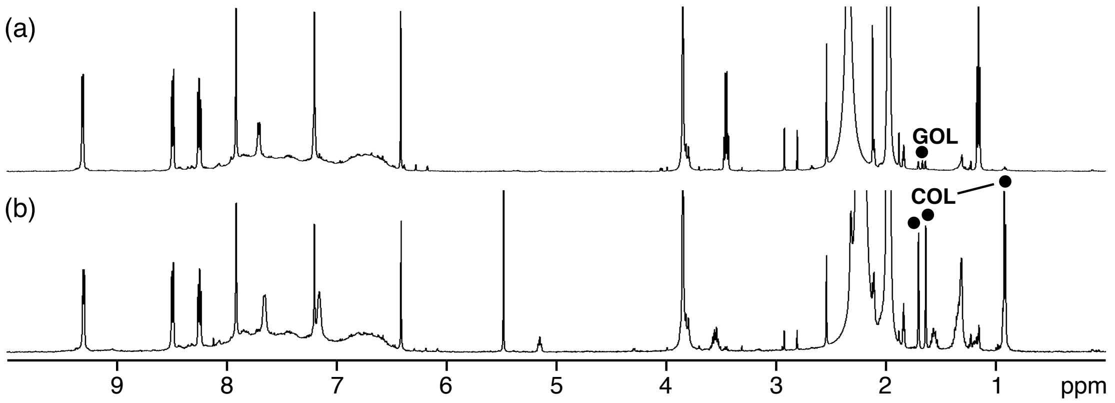

Figure S50. ^{1}H NMR spectra (500 MHz, CD_3CN, r.t.) of products after reaction of (a) **GOL** and (b) **COL** within $(\mathbf{1b})_n$•$(\mathbf{TS})_x$ at r.t. for 6 h.

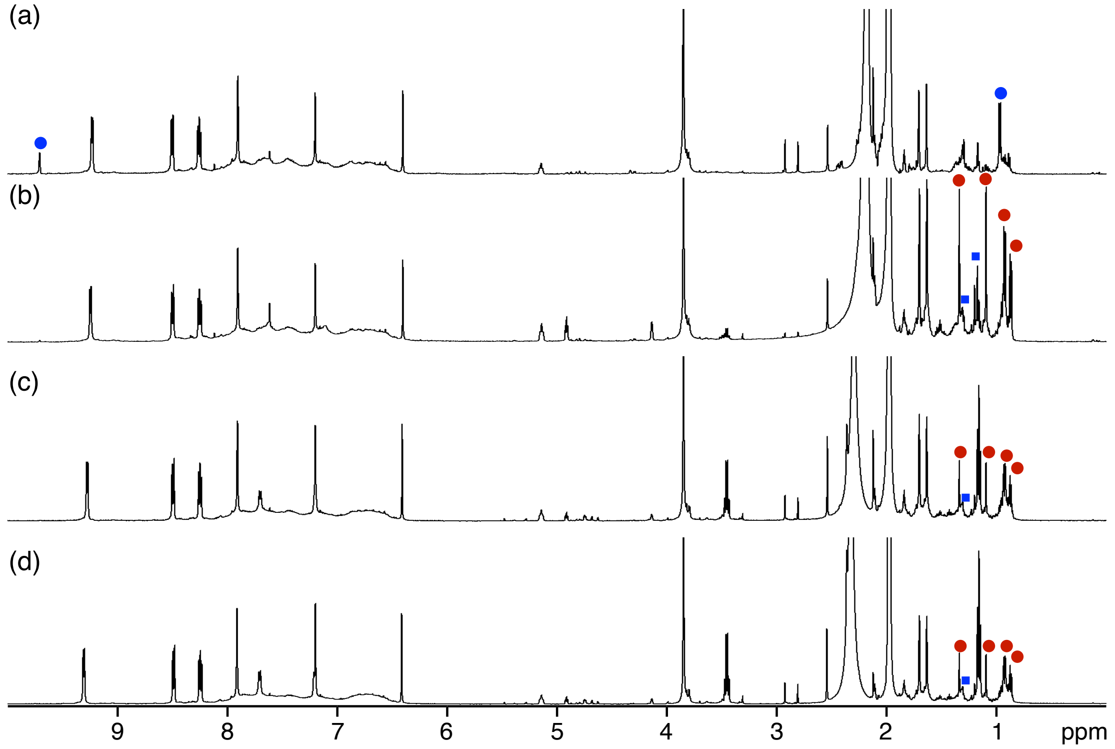

Figure S51a. ^{1}H NMR spectra (500 MHz, CD_3CN, r.t.) of products after reaction of **CAL** within $(\mathbf{1b})_n$•$(\mathbf{TS})_x$, including various equivalent of **TS** at r.t. for 6 h: (a) 0.4, (b) 0.7, (c) 1.2, and (d) 2.0 eq. based on **1b** (red circle: **MCA-a**, blue circle: **CAL**, blue square: **PMD**).

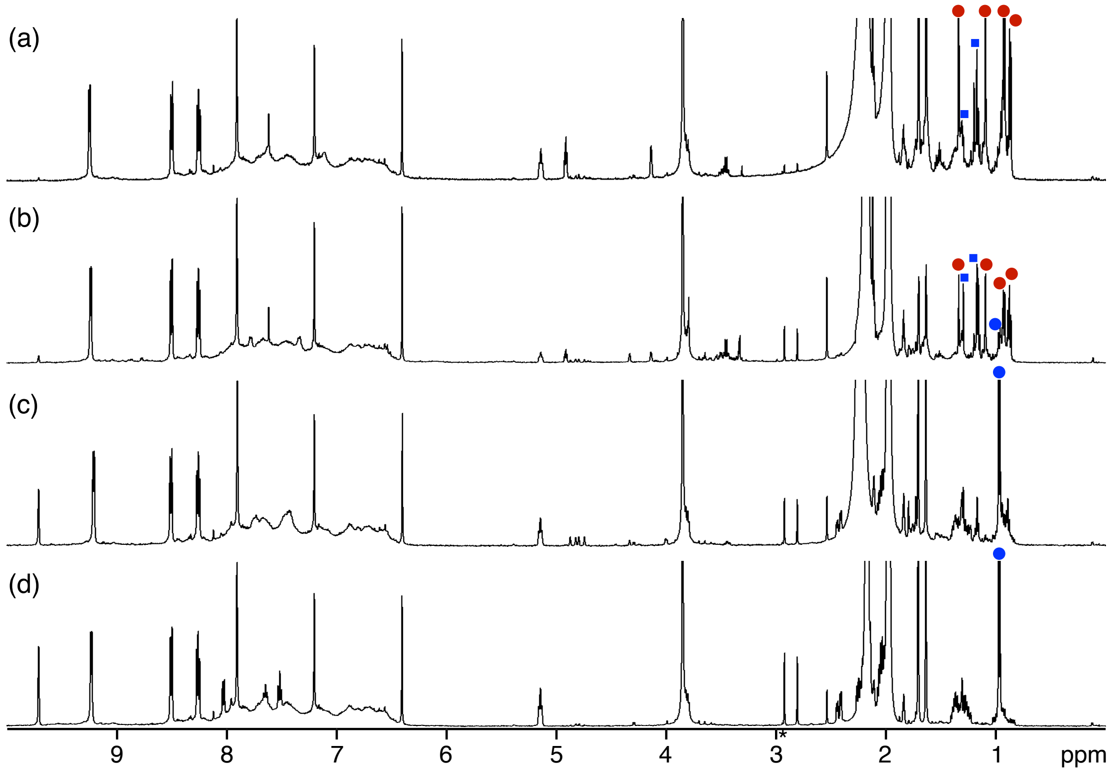

Figure S51b. ^{1}H NMR spectra (500 MHz, CD_3CN, r.t.) of products after reaction of **CAL** within (a) $(\mathbf{1b})_n$•$(\mathbf{TS})_x$, (b) $(\mathbf{1b})_n$•$(\mathbf{BS})_x$, (c) $(\mathbf{1b})_n$•$(\mathbf{BP})_x$, and (d) $(\mathbf{1b})_n$•$(\mathbf{BA})_x$ at r.t. for 6 h (red circle: **MCA-a**, blue circle: **CAL**, blue square: **PMD**).

Acid-catalyzed reactions of CAL RS728

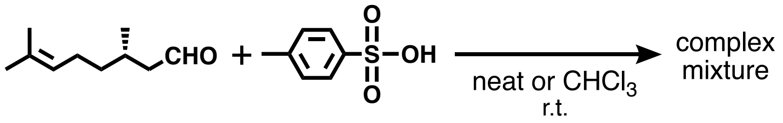

CAL (42.8 mg, 277 μmol) and **TS** (23.7 mg, 138 μmol) were added to a 2 mL test tube. The mixture was stirred at r.t. for 1 d under neat conditions. The generation of a complex mixture of acid-catalyzed products was confirmed by ^{1}H NMR and GC-MS analyses. In a similar way, a complex mixture of acid-catalyzed products was obtained by stirring a mixture of **CAL** (42.8 mg, 277 μmol) and **TS** (23.0 mg, 134 μmol) in $CHCl_3$ (50 μL) at r.t. for 2 h.

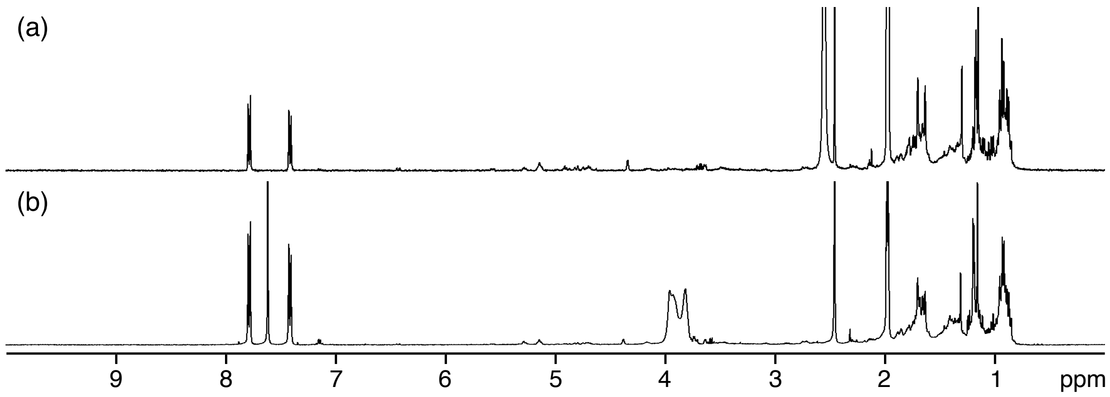

Figure S52a. ^{1}H NMR spectra (400 MHz, CD_3CN, r.t.) of products after stirring a mixture of **CAL** and **TS** (a) without and (b) with solvent ($CHCl_3$) at r.t.

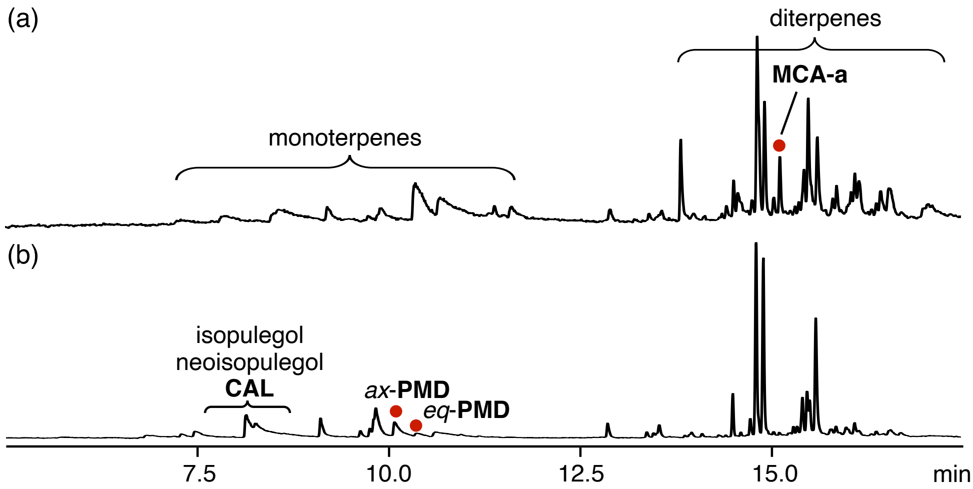

Figure S52b. GC charts of products after stirring a mixture of **CAL** and **TS** (a) without and (b) with solvent ($CHCl_3$) at r.t.

Acid-catalyzed reaction of CAL using (1'•C_{60})$_n$ or other hosts RS772, 822, 839, 1131, 1132

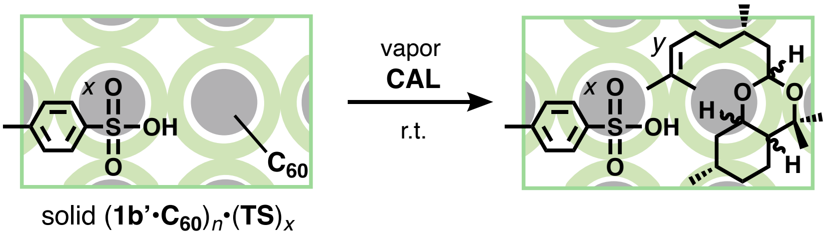

A diethyl ether solution (14.7 μL) of **TS** (0.7 equiv. based on **1b'•C_{60}**) was added to host-guest solid (**1'•C_{60}**)$_n$, prepared from **1'** (1.0 mg, 0.33 μmol) and fullerene C_{60} (**C_{60}**; 0.80 mg, 1.1 μmol), at r.t. and then the solvent was removed under vacuum (480 Pa, 30 min) to give solid (**1b'•C_{60}**)$_n$•(**TS**)$_x$. A small open vessel including solid (**1b'•C_{60}**)$_n$•(**TS**)$_x$ was put in a closed glass vessel (50 mL) including **CAL** (2.5 mg, 16.5 μmol), without

direct host-guest contact. After standing at r.t. for 6 h, the small vessel was taken out from the large vessel and then placed at r.t. for 1 h under vacuum (480 Pa). Resultant purple amorphous solid (**1b'•C60**)$_n$•(**TS**)$_x$•(**MCA**)$_y$ was dissolved in CD_3CN (0.4 mL) to reveal the formation of **MCA-a** and **MCA-b** (163% and 39% yield based on **1b'•C60**, respectively) by ^{1}H NMR analysis. Under similar conditions, acid-catalyzed reaction of **CAL** was examined using **TS** (0.7 equiv. for **1b** (0.19 μmol)) without the host compound. Similar reactions were examined using γ-cyclodextrin (**γCD**; 0.34 mg, 0.26 μmol), cucurbit[6]uril (**CB6**; 0.36 mg, 0.36 μmol), or pillar[5]arene (**P5A**; 0.30 mg, 0.40 μmol) as host solids with **TS** (0.7 equiv. for host compounds).

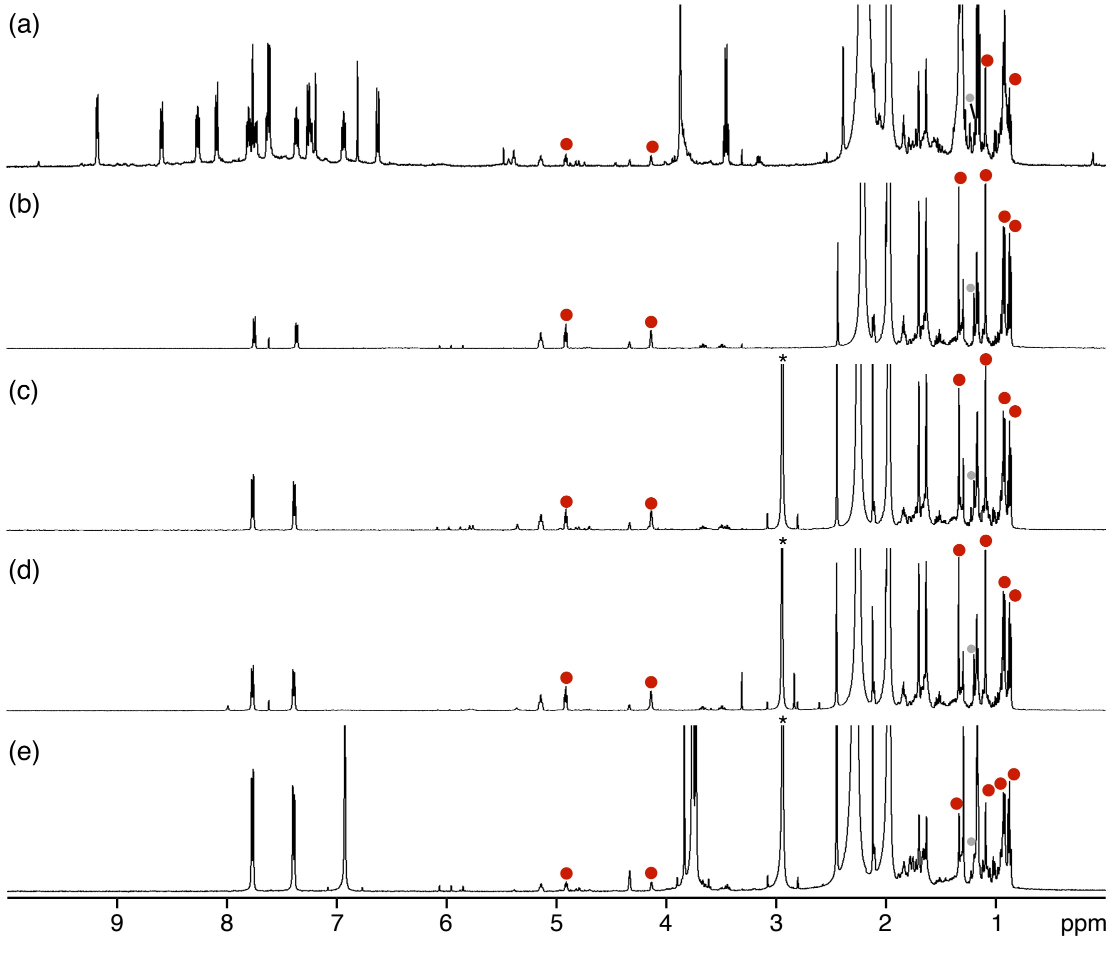

Figure S53. ^{1}H NMR spectra (500 MHz, CD_3CN, r.t.) of products after the reactions of **CAL** with (a) (**1b'•C60**)$_n$•(**TS**)$_x$, (b) **TS**, (c) (**γCD**)$_n$•(**TS**)$_x$, (d) (**CB6**)$_n$•(**TS**)$_x$, and (e) (**P5A**)$_n$•(**TS**)$_x$ at r.t. for 6 h (red circle: **MCA-a**, gray circle: **MCA-b**, *: dimethyl sulfone as an internal standard).

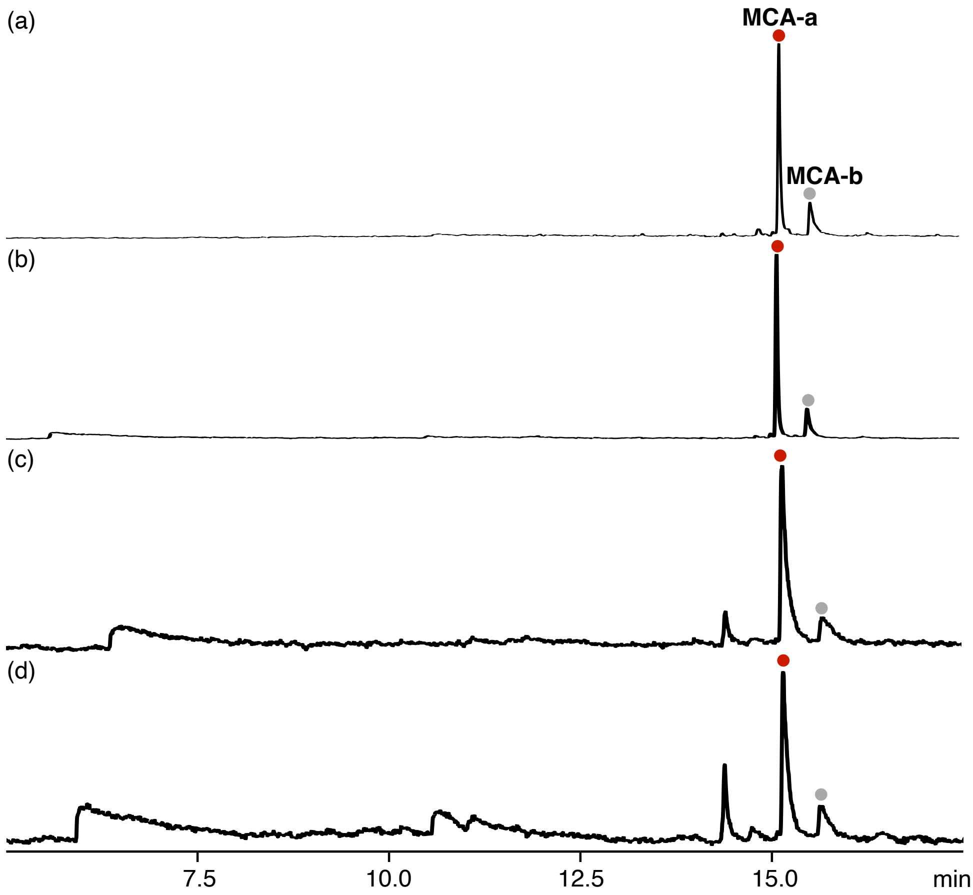

Figure S54. GC charts of products after the reactions of **CAL** with (a) **TS**, (b) **(γCD)**$_n$•**(TS)**$_x$, (c) **(CB6)**$_n$•**(TS)**$_x$, or (d) **(P5A)**$_n$•**(TS)**$_x$ at r.t. for 6 h.

Repeated acid-catalyzed reaction of CAL within solid (1b)$_n$•(TS)$_x$ RS1089

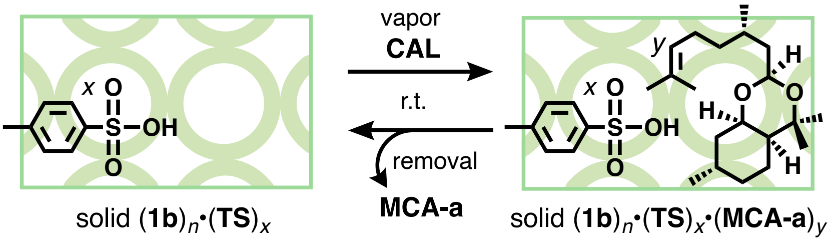

A diethyl ether solution (9.9 μL) of **TS** (0.7 equiv. for **1b**) was added to solid **(1b)**$_n$ (0.6 mg, 0.2 μmol) at r.t. and then the solvent was removed under vacuum (480 Pa, 30 min) to give acid-loaded capsule solid **(1b)**$_n$•**(TS)**$_x$. A small open vessel including solid **(1b)**$_n$•**(TS)**$_x$ was put in a closed glass vessel (50 mL) including **CAL** (1.4 mg, 9.0 μmol), without direct solid-substrate contact. After standing 6 h at r.t., the small vessel was taken out from the large vessel. Resultant pale-yellow amorphous solid **(1b)**$_n$•**(TS)**$_x$•**(MCA)**$_y$ was dissolved in CD_3CN (0.4 mL) to reveal the formation of **MCA** by ^{1}H NMR analysis. After removal of the solvent under reduced pressure (480 Pa at r.t. for 30 min), solid

(1b)$_n$**•(TS)**$_x$**•(MCA)**$_y$ was washed with diethyl ether and CH_3CN to extract **MCA**. The resultant solid (0.6 mg) was reused for the second acid-catalyzed reaction of **CAL** under the same conditions. This procedure was repeated four times, after the addition of **TS** (0.5-0.7 eq. each). The products and their NMR yields for each of the reactions were revealed by ^{1}H NMR and GC-MS analyses.

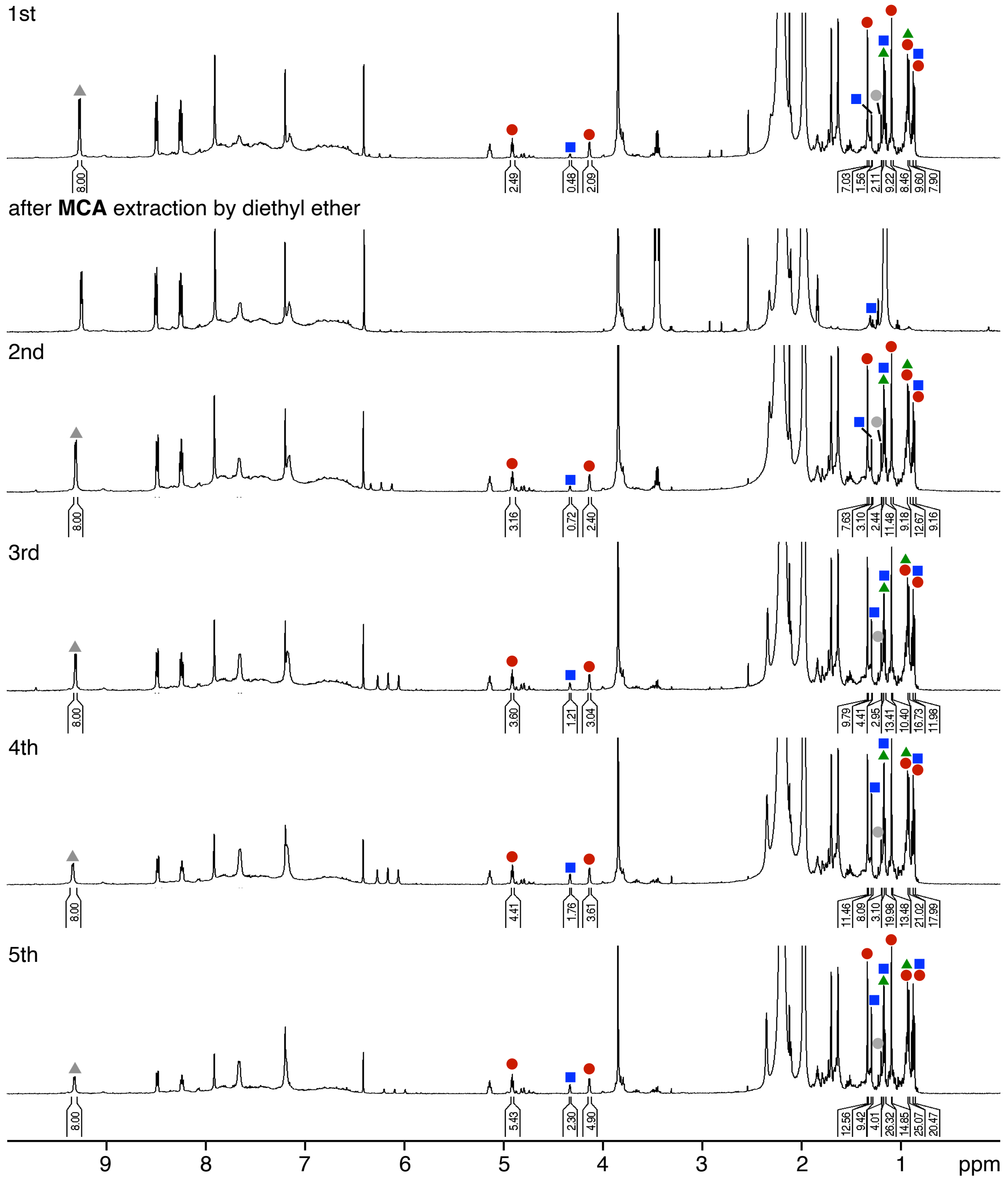

Figure S55. ^{1}H NMR spectra (500 MHz, CD_3CN, r.t.) of products after the repeated acid-catalyzed reaction of **CAL** within **(1b)**$_n$**•(TS)**$_x$ at r.t. for 6 h (red circle: **MCA-a**, gray circle: **MCA-b**, green triangle: *eq*-**PMD**, blue square: *ax*-**PMD**, grey triangle: host reference signal (H_g)).

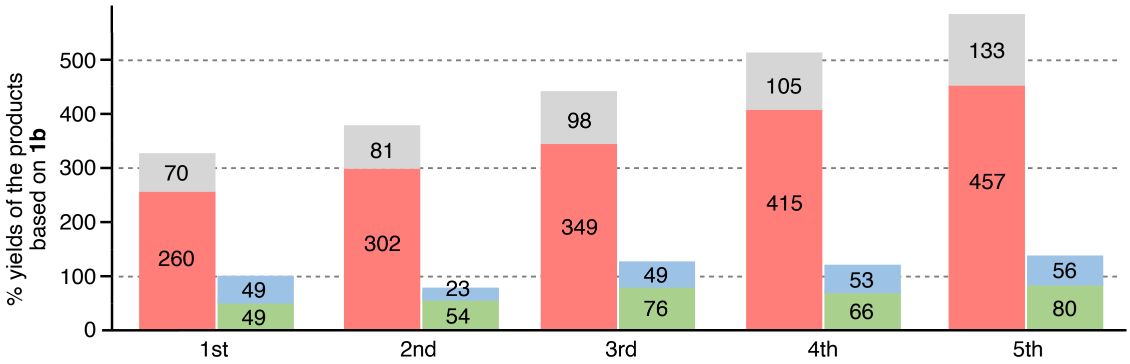

Figure S56. NMR yields (%) of the products (based on **1b**) after the repeated acid-catalyzed reaction of **CAL** within (**1b**)$_n$•(**TS**)$_x$ at r.t. for 6 h (red bar: **MCA-a**, gray bar: **MCA-b**, green bar: *eq*-**PMD**, blue bar: *ax*-**PMD**).

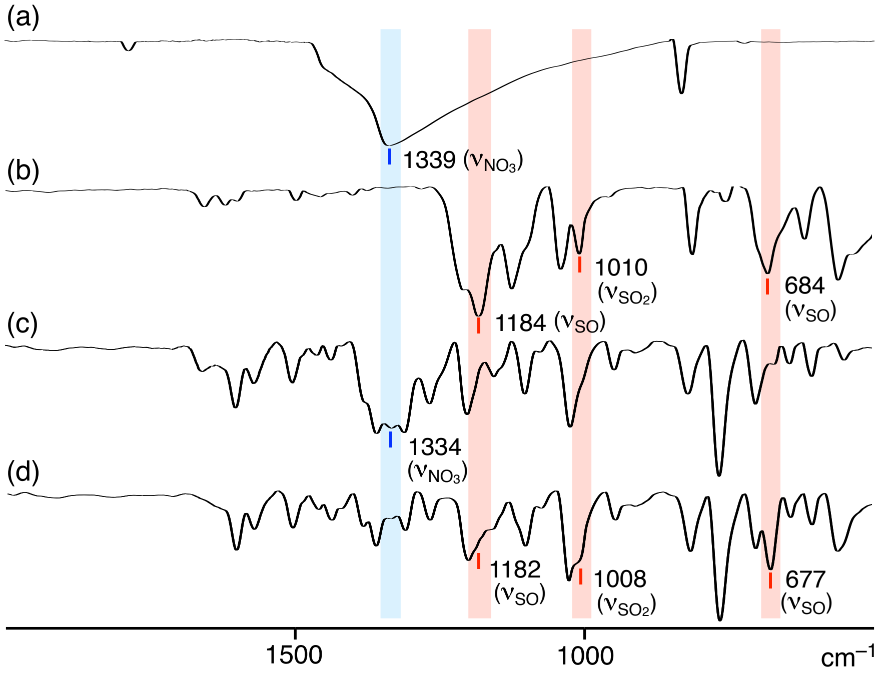

Figure S57. FT-IR (ATR, r.t.) spectra (ATR, r.t.) of (a) $NaNO_3$, (b) sodium *p*-toluenesulfonate (*p*-TsONa), (c) **1b**, and (d) (**1b**)$_n$•(**TS**)$_x$.

Acid-catalyzed reaction from mixtures by solid (1b)$_n$•(TS)$_x$ RS1159

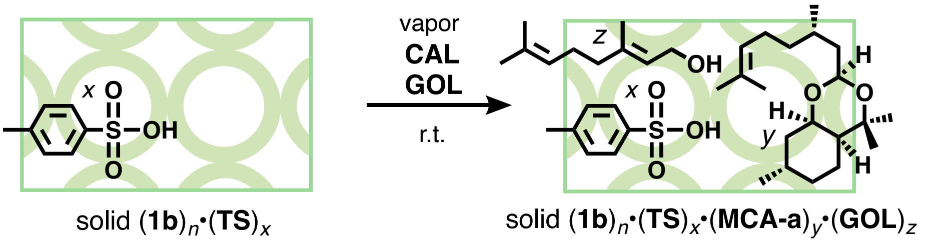

A diethyl ether solution (9.9 μL) of **TS** (0.7 equiv. for **1b**) was added to solid (**1b**)$_n$ (0.6 mg, 0.2 μmol) at r.t. and then the solvent was removed under vacuum (480 Pa, 30 min) to give acid-loaded capsule solid (**1b**)$_n$•(**TS**)$_x$. A small open vessel including solid

(1b)$_n$•**(TS)**$_x$ was put in a closed glass vessel (50 mL) including **CAL** (1.4 mg, 9.0 µmol) and **GOL** (1.4 mg, 9.0 µmol), without direct solid-substrate contact. After standing 6 h at r.t., the small vessel was taken out from the large vessel. Resultant pale-yellow amorphous solid **(1b)**$_n$•**(TS)**$_x$•**(MCA)**$_y$•**(GOL)**$_z$ was dissolved in CD_3CN (0.4 mL) to reveal the formation of **MCA** and binding of **GOL** by ^1H NMR analysis. The amount of bound **CAL** was calculated from the sum of bound **CAL**, **MCA**, and **PMD** in the solid. After removal of the solvent under reduced pressure (480 Pa at r.t. for 30 min), solid **(1b)**$_n$•**(TS)**$_x$•**(MCA)**$_y$•**(GOL)**$_z$ was washed with diethyl ether and CH_3CN to extract **MCA** and **GOL**. The resultant solid (0.6 mg) was reused for the second acid-catalyzed reaction of **CAL** under the same conditions.

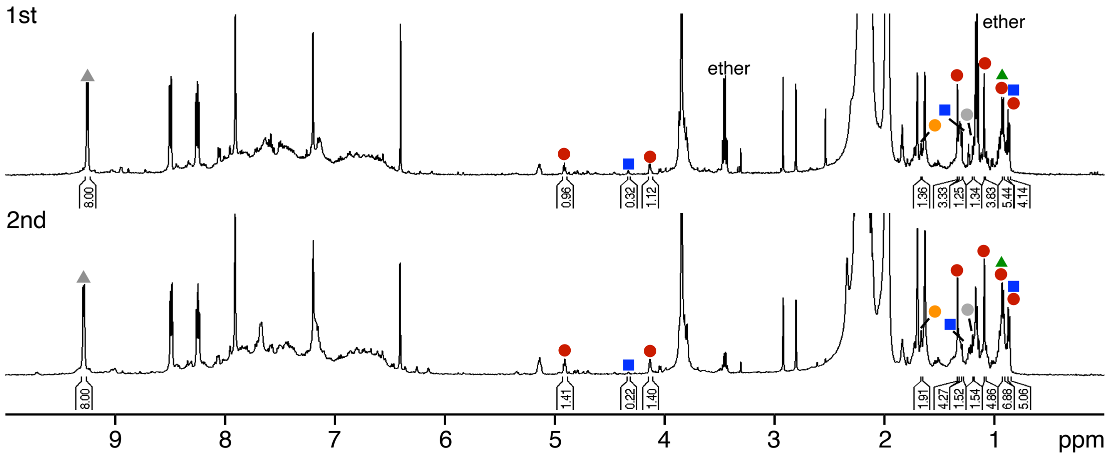

Figure S58a. ^1H NMR spectra (500 MHz, CD_3CN, r.t.) of products after the repeated acid-catalyzed reaction of **CAL** and **GOL** within **(1b)**$_n$•**(TS)**$_x$ at r.t. for 6 h (red circle: **MCA-a**, gray circle: **MCA-b**, orange circle: **GOL**, green triangle: *eq*-**PMD**, blue square: *ax*-**PMD**, grey triangle: host reference signal (H_g)).

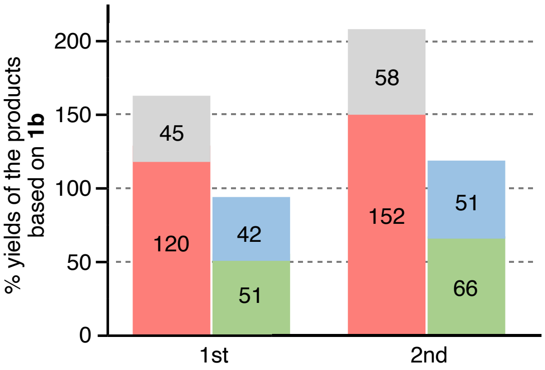

Figure S58b. NMR yields (%) of the products (based on **1b**) after the repeated acid-catalyzed reaction of **CAL** and **GOL** within **(1b)**$_n$•**(TS)**$_x$ at r.t. for 6 h (red bar: **MCA-a**, gray bar: **MCA-b**, green bar: *eq*-**PMD**, blue bar: *ax*-**PMD**).

Table S3. Cyclization-dimerization of vaporized **CAL** using acid-loaded host solids under various conditions.

	n : *x* ratio	time (h)	**yield and TON** based on the host			**conversion** based on **CAL**
			**MCA-a**	**MCA-b**	**PMD**	
$(\mathbf{1b})_n$•$(\mathbf{TS})_x$	1 : 0.7	6	332% (3.3)	87% (0.9)	60% (0.6)	19%
	1 : 0.7	4	76% (0.8)	29% (0.3)	67% (0.7)	6%
	1 : 0.4	6	30% (0.3)	8% (0.1)	58% (0.6)	3%
	1 : 1.2	6	95% (1.0)	32% (0.3)	99% (1.0)	7%
	1 : 2	6	87% (0.9)	35% (0.4)	95% (1.0)	7%
$(\gamma\mathbf{CD})_n$•$(\mathbf{TS})_x$	1 : 0.7	6	120% (1.2)	30% (0.3)	59% (0.6)	7%
$(\mathbf{CB6})_n$•$(\mathbf{TS})_x$	1 : 0.7	6	54% (0.5)	12% (0.1)	43% (0.4)	4%
$(\mathbf{P5A})_n$•$(\mathbf{TS})_x$	1 : 0.7	6	28% (0.2)	5% (0.05)	54% (0.5)	3%
$(\mathbf{1b'}\cdot\mathbf{C_{60}})_n$ •$(\mathbf{TS})_x$	1 : 0.7	6	163% (1.6)	39% (0.4)	108% (1.1)	10%
**TS** (without host)	(0.14 μmol)	6	108%	25% (based on **TS**)	67%	5%
$(\mathbf{1b})_n$•$(\mathbf{BA})_x$	1 : 1.2	6	0%	0%	0%	0%
$(\mathbf{1b})_n$•$(\mathbf{BS})_x$	1 : 0.7	6	80% (0.8)	26% (0.3)	131% (1.3)	7%
$(\mathbf{1b})_n$•$(\mathbf{BP})_x$	1 : 1.2	6	9% (0.09)	7% (0.07)	87% (0.9)	1%

conditions: host compound (0.2 μmol), **CAL** (9.5 μmol), r.t., 1 atom, 50 mL vessel.
yield and conversion estimated by ^{1}H NMR analysis in CD_3CN
BA: benzoic acid, **BS**: benzenesulfonic acid, **BP**: benzenephosphonic acid

Table S4. Cyclization-dimerization of vaporized **CAL** using solid (**1b**)$_n$•(**TS**)$_x$.

repeated time	time (h)	yield and TON based on the host			conversion based on **CAL**
		**MCA-a**	**MCA-b**	**PMD**	
1st	6	260% (2.6)	70% (0.7)	98% (1.0)	17%
2nd	6	302% (3.0)	81% (0.8)	77% (0.8)	19%
3rd	6	349% (3.5)	98% (1.0)	125% (1.3)	23%
4th	6	415% (4.2)	105% (1.1)	119% (1.2)	26%
5th	6	457% (4.6)	133% (1.3)	136% (1.4)	29%

conditions: **1b** (0.2 μmol), **TS** (0.5-0.7 equiv. based on **1b**), **CAL** (9.0 μmol), r.t., 1 atom, 50 mL vessel.
yield and conversion estimated by ^{1}H NMR analysis in CD_3CN

Table S5a. Cartesian coordinates of ***ax*-PMD** (E_{opt} = –0.223697095824 hartree, E_{sp} = –543.331400930 hartree).

C	-2.01466	0.15694	-0.1287	C	1.70013	1.41122	0.08744
C	-2.72035	-0.81197	-1.07975	H	0.13055	1.00873	1.54253
C	-2.36757	-0.12144	1.3307	O	0.07002	-1.72269	1.00301
O	-2.57761	1.43743	-0.51883	H	1.83203	-0.79378	-1.71968
H	-2.43352	-0.63591	-2.12234	H	2.40814	-0.26713	1.27107
H	-3.80815	-0.66305	-1.03181	H	1.78669	1.80132	-0.94483
H	-2.51138	-1.85568	-0.82527	H	2.1934	2.16071	0.73346
H	-3.45373	-0.15593	1.47331	H	-0.27923	2.26205	0.36163
H	-1.97182	0.63373	2.01355	H	2.201	-1.99885	-0.48092
H	-1.95829	-1.09622	1.64367	C	3.89468	0.19595	-0.22906
H	-2.3468	2.13722	0.11373	H	3.98632	0.51675	-1.27267
C	0.2215	1.28074	0.47376	H	4.43032	-0.75518	-0.13289
C	-0.48521	0.23598	-0.40653	H	4.42459	0.93162	0.38583
C	0.22273	-1.13274	-0.29778	H	-0.38598	0.57376	-1.4728
C	1.717	-1.01614	-0.64284	H	-0.27428	-1.89477	-0.94275
C	2.43081	0.0582	0.20065	H	0.49647	-1.16774	1.6864

Table S5b. Cartesian coordinates of ***eq*-PMD** (E_{opt} = – 0.220493757472 hartree, E_{sp} = –543.329849602 hartree).

C	1.97531	-0.24489	-0.00411	C	-1.77003	-1.39089	-0.34597
C	2.80238	0.77935	-0.77604	H	-0.1725	-1.65521	1.10465
C	2.25594	-0.20531	1.50091	H	-0.10049	1.04601	1.41852
O	2.46696	-1.50357	-0.53659	H	-1.85776	1.30444	-1.12084
H	2.80769	0.54299	-1.84813	H	-2.39405	-0.33703	1.44963
H	3.84627	0.78384	-0.44761	H	-1.88272	-1.31154	-1.44447
H	2.39275	1.79407	-0.65838	H	-2.2668	-2.33617	-0.05967
H	3.28545	-0.5237	1.7079	H	0.19083	-2.30193	-0.50788
H	1.58564	-0.85254	2.07239	H	-2.22262	1.96458	0.47405
H	2.15273	0.81384	1.89267	O	0.40183	2.22402	-0.20489
H	2.22868	-2.25499	0.02919	C	-3.94558	-0.13495	-0.04458
C	-0.2838	-1.45107	0.02274	H	-0.09594	3.02745	0.02424
C	0.45647	-0.15316	-0.35275	H	-4.0803	-0.00037	-1.12378
C	-0.25141	1.04237	0.31708	H	-4.45437	0.69723	0.45367
C	-1.74639	1.10886	-0.03582	H	-4.47149	-1.0541	0.23649
C	-2.46484	-0.19968	0.34211	H	0.39039	-0.01427	-1.46234

Table S6a. Cartesian coordinates of **PMD** isomer 1 (E_{opt} = –0.225618046895 hartree, E_{sp} = –543.328587250 hartree).

C	1.92679	0.1001	0.0127	C	-0.30706	-1.03478	-0.57344
C	2.69302	-0.99734	-0.74075	H	-1.72615	-0.68246	-2.16676
C	2.72587	1.40629	0.02662	H	-2.21641	2.35402	0.21793
O	1.84242	-0.26258	1.40075	H	-0.33198	1.09036	1.27906
H	2.1732	-1.96141	-0.70259	H	0.59592	0.62121	-1.6285
H	3.67533	-1.15799	-0.27796	O	-0.30117	-1.64325	0.73413
H	2.858	-0.7382	-1.78902	H	-2.29059	-1.81438	-0.93167
H	3.74323	1.22999	0.3992	H	0.27038	2.32729	0.17933
H	2.27501	2.13373	0.714	H	-1.61037	1.97993	-1.39644
H	2.79645	1.85707	-0.96533	H	0.2208	-1.8135	-1.17557

H	1.3359	-1.11422	1.50365	H	-0.86498	-1.10993	1.35683
C	-1.7476	-0.85866	-1.07411	H	-3.43739	0.48512	-1.01942
C	-2.52754	0.28532	-0.39593	C	-3.01108	-0.10625	1.00573
C	-1.68854	1.57748	-0.36764	H	-3.55159	0.71585	1.48746
C	-0.28448	1.36916	0.20722	H	-2.17192	-0.37405	1.66712
C	0.48946	0.28984	-0.56717	H	-3.68586	-0.96889	0.97029

Table S6b. Cartesian coordinates of **PMD** isomer 2 (E_{opt} = –0.220544849934 hartree, E_{sp} = –543.325925236 hartree).

C	-1.83908	-0.27166	0.10115	C	0.34447	1.09036	0.09863
C	-2.69891	0.98776	0.07188	H	1.83549	1.5165	-1.42454
C	-2.59497	-1.45347	-0.5255	H	2.35884	-2.21319	-0.77193
O	-1.67897	-0.52791	1.51635	H	0.40281	-1.69433	0.66217
H	-2.1892	1.82594	0.58001	H	-0.57596	0.13476	-1.62766
H	-3.64014	0.8288	0.61176	H	2.29831	2.00819	0.2013
H	-2.92851	1.30824	-0.94577	H	-0.11936	-2.20423	-0.95467
H	-3.52501	-1.64057	0.02722	H	1.81998	-1.07073	-2.00628
H	-2.0209	-2.38428	-0.50746	O	-0.28074	2.35036	-0.21296
H	-2.86686	-1.25101	-1.56563	H	3.57515	0.00774	-0.72974
H	-1.14449	-1.32795	1.67246	C	2.87655	-0.43894	1.25943
C	1.80568	1.19442	-0.36646	H	1.95382	-0.5495	1.84032
C	2.59038	-0.11765	-0.21074	H	3.46123	0.35732	1.73352
C	1.82498	-1.25472	-0.91467	H	3.4433	-1.36993	1.36272
C	0.38817	-1.38639	-0.40467	H	0.25556	1.05788	1.21006
C	-0.42821	-0.08577	-0.54595	H	-0.33077	2.49711	-1.17369

Table S7a. Cartesian coordinates of **MCA-a** (E_{opt} = –0.246229259758 hartree, E_{sp} = –933.821983389 hartree).

C	-2.50907	2.52089	0.28857	H	-1.29239	-2.01694	1.85153
C	-2.82063	1.85451	1.64297	H	-1.94431	-3.56209	1.33272
C	-2.45969	0.36445	1.60698	C	1.3771	-0.44228	-0.91398
C	-3.24119	-0.37176	0.50761	C	2.60556	-0.7035	-0.01623
C	-3.04991	0.31336	-0.86464	C	3.92846	-0.42569	-0.74589
C	-3.29588	1.82534	-0.83883	C	5.10676	-0.73642	0.12692
O	-1.69028	0.18296	-1.34259	C	6.12464	0.09986	0.38065
C	-1.07145	-1.10453	-1.18985	C	6.22996	1.48723	-0.17341
O	-2.0193	-2.08489	-0.80532	C	7.26919	-0.30995	1.26024
C	-2.88803	-1.87672	0.35074	H	0.30807	-1.99241	0.20451
C	-2.8291	4.01816	0.3293	H	-0.17067	-0.32977	0.62787
H	-1.41786	2.38786	0.07088	H	2.59342	-1.75275	0.33627
H	-2.25408	2.35643	2.4488	H	2.549	-0.08021	0.89612
H	-3.88848	1.98588	1.89681	H	3.95195	0.63177	-1.08703
H	-2.66325	-0.09183	2.59247	H	3.99109	-1.03822	-1.66944
H	-1.36441	0.25526	1.42083	H	5.08892	-1.73636	0.5599
H	-4.37875	2.01898	-0.73428	H	6.46641	2.21777	0.61037
H	-2.99212	2.25212	-1.81602	H	5.29436	1.81409	-0.65236
C	0.1107	-0.99097	-0.22413	H	7.02246	1.54778	-0.93105
C	-2.21827	-2.52315	1.56176	H	7.22126	-1.36548	1.55499
C	-4.12581	-2.68392	-0.07081	H	7.28524	0.28398	2.18398
H	-2.24513	4.52886	1.10245	H	8.23284	-0.15794	0.75672
H	-3.88705	4.20594	0.54005	H	1.52199	-0.99393	-1.87655

H	-2.59484	4.50041	-0.62622	C	1.21542	1.04861	-1.22803
H	-4.32599	-0.29706	0.77283	H	1.24734	1.66171	-0.32193
H	-4.61741	-2.23971	-0.94367	H	0.2427	1.24445	-1.70741
H	-4.85501	-2.7651	0.73876	H	1.99836	1.40654	-1.90177
H	-3.83404	-3.70006	-0.37033	H	-3.69149	-0.18204	-1.63044
H	-2.88245	-2.5367	2.43135	H	-0.79139	-1.42691	-2.21811

Table S7b. Cartesian coordinates of **MCA-b** (E_{opt} = –0.251000109061 hartree, E_{sp} = –933.838138811 hartree).

C	-5.09638	1.64441	0.33006	H	-1.72621	-3.68489	-0.95105
C	-5.57499	0.38439	-0.42404	H	-3.2188	-2.9357	1.47449
C	-4.72145	-0.84701	-0.08389	H	-2.39661	-1.49154	2.06809
C	-3.24433	-0.56921	-0.38499	H	-1.45325	-2.88921	1.54215
C	-2.7841	0.66475	0.41949	C	2.05057	-0.15365	0.35603
C	-3.60064	1.90783	0.06264	C	3.29373	0.58278	-0.18522
O	-1.42156	0.9757	0.0336	C	4.53578	0.34831	0.6876
C	-0.49527	-0.09945	0.27821	C	5.71205	1.12431	0.17656
O	-0.93785	-1.28389	-0.39592	C	6.91712	0.60783	-0.10768
C	-2.28956	-1.75743	-0.11035	C	7.26659	-0.84099	0.04008
C	-5.93305	2.86272	-0.07313	C	8.0378	1.47275	-0.60517
H	-5.23189	1.46968	1.42639	H	0.79331	1.42645	-0.52116
H	-6.63427	0.18393	-0.18006	H	0.80306	-0.07852	-1.44552
H	-5.54481	0.5692	-1.51497	H	3.08539	1.66863	-0.24633
H	-5.07136	-1.71802	-0.66897	H	3.50443	0.26148	-1.22317
H	-4.8557	-1.11945	0.97996	H	4.76283	-0.73864	0.73042
H	-2.79274	0.47076	1.51455	H	4.3297	0.65221	1.73495
H	-3.42775	2.1768	-0.99767	H	5.52027	2.18944	0.04753
H	-3.24156	2.77637	0.64449	H	7.74302	-1.23434	-0.86684
H	-0.43603	-0.28948	1.36622	H	6.38036	-1.46284	0.23828
C	0.79936	0.32189	-0.40922	H	7.96562	-0.99344	0.87288
C	-2.34695	-2.29117	1.32238	H	7.78692	2.54063	-0.5938
C	-2.44789	-2.87984	-1.13718	H	8.3025	1.2137	-1.63926
H	-6.99649	2.70535	0.13617	H	8.94106	1.34568	0.00557
H	-5.83907	3.0861	-1.14184	H	1.92893	0.1107	1.43599
H	-5.62111	3.75934	0.47399	C	2.19813	-1.67502	0.23786
H	-3.13552	-0.31624	-1.47224	H	2.52926	-1.97128	-0.76385
H	-2.24289	-2.51082	-2.15114	H	1.2374	-2.18062	0.40907
H	-3.45354	-3.30717	-1.12194	H	2.92177	-2.06775	0.95693

Table S7c. Cartesian coordinates of **MCA-c** (E_{opt} = –0.246943802154 hartree, E_{sp} = –933.819645240 hartree).

C	-3.80471	2.25168	0.03237	H	-3.56411	-3.47724	-0.24913
C	-4.60427	1.2223	-0.79078	H	-2.36007	-3.13513	0.99856
C	-4.84337	-0.07859	-0.01238	H	-1.84143	-3.49211	-0.6478
C	-3.56997	-0.70928	0.57726	C	1.83132	-0.83171	0.42228
C	-2.76209	0.3732	1.3337	C	3.08447	0.05781	0.55738
C	-2.47909	1.6216	0.4936	C	4.38427	-0.76268	0.57234
O	-1.54801	-0.20567	1.84183	C	5.57375	0.13106	0.75882
C	-0.67453	-0.84871	0.89347	C	6.34777	0.6066	-0.22876
O	-1.30668	-0.95303	-0.37738	C	6.12757	0.29935	-1.6783
C	-2.6667	-1.48896	-0.42347	C	7.52363	1.49122	0.06233

C	-3.54669	3.51966	-0.7882	H	0.69797	0.65736	1.57475
H	-4.40332	2.53149	0.93428	H	0.3949	0.71003	-0.1666
H	-5.57284	1.65409	-1.10068	H	3.02096	0.6557	1.48733
H	-4.05263	0.99638	-1.73043	H	3.11973	0.79501	-0.26676
H	-5.35616	-0.81164	-0.66379	H	4.46943	-1.35495	-0.3604
H	-5.54816	0.1239	0.82002	H	4.35084	-1.50968	1.39142
H	-3.27099	0.64093	2.29021	H	5.77454	0.3919	1.79712
H	-1.85458	1.34578	-0.38132	H	6.61144	1.02686	-2.34053
H	-1.87817	2.33886	1.07954	H	5.05894	0.29139	-1.93435
H	-0.49005	-1.84728	1.33596	H	6.531	-0.69118	-1.93041
C	0.56847	0.01518	0.6798	H	7.71299	1.60677	1.13675
C	-2.60948	-2.97867	-0.05663	H	7.36788	2.49791	-0.34862
C	-2.98437	-1.31078	-1.90724	H	8.44202	1.09084	-0.38707
H	-4.48338	3.98138	-1.11745	H	1.8848	-1.6339	1.20009
H	-2.95014	3.31113	-1.68321	C	1.75554	-1.49428	-0.95817
H	-3.00102	4.26741	-0.20208	H	1.89922	-0.76672	-1.76409
H	-3.07896	-0.24231	-2.16451	H	0.76903	-1.95105	-1.12252
H	-3.90922	-1.81789	-2.19226	H	2.51375	-2.27346	-1.07362
H	-2.16751	-1.71056	-2.52271	H	-3.90817	-1.44656	1.35222

Table S7d. Cartesian coordinates of **MCA-d** (E_{opt} = –0.244183908982 hartree, E_{sp} = –933.828872934 hartree).

C	-3.39117	2.49385	0.41741	H	-2.40783	-3.5564	1.53844
C	-4.63359	1.58255	0.48365	C	1.51816	-0.63547	-0.3138
C	-4.27476	0.13959	0.87789	C	2.96825	-0.44088	-0.80461
C	-2.84366	-0.24065	0.47274	C	3.74143	0.56603	0.06038
C	-2.43374	0.48391	-0.82464	C	5.12933	0.78179	-0.46289
C	-2.42513	2.00945	-0.68363	C	6.25481	0.67822	0.25972
O	-1.0479	0.15379	-1.11228	C	6.28946	0.32029	1.71367
C	-0.7957	-1.25053	-1.26208	C	7.60141	0.92638	-0.35454
O	-1.22942	-1.95334	-0.09064	H	1.02852	-1.12743	-2.38715
C	-2.61817	-1.76713	0.31813	H	0.94818	-2.515	-1.27695
C	-3.79687	3.9513	0.1757	H	2.96238	-0.09248	-1.85527
H	-2.86134	2.43505	1.40097	H	3.50198	-1.40969	-0.81105
H	-5.36338	1.99349	1.205	H	3.76138	0.21439	1.11437
H	-5.14596	1.58787	-0.49692	H	3.20657	1.53861	0.08068
H	-5.00749	-0.55152	0.42028	H	5.17028	1.04682	-1.51923
H	-4.38369	0.01142	1.97192	H	7.0159	-0.4768	1.91523
H	-2.67198	2.471	-1.65689	H	5.3111	-0.03092	2.07604
H	-1.39064	2.34358	-0.45619	H	6.5717	1.18762	2.32468
C	0.71752	-1.43472	-1.36919	H	7.53552	1.29865	-1.38423
C	-2.64817	-2.49298	1.66424	H	8.19516	0.00283	-0.38127
C	-3.53462	-2.44161	-0.70574	H	8.1719	1.6662	0.22174
H	-4.41857	4.33348	0.9925	H	1.03253	0.3697	-0.20965
H	-4.36649	4.07007	-0.75234	C	1.47139	-1.34282	1.04394
H	-2.91728	4.60121	0.10437	H	2.03701	-2.27883	1.03951
H	-3.67778	-1.83607	-1.60617	H	0.42928	-1.59296	1.30216
H	-4.52587	-2.64472	-0.28763	H	1.87254	-0.7109	1.84026
H	-3.10907	-3.4038	-1.01925	H	-2.13629	0.10554	1.27203
H	-3.62599	-2.41498	2.14617	H	-3.04982	0.15394	-1.68829
H	-1.89217	-2.08429	2.34723	H	-1.35885	-1.62774	-2.13993

Table S8. Cartesian coordinates of **1a** (R = -OCH_3; E_{opt} = –8819.08385474 hartree, E_{sp} = –8819.58378719 hartree).

N	7.04754	-2.22227	-0.42899	C	4.47480	0.63499	4.89473
C	6.03963	-2.80509	0.24588	C	3.23221	2.63684	5.61545
H	5.26684	-2.14779	0.62686	C	5.65020	-1.05777	-3.18224
C	5.96308	-4.17696	0.45721	H	4.91640	-1.33949	-2.43623
C	7.00153	-4.96110	-0.04831	C	-4.65793	5.12731	-2.06145
H	6.98287	-6.03589	0.10066	C	3.39571	1.24051	5.62420
C	8.04751	-4.36115	-0.74017	C	6.38094	0.66017	2.06051
H	8.86574	-4.94748	-1.14222	H	5.49317	0.99703	1.53833
C	8.03834	-2.99021	-0.92037	C	3.13316	0.36823	-5.16425
H	8.82560	-2.48064	-1.46268	H	4.06540	0.81911	-4.84305
N	6.77783	-0.47748	-2.72971	C	3.18354	5.12435	-4.00095
N	7.06697	1.85759	-0.99192	C	3.04179	-1.05600	-5.28075
C	8.16635	2.57418	-0.69054	C	4.38131	4.49003	-3.52845
H	9.01668	2.02271	-0.30845	C	-3.49844	5.90704	-2.39159
C	8.20605	3.94564	-0.86087	C	1.33998	-6.27763	3.28964
H	9.11175	4.48895	-0.61710	C	0.16043	-5.53048	3.28078
C	7.07999	4.59998	-1.34458	H	0.13355	-4.60232	2.71849
H	7.08454	5.67613	-1.48451	C	-0.98959	-5.93902	3.94809
C	5.92917	3.87131	-1.65483	C	-0.95672	-7.12717	4.70350
C	5.97814	2.49414	-1.46505	C	0.22021	-7.88767	4.73549
H	5.11870	1.87617	-1.69701	C	1.34658	-7.47965	4.00456
C	-4.92122	3.94450	-2.77380	C	1.67315	-3.02711	-5.85054
N	-7.31513	-1.38137	-0.58869	N	7.33721	0.08140	1.31069
C	-8.41827	-2.14773	-0.49399	C	-2.64014	5.49334	-3.42608
H	-9.15896	-1.84620	0.23669	C	4.84577	-4.89402	2.58079
C	-8.59384	-3.26502	-1.28926	C	5.38417	1.45473	4.20693
H	-9.49856	-3.85398	-1.19168	C	3.73132	4.45939	-6.28110
C	-7.60184	-3.61150	-2.19909	H	3.50016	4.44678	-7.34093
H	-7.71330	-4.48678	-2.83091	C	-4.08950	3.54104	-3.83219
C	-6.45023	-2.82884	-2.30340	C	-4.33721	2.35445	-4.59503
C	-6.35968	-1.71489	-1.47604	H	-5.17617	1.71753	-4.33744
H	-5.49250	-1.06663	-1.52111	C	-3.54641	2.01094	-5.64822
C	4.58007	-0.79327	4.89957	H	-3.76086	1.10888	-6.21121
H	5.37390	-1.27329	4.33787	C	-2.44406	2.82875	-6.01542
C	4.16916	3.45028	4.95047	H	-1.84816	2.56775	-6.88424
C	5.26762	2.85441	4.24622	C	-2.14545	3.94059	-5.28994
C	6.22049	3.71811	3.61543	H	-1.30244	4.56168	-5.56614
H	7.07364	3.28102	3.10771	C	-2.94294	4.33820	-4.16908
C	6.10057	5.07329	3.67956	N	-6.82261	1.86731	1.89217
H	6.85060	5.70974	3.22073	C	-5.67983	2.02147	2.58758
C	5.00235	5.66468	4.36191	H	-4.91604	1.26708	2.43939
H	4.92041	6.74571	4.41037	C	-5.46012	3.08459	3.45597
C	4.06801	4.87724	4.96288	C	-6.49569	4.00870	3.60992
H	3.23128	5.33058	5.47967	H	-6.36672	4.84884	4.28461
N	-7.16292	1.50588	-0.99223	C	-7.67788	3.85005	2.89663
H	-8.49568	4.55421	2.99813	C	-3.60424	1.55317	8.00788
C	-7.80794	2.77275	2.03959	H	-3.47070	1.11829	8.99258
H	-8.70623	2.61340	1.45557	C	-2.58582	2.23528	7.41567
C	-6.12006	3.12585	-2.43325	H	-1.63844	2.34030	7.92825
C	2.54943	-5.79110	2.56287	C	4.92445	3.84306	-5.81477

C	6.49540	0.82846	3.43615	H	5.59646	3.37459	-6.52671
C	4.79592	-4.75160	1.18513	C	0.73310	-0.73706	-6.06206
C	-3.25453	7.10862	-1.65377	H	-0.19372	-1.15716	-6.43041
H	-2.38282	7.69926	-1.90642	C	2.52308	-5.67071	1.15916
C	2.89241	5.07167	-5.40081	C	6.40666	-0.91881	-5.43353
H	1.98555	5.54712	-5.75475	H	6.26038	-1.09274	-6.49468
C	5.23332	3.85360	-4.48805	C	6.04012	-4.67958	4.69312
H	6.15957	3.39958	-4.15227	H	6.93744	-4.42387	5.24751
N	-6.97770	-1.00558	2.26636	C	1.89426	6.63274	-0.81508
C	-7.96252	-1.01687	3.18435	H	1.02816	7.16254	-1.19138
H	-8.78848	-0.33531	3.02036	C	0.85885	0.61068	-5.92507
C	-7.91846	-1.85739	4.28148	H	0.02720	1.26268	-6.16867
H	-8.73281	-1.84564	4.99676	C	-1.38287	6.24642	-3.71439
C	-6.82397	-2.69821	4.44785	C	-0.15111	5.73668	-3.30232
H	-6.76199	-3.36091	5.30502	H	-0.12516	4.79325	-2.76546
C	-5.79111	-2.68800	3.50857	C	1.04799	6.38701	-3.57113
C	-5.92282	-1.82578	2.42617	C	1.02765	7.61273	-4.26049
H	-5.15651	-1.78545	1.66094	C	-0.20360	8.15963	-4.65154
C	1.80405	-1.62913	-5.73178	C	-1.39678	7.46439	-4.39828
C	-4.57243	-3.53633	3.63935	C	-7.28728	3.16666	-3.19944
C	-4.55805	-4.82965	3.09310	H	-7.33617	3.82046	-4.06419
C	-5.69718	-5.38762	2.42768	C	-8.37399	2.37438	-2.85068
H	-6.60832	-4.80259	2.36229	H	-9.29187	2.39220	-3.42695
C	-5.66781	-6.64486	1.90552	C	3.58986	-5.02104	-0.96681
H	-6.54933	-7.05775	1.42562	H	4.44489	-4.63724	-1.51236
C	-4.48413	-7.42790	1.99125	C	2.48334	-5.40824	-1.65810
H	-4.47011	-8.42647	1.56691	H	2.46207	-5.32279	-2.73951
C	-3.37729	-6.92810	2.60728	C	-5.25422	6.76025	-0.35374
H	-2.47519	-7.52459	2.65933	C	-3.11343	3.92881	3.54004
C	-3.36915	-5.62514	3.19894	C	-3.25780	4.50076	2.23526
C	3.71474	-1.56488	5.61250	H	-4.20360	4.40780	1.71339
H	3.82306	-2.64421	5.60658	C	-2.23893	5.17962	1.64131
C	2.07873	1.17383	-5.46573	H	-2.37703	5.60966	0.65512
H	2.17623	2.25023	-5.37422	C	-1.00409	5.35162	2.32102
C	2.76080	-3.86276	-5.53066	H	-0.20051	5.89870	1.84012
C	2.66584	-5.28814	-5.61900	C	-0.82988	4.83788	3.56883
H	1.72913	-5.72411	-5.94140	H	0.10699	4.99122	4.08839
C	3.72003	-6.09442	-5.31593	C	-1.86176	4.09283	4.22623
H	3.62577	-7.17178	-5.39970	C	-4.10035	7.52637	-0.67241
C	4.95457	-5.52815	-4.89743	H	-3.90566	8.45006	-0.13809
H	5.79318	-6.17988	-4.67427	C	3.86423	5.22142	-1.25425
C	5.08876	-4.17758	-4.78634	C	4.68935	4.52979	-2.15750
H	6.03979	-3.75737	-4.47672	C	-4.39589	-4.12746	-2.88639
C	4.00408	-3.29389	-5.09250	C	-4.45848	-4.83821	-1.64461
C	-1.68120	3.54163	5.51061	H	-5.24762	-4.60984	-0.93669
C	3.70337	-5.41237	3.27802	C	-3.55674	-5.81070	-1.33885
C	-2.23619	-5.12108	3.86798	H	-3.63164	-6.33918	-0.39449
C	7.67259	0.38163	4.03962	C	-2.52470	-6.14644	-2.25628
H	7.80318	0.49916	5.11054	H	-1.84038	-6.95499	-2.01966
C	7.57321	-0.32291	-4.96916	C	-2.40745	-5.47266	-3.43232
H	8.36163	-0.02257	-5.64970	H	-1.62512	-5.73268	-4.13433
C	-8.27887	1.55077	-1.74435	C	-3.32178	-4.43048	-3.79051
H	-9.09709	0.91006	-1.43920	H	-6.41847	5.04375	-0.78298

C	2.33617	5.79372	-3.10070	H	-5.93284	7.11274	0.41642
C	-5.26286	-2.49071	-4.48167	O	-2.59766	8.00775	-4.74126
C	-4.16985	-2.77672	-5.36554	O	2.22774	8.20037	-4.44609
C	-4.10757	-2.07887	-6.61281	O	-1.34669	5.01227	7.91792
H	-3.27624	-2.28226	-7.27645	O	3.39021	4.04894	7.98742
C	-5.07155	-1.19135	-6.98328	O	-2.07946	-7.42216	5.39023
H	-5.01845	-0.69602	-7.94740	O	2.49432	-8.21117	4.05693
C	-6.16393	-0.92173	-6.11405	O	-3.36158	-5.12628	-7.38290
H	-6.93801	-0.22825	-6.42719	O	1.33981	-4.45128	-8.28672
C	-6.24884	-1.54144	-4.90414	C	-3.81341	-5.30670	-8.72647
H	-7.09802	-1.34055	-4.25978	H	-3.46804	-6.25382	-9.14155
C	2.23181	6.71795	0.50018	H	-4.90198	-5.30709	-8.66180
H	1.64176	7.32785	1.17691	H	-3.48007	-4.48924	-9.36966
C	8.46546	-0.35011	1.90589	C	1.33352	-3.79096	-9.55609
H	9.20621	-0.81417	1.26611	H	0.49950	-4.13137	-10.17234
C	6.00645	-4.53530	3.33971	H	1.27984	-2.70561	-9.41729
H	6.88326	-4.16543	2.81915	H	2.27843	-4.04972	-10.03347
C	2.52068	0.38847	6.37182	C	-2.85464	8.14779	-6.14223
H	1.73582	0.84781	6.95962	H	-2.86907	7.16406	-6.62422
C	4.90391	-5.17600	5.38837	H	-3.84069	8.60486	-6.22203
H	4.94193	-5.28682	6.46700	H	-2.10806	8.78648	-6.61744
C	2.03516	3.24929	6.26429	C	2.48210	9.05338	-5.56428
C	2.15370	3.99383	7.45215	H	1.99703	8.67654	-6.46752
C	1.00727	4.57677	8.01055	H	2.14595	10.07306	-5.37510
C	-0.23388	4.45314	7.36601	H	3.56501	9.03924	-5.69218
C	-0.36583	3.69160	6.20027	C	-1.38529	6.44219	7.95110
C	0.78280	3.09144	5.68150	H	-1.36164	6.84470	6.93249
H	0.70260	2.50257	4.77301	H	-2.33122	6.70571	8.42385
C	-5.34130	-3.15288	-3.24487	H	-0.55371	6.84476	8.53216
C	3.36855	6.02163	0.99209	C	3.82093	5.15293	8.78585
H	3.61463	6.06765	2.04742	H	4.91002	5.13179	8.73475
C	-6.11254	2.27823	-1.33024	H	3.44893	6.09937	8.38720
H	-5.23812	2.21220	-0.69356	H	3.49799	5.04813	9.82188
C	5.40936	-1.29360	-4.53080	C	-2.46189	-8.77051	5.66880
C	4.15383	5.30043	0.14614	H	-3.54034	-8.73350	5.82684
H	5.02022	4.78410	0.54442	H	-2.23309	-9.42946	4.82824
C	-3.45179	-3.02245	4.31020	H	-1.96982	-9.14878	6.56509
C	-2.27119	-3.83205	4.43322	C	2.44469	-9.50768	3.45306
C	-1.15799	-3.29078	5.15375	H	1.69140	-10.13579	3.93192
H	-0.27640	-3.90625	5.27779	H	2.23374	-9.41799	2.38189
C	-1.19335	-2.03903	5.68555	H	3.43453	-9.94158	3.59297
H	-0.33353	-1.65095	6.22101	O	-1.08430	-5.50385	-9.08285
C	-2.35656	-1.23489	5.55123	O	-0.24825	9.37046	-5.28448
H	-2.38218	-0.24498	5.99437	O	0.27552	-9.03596	5.47546
C	-3.44638	-1.71378	4.89184	O	1.09325	5.28199	9.17866
H	-4.33605	-1.09721	4.82645	C	-0.68574	10.45718	-4.46396
C	-5.51967	5.60259	-1.02071	H	-0.67632	11.34215	-5.10030
C	-4.15554	3.22588	4.16486	H	-1.69895	10.27969	-4.09368
C	2.68209	5.87638	-1.74075	H	0.00077	10.60400	-3.62285
C	3.65793	-5.13609	0.45899	C	1.01610	-8.92765	6.69389
C	7.72594	-0.10897	-3.61158	H	0.97055	-9.90903	7.16619
H	8.61300	0.36058	-3.20410	H	2.05778	-8.66402	6.49211
C	2.67870	-0.96277	6.37640	H	0.56113	-8.18194	7.35507

H	2.02448	-1.58703	6.97665	C	0.57587	4.60326	10.32629
C	3.77861	-5.51971	4.70335	H	0.71047	5.28260	11.16807
H	2.91816	-5.89616	5.24042	H	-0.48679	4.38056	10.19844
C	-3.19987	-3.73014	-5.00327	H	1.13373	3.67841	10.50967
C	4.12079	-1.89984	-4.97376	C	-0.52453	-6.81954	-9.05173
C	8.66548	-0.20876	3.26661	H	-0.66479	-7.23369	-10.05024
H	9.58984	-0.56017	3.71043	H	0.54206	-6.78138	-8.81483
C	1.36535	-5.94953	-0.96924	H	-1.04947	-7.44328	-8.31989
H	0.49121	-6.26250	-1.53021	Pt	-7.06911	0.25193	0.64070
C	1.39012	-6.08128	0.38491	Pt	7.05698	-0.18406	-0.71183
H	0.54189	-6.51219	0.90108	C	-0.99687	-4.81732	-7.90379
C	0.36965	-3.61018	-6.28505	C	0.23537	-4.27179	-7.50979
C	-0.76735	-3.48286	-5.48536	C	-2.73218	2.83029	6.12213
H	-0.68484	-2.96647	-4.53398	C	-3.98880	2.67455	5.44527
C	-2.01231	-3.96031	-5.87833	C	-5.03447	1.95135	6.10455
C	-2.13365	-4.64527	-7.10116	H	-5.99472	1.84577	5.61099
H	-5.66161	0.88051	7.83020	C	-4.85206	1.41374	7.34229

Table S9a. Cartesian coordinates of **1a•CAL** (R = -OCH_3; E_{opt} = 1.66758347018 hartree).

Pt	-7.25451	0.49312	0.31063	C	2.39431	1.77661	5.93314
Pt	7.24574	-0.598	-0.26865	H	1.57466	2.32572	6.40495
N	-7.01869	2.08944	1.48581	C	2.56195	0.44385	6.16117
C	-5.78731	2.39632	2.0012	H	1.8863	-0.11029	6.81278
H	-4.94346	1.73341	1.73934	C	3.65893	-0.25721	5.5583
C	-5.60309	3.51674	2.8325	H	3.76163	-1.32699	5.75884
C	-6.71612	4.33184	3.12172	C	4.55277	0.40267	4.76712
H	-6.591	5.21403	3.76589	H	5.39728	-0.128	4.33329
C	-7.9653	4.00947	2.58858	C	4.85706	-5.51872	-3.27183
H	-8.84197	4.6339	2.80498	C	4.49963	-4.27363	2.6516
C	-8.10046	2.88017	1.77176	C	-3.37288	-4.60788	-3.23762
H	-9.06684	2.59244	1.33352	C	-3.31449	4.49514	2.61033
N	-7.21671	1.68059	-1.29387	C	-8.39143	1.96519	-1.93934
N	-7.49867	-1.09816	-0.8732	H	-9.31058	1.51583	-1.53636
C	-8.72564	-1.69873	-0.97473	C	-1.98927	-1.24297	6.15904
H	-9.54895	-1.27054	-0.38549	H	-1.24248	-0.77138	6.7963
C	-8.91035	-2.81618	-1.79935	C	-2.4943	3.51247	6.70119
H	-9.90188	-3.28271	-1.86921	H	-1.52504	3.77654	7.13247
C	-7.83359	-3.32952	-2.52494	C	2.6724	4.57462	-4.13207
H	-7.95966	-4.20782	-3.17406	C	-4.72433	3.60364	-3.35843
C	-6.57094	-2.71357	-2.42213	C	-8.54231	-1.58074	3.79538
C	-6.43729	-1.58908	-1.58606	H	-9.47433	-1.6315	4.37365
H	-5.47134	-1.0718	-1.48548	C	-1.39322	5.92504	1.08774
C	4.32391	-3.19954	-4.02992	H	-0.69619	6.50302	0.48267
N	7.41046	1.08962	-1.31833	C	-1.12361	5.62044	2.38838
C	8.61093	1.72479	-1.48649	H	-0.19566	5.95407	2.8589
H	9.49851	1.25676	-1.03781	C	-1.00037	5.2313	-4.79922
C	8.68777	2.92544	-2.20556	C	0.18952	4.67454	-4.32341
H	9.6594	3.41898	-2.33587	H	0.13897	3.8147	-3.64833
C	7.53019	3.4882	-2.7471	C	1.44048	5.19304	-4.6665
H	7.57336	4.43427	-3.30386	C	1.50303	6.32686	-5.52744
C	6.29446	2.83425	-2.57479	C	0.31961	6.83669	-6.1114
C	6.27311	1.62203	-1.86332	C	-0.93563	6.31356	-5.72406

H	5.34043	1.0534	-1.7152	C	3.10923	-4.60297	4.65282
C	-4.11429	-1.03532	4.99766	C	4.32366	-4.14452	4.04751
H	-4.99407	-0.45727	4.72636	C	5.34222	-3.57141	4.89636
C	-3.767	-5.11914	3.74492	H	6.27113	-3.23291	4.44287
C	-4.93781	-4.36984	3.39963	C	5.16157	-3.47041	6.24504
C	-5.98608	-5.02682	2.65558	H	5.94035	-3.06146	6.89326
H	-6.88297	-4.46515	2.40795	C	3.93506	-3.90488	6.8494
C	-5.87731	-6.33927	2.29667	H	3.82272	-3.80271	7.93056
H	-6.67973	-6.8489	1.75846	C	2.94774	-4.44431	6.08061
C	-4.69996	-7.08836	2.62656	H	2.01003	-4.77846	6.53296
H	-4.64838	-8.13665	2.32676	C	-5.94336	-1.653	-6.50909
C	-3.68471	-6.49799	3.31875	H	-6.66122	-1.02978	-7.04714
H	-2.79006	-7.06797	3.58331	C	-1.87583	2.57808	-5.5934
N	7.05326	-1.62195	-1.96556	H	-0.98303	3.03188	-6.02986
C	-4.02353	-2.40921	4.5617	C	-2.06227	4.87362	3.19464
C	-2.74189	-4.51262	4.50779	C	-7.21251	3.33846	-3.54235
C	-6.03833	2.21207	-1.74693	H	-7.20391	3.99018	-4.4277
H	-5.11816	1.96611	-1.1931	C	-4.71782	2.56601	6.89148
C	3.96757	-4.55843	-3.88255	H	-5.46271	2.08967	7.5333
C	-2.86815	-3.16996	4.93195	C	-2.50696	-5.55738	-2.57684
C	-6.22068	-1.40987	2.29411	H	-1.68347	-5.98846	-3.15106
H	-5.31522	-1.31759	1.67312	C	-2.23172	1.30185	-5.91345
C	-4.2301	1.46136	-4.54062	H	-1.6356	0.70088	-6.59895
H	-5.15191	1.03307	-4.15547	C	0.5078	-4.57761	-5.49567
C	-4.05318	-3.32495	-5.21393	C	-0.67035	-4.21713	-4.83967
C	-3.89038	2.81888	-4.18347	H	-0.61168	-3.56096	-3.96606
C	-5.20627	-2.85009	-4.50851	C	-1.9249	-4.65712	-5.27335
C	2.69237	-5.00922	-4.35333	C	-1.99903	-5.54883	-6.38153
C	-0.48878	4.93165	5.14318	C	-0.81703	-5.95005	-7.04655
C	0.68548	4.28989	4.76841	C	0.42858	-5.41077	-6.64925
H	0.64664	3.52571	3.98718	C	6.80028	-3.03011	-4.36057
C	1.92967	4.58549	5.35979	H	6.69443	-3.58222	-5.30471
C	1.96899	5.52963	6.40692	C	8.05521	-2.6053	-3.92093
C	0.78945	6.24242	6.77395	H	8.95489	-2.81853	-4.51184
C	-0.43022	5.96557	6.12866	C	-3.55834	4.8297	1.2264
C	-2.29591	4.69225	-4.33235	H	-4.5051	4.54076	0.77687
N	-7.32117	-0.68514	1.92212	C	-2.63194	5.51141	0.49383
C	1.81644	-4.10292	-4.99495	H	-2.81678	5.76816	-0.55263
C	-4.01034	3.50969	4.74928	C	4.5023	-6.83082	-3.1518
C	-5.0396	-3.0173	3.79411	C	3.49015	-4.84643	1.84836
C	-4.78071	-2.11031	-7.2132	C	3.62967	-4.97091	0.41605
H	-4.6546	-1.81513	-8.25624	H	4.53733	-4.60109	-0.05529
C	3.44535	-2.2862	-4.65082	C	2.65549	-5.55581	-0.33821
C	3.78821	-0.89326	-4.823	H	2.75925	-5.6504	-1.42279
H	4.75173	-0.54404	-4.46003	C	1.47062	-6.07827	0.27916
C	2.9405	-0.03242	-5.45593	H	0.72659	-6.56316	-0.35207
H	3.20426	1.01903	-5.59823	C	1.30271	-5.98032	1.62765
C	1.68349	-0.49485	-5.96942	H	0.41392	-6.39507	2.11096
H	1.04208	0.21569	-6.48881	C	2.29435	-5.34135	2.463
C	1.32442	-1.80178	-5.82529	C	3.21825	-7.28144	-3.60582
H	0.38165	-2.17001	-6.23715	H	2.96774	-8.33717	-3.49056
C	2.18257	-2.74625	-5.14695	C	-4.50075	-4.09997	-2.5167
N	7.09766	-2.29161	0.76692	C	-5.3998	-3.22223	-3.16035

C	5.89952	-2.67045	1.31146	C	4.14109	4.04299	-2.22504
H	5.03686	-1.99775	1.1398	C	4.38373	4.1152	-0.80246
C	5.78257	-3.86638	2.04427	H	5.2946	3.67505	-0.404
C	6.92378	-4.67856	2.19708	C	3.5153	4.75997	0.02907
H	6.85151	-5.6191	2.75894	H	3.70338	4.82495	1.10413
C	8.13808	-4.2815	1.63383	C	2.34343	5.39663	-0.49575
H	9.03482	-4.90407	1.74461	H	1.68887	5.92919	0.1929
C	8.20836	-3.07896	0.92188	C	2.08556	5.35687	-1.83406
H	9.14309	-2.72575	0.46242	H	1.21635	5.87513	-2.24728
C	5.65725	-2.74934	-3.5855	C	2.95565	4.65283	-2.74753
C	-1.78625	4.54997	4.54207	H	5.83084	-5.18232	-2.92382
C	-6.24093	-2.2445	3.4273	H	5.18182	-7.56475	-2.71273
C	-4.27655	3.82797	3.39817	O	1.62828	-5.59148	-7.25022
C	2.34855	-6.40246	-4.17893	O	-0.88923	-6.80314	-8.13979
H	1.3713	-6.73882	-4.53221	O	-3.27026	-5.9102	-6.6768
C	-3.87097	-2.90882	-6.58615	O	3.03694	5.91537	7.15511
H	-2.98772	-3.26742	-7.1202	O	0.76679	7.09914	7.85602
C	-6.14105	-1.99676	-5.20319	O	-1.63046	6.5534	6.35525
H	-7.02659	-1.6526	-4.67508	O	1.7665	-7.72722	5.64007
N	7.47703	0.42765	1.42685	O	-0.57608	-8.29675	6.74903
C	8.69759	0.49055	2.04492	O	-2.80076	-6.5099	6.34395
H	9.53082	-0.04902	1.57178	O	2.76175	6.79881	-5.68706
C	8.86087	1.21499	3.23244	O	0.38497	7.90608	-6.99464
H	9.84561	1.25264	3.71592	O	-2.15892	6.73086	-6.12637
C	7.77086	1.88511	3.79298	C	4.25145	5.12203	7.18593
H	7.8849	2.45797	4.72271	C	1.67167	8.23826	7.77977
C	6.51644	1.82362	3.15695	C	-1.73257	7.62708	7.33838
C	6.40209	1.07864	1.96931	C	2.96791	-7.82392	4.83241
H	5.44394	0.98697	1.43272	C	0.31476	-8.41757	7.89531
C	-2.67485	3.37802	-4.69339	C	-3.01354	-7.56515	7.32916
C	5.33879	2.5238	3.70815	C	1.70533	-6.22347	-8.56302
C	5.1681	3.90241	3.45545	C	-0.73142	-8.21039	-7.7855
C	6.11035	4.65547	2.66135	C	-3.56356	-6.66842	-7.88731
H	6.98559	4.14481	2.26597	C	2.98854	8.08253	-6.34291
C	5.92854	5.98747	2.42815	C	0.54049	7.49628	-8.38701
H	6.64878	6.56553	1.84404	C	-2.30329	7.94336	-6.9238
C	4.77894	6.66815	2.95241	H	4.72928	5.50794	8.1013
H	4.66493	7.7336	2.74203	H	4.85735	5.34927	6.30474
C	3.86669	5.99087	3.7044	H	4.03092	4.05699	7.28761
H	2.99079	6.50266	4.11228	H	1.4702	8.83103	6.88682
C	4.03094	4.5847	3.99751	H	2.71222	7.8948	7.8178
C	-3.13321	-0.47482	5.76245	H	1.41004	8.78311	8.69712
H	-3.20145	0.56298	6.09863	H	-1.15345	8.49188	7.00433
C	-3.42789	0.73027	-5.36681	H	-1.39135	7.27705	8.32079
H	-3.68405	-0.29691	-5.63991	H	-2.81208	7.82448	7.32443
C	-3.13235	5.47074	-3.49852	H	3.67561	-7.0502	5.14208
C	-2.78733	6.82283	-3.1208	H	2.73789	-7.78652	3.76521
H	-1.83838	7.23124	-3.47618	H	3.33093	-8.82505	5.1164
C	-3.62435	7.58363	-2.36012	H	-0.04323	-9.35154	8.34944
H	-3.37302	8.61142	-2.0924	H	0.19261	-7.5687	8.56907
C	-4.87329	7.04483	-1.90527	H	1.35153	-8.52521	7.55539
H	-5.52783	7.68749	-1.31208	H	-2.77086	-8.54389	6.89667
C	-5.22654	5.76314	-2.21333	H	-2.41262	-7.36695	8.22045

H	-6.1751	5.35976	-1.86811	H	-4.08807	-7.45703	7.52561
C	-4.37126	4.92949	-3.02482	H	2.67259	-5.85025	-8.92547
C	2.11418	-5.22139	3.86001	H	0.87887	-5.90964	-9.21078
C	-2.74322	3.85633	5.31942	H	1.7164	-7.30864	-8.4279
C	3.11362	3.8875	4.81722	H	0.25392	-8.38517	-7.34681
C	-7.42596	-2.32351	4.18479	H	-0.82461	-8.69939	-8.76463
H	-7.46153	-2.96925	5.07404	H	-1.52886	-8.52527	-7.10739
C	-8.40721	2.79504	-3.06723	H	-2.98031	-6.30484	-8.7409
H	-9.35828	3.01257	-3.57072	H	-4.63711	-6.47021	-8.00969
C	8.16467	-1.90062	-2.71614	H	-3.37053	-7.72745	-7.69282
H	9.13275	-1.54689	-2.33272	H	2.22506	8.81787	-6.0646
C	-3.14296	-4.19685	-4.57203	H	3.01288	7.92263	-7.42446
C	4.75775	3.30888	-4.48498	H	3.9782	8.35277	-5.95061
C	3.55016	3.88386	-4.99888	H	1.47881	6.9531	-8.52228
C	3.27272	3.74742	-6.41136	H	0.56236	8.46518	-8.90411
H	2.34733	4.18001	-6.79785	H	-0.31506	6.89551	-8.70631
C	4.13965	3.10857	-7.2464	H	-2.10692	7.69894	-7.97178
H	3.93728	3.01548	-8.31486	H	-3.36201	8.18462	-6.7574
C	5.35745	2.55167	-6.73152	H	-1.64114	8.7405	-6.56721
H	6.03812	2.06123	-7.43161	C	-2.58264	0.77059	0.75699
C	5.65036	2.6399	-5.40181	C	-3.64303	-0.06575	0.09442
H	6.57564	2.21722	-5.0169	C	-2.51724	0.66988	2.24689
C	-2.71894	-5.92935	-1.28268	C	-1.75823	1.53066	0.0204
H	-2.07405	-6.65389	-0.78718	C	-0.65017	2.38416	0.5572
C	-8.47365	-0.76494	2.65877	C	0.72865	1.83077	0.16009
H	-9.33121	-0.16489	2.32272	C	1.08954	0.55116	0.94237
C	-4.98512	2.84695	5.58261	C	2.09653	-0.3289	0.16367
H	-5.95235	2.59274	5.15674	C	3.39283	0.35673	-0.0962
C	-1.86734	-2.54168	5.76335	C	1.62607	0.87535	2.33761
H	-1.01432	-3.14454	6.08452	H	-4.65269	0.20647	0.44158
C	-3.44386	2.89622	7.46067	H	-3.47266	-1.13177	0.31817
H	-3.26477	2.6579	8.51086	H	-3.61703	0.02607	-1.00108
C	-1.52143	-5.27853	4.84475	H	-2.74744	1.63339	2.72967
C	-1.57672	-6.35485	5.78282	H	-1.50732	0.38926	2.58535
C	-0.43302	-7.14643	5.99923	H	-3.19455	-0.08235	2.66953
C	0.78566	-6.83781	5.32684	H	-1.84903	1.56246	-1.06688
C	0.86773	-5.70566	4.48895	H	-0.71301	2.50368	1.65749
C	-0.30605	-4.96594	4.24609	H	-0.76265	3.4167	0.15656
H	-0.25603	-4.11613	3.55924	H	1.49053	2.61832	0.32503
C	5.03791	3.39716	-3.10397	H	0.73668	1.62888	-0.92867
C	-3.82554	-5.38615	-0.55034	H	1.64616	-0.64139	-0.80811
H	-3.96581	-5.70147	0.48706	H	3.37251	1.45111	-0.26376
C	5.81627	-2.03658	-2.38295	H	2.51728	1.51166	2.31795
H	4.95775	-1.7841	-1.73392	H	1.88466	-0.02976	2.90106
C	-6.00372	3.04808	-2.87897	H	0.87459	1.41043	2.93348
C	-4.68623	-4.51013	-1.1445	O	4.46269	-0.24221	-0.13713
H	-5.54031	-4.125	-0.59337	H	0.15609	-0.06279	1.05619
C	4.41265	1.81592	4.50398	H	2.25212	-1.28853	0.70571
C	3.29913	2.5103	5.07758				

Table S9b. Cartesian coordinates of **1a•(CAL)**$_2$ (R = -OCH_3; E_{opt} = 1.56335818372 hartree).

Pt	7.13252	-0.32398	-0.70811	C	2.77431	1.9206	5.83163

Pt	-7.12055	0.13664	0.74664	H	2.1873	1.53016	6.66316
N	7.2945	-1.84576	0.57489	C	3.90066	-2.68426	6.57377
C	6.24878	-2.2062	1.38104	H	3.0303	-2.91235	7.19548
H	5.30895	-1.64134	1.28254	C	-3.19167	-5.15111	-3.65997
C	6.37007	-3.2626	2.30598	C	4.17036	-3.70034	-3.77678
C	7.59291	-3.95634	2.38279	C	8.80481	2.02925	2.41655
H	7.70465	-4.78343	3.09894	H	9.80295	2.13814	2.86109
C	8.65026	-3.58271	1.55056	C	2.36796	-6.48619	1.91898
H	9.61052	-4.11292	1.59774	H	1.66228	-7.24376	1.58051
C	8.48494	-2.52152	0.65258	C	2.19633	-5.83452	3.10429
H	9.2942	-2.19674	-0.0165	H	1.34195	-6.05913	3.74757
N	6.89462	-1.61586	-2.20967	C	0.40511	-5.56189	-4.76327
N	7.03667	1.1981	-1.99904	C	-0.75667	-5.09899	-4.1419
C	8.20563	1.81189	-2.36523	H	-0.67514	-4.2555	-3.43727
H	9.13628	1.43148	-1.91948	C	-2.00521	-5.68046	-4.36663
C	8.20305	2.88114	-3.26967	C	-2.10012	-6.78118	-5.26503
H	9.15003	3.36069	-3.55023	C	-0.95527	-7.20969	-5.97781
C	6.99502	3.32717	-3.80678	C	0.30494	-6.62926	-5.70122
H	6.971	4.1651	-4.51844	C	-2.59202	4.79351	4.7067
C	5.79065	2.69604	-3.434	C	-3.84068	4.20142	4.32915
C	5.84416	1.62622	-2.52044	C	-4.67478	3.6365	5.36426
H	4.93107	1.10259	-2.20181	H	-5.62904	3.19516	5.08503
C	-5.09045	2.79338	-3.53551	C	-4.29182	3.66628	6.67368
N	-7.38735	-1.61733	-0.16307	H	-4.93099	3.26039	7.46181
C	-8.60402	-2.24466	-0.11116	C	-3.02958	4.23574	7.04998
H	-9.40388	-1.74719	0.45554	H	-2.75269	4.23492	8.10637
C	-8.80551	-3.46996	-0.75868	C	-2.21173	4.77204	6.10139
H	-9.78702	-3.95852	-0.70706	H	-1.24887	5.20955	6.37903
C	-7.75665	-4.06116	-1.46655	C	4.54558	1.41267	-7.30514
H	-7.89746	-5.02219	-1.98056	H	5.17163	0.75024	-7.90724
C	-6.50622	-3.4163	-1.52133	C	1.00008	-2.85321	-5.61995
C	-6.35295	-2.18609	-0.85615	H	0.08629	-3.36045	-5.93831
H	-5.39876	-1.63358	-0.86792	C	3.14731	-4.84875	3.56205
C	4.61931	1.56379	4.28935	C	6.59633	-3.41896	-4.31837
H	5.42327	0.9436	3.90039	H	6.47226	-4.12926	-5.14839
C	4.02777	5.52545	2.76383	C	6.02494	-1.5748	6.20917
C	5.13652	4.74565	2.29933	H	6.80375	-0.92505	6.61425
C	6.05325	5.34014	1.35596	C	1.74843	5.56151	-3.12509
H	6.90149	4.75488	1.01073	H	0.85905	5.98201	-3.60051
C	5.88823	6.62638	0.92949	C	1.2337	-1.55852	-5.97603
H	6.59676	7.09113	0.23996	H	0.5184	-0.99596	-6.57403
C	4.77412	7.40637	1.38425	C	-1.65468	4.41673	-5.57517
H	4.67595	8.43299	1.02618	C	-0.39354	4.12705	-5.05065
C	3.87547	6.87114	2.25861	H	-0.32577	3.53428	-4.1311
H	3.02986	7.46464	2.61652	C	0.78492	4.5505	-5.67046
N	-7.28772	1.06555	-1.00762	C	0.69585	5.38665	-6.81989
C	4.44509	2.89448	3.75585	C	-0.57191	5.74522	-7.33521
C	3.14061	4.98555	3.72335	C	-1.74517	5.20454	-6.75746
C	5.67042	-2.18036	-2.45432	C	-7.55921	2.398	-3.44342
H	4.83174	-1.88887	-1.78979	H	-7.65998	2.92295	-4.40359
C	-4.84795	4.18422	-3.48848	C	-8.68188	1.8855	-2.79108
C	3.36605	3.6939	4.25282	H	-9.68168	1.99878	-3.22886
C	6.30829	1.70705	1.25193	C	4.43499	-5.27861	1.49576

H	5.33582	1.55101	0.76317	H	5.31202	-5.08335	0.88398
C	3.40193	-1.59634	-4.88098	C	3.51274	-6.20477	1.10284
H	4.33953	-1.11343	-4.61852	H	3.62788	-6.75572	0.16661
C	2.88074	3.18091	-5.84054	C	-5.47148	6.42949	-2.74469
C	3.19159	-2.96942	-4.48389	C	-3.39937	4.77198	1.9833
C	4.11583	2.71769	-5.2822	C	-3.7583	4.76936	0.58423
C	-3.71051	4.72711	-4.16988	H	-4.68864	4.29289	0.28336
C	1.78436	-4.44734	5.62153	C	-2.96543	5.37216	-0.34767
C	0.57233	-3.85642	5.28627	H	-3.23943	5.37683	-1.40676
H	0.52909	-3.19241	4.41225	C	-1.75508	6.0351	0.04397
C	-0.59898	-4.06706	6.04236	H	-1.16119	6.52901	-0.72504
C	-0.5049	-4.82848	7.22629	C	-1.37883	6.0532	1.35355
C	0.72098	-5.46389	7.57984	H	-0.46804	6.57128	1.66672
C	1.85707	-5.29803	6.76663	C	-2.17377	5.40436	2.37137
C	1.70382	-4.93467	-4.43405	C	-4.32849	6.97426	-3.41908
N	7.37125	0.97129	0.79502	H	-4.16796	8.053	-3.38114
C	-2.85688	3.875	-4.90568	C	3.71093	4.06607	-3.27101
C	5.11904	-3.0044	4.44629	C	4.50467	3.1506	-3.99514
C	5.31125	3.42768	2.77786	C	-4.43253	-4.8016	-1.5599
C	3.29916	1.85606	-7.85813	C	-4.54	-5.04456	-0.14003
H	3.02053	1.51177	-8.85551	H	-5.37273	-4.60429	0.40313
C	-4.24109	1.93696	-4.26844	C	-3.64199	-5.84324	0.50584
C	-4.47066	0.5127	-4.3425	H	-3.72742	-6.0396	1.57742
H	-5.32611	0.09283	-3.81849	C	-2.56578	-6.46066	-0.21348
C	-3.65348	-0.29474	-5.07774	H	-1.88657	-7.11791	0.32903
H	-3.83108	-1.37155	-5.14118	C	-2.425	-6.24435	-1.55196
C	-2.54798	0.25732	-5.80567	H	-1.62351	-6.73001	-2.11496
H	-1.93637	-0.41323	-6.4077	C	-3.3405	-5.39081	-2.27418
C	-2.29881	1.5966	-5.75886	H	-6.58341	4.68067	-2.25516
H	-1.47876	2.03183	-6.33589	H	-6.13779	7.11791	-2.21986
C	-3.12119	2.48656	-4.97159	O	-3.01631	5.3432	-7.20207
N	-6.91878	1.90341	1.64549	O	-0.66106	6.55297	-8.46115
C	-5.68463	2.40018	1.97183	O	1.90973	5.74114	-7.30357
H	-4.81081	1.77548	1.71518	O	-1.48514	-5.10029	8.13145
C	-5.55039	3.65327	2.59854	O	0.87342	-6.12159	8.78478
C	-6.71484	4.39724	2.87674	O	3.08861	-5.83241	6.95414
H	-6.63046	5.3802	3.35891	O	-1.33801	8.10503	5.2011
C	-7.96603	3.87916	2.53911	O	1.10072	8.90457	5.87079
H	-8.88136	4.4462	2.75101	O	3.35266	7.19889	5.30595
C	-8.04979	2.62486	1.9241	O	-3.34721	-7.30395	-5.32513
H	-9.01375	2.17638	1.64128	O	-1.05322	-8.25826	-6.88277
C	-6.28377	2.24252	-2.8647	O	1.50196	-6.9738	-6.23202
C	2.99277	-4.19337	4.80551	C	-2.68051	-4.28137	8.19834
C	6.45829	2.63029	2.30323	C	0.0103	-7.27808	8.98228
C	5.23135	-3.61005	3.17461	C	3.32379	-6.71767	8.09002
C	-3.48113	6.15267	-4.10041	C	-2.6634	8.03207	4.61593
H	-2.61308	6.55884	-4.62475	C	0.38468	9.10424	7.12377
C	2.49877	2.70214	-7.14952	C	3.63904	8.35709	6.14647
H	1.55268	3.05141	-7.57008	C	-3.2767	5.91912	-8.51723
C	4.93192	1.81536	-6.0594	C	-0.79218	7.97191	-8.14469
H	5.87878	1.47822	-5.64567	C	2.02351	6.44395	-8.5763
N	-6.99526	-0.77736	2.51358	C	-3.58068	-8.57466	-6.00357
C	-8.09385	-0.90425	3.32077	C	-1.36108	-7.8221	-8.24126

H	-9.04229	-0.48786	2.95304	C	1.6279	-8.16851	-7.05902
C	-7.99418	-1.53945	4.5653	H	-3.03424	-4.5086	9.21707
H	-8.88348	-1.63152	5.20186	H	-3.39831	-4.62773	7.45011
C	-6.76489	-2.04973	4.98813	H	-2.44506	-3.21907	8.10174
H	-6.67269	-2.54881	5.96169	H	0.1614	-8.0113	8.18926
C	-5.63422	-1.92196	4.1574	H	-1.03606	-6.95813	9.05026
C	-5.78632	-1.27394	2.91858	H	0.36574	-7.65023	9.95265
H	-4.94014	-1.13512	2.22263	H	2.73938	-7.63372	7.97236
C	1.95532	-3.60158	-4.83511	H	3.07512	-6.20668	9.02871
C	-4.3207	-2.45363	4.56865	H	4.4016	-6.90217	7.9952
C	-4.08342	-3.84496	4.50396	H	-3.2377	7.2449	5.11175
C	-5.07217	-4.7639	3.99198	H	-2.61512	7.90503	3.53206
H	-6.02806	-4.3697	3.65471	H	-3.05956	9.02816	4.87256
C	-4.8307	-6.10662	3.94929	H	0.74657	10.0974	7.42304
H	-5.58695	-6.80768	3.58744	H	0.65953	8.33899	7.85064
C	-3.57114	-6.63367	4.38743	H	-0.69584	9.12556	6.9396
H	-3.41099	-7.71218	4.33505	H	3.27163	9.27394	5.66869
C	-2.61166	-5.79638	4.87354	H	3.191	8.21878	7.13385
H	-1.64956	-6.19014	5.21329	H	4.73567	8.32364	6.1849
C	-2.83412	-4.37133	4.96536	H	-4.27715	5.52041	-8.73299
C	3.81445	1.0965	5.28649	H	-2.53781	5.58793	-9.25567
H	3.9446	0.09039	5.69672	H	-3.2877	7.0088	-8.42525
C	2.45559	-0.91561	-5.58889	H	-1.71348	8.15444	-7.58631
H	2.61217	0.12364	-5.88936	H	-0.83699	8.42128	-9.14597
C	2.67189	-5.6479	-3.69019	H	0.08272	8.32245	-7.59117
C	2.44153	-7.00404	-3.24611	H	1.33278	6.03705	-9.32351
H	1.48018	-7.46641	-3.48251	H	3.07231	6.24755	-8.83712
C	3.39605	-7.69965	-2.56563	H	1.84493	7.50952	-8.40532
H	3.22922	-8.72981	-2.24684	H	-2.76298	-9.28251	-5.82669
C	4.65966	-7.08856	-2.27235	H	-3.71684	-8.38024	-7.07106
H	5.41118	-7.68216	-1.74685	H	-4.51528	-8.9036	-5.52901
C	4.91122	-5.8019	-2.65124	H	-2.33375	-7.3251	-8.27097
H	5.87051	-5.34341	-2.42521	H	-1.38511	-8.77757	-8.78272
C	3.92669	-5.03274	-3.37537	H	-0.5707	-7.17003	-8.62164
C	-1.77819	5.41074	3.72873	H	1.32083	-7.92192	-8.07944
C	3.98347	-3.29742	5.26803	H	2.70766	-8.36003	-6.99718
C	-1.86252	-3.50557	5.52339	H	1.04136	-9.00146	-6.65477
C	7.73124	2.78406	2.89005	C	2.16356	-1.7135	-0.49155
H	7.86616	3.49927	3.71454	C	3.05945	-0.50522	-0.52892
C	7.83793	-2.83784	-4.05769	C	2.53463	-2.74818	0.519
H	8.70999	-3.08194	-4.67823	C	1.11362	-1.822	-1.31882
C	-8.52818	1.22377	-1.56724	C	0.18015	-2.9894	-1.38471
H	-9.38513	0.8101	-1.01634	C	-1.24552	-2.64207	-0.93186
C	2.08445	4.10488	-5.12328	C	-1.33449	-2.27628	0.56156
C	-5.25654	-3.80788	-3.64744	C	-2.76996	-1.82582	0.9175
C	-4.14565	-4.37333	-4.35402	C	-3.18438	-0.53192	0.3041
C	-4.04776	-4.14448	-5.77792	C	-0.93229	-3.44638	1.46207
H	-3.19986	-4.58071	-6.31079	H	4.10474	-0.79041	-0.72324
C	-4.99308	-3.42552	-6.44635	H	3.0107	0.02935	0.43711
H	-4.93263	-3.26913	-7.52467	H	2.75052	0.2166	-1.29958
C	-6.11035	-2.87004	-5.73777	H	3.11875	-2.35619	1.3594
H	-6.85783	-2.31352	-6.30783	H	3.1145	-3.56326	0.05964
C	-6.23095	-3.04322	-4.38973	H	1.64113	-3.22557	0.97069

H	-7.08364	-2.62787	-3.85783	H	0.8814	-1.02413	-2.03824
C	2.14108	5.98364	-1.88984	H	0.56089	-3.85339	-0.80166
H	1.58225	6.74364	-1.34566	H	0.14152	-3.3581	-2.44161
C	8.6072	1.1263	1.36371	H	-1.90705	-3.5099	-1.1405
H	9.42878	0.51559	0.96254	H	-1.63172	-1.81185	-1.55222
C	6.12838	-2.10792	4.95617	H	-2.86746	-1.7623	2.02983
H	6.9968	-1.893	4.33996	H	-2.42691	0.06741	-0.25505
C	2.55752	3.16864	5.32908	H	-1.09914	-3.23152	2.5246
H	1.78281	3.81403	5.75086	H	0.14279	-3.67603	1.34786
C	4.88778	-1.86284	7.03293	H	-1.48493	-4.36333	1.22214
H	4.84325	-1.426	8.03183	O	-4.33304	-0.11096	0.39892
C	1.94859	5.74868	4.15518	H	-0.6267	-1.42412	0.76857
C	2.07208	6.92259	4.95927	H	-3.48494	-2.63948	0.64606
C	0.92297	7.67527	5.26879	C	0.02157	2.03146	-1.90338
C	-0.36018	7.23734	4.82635	C	0.45096	1.27031	-3.12142
C	-0.49094	6.02245	4.12054	C	-1.45478	2.05132	-1.64909
C	0.68018	5.32124	3.77892	C	0.92352	2.6211	-1.10258
H	0.58697	4.39536	3.18599	C	0.61056	3.35752	0.16561
C	-5.37044	-4.00842	-2.25459	C	0.98682	2.54044	1.41316
C	3.32604	5.44405	-1.28984	C	0.08722	1.2996	1.58676
H	3.60875	5.79493	-0.29397	C	0.86883	0.09522	2.14022
C	-6.17718	1.55936	-1.63889	C	1.04195	0.05588	3.63225
H	-5.20383	1.3905	-1.14227	C	-1.14993	1.61607	2.43353
C	5.48775	-3.0925	-3.51011	H	-0.32367	1.24326	-3.90054
C	4.08567	4.52635	-1.95505	H	0.66886	0.21501	-2.86299
H	4.99833	4.14417	-1.50542	H	1.35706	1.68608	-3.58299
C	-3.33385	-1.57777	5.07207	H	-2.02275	2.25719	-2.56897
C	-2.09712	-2.11151	5.5606	H	-1.73762	2.82641	-0.91874
C	-1.12158	-1.19925	6.11158	H	-1.80112	1.07855	-1.26162
H	-0.17972	-1.61452	6.48733	H	1.98523	2.59888	-1.34966
C	-1.35302	0.14404	6.15264	H	-0.46341	3.64174	0.21124
H	-0.62256	0.82937	6.58043	H	1.16651	4.31985	0.17313
C	-2.58337	0.67656	5.64846	H	0.92251	3.19551	2.31266
H	-2.73871	1.75744	5.69833	H	2.05037	2.24495	1.35015
C	-3.53745	-0.15004	5.12903	H	1.87791	0.04472	1.66402
H	-4.47849	0.25931	4.77096	H	1.05007	1.02298	4.15928
C	-5.71448	5.08686	-2.7673	H	-1.76663	0.72844	2.60363
C	-4.22791	4.1956	2.97026	H	-1.7707	2.38265	1.95208
C	2.50217	4.56665	-3.85335	H	-0.88942	2.01269	3.42298
C	4.28655	-4.56548	2.74299	O	1.18053	-0.99648	4.22192
C	7.9703	-1.93488	-2.99562	H	-0.26379	0.99189	0.56517
H	8.92979	-1.45326	-2.75931	H	0.3764	-0.85115	1.79709

Table S10a. Cartesian coordinates of **1a•*eq*-PMD** (R = -OCH_3; E_{opt} = .53111329886 hartree, E_{sp} = – 9363.65675418 hartree).

Pt	-7.14976	0.27571	0.21283	H	1.17977	5.75238	-3.84588
Pt	7.33413	-0.18202	-0.20211	C	2.36542	5.88255	-2.06977
N	-7.21307	1.05693	-1.61856	H	1.74795	6.64313	-1.59199
C	-6.04416	1.47835	-2.18885	C	3.53367	5.41677	-1.38075
H	-5.11656	1.32617	-1.59292	H	3.7498	5.82892	-0.39129
C	-6.02402	2.06641	-3.46686	C	4.35591	4.49154	-1.95517
C	-7.23887	2.19164	-4.16689	H	5.25348	4.16247	-1.43827

H	-7.24657	2.63868	-5.17024	C	5.79041	-1.79678	5.54102
C	-8.42587	1.7484	-3.57653	C	5.14972	3.66588	2.94731
H	-9.37972	1.84018	-4.11064	C	-2.45018	-1.84464	5.45502
C	-8.39805	1.18737	-2.29501	C	-3.82127	1.63221	-4.56784
H	-9.30873	0.83461	-1.79164	C	-8.47035	-2.21255	-0.54406
N	-7.27003	-1.55371	-0.5639	H	-9.32428	-1.68807	-0.09023
N	-7.0994	-0.50145	2.05117	C	-1.81553	6.29423	-0.26697
C	-8.23664	-0.48425	2.81648	H	-1.15776	6.78406	-0.98353
H	-9.13756	-0.04213	2.36747	C	-3.07556	5.84915	-4.75107
C	-8.23533	-1.00803	4.11428	H	-2.15227	6.21161	-5.20993
H	-9.15459	-0.98477	4.71279	C	2.19942	-5.11431	-3.85313
C	-7.0619	-1.55506	4.63842	C	-5.0429	-4.07955	-2.2513
H	-7.04181	-1.96735	5.65641	C	-8.2003	4.13999	1.64367
C	-5.89384	-1.57697	3.85256	H	-9.12751	4.7208	1.72442
C	-5.94547	-1.03984	2.55275	C	-1.94817	-0.18516	-5.68431
H	-5.04708	-1.04	1.89745	H	-1.26731	-0.91096	-6.1256
C	4.99895	-3.21912	3.64774	C	-1.72048	1.15478	-5.78143
N	7.22989	-1.59261	-1.61024	H	-0.84806	1.53297	-6.319
C	8.35928	-1.95534	-2.29402	C	-1.50929	-5.87654	-3.94919
H	9.29587	-1.44212	-2.03328	C	-0.27116	-5.32132	-3.62321
C	8.30925	-2.94237	-3.28719	H	-0.22592	-4.49709	-2.89326
H	9.22496	-3.22009	-3.82588	C	0.9197	-5.77042	-4.19899
C	7.09569	-3.56319	-3.58422	C	0.87135	-6.84422	-5.13295
H	7.03617	-4.33839	-4.36199	C	-0.35682	-7.49573	-5.39643
C	5.93096	-3.19284	-2.88036	C	-1.55379	-6.9898	-4.83874
C	6.03208	-2.19716	-1.89053	C	3.77952	5.70474	2.85042
H	5.15287	-1.87644	-1.31297	C	4.94863	4.97588	2.45826
C	-3.92069	5.14415	0.12195	C	5.89495	5.61409	1.57421
H	-4.87061	4.75356	-0.23718	H	6.79155	5.07075	1.28655
C	-2.96872	4.92763	4.29439	C	5.69014	6.88599	1.12291
C	-4.21199	4.39516	3.82328	H	6.41644	7.38212	0.47443
C	-5.12816	3.82544	4.78356	C	4.50797	7.60848	1.49396
H	-6.0763	3.42836	4.42885	H	4.37454	8.62181	1.10964
C	-4.83269	3.81009	6.11549	C	3.58776	7.03565	2.32023
H	-5.53376	3.40439	6.84815	H	2.68392	7.57991	2.60892
C	-3.58499	4.33625	6.58964	C	-5.31093	-5.80239	3.97284
H	-3.38444	4.31219	7.66219	H	-6.08964	-6.48868	3.6332
C	-2.68818	4.86722	5.7115	C	-2.17821	-6.25773	-1.14791
H	-1.74098	5.27942	6.06806	H	-1.31673	-6.75648	-1.5988
N	7.36053	-1.59829	1.20434	C	-2.63069	2.11579	-5.2009
C	-3.6207	5.04647	1.53194	C	-7.47449	-4.12137	-1.64277
C	-2.06692	5.52304	3.38216	H	-7.5487	-5.13142	-2.06896
C	-6.16765	-2.14994	-1.11705	C	-5.20472	6.24562	-3.66196
H	-5.22841	-1.56583	-1.1156	H	-5.92193	6.97788	-3.28404
C	4.80932	-2.66395	4.93294	C	-1.47664	-0.94233	6.02723
C	-2.383	5.58965	2.00736	H	-0.60619	-1.37395	6.52599
C	-5.88714	2.63092	1.40653	C	-2.45311	-6.4108	0.17853
H	-4.98716	1.98895	1.27264	H	-1.82211	-7.02302	0.82234
C	-4.45046	-5.04201	-0.02652	C	1.39996	-4.12566	5.84118
H	-5.35421	-4.61271	0.40261	C	0.19601	-3.58815	5.37637
C	-3.25921	-4.10209	4.94504	H	0.20307	-2.98427	4.46398
C	-4.18757	-4.84731	-1.43336	C	-1.01611	-3.80839	6.03297
C	-4.46926	-3.54845	4.41379	C	-1.0196	-4.56535	7.24137

C	3.61475	-2.9658	5.66273	C	0.1895	-5.09824	7.74497
C	-1.14179	4.00077	-5.90619	C	1.38838	-4.93476	7.01173
C	0.09577	3.7748	-5.31666	C	7.43227	-3.5913	3.16027
H	0.14423	3.19619	-4.3897	H	7.45348	-4.37557	3.93098
C	1.2975	4.26098	-5.87067	C	8.58265	-3.26635	2.43997
C	1.22604	5.065	-7.02661	H	9.52833	-3.79009	2.63293
C	-0.02399	5.2741	-7.67913	C	-4.02731	0.20412	-4.48917
C	-1.20077	4.71946	-7.1411	H	-4.93307	-0.16826	-4.01605
C	-2.74666	-5.29824	-3.38104	C	-3.12088	-0.66814	-5.01476
N	-7.08074	2.11286	0.98805	H	-3.27436	-1.74799	-4.95356
C	2.655	-3.84663	5.11134	C	5.59642	-1.28209	6.79042
C	-4.51346	3.94225	-4.11146	C	4.22293	3.07687	3.83539
C	-4.51486	4.44911	2.44469	C	4.4013	1.73985	4.35023
C	-4.08902	-6.35916	4.47835	H	5.2719	1.16907	4.03777
H	-3.9811	-7.44468	4.49696	C	3.50972	1.2006	5.23129
C	4.05065	-4.10799	3.097	H	3.64302	0.18781	5.6221
C	4.23357	-4.71275	1.79849	C	2.37811	1.95877	5.67924
H	5.1227	-4.46337	1.22568	H	1.70349	1.50629	6.40559
C	3.32577	-5.60369	1.30448	C	2.17546	3.22361	5.21378
H	3.46601	-6.06946	0.32551	H	1.3276	3.81854	5.56546
C	2.16636	-5.96298	2.06801	C	3.07694	3.82467	4.25757
H	1.4827	-6.70536	1.65806	C	4.39273	-1.56659	7.51584
C	1.95236	-5.40073	3.29132	H	4.27145	-1.13668	8.5114
H	1.08371	-5.68349	3.89192	C	-3.63639	-1.28737	4.87753
C	2.87513	-4.43697	3.84549	C	-4.63805	-2.14734	4.37949
N	7.43707	1.2287	1.2076	C	3.68556	-3.15107	-3.96916
C	6.31489	1.92552	1.57276	C	3.91461	-1.81013	-4.45372
H	5.37241	1.67656	1.06307	H	4.84647	-1.31267	-4.19924
C	6.36577	2.92424	2.56387	C	2.99577	-1.17976	-5.24131
C	7.60332	3.19787	3.18095	H	3.16873	-0.16573	-5.61174
H	7.66421	3.97239	3.95818	C	1.7819	-1.84295	-5.61777
C	8.73904	2.48228	2.79944	H	1.08584	-1.31971	-6.27236
H	9.71031	2.68373	3.27066	C	1.53049	-3.10893	-5.17923
C	8.63827	1.49817	1.80777	H	0.62116	-3.63327	-5.48401
H	9.50958	0.91217	1.48147	C	2.46118	-3.80525	-4.32086
C	6.229	-2.90577	2.89777	H	6.70634	-1.58037	4.99753
C	-2.3819	3.50563	-5.26744	H	6.34876	-0.64904	7.26634
C	-5.80846	3.91884	1.9691	O	2.59967	-5.47262	7.29189
C	-4.75677	2.55174	-4.04808	O	0.18577	-5.85231	8.91099
C	3.43914	-2.37288	6.96873	O	-2.25646	-4.68799	7.77745
H	2.52278	-2.60128	7.51818	O	2.24403	5.68851	-7.68043
C	-3.10339	-5.53957	4.94079	O	-0.14743	6.13478	-8.75145
H	-2.17457	-5.95721	5.33575	O	-2.45552	4.81306	-7.64376
C	-5.48819	-4.45076	3.92869	O	2.69261	7.57927	5.63493
H	-6.41486	-4.03209	3.54311	O	0.37432	8.79091	6.09438
N	7.33822	1.23114	-1.61327	O	-1.99488	7.86101	4.73998
C	8.5267	1.81513	-1.96449	O	2.07829	-7.12045	-5.681
H	9.43165	1.47298	-1.4417	O	-0.39817	-8.5724	-6.27201
C	8.574	2.8088	-2.95015	O	-2.81321	-7.4365	-5.05183
H	9.5358	3.26616	-3.21796	C	3.51947	5.90904	-7.02495
C	7.39682	3.20902	-3.58408	C	0.6348	5.79068	-9.93101
H	7.41494	3.98738	-4.35968	C	-2.67862	5.52237	-8.89935
C	6.17292	2.60833	-3.22664	C	3.90769	6.82494	5.87879

C	6.17594	1.61459	-2.22917	C	1.22857	9.92836	5.78085
H	5.24573	1.11706	-1.91788	C	-2.14473	9.10591	5.48664
C	-3.02474	-5.45821	-2.00349	C	2.72395	-6.51499	8.30531
C	4.91618	3.01014	-3.88634	C	0.45589	-5.06414	10.10946
C	4.60258	2.48729	-5.16057	C	-2.49084	-5.58514	8.90294
C	5.46692	1.54665	-5.83315	C	2.17827	-8.02855	-6.81895
H	6.39162	1.2455	-5.34736	C	-0.21303	-9.86125	-5.61211
C	5.15014	1.05767	-7.06761	C	-3.07971	-8.44454	-6.07185
H	5.81168	0.36204	-7.58971	H	3.93842	6.7165	-7.64738
C	3.93205	1.45024	-7.71534	H	4.12712	5.00341	-7.10063
H	3.70873	1.03481	-8.70015	H	3.38759	6.2475	-5.99476
C	3.08888	2.33297	-7.10919	H	0.38099	4.79016	-10.283
H	2.15946	2.63941	-7.59752	H	1.70493	5.88997	-9.71427
C	3.39745	2.89929	-5.81567	H	0.3096	6.56514	-10.63903
C	-3.05062	5.73988	-0.74264	H	-2.18353	4.99288	-9.71734
H	-3.27787	5.81994	-1.80903	H	-2.31994	6.55642	-8.82127
C	-3.61344	-5.79075	0.74876	H	-3.77225	5.47164	-8.97878
H	-3.81382	-5.94372	1.81313	H	4.54902	6.87832	4.995
C	-3.6304	-4.56358	-4.20432	H	3.69004	5.79917	6.18432
C	-3.40808	-4.42306	-5.62618	H	4.34427	7.39418	6.71562
H	-2.51591	-4.88424	-6.05596	H	0.94831	10.6341	6.57469
C	-4.29354	-3.75778	-6.42028	H	0.99657	10.32249	4.79074
H	-4.13831	-3.67104	-7.49726	H	2.28307	9.64264	5.87186
C	-5.46831	-3.16322	-5.84973	H	-1.79353	8.97576	6.51793
H	-6.16349	-2.64921	-6.51764	H	-1.60095	9.90857	4.98196
C	-5.70313	-3.24587	-4.50819	H	-3.23164	9.25161	5.43775
H	-6.59694	-2.79821	-4.07959	H	3.65849	-7.00384	7.99875
C	-4.79846	-3.95778	-3.63651	H	1.87445	-7.2069	8.27843
C	2.85836	5.12998	3.75775	H	2.81961	-6.03914	9.28527
C	-3.30412	4.42182	-4.7116	H	1.45342	-4.62139	10.05778
C	2.55276	3.85669	-5.20557	H	0.39876	-5.82781	10.89693
C	-6.98663	4.67937	2.08079	H	-0.31181	-4.29768	10.24385
H	-6.94587	5.689	2.51176	H	-1.93986	-6.52566	8.78971
C	-8.59129	-3.5002	-1.08168	H	-3.57687	-5.73577	8.83451
H	-9.56209	-4.01158	-1.05922	H	-2.21444	-5.06799	9.8263
C	8.52958	-2.26393	1.46318	H	1.34862	-7.88726	-7.52105
H	9.41378	-1.98051	0.87455	H	2.21309	-9.05548	-6.44454
C	-2.26644	-3.24697	5.47571	H	3.13882	-7.72289	-7.2549
C	4.38502	-5.14381	-2.71543	H	0.77227	-9.91128	-5.14262
C	3.1453	-5.78262	-3.04249	H	-0.28781	-10.55813	-6.458
C	2.9079	-7.11917	-2.5465	H	-1.00897	-10.03549	-4.88344
H	1.95841	-7.59845	-2.79616	H	-2.86842	-9.43211	-5.6516
C	3.84235	-7.77553	-1.80221	H	-4.15573	-8.2997	-6.24014
H	3.67277	-8.79196	-1.44287	H	-2.49532	-8.26178	-6.98068
C	5.08938	-7.1397	-1.49017	C	-2.86298	0.76946	0.0845
H	5.82358	-7.70081	-0.90702	C	-2.52251	1.2243	1.50131
C	5.3452	-5.86875	-1.91737	C	-2.78842	1.91906	-0.91371
H	6.29197	-5.39259	-1.67558	O	-4.28568	0.39476	0.08155
C	-1.6432	0.40906	5.97208	H	-2.77036	0.47015	2.26003
H	-0.91484	1.08974	6.40988	H	-3.06479	2.14196	1.77433
C	-8.23137	2.85087	1.10242	H	-1.45519	1.45235	1.60825
H	-9.16374	2.38734	0.7508	H	-3.55025	2.68371	-0.70635
C	-5.45063	4.90589	-3.58301	H	-2.931	1.58698	-1.9514

H	-6.37263	4.5466	-3.13192	H	-1.82116	2.43591	-0.8664
C	-1.50002	6.227	1.05695	H	-4.39568	-0.51595	0.50905
H	-0.57666	6.67187	1.43441	C	-0.58297	-0.1252	-0.64619
C	-3.98947	6.72816	-4.25194	C	-2.05944	-0.47692	-0.38621
H	-3.82701	7.80647	-4.30099	C	-2.12825	-1.64478	0.62384
C	-0.78473	6.07429	3.87692	C	-1.323	-2.86532	0.17885
C	-0.77185	7.28866	4.63223	C	0.14691	-2.49158	-0.09484
C	0.43335	7.73623	5.20543	C	0.2112	-1.34575	-1.12347
C	1.63972	7.0093	4.98632	H	-0.12477	0.29096	0.27202
C	1.64433	5.87391	4.15057	H	-1.85662	-1.32018	1.6546
C	0.41398	5.41712	3.6335	H	-1.77178	-3.31789	-0.7293
H	0.40814	4.51084	3.02163	H	0.60717	-2.14113	0.86237
C	4.63607	-3.8323	-3.17725	H	-0.17608	-1.69116	-2.10214
C	-2.81126	0.96549	5.35296	H	1.26327	-1.06098	-1.3079
H	-2.91397	2.05339	5.32262	H	-0.51632	0.67367	-1.41134
C	6.22446	-1.90734	1.90505	H	-1.37728	-3.65683	0.95364
H	5.30922	-1.34649	1.66593	O	-3.52951	-2.01645	0.77638
C	-6.23948	-3.4439	-1.66427	C	0.92512	-3.71071	-0.59126
C	-3.77391	0.15052	4.8351	H	-3.74982	-2.81142	0.23981
H	-4.6735	0.57732	4.39722	H	0.50866	-4.11014	-1.53014
C	4.06541	3.94718	-3.26043	H	0.90929	-4.52469	0.14461
C	2.88231	4.38725	-3.93676	H	1.97697	-3.47212	-0.78222
C	2.05718	5.38976	-3.30245	H	-2.51793	-0.82003	-1.34828

Table S10b. Cartesian coordinates of **1a•*ax*-PMD** (R = -OCH$_3$; E_{opt} = 1.54133491783 hartree, E_{sp} = – 9363.63878602 hartree).

C	-2.75631	0.58489	-0.17374	C	-4.78298	2.48826	6.71868
C	-2.78181	2.09631	-0.35781	H	-5.51322	1.95142	7.3283
C	-2.43892	-0.15069	-1.46553	C	-5.04284	2.80641	5.41763
O	-4.16584	0.2576	0.17245	H	-5.98872	2.52207	4.96305
H	-2.90585	2.64263	0.58624	C	1.69606	-6.04974	0.23225
H	-3.59564	2.41181	-1.02967	H	0.99048	-6.56503	-0.41801
H	-1.8487	2.45366	-0.82087	C	8.38287	-1.57127	-2.56064
H	-3.00114	0.24394	-2.32137	H	9.3161	-1.16274	-2.14703
H	-2.63214	-1.22626	-1.41041	C	5.76693	2.67944	-5.22788
H	-1.36669	-0.02901	-1.71379	H	6.67227	2.27799	-4.78056
H	-4.2012	-0.64856	0.55964	C	1.64963	-1.8869	-5.88318
C	-1.84631	-1.35428	1.26924	H	0.7413	-2.3117	-6.31663
C	-1.88519	0.16746	1.03976	C	4.34315	3.04954	-7.16192
C	-0.43802	0.71628	0.94416	H	4.18989	2.90531	-8.23297
C	0.35667	0.37951	2.21341	C	0.94151	-4.67895	-5.46616
C	0.36404	-1.13212	2.51612	C	0.90087	-5.58979	-6.56683
C	-1.07434	-1.68553	2.55177	C	-0.32059	-6.19148	-6.92463
H	-1.378	-1.86249	0.40302	C	-1.51625	-5.84641	-6.22927
O	0.2398	0.21613	-0.21754	C	-1.49306	-4.85737	-5.22394
H	-0.06074	0.93539	3.07499	C	-0.24814	-4.31551	-4.8465
H	0.92261	-1.65026	1.69644	H	-0.22079	-3.58302	-4.03429
H	-1.61272	-1.28005	3.43328	C	-4.35062	3.91284	3.28785
H	-1.04443	-2.78665	2.70778	C	2.90076	-5.52843	-0.34597
H	-2.87431	-1.75984	1.33829	H	3.05513	-5.65097	-1.42177
H	1.39613	0.74345	2.10006	C	-6.11259	-1.43957	2.14527
C	1.08112	-1.40653	3.83948	H	-5.17644	-1.32138	1.57471

H	0.52422	-1.01239	4.69853	C	6.44555	2.00867	3.31693
H	2.07491	-0.94269	3.86559	C	3.83317	-4.91118	0.43497
H	1.22543	-2.4802	4.01053	H	4.75689	-4.54441	-0.00538
H	-2.34712	0.63871	1.94822	C	-3.72915	2.72428	-4.36702
H	-0.43139	1.81467	0.74961	C	-2.52202	3.28746	-4.89346
H	0.39086	-0.74994	-0.14344	C	-1.68043	2.45949	-5.72708
Pt	7.3277	-0.38338	-0.10943	H	-0.78388	2.91091	-6.16112
Pt	-7.08308	0.46577	0.11785	C	-1.99552	1.15772	-5.97823
N	7.41919	1.33588	-1.11817	H	-1.36489	0.53252	-6.60967
C	6.27929	1.85267	-1.67706	C	-3.1889	0.58687	-5.42424
H	5.34281	1.29123	-1.53847	H	-3.41103	-0.46101	-5.64311
C	6.30491	3.05686	-2.40512	C	-4.02725	1.3411	-4.6565
C	7.53417	3.73248	-2.54706	H	-4.94605	0.91101	-4.26516
H	7.57306	4.67555	-3.11116	C	-5.89921	-5.05668	2.4026
C	8.68703	3.19604	-1.97209	C	-4.94987	-3.23418	-3.22073
H	9.65206	3.70943	-2.07445	C	2.41203	-5.24554	2.43106
C	8.61266	1.99188	-1.25992	C	4.15978	4.31994	-2.21659
H	9.49869	1.53745	-0.79448	C	8.64647	0.64901	2.28552
N	7.4536	0.61892	1.61198	H	9.48997	0.09965	1.84334
N	7.20829	-2.10779	0.88993	C	1.94269	-0.56521	-6.03996
C	8.31886	-2.89618	1.02885	H	1.28043	0.10241	-6.58925
H	9.25595	-2.53344	0.58243	C	3.44025	3.73156	-6.40159
C	8.24957	-4.11748	1.71192	H	2.53641	4.1467	-6.85519
H	9.15041	-4.73701	1.8129	C	-1.91522	4.70052	4.5012
C	7.03533	-4.53705	2.25625	C	5.26421	2.72369	3.83533
H	6.96083	-5.49372	2.79289	C	8.35366	-2.26	-3.77996
C	5.89023	-3.72601	2.11648	H	9.28234	-2.40206	-4.34813
C	6.01005	-2.50732	1.42329	C	2.33913	5.8487	-0.66808
H	5.14635	-1.83922	1.28985	H	1.67551	6.44916	-0.04798
C	-5.0103	-3.09101	3.65948	C	2.10718	5.68021	-2.00064
N	-6.96052	2.06949	1.29243	H	1.249	6.15614	-2.48093
C	-8.08489	2.80731	1.5583	C	1.78315	4.79055	5.31293
H	-9.02539	2.47662	1.09566	C	0.54727	4.44329	4.76059
C	-8.01998	3.93473	2.38385	H	0.50617	3.6487	4.0101
H	-8.92702	4.51885	2.58399	C	-0.63282	5.0936	5.12479
C	-6.79781	4.30636	2.94915	C	-0.57768	6.13849	6.09128
H	-6.72807	5.18658	3.60335	C	0.64611	6.44375	6.73168
C	-5.64422	3.54476	2.67989	C	1.83481	5.79919	6.31663
C	-5.75466	2.42566	1.83346	C	-3.58907	-3.48744	-5.25155
H	-4.8803	1.79517	1.56533	C	-4.74361	-2.95692	-4.58958
C	4.03597	-0.83324	-4.83461	C	-5.67103	-2.15635	-5.35413
H	4.96731	-0.42479	-4.45104	H	-6.55653	-1.76557	-4.85799
C	3.14256	-4.99282	-4.31461	C	-5.46098	-1.9083	-6.67939
C	4.3849	-4.47017	-3.83096	H	-6.17162	-1.32226	-7.26738
C	5.31117	-5.37143	-3.18742	C	-4.29182	-2.41635	-7.33831
H	6.26013	-4.98035	-2.83011	H	-4.15275	-2.19144	-8.39783
C	5.02288	-6.69882	-3.05212	C	-3.39054	-3.17006	-6.64814
H	5.73122	-7.3891	-2.58852	H	-2.49708	-3.56038	-7.14331
C	3.77467	-7.22333	-3.52394	C	5.11038	-3.26772	6.28425
H	3.57838	-8.2901	-3.40205	H	5.86018	-2.83959	6.95359
C	2.87053	-6.39996	-4.12651	C	2.31682	2.04374	6.07407
H	1.92252	-6.79753	-4.49809	H	1.48096	2.59993	6.50523
N	-7.19325	-0.70896	1.72912	C	2.99399	4.88958	-2.82317

C	3.76344	-2.23942	-4.6504	C	7.67235	2.03729	4.0089
C	2.23165	-4.13988	-4.98142	H	7.75229	2.59832	4.95111
C	6.36589	1.28448	2.1122	C	5.53333	2.5196	-6.56331
H	5.4293	1.23591	1.53688	H	6.24285	1.99161	-7.2044
C	-4.88877	-4.4278	3.22005	C	1.46684	-5.91829	1.56916
C	2.53651	-2.77216	-5.16307	H	0.56602	-6.33692	2.02357
C	6.04144	-1.86518	-2.29884	C	2.51828	0.73388	6.39286
H	5.14619	-1.68733	-1.685	H	1.85511	0.20811	7.07865
C	4.51506	0.65153	5.00964	C	-1.49989	-5.3513	4.72222
H	5.37208	0.11431	4.61154	C	-0.29817	-4.95075	4.13148
C	3.13129	-4.45344	4.63678	H	-0.31027	-4.08961	3.44484
C	4.34437	2.04276	4.66115	C	0.91066	-5.59225	4.40456
C	4.36597	-3.99746	4.07211	C	0.90864	-6.73094	5.26334
C	-3.7321	-5.18914	3.58659	C	-0.30115	-7.18017	5.84053
C	1.52366	5.24974	-4.8181	C	-1.4951	-6.45192	5.62434
C	0.27384	4.73052	-4.50065	C	-7.4073	-2.38806	3.95612
H	0.20451	3.93281	-3.75451	H	-7.4861	-3.05033	4.82989
C	-0.91177	5.19343	-5.1052	C	-8.50129	-1.63424	3.5293
C	-0.81211	6.17392	-6.1142	H	-9.45908	-1.69003	4.06313
C	0.4496	6.76298	-6.42009	C	4.37034	4.52119	-0.80201
C	1.61039	6.32569	-5.7548	H	5.2558	4.09486	-0.33798
C	3.00587	4.10345	4.84412	C	3.48975	5.24955	-0.05712
N	7.2361	-1.37925	-1.83655	H	3.64714	5.40239	1.01355
C	-2.74105	-4.60996	4.41159	C	-5.7699	-6.35364	1.99792
C	4.84017	3.40475	-4.39174	C	-4.04094	-4.04061	-2.50203
C	4.67222	-3.09645	-3.99376	C	-4.22391	-4.33459	-1.09983
C	3.86504	-3.70037	6.84869	H	-5.08689	-3.91851	-0.58582
H	3.71002	-3.57911	7.92208	C	-3.35261	-5.1435	-0.43103
C	-4.03987	-2.51831	4.50899	H	-3.49347	-5.37193	0.62906
C	-4.15186	-1.16291	4.99471	C	-2.23657	-5.73256	-1.11213
H	-5.00884	-0.56705	4.68972	H	-1.57798	-6.3963	-0.55293
C	-3.21781	-0.6408	5.84094	C	-2.03098	-5.47781	-2.43521
H	-3.30242	0.38288	6.21456	H	-1.19734	-5.94207	-2.96971
C	-2.10236	-1.43159	6.27424	C	-2.91141	-4.60459	-3.17724
H	-1.39725	-0.99305	6.97954	C	-4.60769	-7.11513	2.35411
C	-1.95579	-2.71031	5.8259	H	-4.54092	-8.15047	2.01574
H	-1.12329	-3.33041	6.16798	C	3.62902	-4.75429	1.85626
C	-2.90455	-3.29542	4.9058	C	4.59669	-4.14909	2.68658
N	-7.21468	-1.13128	-1.0656	C	-3.41907	4.67369	2.55034
C	-6.09796	-1.59466	-1.70984	C	-3.67183	5.07895	1.187
H	-5.15502	-1.03812	-1.54472	H	-4.6118	4.7904	0.72242
C	-6.15929	-2.72401	-2.54607	C	-2.76712	5.83248	0.49826
C	-7.40019	-3.36886	-2.72145	H	-2.95793	6.14442	-0.53177
H	-7.46836	-4.25056	-3.37252	C	-1.542	6.24764	1.11808
C	-8.53152	-2.88092	-2.06595	H	-0.85504	6.86923	0.54493
H	-9.50572	-3.36984	-2.19416	C	-1.26915	5.88663	2.40363
C	-8.42008	-1.75781	-1.23692	H	-0.3511	6.21931	2.89436
H	-9.28482	-1.34281	-0.69901	C	-2.18972	5.07446	3.16626
C	-6.18759	-2.29908	3.25599	H	-6.78696	-4.48728	2.13873
C	2.74765	4.6918	-4.20101	H	-6.54465	-6.84242	1.40291
C	5.96293	-2.5676	-3.51613	O	-2.70455	-6.67489	6.18989
C	5.07128	3.59137	-3.01043	O	-0.3015	-8.27912	6.68906
C	-3.62687	-6.55087	3.11421	O	2.14338	-7.26118	5.42941

H	-2.74399	-7.12715	3.40002	O	-1.80873	6.70503	-6.87316
C	2.914	-4.26726	6.0536	O	0.60006	7.65385	-7.4641
H	1.96663	-4.60815	6.4781	O	2.87434	6.7863	-5.92155
C	5.34604	-3.39717	4.94631	O	-2.58515	-6.54995	-6.69231
H	6.29122	-3.06594	4.52349	O	-0.28877	-7.21811	-7.84704
N	-7.0735	1.63447	-1.49381	O	2.11581	-5.8016	-7.12827
C	-8.25872	1.93191	-2.11695	O	-1.77585	6.73901	6.28399
H	-9.175	1.50095	-1.68887	O	0.69509	7.4355	7.70222
C	-8.28209	2.75073	-3.25109	O	3.0928	6.02785	6.76259
H	-9.23781	2.97685	-3.74092	C	-3.0941	6.04068	-6.98127
C	-7.08776	3.27697	-3.7514	C	-0.17162	8.88485	-7.35923
H	-7.09117	3.92288	-4.6393	C	3.12422	7.88572	-6.84779
C	-5.87272	2.97696	-3.10803	C	-3.80922	-6.63405	-5.91769
C	-5.9005	2.14021	-1.97616	C	-1.15051	-7.04912	-9.00934
H	-4.96526	1.86614	-1.43155	C	2.23528	-6.70411	-8.2688
C	3.21126	2.74536	5.18224	C	-2.83257	-7.59779	7.31314
C	-4.59399	3.52995	-3.59524	C	-0.58614	-9.53699	6.00549
C	-4.26706	4.87479	-3.31236	C	2.37332	-8.30235	6.42464
C	-5.13848	5.72553	-2.53623	C	-1.86363	7.9723	7.05869
H	-6.0784	5.31785	-2.17123	C	0.46922	6.93262	9.05377
C	-4.80893	7.0244	-2.27866	C	3.37054	7.15161	7.64962
H	-5.47588	7.6776	-1.7107	H	-3.48993	6.49888	-7.90219
C	-3.56952	7.56868	-2.75403	H	-3.71177	6.30386	-6.11835
H	-3.33717	8.61075	-2.52566	H	-2.97856	4.96183	-7.10753
C	-2.71834	6.79476	-3.48446	H	0.0715	9.41446	-6.4374
H	-1.77217	7.20217	-3.85113	H	-1.24291	8.66401	-7.43465
C	-3.03939	5.42256	-3.8066	H	0.17434	9.43283	-8.24617
C	3.15681	-0.02781	-5.49725	H	2.63284	8.79309	-6.48728
H	3.36203	1.03688	-5.63904	H	2.77828	7.62088	-7.85485
C	3.63672	0.02377	5.84365	H	4.21788	7.96178	-6.79543
H	3.76835	-1.02563	6.11844	H	-4.42195	-5.74997	-6.11395
C	3.94222	4.78569	4.03414	H	-3.60359	-6.78055	-4.85508
C	3.78882	6.18551	3.7081	H	-4.27081	-7.53591	-6.35178
H	2.90929	6.70935	4.09029	H	-0.88997	-7.93655	-9.60209
C	4.72149	6.84696	2.96607	H	-0.90816	-6.12612	-9.53737
H	4.61798	7.90928	2.73813	H	-2.2031	-7.08295	-8.70455
C	5.87905	6.15326	2.47998	H	1.86532	-7.70259	-8.0041
H	6.61379	6.71785	1.90153	H	1.69143	-6.2949	-9.1241
C	6.05168	4.82511	2.74253	H	3.32094	-6.69719	-8.43062
H	6.93386	4.30535	2.37801	H	-3.76198	-7.24237	7.77884
C	5.09324	4.09314	3.53642	H	-1.97953	-7.5219	7.99685
C	-2.69076	-4.32146	-4.54591	H	-2.9404	-8.6126	6.92027
C	3.65619	3.94626	-4.98848	H	-1.58605	-9.51415	5.56556
C	-2.18417	4.63055	-4.60832	H	-0.53109	-10.25805	6.83245
C	7.1434	-2.75991	-4.26234	H	0.17563	-9.74183	5.24897
H	7.09942	-3.30301	-5.21751	H	1.825	-8.10081	7.35198
C	8.77309	1.35413	3.48871	H	3.45982	-8.22751	6.567
H	9.73689	1.36701	4.01446	H	2.08934	-9.26691	5.9941
C	-8.37835	-0.80146	2.40856	H	-1.01457	8.63559	6.85801
H	-9.21961	-0.19714	2.03864	H	-1.9265	7.71452	8.11961
C	2.17289	-5.09286	3.81579	H	-2.80749	8.39354	6.6872
C	-4.08974	3.55111	4.62802	H	-0.52942	6.49696	9.13664
C	-2.85199	3.93811	5.23547	H	0.55474	7.8505	9.65107

C	-2.60869	3.55491	6.60777	H	1.24158	6.20873	9.32612
H	-1.66076	3.85073	7.06286	H	3.12915	6.85647	8.67484
C	-3.53731	2.8613	7.32475	H	4.45318	7.27363	7.50919
H	-3.36556	2.59135	8.36778	H	2.81569	8.04779	7.34951

Table S11a. Cartesian coordinates of **1a•MCA-a** (R = -OCH_3; E_{opt} = 1.53304601856 hartree, E_{sp} = –9754.15660338 hartree).

Pt	-7.2952	0.45958	0.02661	C	-1.61738	6.14828	0.56895
Pt	7.19959	-0.55359	-0.1026	H	-0.89948	6.67905	-0.05464
N	-7.15386	1.51133	-1.66548	C	-2.81812	6.73513	-3.73598
C	-5.93686	2.0323	-2.02601	H	-1.87153	7.1652	-4.07392
H	-5.08176	1.83634	-1.35979	C	1.93181	-4.34941	-4.69217
C	-5.78569	2.79128	-3.20107	C	-5.39553	-3.50652	-3.13613
C	-6.92067	3.00555	-4.01196	C	-8.18881	4.14986	1.90789
H	-6.82347	3.59471	-4.93511	H	-9.09252	4.76425	2.01188
C	-8.15225	2.47131	-3.634	C	-1.46943	0.86779	-5.26131
H	-9.04415	2.63147	-4.25366	H	-0.72731	0.20646	-5.70665
C	-8.25282	1.72505	-2.45188	C	-1.28061	2.21687	-5.21175
H	-9.20645	1.29059	-2.11965	H	-0.38323	2.6648	-5.64529
N	-7.51985	-1.22286	-1.03079	C	-1.79914	-4.96511	-5.02076
N	-7.35329	-0.57056	1.73482	C	-0.56368	-4.50936	-4.55511
C	-8.49881	-0.63696	2.48296	H	-0.53422	-3.82836	-3.6987
H	-9.39002	-0.12687	2.09103	C	0.63513	-4.88174	-5.16851
C	-8.51798	-1.32958	3.70025	C	0.59724	-5.76784	-6.28352
H	-9.44511	-1.37339	4.28658	C	-0.63464	-6.31228	-6.71471
C	-7.35888	-1.95828	4.16044	C	-1.83848	-5.87675	-6.11476
H	-7.35677	-2.50312	5.11496	C	3.8082	4.90877	3.87513
C	-6.18087	-1.89398	3.39074	C	4.94914	4.1751	3.41443
C	-6.21239	-1.18675	2.1746	C	6.03257	4.9016	2.79558
H	-5.3063	-1.12624	1.53072	H	6.90786	4.35111	2.45962
C	4.73668	-4.14131	3.18848	C	5.97733	6.25709	2.645
N	7.09741	-1.61558	-1.79177	H	6.80426	6.8128	2.19619
C	8.22071	-1.84795	-2.54151	C	4.82233	6.98995	3.07585
H	9.17195	-1.44453	-2.16715	H	4.80794	8.07211	2.93102
C	8.14444	-2.572	-3.7379	C	3.77846	6.33804	3.6616
H	9.05508	-2.75037	-4.32477	H	2.89423	6.89083	3.99108
C	6.91251	-3.06327	-4.17652	C	-5.54359	-6.04503	2.64093
H	6.83633	-3.63291	-5.1135	H	-6.30145	-6.64707	2.13555
C	5.75705	-2.82797	-3.40649	C	-2.82135	-6.20362	-2.59868
C	5.88675	-2.09496	-2.21192	H	-1.9176	-6.56964	-3.09167
H	4.9991	-1.9062	-1.56569	C	-2.25819	3.09307	-4.60809
C	-3.78043	5.04928	0.71625	C	-7.85277	-3.52796	-2.57812
H	-4.71261	4.72621	0.26014	H	-7.98221	-4.43003	-3.19278
C	-3.09178	4.37364	4.8925	C	-5.02319	6.96311	-2.75639
C	-4.33144	3.96437	4.3046	H	-5.77762	7.63113	-2.33409
C	-5.3307	3.35449	5.14974	C	-1.84491	-1.71226	5.81207
H	-6.27713	3.05355	4.7082	H	-0.97893	-2.23575	6.22375
C	-5.113	3.18335	6.4863	C	-3.28988	-6.80015	-1.46669
H	-5.87667	2.74831	7.13479	H	-2.77928	-7.65384	-1.02086
C	-3.86711	3.57833	7.07585	C	1.00209	-4.98116	5.17357
H	-3.7273	3.42858	8.14789	C	-0.14025	-4.3154	4.72211
C	-2.89473	4.14438	6.30596	H	-0.05421	-3.61124	3.88567

H	-1.94714	4.45501	6.75355	C	-1.39334	-4.53683	5.29993
N	7.12287	-2.23179	0.98664	C	-1.5002	-5.4275	6.40738
C	-3.57505	4.8078	2.12545	C	-0.34884	-6.07641	6.90946
C	-2.11052	5.00989	4.09533	C	0.89215	-5.9006	6.25513
C	-6.4456	-1.78812	-1.66043	C	7.12314	-4.56465	2.5278
H	-5.45697	-1.29625	-1.52009	H	7.11974	-5.47848	3.13942
C	4.46496	-3.5474	4.44133	C	8.26675	-4.18155	1.82346
C	-2.34732	5.2427	2.72264	H	9.17946	-4.79003	1.86993
C	-5.9377	2.55139	1.61039	C	-3.65063	1.08784	-4.21598
H	-5.06574	1.89131	1.47379	H	-4.58179	0.64687	-3.86901
C	-5.17429	-5.26572	-1.3757	C	-2.6767	0.2934	-4.74579
H	-6.0945	-4.91895	-0.91094	H	-2.80439	-0.78964	-4.80826
C	-3.55842	-4.57993	4.03484	C	5.14989	-2.1429	6.32158
C	-4.70939	-4.6039	-2.57271	C	3.90973	2.08635	4.17744
C	-4.75185	-3.93144	3.58081	C	3.88523	0.64737	4.29031
C	3.24115	-3.85453	5.119	H	4.72081	0.07323	3.89844
C	-0.78524	5.07896	-5.01476	C	2.83812	0.00554	4.88413
C	0.43647	4.75237	-4.43795	H	2.80692	-1.08554	4.94547
H	0.46034	4.03037	-3.60474	C	1.7589	0.75156	5.46204
C	1.65795	5.29652	-4.87802	H	0.96273	0.20675	5.96817
C	1.62508	6.28819	-5.88027	C	1.75492	2.11224	5.38875
C	0.39428	6.6163	-6.52058	H	0.95486	2.69117	5.85823
C	-0.80032	5.98855	-6.11791	C	2.8042	2.83096	4.70147
C	-3.05752	-4.49588	-4.39763	C	3.92823	-2.45479	7.00531
N	-7.12957	2.15761	1.06289	H	3.76676	-2.03495	7.99913
C	2.3123	-4.74144	4.52801	C	-3.96825	-1.81723	4.54838
C	-4.25887	4.74686	-3.45417	C	-4.93833	-2.55484	3.83714
C	-4.54809	4.17046	2.92364	C	3.57031	-2.51168	-4.57349
C	-4.33785	-6.69136	3.07067	C	3.92186	-1.15468	-4.92098
H	-4.21566	-7.75575	2.86642	H	4.87575	-0.7587	-4.58175
C	3.86399	-5.11319	2.65064	C	3.08935	-0.38535	-5.68004
C	4.17351	-5.83765	1.44	H	3.35655	0.63919	-5.95136
H	5.11805	-5.64008	0.93886	C	1.84165	-0.90978	-6.15407
C	3.31255	-6.77025	0.93902	H	1.21968	-0.27681	-6.78652
H	3.54875	-7.32665	0.02968	C	1.46932	-2.18165	-5.83535
C	2.07227	-7.05136	1.60229	H	0.52959	-2.59862	-6.20594
H	1.41698	-7.81257	1.1794	C	2.3108	-3.02762	-5.02037
C	1.7461	-6.39045	2.74855	H	6.33633	-2.42885	4.57477
H	0.8145	-6.60975	3.27616	H	5.86999	-1.49423	6.8242
C	2.62953	-5.39908	3.31882	O	2.06273	-6.52247	6.53346
N	7.26455	0.53147	1.57123	O	-0.45276	-6.94881	7.98471
C	6.16125	1.23986	1.9708	O	-2.77125	-5.54647	6.86098
H	5.25693	1.17761	1.34298	O	2.66566	7.0039	-6.3854
C	6.18036	2.01994	3.14279	O	0.30198	7.64572	-7.43579
C	7.36699	2.062	3.90323	O	-2.03713	6.16995	-6.64319
H	7.40307	2.66421	4.82138	O	2.65584	6.30451	6.83476
C	8.48407	1.33856	3.48392	O	0.41114	7.62096	7.38638
H	9.41504	1.36179	4.06538	O	-1.94157	7.12213	5.79793
C	8.41586	0.5756	2.31136	O	1.82116	-5.98461	-6.82232
H	9.27325	-0.00965	1.94884	O	-0.66898	-7.19951	-7.78216
C	5.96343	-3.76947	2.45721	O	-3.10172	-6.21887	-6.4617
C	-2.04308	4.4882	-4.50547	C	3.92996	7.06901	-5.67502
C	-5.83249	3.75974	2.32648	C	1.13041	7.51159	-8.62625

C	-4.47765	3.35618	-3.58238	C	-2.21495	7.07228	-7.77588
C	3.00693	-3.27191	6.42021	C	3.76416	5.38849	7.03378
H	2.07661	-3.52343	6.93479	C	1.37	8.71505	7.31384
C	-3.38404	-5.98388	3.74013	C	-2.03658	8.24329	6.72717
H	-2.46759	-6.47019	4.08322	C	2.09334	-7.65625	7.45106
C	-5.73798	-4.71467	2.87448	C	-0.21843	-6.30626	9.27417
H	-6.65531	-4.22969	2.55015	C	-3.11001	-6.56321	7.85
N	7.34228	1.12834	-1.1713	C	1.94939	-6.67929	-8.09878
C	8.58106	1.69745	-1.32516	C	-0.56613	-8.59728	-7.37449
H	9.43279	1.1857	-0.85429	C	-3.35228	-6.98176	-7.67936
C	8.73984	2.88274	-2.05134	H	4.38071	7.96564	-6.13041
H	9.73854	3.32475	-2.16341	H	4.51981	6.17691	-5.90222
C	7.62471	3.49469	-2.62898	H	3.77955	7.21596	-4.60292
H	7.73184	4.42597	-3.20195	H	0.90488	6.58224	-9.15025
C	6.35301	2.91022	-2.47607	H	2.19074	7.583	-8.35594
C	6.24399	1.7182	-1.7333	H	0.81911	8.39127	-9.20595
H	5.25495	1.22476	-1.59133	H	-1.68624	6.6809	-8.64878
C	-3.51437	-5.08658	-3.19901	H	-1.8635	8.07947	-7.51834
C	5.15837	3.5261	-3.08671	H	-3.30424	7.03768	-7.90477
C	4.87205	3.28345	-4.4483	H	4.50308	5.54803	6.24349
C	5.72072	2.45156	-5.26896	H	3.4279	4.35137	7.09496
H	6.61116	2.01414	-4.82417	H	4.15029	5.73028	8.00767
C	5.43528	2.2368	-6.58593	H	1.09726	9.3007	8.20229
H	6.08806	1.62938	-7.21714	H	1.23174	9.28743	6.3958
C	4.26297	2.81273	-7.17898	H	2.39107	8.32634	7.40526
H	4.06395	2.61716	-8.23445	H	-1.75461	7.92391	7.73825
C	3.43404	3.59683	-6.43351	H	-1.40489	9.06582	6.38194
H	2.53949	4.03935	-6.88008	H	-3.10441	8.48712	6.65545
C	3.71409	3.87915	-5.044	H	3.02234	-8.15626	7.14515
C	-2.83713	5.69059	-0.03154	H	1.21944	-8.30442	7.32011
H	-2.99336	5.88014	-1.09683	H	2.15827	-7.27548	8.47419
C	-4.49349	-6.32387	-0.84799	H	0.79844	-5.9091	9.32288
H	-4.84932	-6.83511	0.04892	H	-0.35226	-7.14554	9.96994
C	-3.79906	-3.45693	-5.00554	H	-0.95911	-5.52215	9.45055
C	-3.41929	-2.90737	-6.28709	H	-2.59207	-7.50809	7.64975
H	-2.5293	-3.31139	-6.77573	H	-4.19488	-6.65238	7.70453
C	-4.16153	-1.93436	-6.8874	H	-2.86742	-6.17812	8.84459
H	-3.89091	-1.53431	-7.86592	H	1.16265	-6.37976	-8.80064
C	-5.33669	-1.42457	-6.24173	H	1.92967	-7.75701	-7.91421
H	-5.91233	-0.65202	-6.75568	H	2.94108	-6.34249	-8.42896
C	-5.72258	-1.90437	-5.0236	H	0.38935	-8.78008	-6.87692
H	-6.62454	-1.52885	-4.54743	H	-0.6185	-9.11651	-8.34103
C	-4.9785	-2.95355	-4.36767	H	-1.40675	-8.87056	-6.73156
C	2.7556	4.23603	4.54077	H	-3.19838	-8.04322	-7.46499
C	-3.02079	5.31305	-3.9013	H	-4.41199	-6.75581	-7.86029
C	2.88773	4.73692	-4.2788	H	-2.7157	-6.64315	-8.50462
C	-6.97984	4.56499	2.46907	C	1.77422	-2.61054	-0.17313
H	-6.91293	5.51276	3.02247	C	1.12794	-2.27544	-1.52927
C	-8.94272	-2.94871	-1.9229	C	-0.22078	-1.58873	-1.3048
H	-9.94347	-3.39175	-2.01097	C	-1.18633	-2.5005	-0.52779
C	8.24959	-3.01066	1.0572	C	-0.53953	-2.96155	0.79797
H	9.12964	-2.67627	0.48923	C	0.85915	-3.55665	0.62671
C	-2.59998	-3.85476	4.78101	O	-0.3346	-1.811	1.65714

C	4.06903	-4.66384	-3.50607	C	-1.49379	-1.02163	1.94802
C	2.79453	-5.1656	-3.92571	O	-2.24534	-0.703	0.76392
C	2.43821	-6.52141	-3.57409	C	-2.50853	-1.76657	-0.18784
H	1.4612	-6.8929	-3.89183	C	3.15815	-3.23119	-0.36674
C	3.29649	-7.32209	-2.88114	H	1.87482	-1.66146	0.41565
H	3.0375	-8.35222	-2.63358	H	1.7945	-1.60498	-2.11855
C	4.58123	-6.82435	-2.48219	H	1.01011	-3.19024	-2.13916
H	5.25623	-7.50097	-1.95389	H	-0.65535	-1.2914	-2.27497
C	4.94852	-5.54234	-2.77081	H	-0.07539	-0.64694	-0.71453
H	5.92493	-5.17104	-2.47	H	0.78574	-4.54296	0.13064
C	-2.02692	-0.38095	6.03869	H	1.3021	-3.7473	1.62564
H	-1.32081	0.19726	6.63325	C	-0.94033	0.29188	2.50238
C	-8.24625	2.93989	1.20686	C	-3.08699	-0.99614	-1.37909
H	-9.17781	2.57737	0.74847	C	-3.54221	-2.72428	0.41029
C	-5.24964	5.62204	-2.87447	H	3.8323	-2.53504	-0.88274
H	-6.19327	5.19822	-2.54089	H	3.11012	-4.15034	-0.96791
C	-1.38662	5.93665	1.89521	H	3.60404	-3.50315	0.59552
H	-0.473	6.30386	2.36839	H	-1.4177	-3.39991	-1.14593
C	-3.77987	7.53105	-3.18866	H	-3.12899	-3.34205	1.22022
H	-3.63171	8.60711	-3.07816	H	-3.92926	-3.42002	-0.34366
C	-0.82024	5.40569	4.70258	H	-4.38618	-2.17243	0.84237
C	-0.75914	6.47842	5.64537	H	-4.11794	-0.667	-1.19019
C	0.44351	6.72751	6.33419	H	-3.07639	-1.60512	-2.29183
C	1.60318	5.95195	6.04519	H	-2.49693	-0.08631	-1.57236
C	1.57642	4.98595	5.01754	C	-0.33067	1.18632	1.39673
C	0.3449	4.71976	4.38534	C	0.87143	1.97484	1.95063
H	0.30848	3.92773	3.61478	C	1.63779	2.7623	0.87691
C	4.4365	-3.33817	-3.82585	C	2.38753	1.87552	-0.07131
C	-3.18326	0.28696	5.51465	C	2.02659	1.60169	-1.33485
H	-3.29611	1.35714	5.70909	C	0.77945	2.10314	-1.98708
C	5.99536	-2.59787	1.67234	C	2.88582	0.74704	-2.22053
H	5.11226	-1.9442	1.59004	H	-0.16813	0.03576	3.25904
C	-6.57768	-2.9452	-2.45242	H	-1.73717	0.82992	3.04899
C	-4.12034	-0.40386	4.80436	H	1.56607	1.27787	2.46104
H	-5.00624	0.10316	4.42967	H	0.5246	2.68509	2.73676
C	4.3353	4.37534	-2.31634	H	2.36043	3.4443	1.37561
C	3.20032	5.00177	-2.92662	H	0.94178	3.43331	0.33258
C	2.40338	5.90946	-2.13227	H	3.2916	1.44708	0.35427
H	1.56527	6.4162	-2.61815	H	0.98589	3.005	-2.59269
C	2.69267	6.1414	-0.82142	H	0.00225	2.37954	-1.24866
H	2.09755	6.82986	-0.22176	H	0.33094	1.35481	-2.6537
C	3.81095	5.48751	-0.20375	H	2.42784	-0.25505	-2.35752
H	4.01018	5.68956	0.85197	H	3.89831	0.595	-1.82074
C	4.60648	4.64554	-0.9237	H	2.98853	1.18894	-3.22314
H	5.46273	4.16701	-0.45531	H	0.03525	0.50774	0.57282
C	5.40182	-2.65448	5.08131	C	-1.40277	2.11652	0.81774
C	4.98647	2.77159	3.57251	H	-1.7273	2.87567	1.53606
C	-2.79492	-2.47891	5.03822	H	-2.28123	1.53459	0.51084
C	-3.47835	2.51886	-4.12405	H	-1.02611	2.64336	-0.07312
C	-8.7608	-1.79237	-1.15473	H	-1.20969	-3.66821	1.34105
H	-9.59525	-1.30616	-0.62979	H	-2.15421	-1.58855	2.63391

Table S11b. Cartesian coordinates of **1a•MCA-b** (R = -OCH_3; E_{opt} = 1.52995493021 hartree, E_{sp} = –9754.15235992 hartree).

Pt	-6.73623	-1.71324	-1.88883	C	-2.23798	-4.296	4.22642
Pt	6.59984	1.61101	1.66555	H	-1.90099	-3.96963	5.20941
N	-7.47476	-1.05435	-0.15567	C	-4.97859	-1.56693	6.44203
C	-6.61307	-0.74448	0.86146	H	-4.28914	-1.33096	7.25695
H	-5.51892	-0.8309	0.66685	C	0.66884	6.76609	-0.22512
C	-7.08323	-0.31909	2.1184	C	-5.57759	3.52675	-2.71161
C	-8.47416	-0.1954	2.30783	C	-7.41033	-5.61142	-0.36584
H	-8.86294	0.14251	3.27897	H	-8.2853	-6.26903	-0.28328
C	-9.34306	-0.50436	1.25962	C	-3.90208	3.59714	3.16993
H	-10.42895	-0.41107	1.39064	H	-3.36749	4.54668	3.14648
C	-8.82662	-0.93931	0.03229	C	-3.77413	2.73516	4.21788
H	-9.47925	-1.19864	-0.81292	H	-3.13639	2.98544	5.06919
N	-7.06634	0.07214	-2.71692	C	-2.82713	6.68369	-1.68512
N	-5.94663	-2.37991	-3.59683	C	-1.56497	6.30287	-1.22273
C	-6.72256	-3.07967	-4.48126	H	-1.28618	5.23595	-1.23541
H	-7.77642	-3.2403	-4.21275	C	-0.64473	7.23041	-0.72422
C	-6.18053	-3.57003	-5.6761	C	-1.00798	8.60708	-0.69333
H	-6.81631	-4.13041	-6.37366	C	-2.22619	9.03129	-1.27457
C	-4.83519	-3.34327	-5.96763	C	-3.15148	8.07025	-1.74199
H	-4.39235	-3.72239	-6.89955	C	4.16109	-5.27275	3.36839
C	-4.03258	-2.62129	-5.0591	C	5.16488	-4.25299	3.29107
C	-4.6232	-2.14981	-3.87241	C	5.79825	-3.81583	4.51319
H	-4.04159	-1.57845	-3.12691	H	6.56951	-3.05165	4.45753
C	6.45452	0.51553	-3.68312	C	5.46262	-4.36541	5.71629
N	6.07236	3.53684	1.61912	H	5.9581	-4.05657	6.63974
C	6.94972	4.45153	2.14172	C	4.44483	-5.37334	5.79816
H	7.91123	4.07215	2.51447	H	4.2017	-5.78946	6.77787
C	6.61963	5.81	2.20067	C	3.81351	-5.80054	4.66846
H	7.33365	6.52835	2.62298	H	3.03534	-6.56722	4.72184
C	5.37917	6.23713	1.72632	C	-2.92153	0.38886	-7.86728
H	5.09679	7.29884	1.77397	H	-3.67478	1.02784	-8.33351
C	4.47505	5.30219	1.17973	C	-2.50578	5.15655	-4.14548
C	4.85665	3.94492	1.13744	H	-1.86454	6.01572	-3.92722
H	4.18448	3.17687	0.71924	C	-4.48995	1.48052	4.24971
C	-3.95341	-4.1783	2.50731	C	-7.60376	2.5707	-3.84515
H	-4.90338	-3.7972	2.14167	H	-7.81215	3.55702	-4.28358
C	-1.7398	-7.01951	0.18405	C	-6.63612	-3.00173	5.40862
C	-2.99614	-6.53548	-0.30221	H	-7.22473	-3.92009	5.47077
C	-3.47945	-7.02454	-1.5717	C	0.73947	-3.94788	-4.65766
H	-4.43505	-6.66403	-1.9434	H	1.76331	-3.88099	-5.03231
C	-2.76764	-7.94122	-2.29054	C	-2.29183	4.39523	-5.25595
H	-3.14011	-8.32945	-3.24118	H	-1.48088	4.61973	-5.949
C	-1.50648	-8.42348	-1.8084	C	3.79688	-1.36925	-6.4802
H	-0.96778	-9.16094	-2.40594	C	2.49424	-1.49692	-5.99081
C	-1.01306	-7.9766	-0.61879	H	2.26114	-1.13522	-4.97702
H	-0.05836	-8.34974	-0.23906	C	1.46957	-2.06074	-6.75378
N	7.28506	1.70017	-0.21088	C	1.77629	-2.56862	-8.05019
C	-3.23278	-5.13859	1.70459	C	3.09957	-2.50111	-8.54438
C	-1.25275	-6.57827	1.43683	C	4.09892	-1.84853	-7.78515
C	-6.22924	1.12579	-2.46795	C	8.42937	1.766	-2.76201

H	-5.35313	0.94091	-1.82174	H	8.87888	1.78135	-3.76542
C	6.51424	-0.88692	-3.84983	C	9.0871	2.35046	-1.6763
C	-1.98686	-5.64425	2.1997	H	10.06042	2.83943	-1.81296
C	-5.23633	-3.89888	-0.59719	C	-5.49618	2.11855	2.08443
H	-4.40127	-3.18729	-0.70102	H	-6.18805	1.90478	1.27343
C	-4.20535	2.99426	-4.72663	C	-4.77495	3.2765	2.07941
H	-4.87387	2.17029	-4.96338	H	-4.85786	3.98945	1.25521
C	-0.90794	-1.26773	-6.75834	C	7.50656	-3.05319	-3.30854
C	-4.47415	3.78859	-3.55098	C	4.91082	-4.18923	0.85041
C	-2.27014	-1.40412	-6.33733	C	5.24487	-3.65502	-0.44844
C	5.6882	-1.51487	-4.83666	H	5.97405	-2.85076	-0.51468
C	-3.39252	0.88288	6.41547	C	4.66952	-4.15312	-1.58076
C	-2.02297	0.86577	6.18211	H	4.91015	-3.74129	-2.56379
H	-1.65168	0.62021	5.17391	C	3.74124	-5.24311	-1.51138
C	-1.0799	1.1498	7.18933	H	3.33756	-5.64325	-2.44069
C	-1.54516	1.38593	8.49878	C	3.39838	-5.77001	-0.30245
C	-2.94597	1.4388	8.76207	H	2.70761	-6.61547	-0.23907
C	-3.86757	1.2181	7.72137	C	3.9455	-5.24318	0.92701
C	-3.81052	5.64999	-2.07642	C	6.68898	-3.68171	-4.30552
N	-6.46477	-3.50747	-1.06013	H	6.81046	-4.75219	-4.47822
C	4.82949	-0.72664	-5.63712	C	-1.61044	-3.18883	-4.78922
C	-6.00311	-0.92327	4.28503	C	-2.60982	-2.38087	-5.37337
C	-3.72647	-5.59994	0.46588	C	1.98375	5.42317	1.37431
C	-1.55606	0.53958	-8.27775	C	1.99999	4.58969	2.55364
H	-1.31865	1.29142	-9.03189	H	2.95149	4.21422	2.91867
C	5.73875	1.31445	-4.60129	C	0.84826	4.28655	3.21928
C	5.79593	2.7566	-4.57674	H	0.85929	3.6528	4.10887
H	6.38766	3.24428	-3.80599	C	-0.40861	4.81418	2.77768
C	5.15058	3.5007	-5.52132	H	-1.30497	4.56898	3.34667
H	5.21879	4.59057	-5.52594	C	-0.46248	5.61704	1.67777
C	4.36797	2.86658	-6.54374	H	-1.41182	6.05077	1.35527
H	3.88919	3.49425	-7.2964	C	0.72598	5.93885	0.92131
C	4.24763	1.50945	-6.56938	H	8.03922	-1.23682	-2.32699
H	3.65748	1.01149	-7.34266	H	8.213	-3.67536	-2.75537
C	4.92755	0.68122	-5.59943	O	5.38027	-1.60376	-8.1472
N	7.12747	-0.31485	1.76614	O	3.39689	-2.99426	-9.80775
C	6.17791	-1.30071	1.82171	O	0.69234	-3.08126	-8.68114
H	5.11973	-0.99711	1.75821	O	-0.81579	1.61406	9.62387
C	6.54156	-2.65558	1.95337	O	-3.43823	1.55733	10.0464
C	7.9098	-2.98502	2.01023	O	-5.22037	1.24685	7.80412
H	8.21313	-4.03643	2.10853	O	3.73574	-8.75459	2.95892
C	8.86801	-1.97175	1.94271	O	1.55023	-10.27298	3.02667
H	9.9383	-2.21232	1.98389	O	-1.01344	-9.07931	2.46957
C	8.45814	-0.63876	1.82591	O	-0.08366	9.38463	-0.07987
H	9.18119	0.18796	1.77688	O	-2.55036	10.38124	-1.30311
C	7.18046	1.14903	-2.56626	O	-4.38439	8.29614	-2.25401
C	-4.34495	0.57393	5.32544	C	0.60811	1.33821	9.65496
C	-5.05274	-5.16444	-0.00884	C	-3.05884	2.76979	10.75903
C	-6.13122	-0.02118	3.2045	C	-5.8584	1.57598	9.07442
C	5.79873	-2.94165	-5.02646	C	5.02584	-8.26287	2.51048
H	5.1739	-3.4068	-5.79259	C	2.16908	-10.6069	4.30228
C	-0.58909	-0.25808	-7.74192	C	-1.04909	-10.45917	2.94332
H	0.45329	-0.1561	-8.05365	C	5.80983	-1.81606	-9.52536

C	-3.26418	-0.53829	-6.92608	C	3.85148	-4.3812	-9.79448
H	-4.30061	-0.64601	-6.61608	C	0.76506	-3.44644	-10.09134
N	6.04161	1.53196	3.58508	C	-0.39419	10.7709	0.25257
C	7.04882	1.57402	4.51616	C	-2.08245	11.06143	-2.50693
H	8.07947	1.64124	4.13766	C	-4.96543	9.63321	-2.22534
C	6.76663	1.53092	5.88513	H	0.79639	1.33626	10.74093
H	7.58617	1.56441	6.61461	H	1.14982	2.15264	9.16672
C	5.43933	1.44873	6.31048	H	0.83302	0.35966	9.22429
H	5.19608	1.41636	7.38146	H	-3.36745	3.656	10.20342
C	4.40272	1.41058	5.35787	H	-1.98073	2.76613	10.95844
C	4.7361	1.4507	3.98906	H	-3.62589	2.65749	11.69311
H	3.95817	1.41986	3.20932	H	-5.62741	2.60846	9.34872
C	-3.59122	4.87153	-3.23649	H	-5.53482	0.87628	9.85533
C	2.99719	1.3413	5.80345	H	-6.91779	1.44538	8.81876
C	2.31957	2.53304	6.14253	H	5.39666	-7.52379	3.2259
C	2.95204	3.82694	6.03924	H	4.97868	-7.87706	1.48988
H	3.9863	3.87836	5.70869	H	5.6289	-9.18451	2.54968
C	2.27861	4.96744	6.36851	H	2.07061	-11.70097	4.31025
H	2.75725	5.94746	6.3061	H	1.62314	-10.14687	5.12675
C	0.91471	4.90647	6.80979	H	3.2256	-10.31392	4.29552
H	0.4062	5.84011	7.05811	H	-0.39177	-11.0886	2.33034
C	0.28476	3.70331	6.92087	H	-0.76134	-10.49695	3.99717
H	-0.75312	3.64703	7.26005	H	-2.10835	-10.70602	2.79678
C	0.96618	2.46773	6.60705	H	6.69613	-1.16849	-9.57661
C	-3.47211	-3.77139	3.71714	H	5.03836	-1.50861	-10.24021
H	-4.01971	-3.04908	4.32867	H	6.07345	-2.87007	-9.65021
C	-3.15424	3.28703	-5.54599	H	4.78073	-4.47208	-9.22654
H	-2.95156	2.69459	-6.44059	H	4.01807	-4.57197	-10.86337
C	-4.94906	5.41536	-1.26937	H	3.07418	-5.03452	-9.39046
C	-5.25511	6.2402	-0.12224	H	1.34909	-2.71865	-10.66638
H	-4.56295	7.04566	0.13418	H	-0.29609	-3.4326	-10.37306
C	-6.38689	6.04479	0.61217	H	1.19059	-4.45084	-10.1708
H	-6.63039	6.68439	1.46232	H	-1.42964	10.88152	0.59376
C	-7.29571	4.99009	0.2678	H	-0.19539	11.39354	-0.62418
H	-8.20022	4.87128	0.86827	H	0.32503	10.9654	1.05944
C	-7.02987	4.1654	-0.7865	H	-0.99216	11.02488	-2.5664
H	-7.72483	3.37209	-1.0498	H	-2.43888	12.08651	-2.3377
C	-5.85234	4.35396	-1.60062	H	-2.54283	10.62339	-3.39644
C	3.55337	-5.75552	2.18666	H	-4.56891	10.20596	-3.06878
C	-5.09078	-0.62655	5.3496	H	-6.03209	9.4063	-2.35847
C	0.33515	1.21239	6.76821	H	-4.76973	10.13618	-1.27168
C	-6.16477	-6.02357	0.10889	C	0.83334	3.79626	-3.43689
H	-6.0393	-7.01257	0.57272	C	2.09141	3.10307	-4.00169
C	-8.44581	1.4849	-4.0974	C	2.12403	1.60212	-3.6886
H	-9.32773	1.60163	-4.74053	C	2.0106	1.37426	-2.1768
C	8.50282	2.30412	-0.40676	C	0.68026	1.99799	-1.70145
H	8.99177	2.74654	0.47252	C	0.67043	3.51009	-1.92741
C	0.08356	-2.13158	-6.23681	O	0.53714	1.78354	-0.27242
C	3.11658	6.59289	-0.46359	C	0.5414	0.38118	0.05983
C	1.85824	7.11377	-0.90788	O	1.82033	-0.15818	-0.31646
C	1.84829	8.0004	-2.04988	C	2.1268	-0.10926	-1.74574
H	0.8905	8.41199	-2.375	C	0.91497	5.29944	-3.70722
C	2.99698	8.33121	-2.70521	H	-0.06458	3.38819	-3.96602

H	2.99032	9.01432	-3.5558	H	2.13693	3.25469	-5.09771
C	4.24989	7.78336	-2.27678	H	3.00126	3.5884	-3.59819
H	5.15134	8.06099	-2.8274	H	3.06469	1.16284	-4.07055
C	4.30583	6.9395	-1.20543	H	1.30432	1.08812	-4.22545
H	5.25958	6.51943	-0.89662	H	-0.19772	1.51428	-2.2036
C	0.4122	-4.84664	-3.687	H	1.47541	3.99475	-1.34149
H	1.15754	-5.5118	-3.25283	H	-0.27213	3.95433	-1.5425
C	-7.54215	-4.34703	-0.9526	H	-0.26031	-0.13512	-0.5057
H	-8.50455	-3.98479	-1.34189	C	0.45348	0.27023	1.57507
C	-6.76203	-2.14958	4.3496	C	1.20474	-1.06372	-2.50207
H	-7.45588	-2.3804	3.54539	C	3.57197	-0.61314	-1.77858
C	-1.52679	-5.20015	3.49571	H	0.93002	5.51266	-4.78226
H	-0.59611	-5.62327	3.88127	H	1.82565	5.73947	-3.2804
C	-5.72376	-2.7077	6.47508	H	0.06456	5.84212	-3.27897
H	-5.65368	-3.4086	7.30906	H	2.83726	1.92614	-1.66342
C	0.04787	-7.08393	1.93034	H	4.23887	0.0379	-1.19588
C	0.1697	-8.42013	2.42142	H	3.95521	-0.67004	-2.80467
C	1.44291	-8.92468	2.7492	H	3.63465	-1.61848	-1.33681
C	2.59265	-8.09053	2.63115	H	1.54845	-1.22556	-3.53646
C	2.45512	-6.74071	2.25023	H	0.17215	-0.70551	-2.55454
C	1.17439	-6.27286	1.88983	H	1.17694	-2.04686	-2.01213
H	1.07001	-5.23514	1.55677	C	-0.70165	-0.66531	1.9954
C	3.1663	5.75622	0.67639	C	-2.07537	-0.00519	1.7405
C	-0.94298	-4.94977	-3.2284	C	-2.99683	-0.83402	0.83057
H	-1.17317	-5.68458	-2.45156	C	-2.6907	-0.73586	-0.63516
C	6.62622	1.1384	-1.26833	C	-2.7123	0.39165	-1.36748
H	5.63384	0.67172	-1.07544	C	-3.01078	1.7455	-0.81247
C	-6.47878	2.39911	-3.01691	C	-2.35859	0.38195	-2.82369
C	-1.91653	-4.15807	-3.76295	H	0.33269	1.28113	2.0162
H	-2.94607	-4.25941	-3.42931	H	1.4183	-0.1122	1.96647
C	2.36844	0.08615	5.94881	H	-1.93235	1.00874	1.31642
C	1.03346	0.02214	6.46331	H	-2.5994	0.17134	2.70183
C	0.43507	-1.27645	6.6748	H	-4.05222	-0.50747	1.00514
H	-0.56696	-1.31426	7.11146	H	-2.95856	-1.89621	1.15581
C	1.1007	-2.42073	6.35191	H	-2.39949	-1.68176	-1.0911
H	0.65735	-3.40226	6.51947	H	-3.33367	2.46933	-1.57232
C	2.42031	-2.35198	5.79308	H	-2.09234	2.16522	-0.35391
H	2.92063	-3.28783	5.53032	H	-3.76934	1.73038	-0.01833
C	3.03372	-1.14817	5.60469	H	-2.21451	-0.62065	-3.24573
H	4.04111	-1.10457	5.19804	H	-1.40418	0.93218	-2.97775
C	7.40653	-1.71236	-3.07188	H	-3.10191	0.90628	-3.44011
C	5.51077	-3.70836	2.03472	H	-0.62418	-1.60087	1.38465
C	-0.25267	-3.06891	-5.23374	C	-0.55302	-1.0574	3.46814
C	-5.38346	1.17602	3.17269	H	-0.69074	-0.1979	4.14259
C	-8.16444	0.24136	-3.52075	H	0.43572	-1.48031	3.682
H	-8.80586	-0.63531	-3.68881	H	-1.29392	-1.81746	3.74715

Table S11c. Cartesian coordinates of **1a•MCA-c** (R = -OCH_3; E_{opt} = 1.53215043365 hartree, E_{sp} = –9754.14087049 hartree).

Pt	-6.75999	0.95752	1.8626	C	-1.43131	-3.91755	5.32097
Pt	6.87275	-1.03636	-1.71165	H	-0.54236	-3.88143	5.94946
N	-6.04886	1.76657	3.54319	C	-0.73245	0.204	7.95863

C	-4.72149	1.66047	3.86629	H	0.30972	0.15404	8.28449
H	-4.06471	1.12705	3.15866	C	2.94411	5.94416	-2.10142
C	-4.21474	2.21936	5.05506	C	-4.26105	5.27698	-0.4197
C	-5.10178	2.91099	5.90559	C	-8.1264	-2.27997	4.26221
H	-4.7229	3.35969	6.83486	H	-8.99047	-2.45526	4.91619
C	-6.45002	3.01741	5.56168	C	0.15145	4.57001	3.63462
H	-7.15179	3.55303	6.2136	H	0.87661	5.23851	3.1729
C	-6.9091	2.43392	4.37485	C	0.50034	3.7401	4.65785
H	-7.96329	2.49214	4.0689	H	1.51976	3.73901	5.05023
N	-6.68253	2.75406	0.98567	C	-0.60101	7.18392	-1.66673
N	-7.4656	0.15515	0.17557	C	0.52798	6.369	-1.74676
C	-8.80433	-0.12268	0.07588	H	0.42077	5.29284	-1.52778
H	-9.43342	0.10938	0.94694	C	1.7881	6.86667	-2.08329
C	-9.3369	-0.68219	-1.09149	C	1.9233	8.25295	-2.37288
H	-10.41128	-0.89899	-1.15183	C	0.77922	9.08668	-2.37963
C	-8.49581	-0.95934	-2.17113	C	-0.47589	8.56677	-1.98325
H	-8.89512	-1.39827	-3.09642	C	2.78049	-6.52884	1.57369
C	-7.12088	-0.67172	-2.07219	C	3.97069	-5.93889	1.03865
C	-6.63118	-0.11213	-0.87627	C	5.15605	-5.91436	1.86275
H	-5.55144	0.12788	-0.75712	H	6.06503	-5.47398	1.46107
C	3.10145	-1.41204	-5.65931	C	5.1519	-6.44768	3.11934
N	7.14558	0.93178	-1.93533	H	6.05609	-6.45967	3.73229
C	8.41023	1.41934	-2.13234	C	3.95522	-7.01766	3.66527
H	9.23657	0.69489	-2.13739	H	3.98616	-7.42836	4.67596
C	8.6303	2.79	-2.31846	C	2.81369	-7.05047	2.92096
H	9.65166	3.16261	-2.4718	H	1.89472	-7.48046	3.32931
C	7.54803	3.6706	-2.30977	C	-6.74035	2.11167	-5.28283
H	7.69988	4.74979	-2.45741	H	-7.34876	3.01816	-5.3181
C	6.244	3.17241	-2.11058	C	-2.12666	5.54399	-3.5086
C	6.07464	1.78753	-1.92091	H	-1.2422	6.09488	-3.83682
H	5.07778	1.35261	-1.75637	C	-0.46326	2.85317	5.26875
C	-3.59555	-2.9532	4.78007	C	-6.74034	5.3146	-0.12653
H	-4.37434	-2.21473	4.95147	H	-6.75721	6.32357	-0.5615
C	-4.15437	-6.00328	1.82195	C	-3.04711	-0.49531	8.12372
C	-5.20108	-5.05704	2.07059	H	-3.78491	-1.12071	8.63165
C	-6.43483	-5.17734	1.32997	C	-3.91767	-3.69249	-4.37338
H	-7.23714	-4.47272	1.53334	H	-3.26798	-3.89596	-5.22759
C	-6.61269	-6.17605	0.41725	C	-2.79448	4.72343	-4.36737
H	-7.55494	-6.28986	-0.12316	H	-2.46649	4.59199	-5.39787
C	-5.5604	-7.11316	0.15091	C	-1.00414	-1.71192	-6.95169
H	-5.73834	-7.90164	-0.58221	C	-2.02856	-1.59022	-6.00928
C	-4.37813	-7.0246	0.82372	H	-1.76498	-1.42258	-4.95941
H	-3.57828	-7.74481	0.63317	C	-3.3767	-1.65576	-6.36837
N	6.25516	-1.14465	-3.60789	C	-3.72	-1.92171	-7.72519
C	-3.81357	-3.97354	3.78126	C	-2.6999	-2.09367	-8.68976
C	-2.95067	-5.93294	2.56138	C	-1.3456	-1.92256	-8.31777
C	-5.5202	3.3339	0.56153	C	5.51492	-1.28248	-6.29706
H	-4.57478	2.75046	0.65774	H	5.21571	-1.3351	-7.354
C	2.58871	-2.66356	-6.07041	C	6.86085	-1.19769	-5.94034
C	-2.78409	-4.94375	3.55769	H	7.64342	-1.18293	-6.71031
C	-5.97614	-1.78039	2.59764	C	-2.14824	3.7909	3.72055
H	-5.12647	-1.54097	1.92124	H	-3.17546	3.82976	3.36589
C	-4.44463	4.19552	-2.66294	C	-1.19964	4.59026	3.15382

H	-5.34665	3.68064	-2.33965	H	-1.44962	5.2697	2.33505
C	-5.16451	-0.24552	-5.31159	C	2.89385	-5.05285	-6.49477
C	-3.7721	5.06629	-1.72579	C	2.78358	-5.44459	-1.05186
C	-6.08169	0.00096	-4.23891	C	2.73182	-4.89322	-2.3859
C	1.22181	-2.76518	-6.48548	H	3.6281	-4.43989	-2.80034
C	1.28429	1.97304	6.8376	C	1.58932	-4.95317	-3.1292
C	2.32924	1.44806	6.08749	H	1.54839	-4.53595	-4.13908
H	2.12067	1.05387	5.08854	C	0.41173	-5.5833	-2.60741
C	3.65529	1.4076	6.56553	H	-0.4762	-5.62999	-3.23703
C	3.91329	1.8537	7.87713	C	0.42426	-6.1243	-1.35677
C	2.8664	2.42749	8.65731	H	-0.46097	-6.62793	-0.9581
C	1.56413	2.51437	8.13166	C	1.60321	-6.06201	-0.5233
C	-1.88728	6.58138	-1.25202	C	1.52072	-5.16114	-6.89316
N	-6.92342	-0.81186	2.77902	H	1.14628	-6.13598	-7.21024
C	0.40716	-1.60983	-6.52169	C	-5.4967	-2.16393	-3.24102
C	-2.43457	1.19293	6.46305	C	-6.20546	-0.94432	-3.19727
C	-5.00388	-4.03546	3.02515	C	4.55672	4.53803	-0.87724
C	-5.81376	1.87437	-6.35172	C	5.07091	4.08003	0.39149
H	-5.75098	2.60696	-7.15792	H	5.88731	3.36285	0.3985
C	2.28609	-0.25979	-5.66955	C	4.56187	4.55167	1.56662
C	2.78108	1.03123	-5.2518	H	4.94838	4.2063	2.52845
H	3.81069	1.11276	-4.91319	C	3.50775	5.52295	1.56972
C	1.9876	2.13999	-5.30251	H	3.15041	5.90003	2.52734
H	2.36468	3.11913	-4.99396	C	2.99114	5.97244	0.39114
C	0.63997	2.04959	-5.78438	H	2.20111	6.72801	0.37981
H	0.04533	2.96064	-5.8409	C	3.48276	5.48498	-0.87699
C	0.13713	0.84791	-6.18677	H	4.4461	-3.78103	-5.78415
H	-0.87836	0.77297	-6.58462	H	3.51421	-5.95153	-6.52927
C	0.93071	-0.35809	-6.12233	O	-0.26494	-1.93178	-9.13439
N	6.58947	-3.00205	-1.50724	O	-3.03037	-2.34133	-10.01538
C	5.41655	-3.49652	-1.00308	O	-5.05774	-1.96462	-7.92502
H	4.62133	-2.76797	-0.72725	O	5.096	1.8552	8.54927
C	5.21382	-4.88128	-0.84551	O	3.05403	2.7731	9.98075
C	6.24521	-5.75863	-1.23907	O	0.47062	3.0424	8.73524
H	6.1063	-6.8433	-1.13284	O	1.10118	-9.63587	1.20284
C	7.43329	-5.24338	-1.75959	O	-1.1522	-10.49637	2.3005
H	8.24334	-5.91515	-2.07223	O	-3.11156	-8.58053	3.19703
C	7.59135	-3.85795	-1.88373	O	3.20509	8.61585	-2.61737
H	8.51189	-3.4127	-2.28661	O	0.90582	10.43445	-2.6889
C	4.52559	-1.30381	-5.29218	O	-1.64129	9.24112	-1.84725
C	-0.10433	1.98275	6.32288	C	6.21912	1.06418	8.08287
C	-6.05939	-3.02822	3.24391	C	4.06541	3.79094	10.23127
C	-2.79287	2.08424	5.42556	C	0.59301	3.62101	10.06903
C	0.71789	-4.0595	-6.88473	C	2.16981	-9.41436	0.24777
H	-0.32779	-4.13089	-7.19344	C	-0.16746	-11.22942	3.08472
C	-5.05255	0.74365	-6.35922	C	-3.36324	-9.96373	3.58739
H	-4.3536	0.55096	-7.17647	C	-0.43818	-1.9053	-10.5829
C	-6.85812	1.21842	-4.25819	C	-3.08578	-3.76322	-10.34099
H	-7.56599	1.39928	-3.45292	C	-5.60386	-2.05008	-9.27458
N	7.58433	-0.92708	0.15545	C	3.56615	10.027	-2.70083
C	8.91565	-1.19897	0.35021	C	0.72871	10.72027	-4.10927
H	9.50248	-1.49662	-0.53035	C	-1.66956	10.69613	-1.94394
C	9.49095	-1.1022	1.62153	H	6.86143	1.06629	8.97881

H	10.55598	-1.33201	1.75851	H	6.71015	1.57536	7.25093
C	8.70586	-0.70814	2.70855	H	5.91131	0.04564	7.83483
H	9.14395	-0.61692	3.71244	H	3.83787	4.70418	9.68049
C	7.34236	-0.42444	2.51211	H	5.06002	3.40074	9.98475
C	6.80335	-0.55815	1.21522	H	3.96383	3.93373	11.31569
H	5.72429	-0.38511	1.02866	H	1.23198	4.50702	10.03124
C	-2.57881	5.7357	-2.1492	H	0.98877	2.87703	10.77199
C	6.47949	0.02562	3.62155	H	-0.45199	3.87576	10.28609
C	6.42903	1.39972	3.94642	H	3.01587	-8.94135	0.75338
C	7.28895	2.36766	3.30742	H	1.82159	-8.8475	-0.61863
H	7.9871	2.02567	2.54748	H	2.41273	-10.45042	-0.03884
C	7.26475	3.68146	3.67629	H	-0.60416	-12.23723	3.0928
H	7.93997	4.41099	3.22276	H	-0.09076	-10.81515	4.09075
C	6.34752	4.13956	4.67997	H	0.799	-11.23013	2.56731
H	6.35801	5.19711	4.95093	H	-3.32265	-10.62042	2.70945
C	5.49844	3.26028	5.28302	H	-2.63954	-10.27238	4.3461
H	4.79358	3.60082	6.04679	H	-4.37892	-9.88569	3.99674
C	5.51834	1.85318	4.95427	H	0.53474	-1.51462	-10.90959
C	-2.44708	-2.92407	5.51566	H	-1.26227	-1.24568	-10.87791
H	-2.27847	-2.15386	6.27236	H	-0.60135	-2.92808	-10.93408
C	-3.97328	4.03074	-3.93177	H	-2.10629	-4.22568	-10.19795
H	-4.48359	3.37964	-4.64527	H	-3.36435	-3.73868	-11.40325
C	-2.39143	6.80303	0.04898	H	-3.85152	-4.26294	-9.7419
C	-1.73364	7.69664	0.97563	H	-5.0575	-1.40447	-9.97137
H	-0.8206	8.19754	0.64541	H	-6.63266	-1.70034	-9.11181
C	-2.24499	7.93136	2.21672	H	-5.58192	-3.09552	-9.59582
H	-1.7606	8.62016	2.91085	H	3.02732	10.62512	-1.95709
C	-3.45473	7.28107	2.63238	H	3.36603	10.37934	-3.71661
H	-3.84204	7.50145	3.62938	H	4.64328	9.9872	-2.4903
C	-4.09936	6.41743	1.79573	H	1.49908	10.21689	-4.69838
H	-5.01977	5.93259	2.11144	H	0.8556	11.81103	-4.13545
C	-3.59663	6.15124	0.4681	H	-0.27316	10.42963	-4.43561
C	1.61233	-6.61216	0.77922	H	-1.70999	10.9759	-3.00057
C	-1.07248	1.12866	6.90076	H	-2.61203	10.92733	-1.429
C	4.68666	0.92475	5.62375	H	-0.80757	11.14982	-1.44179
C	-7.15883	-3.27296	4.08804	C	0.6934	2.65009	0.46932
H	-7.24657	-4.24018	4.60285	C	1.09569	2.40418	-0.99803
C	-7.9243	4.70426	0.3008	C	-0.00871	2.83133	-1.97135
H	-8.88522	5.22462	0.2028	C	-1.39142	2.22984	-1.67577
C	7.21399	-1.13169	-4.58704	C	-1.73266	2.40947	-0.17833
H	8.26327	-1.06658	-4.26424	C	-0.63315	1.9312	0.76894
C	-4.41059	-1.44183	-5.33194	O	-2.98518	1.72947	0.1107
C	4.567	4.55824	-3.33242	C	-3.03926	0.31462	-0.18772
C	3.47624	5.48711	-3.32801	O	-1.92597	-0.06568	-0.95799
C	2.96073	5.95205	-4.5953	C	-1.59031	0.75797	-2.1315
H	2.13414	6.66657	-4.5813	C	1.78996	2.16493	1.4227
C	3.49711	5.53034	-5.77533	H	0.55299	3.74884	0.6256
H	3.1215	5.89328	-6.73344	H	2.02894	2.95205	-1.22933
C	4.59285	4.60499	-5.77719	H	1.31981	1.32538	-1.14459
H	5.00797	4.29909	-6.74029	H	0.29121	2.58977	-3.01039
C	5.10104	4.12879	-4.60312	H	-0.10328	3.9442	-1.94392
H	5.93517	3.43221	-4.61169	H	-2.02857	3.46498	0.03228
C	-4.09798	-4.62686	-3.39785	H	-0.49843	0.82925	0.6546

H	-3.59916	-5.59466	-3.4388	H	-0.93623	2.09859	1.81923
C	-7.9937	-1.05248	3.60153	H	-3.99205	0.20904	-0.76426
H	-8.73166	-0.24711	3.71971	C	-3.04164	-0.45391	1.14012
C	-3.40752	0.34445	7.10966	C	-2.73098	0.64607	-3.14429
H	-4.44385	0.39373	6.78555	C	-0.34779	0.01555	-2.61629
C	-1.59827	-4.8911	4.38203	H	1.52905	2.38846	2.46533
H	-0.84244	-5.6682	4.24586	H	2.74505	2.6612	1.21584
C	-1.68088	-0.57544	8.55108	H	1.94679	1.08331	1.3553
H	-1.42812	-1.25931	9.36328	H	0.47348	0.10744	-1.88723
C	-1.8422	-6.86924	2.26852	H	0.00642	0.39791	-3.58053
C	-1.95273	-8.26087	2.57169	H	-0.55728	-1.05635	-2.72641
C	-0.92597	-9.13981	2.17653	H	-2.41832	0.97646	-4.14196
C	0.23776	-8.63424	1.52675	H	-3.6032	1.25225	-2.8727
C	0.39375	-7.24673	1.32514	H	-3.07592	-0.3931	-3.23335
C	-0.67086	-6.39924	1.68601	C	-2.34856	-1.83853	1.13184
H	-0.56837	-5.31578	1.50289	C	-0.82456	-1.63175	1.22766
C	5.0876	4.08802	-2.10608	C	-0.00405	-2.91516	1.05155
C	-4.97904	-4.35865	-2.29803	C	1.3538	-2.73895	1.65925
H	-5.09985	-5.12884	-1.53158	C	2.40536	-2.17106	1.05127
C	4.92996	-1.22964	-3.94526	C	2.38398	-1.66374	-0.35834
H	4.19386	-1.23941	-3.12841	C	3.70568	-2.00591	1.77911
C	-5.51957	4.63056	0.00611	H	-4.10061	-0.57496	1.45909
C	-5.65327	-3.17514	-2.22219	H	-2.56025	0.18405	1.91308
H	-6.33791	-2.9864	-1.39873	H	-0.58992	-1.17877	2.21226
C	5.72889	-0.91548	4.35825	H	-0.5035	-0.88014	0.46628
C	4.81105	-0.45758	5.35782	H	0.06794	-3.17563	-0.02524
C	4.04833	-1.43679	6.09781	H	-0.52113	-3.77969	1.5328
H	3.35185	-1.07529	6.8601	H	1.43254	-3.09078	2.6893
C	4.2033	-2.77109	5.86894	H	2.20608	-0.57887	-0.37544
H	3.64134	-3.5136	6.43696	H	1.57433	-2.1304	-0.94429
C	5.13534	-3.22834	4.87875	H	3.32784	-1.87503	-0.88174
H	5.24461	-4.3051	4.73291	H	4.3098	-1.17596	1.39037
C	5.8656	-2.33736	4.14715	H	4.29824	-2.93157	1.73729
H	6.58209	-2.68793	3.40843	H	3.54544	-1.79689	2.85051
C	3.40464	-3.8544	-6.08634	H	-2.6772	-2.38052	2.05801
C	3.96178	-5.41342	-0.27426	C	-2.77467	-2.66979	-0.07779
C	-4.59793	-2.41833	-4.32669	H	-2.36171	-2.25374	-1.00942
C	-1.81857	2.89439	4.80429	H	-3.86196	-2.71136	-0.18905
C	-7.87573	3.42644	0.86055	H	-2.42331	-3.70443	0.0014
H	-8.77933	2.91289	1.21845	H	-2.13336	2.85279	-2.24903

Table S11d. Cartesian coordinates of **1a•MCA-d** (R = -OCH_3; E_{opt} = 1.53985012415 hartree, E_{sp} = –9754.14661262 hartree).

Pt	-6.19193	2.46489	-1.95282	C	-3.68298	2.07777	5.93599
Pt	6.19784	-2.58263	2.02934	H	-3.01947	2.52408	6.67597
N	-5.59766	4.31182	-1.46988	C	-2.03873	6.6314	4.06276
C	-4.48384	4.55825	-0.71253	H	-1.14782	6.81757	4.66857
H	-3.89886	3.69266	-0.36155	C	5.36513	1.49072	-4.73049
C	-4.10173	5.87685	-0.39177	C	-1.99896	2.8262	-5.43576
C	-4.88729	6.94494	-0.86669	C	-8.79459	2.8622	1.37642
H	-4.60223	7.97882	-0.62359	H	-9.72007	3.41851	1.57358
C	-6.02011	6.67964	-1.63882	C	0.83796	6.91254	-1.42169

H	-6.64346	7.50082	-2.01538	H	1.76835	7.16566	-1.92909
C	-6.36067	5.35492	-1.9309	C	0.74117	6.93534	-0.06194
H	-7.24207	5.10284	-2.53794	H	1.60273	7.19517	0.55858
N	-5.38012	2.70254	-3.76264	C	2.20106	2.67446	-6.4172
N	-6.86225	0.65275	-2.48201	C	3.11269	2.18446	-5.47996
C	-8.20043	0.53727	-2.76274	H	2.75148	1.88733	-4.48084
H	-8.81859	1.43892	-2.64312	C	4.47508	2.0652	-5.76227
C	-8.74629	-0.68092	-3.18069	C	4.96123	2.48577	-7.03086
H	-9.81968	-0.75278	-3.39925	C	4.05166	2.93326	-8.01812
C	-7.91716	-1.79557	-3.32381	C	2.6785	3.06136	-7.7032
H	-8.32521	-2.75893	-3.66211	C	0.18506	-2.87693	6.6838
C	-6.54339	-1.68297	-3.03715	C	1.5546	-3.05421	6.3043
C	-6.04159	-0.43636	-2.60723	C	2.58038	-2.45897	7.12775
H	-4.97586	-0.305	-2.36059	H	3.62172	-2.60231	6.85118
C	3.25408	-5.85374	-1.21756	C	2.25877	-1.74226	8.24404
N	7.24729	-1.61446	0.62925	H	3.03259	-1.30865	8.8819
C	8.61296	-1.59028	0.75846	C	0.88715	-1.54455	8.61323
H	9.04509	-2.10021	1.63121	H	0.66756	-0.96328	9.5107
C	9.41397	-0.93871	-0.18568	C	-0.10908	-2.08988	7.85957
H	10.5052	-0.93042	-0.06446	H	-1.15832	-1.94688	8.13318
C	8.81721	-0.30711	-1.279	C	-4.31001	-2.21398	-6.63601
H	9.42899	0.20662	-2.03488	H	-4.42949	-1.53179	-7.48006
C	7.41645	-0.33338	-1.41704	C	0.66831	0.24652	-6.06208
C	6.65047	-0.99736	-0.43689	H	1.70703	0.21737	-6.39965
H	5.55266	-1.04023	-0.50429	C	-0.50703	6.64321	0.6037
C	-5.16091	2.42806	4.03587	C	-4.31951	3.11012	-6.31235
H	-5.63113	3.08405	3.3078	H	-3.89545	3.27067	-7.31375
C	-6.09604	-1.75475	4.07394	C	-4.43191	6.31522	3.83894
C	-6.76411	-0.88802	3.1504	H	-5.40424	6.27643	4.3348
C	-7.78726	-1.44344	2.29645	C	-4.62222	-5.93319	-1.30158
H	-8.31373	-0.78105	1.61447	H	-3.91495	-6.75735	-1.41951
C	-8.12382	-2.76396	2.37156	C	-0.01756	-0.91012	-5.83965
H	-8.92312	-3.17951	1.755	H	0.45168	-1.8834	-5.97784
C	-7.44326	-3.63862	3.28109	C	-0.53718	-6.81643	-3.04021
H	-7.74387	-4.68622	3.32258	C	-1.56426	-5.87298	-2.94064
C	-6.46381	-3.15183	4.09525	H	-1.38604	-4.95102	-2.37913
H	-5.95082	-3.80907	4.8024	C	-2.80129	-6.0632	-3.55834
N	5.94827	-4.13661	0.79372	C	-3.04421	-7.27192	-4.27129
C	-5.48717	1.02165	4.01743	C	-2.0431	-8.26923	-4.32951
C	-5.12245	-1.22702	4.95304	C	-0.77032	-8.0191	-3.76387
C	-4.02298	2.66478	-3.94863	C	5.67557	-6.36723	-0.86622
H	-3.39089	2.47109	-3.06715	H	5.5612	-7.24476	-1.51908
C	2.41563	-6.81372	-0.60807	C	6.90828	-6.06626	-0.28549
C	-4.84823	0.15863	4.9653	H	7.78389	-6.70137	-0.47346
C	-6.46017	1.47556	0.81371	C	-1.50415	6.2943	-1.63093
H	-5.53109	0.91718	0.56144	H	-2.36901	6.04686	-2.24087
C	-2.03361	0.33303	-5.29992	C	-0.30367	6.56945	-2.2184
H	-3.08544	0.35805	-5.02612	H	-0.18635	6.54092	-3.30433
C	-3.96647	-4.06275	-4.51947	C	1.97791	-8.4329	1.17125
C	-1.34245	1.5799	-5.53102	C	0.84178	-4.37419	4.35992
C	-4.87743	-2.96666	-4.38003	C	1.12759	-5.12355	3.15981
C	1.1585	-7.14192	-1.21278	H	2.16248	-5.25232	2.85473
C	0.59825	6.85168	2.84181	C	0.12259	-5.67614	2.42013

C	1.4765	5.79169	3.03889	H	0.33706	-6.23872	1.50745
H	1.27746	4.83166	2.52751	C	-1.24446	-5.54189	2.83041
C	2.60672	5.89551	3.87378	H	-2.0191	-6.01229	2.22557
C	2.83524	7.11282	4.54749	C	-1.55296	-4.84725	3.96171
C	1.97591	8.22943	4.32642	H	-2.59127	-4.75181	4.2921
C	0.87065	8.10745	3.4643	C	-0.5246	-4.22135	4.76111
C	0.76343	2.74224	-6.07565	C	0.71436	-8.75501	0.57357
N	-7.02458	2.26589	-0.14831	H	0.09354	-9.51641	1.04852
C	0.78036	-6.53152	-2.4295	C	-5.59639	-3.84331	-2.20428
C	-3.03821	6.26397	1.83189	C	-5.64358	-2.84203	-3.19909
C	-6.41255	0.47904	3.10074	C	6.46436	1.68813	-2.53584
C	-3.40399	-3.31663	-6.7808	C	6.85432	2.52996	-1.43036
H	-2.87577	-3.43459	-7.728	H	7.3743	2.08082	-0.58809
C	2.88773	-5.25187	-2.44101	C	6.60629	3.87224	-1.45097
C	3.72821	-4.27845	-3.09749	H	6.91641	4.51773	-0.62683
H	4.65888	-3.98263	-2.62016	C	5.93398	4.47735	-2.56369
C	3.37718	-3.74881	-4.30504	H	5.77614	5.55617	-2.55303
H	4.01112	-3.0119	-4.80379	C	5.52435	3.70953	-3.61269
C	2.16717	-4.15482	-4.95838	H	5.02419	4.15765	-4.47548
H	1.94446	-3.74514	-5.94277	C	5.7682	2.28614	-3.63501
C	1.33602	-5.05268	-4.35762	H	3.74844	-7.25294	1.06668
H	0.41702	-5.38121	-4.84987	H	2.2692	-8.96542	2.07935
C	1.65104	-5.61622	-3.06469	O	0.32541	-8.81334	-3.82611
N	5.23198	-3.58455	3.46283	O	-2.27723	-9.45111	-5.01946
C	3.9234	-3.33899	3.78287	O	-4.27199	-7.30146	-4.84035
H	3.39379	-2.55906	3.21502	O	3.83343	7.42492	5.41817
C	3.27766	-4.06085	4.80655	O	2.10639	9.39512	5.05569
C	4.00778	-5.04456	5.50334	O	-0.04192	9.06188	3.15648
H	3.52101	-5.6164	6.30543	O	-2.34143	-4.87378	8.25799
C	5.34124	-5.28444	5.16828	O	-4.88575	-4.33319	8.75304
H	5.92275	-6.04803	5.70116	O	-6.23489	-2.31911	7.18622
C	5.93836	-4.54367	4.14236	O	6.30782	2.39164	-7.13278
H	6.98469	-4.70398	3.84425	O	4.51814	3.32375	-9.26626
C	4.56325	-5.53907	-0.61265	O	1.69447	3.5316	-8.50658
C	-0.6153	6.67462	2.0122	C	4.65668	6.39159	6.01501
C	-7.04228	1.35123	2.09189	C	3.36554	10.10659	4.88205
C	-2.90599	6.14366	0.43034	C	0.09656	10.39943	3.7228
C	0.31984	-8.12799	-0.57069	C	-1.11453	-5.57097	7.92465
H	-0.63598	-8.37153	-1.04088	C	-4.31958	-4.21455	10.09004
C	-3.23231	-4.19924	-5.75607	C	-6.97922	-2.82127	8.33641
H	-2.55345	-5.04948	-5.85821	C	0.3438	-9.9727	-4.71143
C	-5.00604	-2.03493	-5.47557	C	-2.80918	-10.51691	-4.17588
H	-5.70306	-1.20708	-5.37678	C	-4.63147	-8.36963	-5.76628
N	6.47356	-1.05434	3.28793	C	7.00504	2.99772	-8.26226
C	7.47185	-1.15719	4.22225	C	4.56276	2.2328	-10.23482
H	8.06508	-2.08267	4.22626	C	2.01792	4.16408	-9.7802
C	7.71431	-0.12165	5.13168	H	5.13163	6.95299	6.83619
H	8.51826	-0.22205	5.87256	H	5.40386	6.05202	5.29313
C	6.92976	1.03239	5.08781	H	4.0457	5.57719	6.41121
H	7.10516	1.856	5.79393	H	3.52526	10.35604	3.83246
C	5.9036	1.14257	4.12908	H	4.19112	9.51321	5.29199
C	5.69729	0.07299	3.23472	H	3.19285	11.00391	5.49141
H	4.90752	0.11558	2.46894	H	1.00011	10.87468	3.33236

C	0.0448	1.53779	-5.88326	H	0.11778	10.34889	4.81865
C	5.06547	2.35536	4.06532	H	-0.816	10.88259	3.35086
C	5.50853	3.47169	3.32291	H	-0.25796	-4.93093	8.15234
C	6.78477	3.48056	2.64736	H	-1.12205	-5.91912	6.88923
H	7.41926	2.60095	2.71847	H	-1.16331	-6.42396	8.62083
C	7.20912	4.5818	1.96233	H	-5.05071	-4.7779	10.68541
H	8.1927	4.60835	1.48789	H	-4.27058	-3.16877	10.39592
C	6.37325	5.74273	1.85704	H	-3.33633	-4.69789	10.12519
H	6.7403	6.60054	1.29144	H	-7.0166	-3.91757	8.31667
C	5.15721	5.76737	2.47226	H	-6.52249	-2.4579	9.26056
H	4.51053	6.64645	2.39951	H	-7.96568	-2.37225	8.16187
C	4.69062	4.64557	3.25462	H	1.42327	-10.13585	-4.83085
C	-4.2958	2.93398	4.96155	H	-0.14421	-9.75572	-5.6685
H	-4.05349	3.99869	4.98674	H	-0.13693	-10.81134	-4.19993
C	-1.39181	-0.86297	-5.4346	H	-2.0926	-10.77728	-3.39315
H	-1.91267	-1.80622	-5.25235	H	-2.92631	-11.33625	-4.89808
C	0.11337	3.98774	-5.92409	H	-3.77203	-10.22298	-3.75028
C	0.82012	5.23733	-6.09311	H	-3.80036	-8.6184	-6.43586
H	1.88988	5.19636	-6.31243	H	-5.4705	-7.91145	-6.3076
C	0.17531	6.43458	-6.0011	H	-4.95383	-9.24025	-5.18795
H	0.70396	7.37792	-6.14826	H	6.55736	3.95712	-8.5454
C	-1.2313	6.47479	-5.72372	H	6.99665	2.29069	-9.09655
H	-1.72047	7.45049	-5.67593	H	8.01521	3.12474	-7.8492
C	-1.93253	5.32019	-5.52959	H	5.25343	1.45452	-9.90104
H	-2.99747	5.35718	-5.31481	H	4.94187	2.74424	-11.13006
C	-1.28736	4.03187	-5.6247	H	3.56101	1.82919	-10.40234
C	-0.84297	-3.48314	5.92459	H	2.15762	3.38308	-10.53308
C	-1.87641	6.51315	2.63184	H	1.10706	4.74834	-9.96791
C	3.47502	4.7032	3.97497	H	2.90394	4.80422	-9.69857
C	-8.2248	2.06061	2.36928	C	0.99527	-0.98324	-1.84882
H	-8.69191	1.97557	3.36085	C	2.24085	-0.51017	-2.62863
C	-5.69964	3.14451	-6.10967	C	2.81823	0.81084	-2.08552
H	-6.38299	3.33165	-6.94793	C	1.93475	1.40951	-0.98679
C	7.02605	-4.94627	0.54607	C	0.47834	1.48364	-1.48944
H	7.97887	-4.67817	1.02468	C	-0.0917	0.11022	-1.85911
C	-3.83327	-5.00483	-3.47392	O	-0.36584	1.93599	-0.3972
C	6.46894	-0.46263	-3.71894	C	0.02379	3.18121	0.20048
C	5.75915	0.13564	-4.8093	O	1.41058	3.16372	0.56389
C	5.49706	-0.66336	-5.9838	C	2.38001	2.79839	-0.46605
H	4.95886	-0.19696	-6.81227	C	0.45206	-2.29067	-2.42582
C	5.93631	-1.95077	-6.07523	H	1.29271	-1.17043	-0.78622
H	5.77442	-2.54442	-6.9762	H	3.01304	-1.30005	-2.61068
C	6.64996	-2.54689	-4.983	H	1.97193	-0.38771	-3.69652
H	7.00001	-3.57523	-5.09623	H	2.92463	1.53474	-2.9263
C	6.88915	-1.83854	-3.8411	H	3.84347	0.65622	-1.70371
H	7.44003	-2.29097	-3.02079	H	-0.57619	0.15898	-2.85233
C	-5.45359	-5.87436	-0.22382	H	-0.89641	-0.15036	-1.13601
H	-5.43712	-6.64181	0.55042	C	-0.71885	3.32043	1.52884
C	-8.18156	2.95303	0.12243	C	3.67593	2.73358	0.34295
H	-8.59891	3.57324	-0.68292	C	2.43458	3.88412	-1.53581
C	-4.32008	6.16074	2.4873	H	0.13104	-2.1798	-3.46757
H	-5.20493	5.98983	1.88018	H	-0.4169	-2.64531	-1.8471
C	-3.94656	0.74092	5.93276	H	1.20881	-3.08337	-2.40449

H	-3.49732	0.07995	6.67768	H	1.56982	3.86837	-2.20696
C	-3.26856	6.54479	4.64498	H	3.33579	3.79313	-2.15388
H	-3.39587	6.66807	5.7216	H	2.46069	4.87827	-1.06825
C	-4.3533	-2.13008	5.83767	H	4.51051	2.36538	-0.26029
C	-4.95401	-2.71713	6.99258	H	3.5553	2.10018	1.22871
C	-4.22039	-3.64023	7.76108	H	3.95	3.73017	0.72013
C	-2.87384	-3.95233	7.40939	C	-0.94165	1.98281	2.26789
C	-2.25613	-3.30704	6.31891	C	-2.25508	1.32008	1.80255
C	-3.02662	-2.41901	5.54183	C	-2.38439	-0.14003	2.27298
H	-2.56039	-1.94459	4.67139	C	-2.1013	-1.10593	1.16213
C	6.75538	0.30689	-2.56941	C	-3.03898	-1.71821	0.42358
C	-6.39363	-4.79768	-0.09927	C	-4.50799	-1.50631	0.62767
H	-7.05379	-4.78771	0.77085	C	-2.68351	-2.69885	-0.65086
C	4.73317	-4.41974	0.22544	H	-1.67615	3.84977	1.37466
H	3.89517	-3.74251	0.44834	H	-0.09891	3.99413	2.17192
C	-3.4583	2.86759	-5.22265	H	-2.28843	1.35767	0.69385
C	-6.46106	-3.8194	-1.04803	H	-3.11817	1.90287	2.17199
H	-7.18573	-3.01392	-0.95194	H	-3.3941	-0.30263	2.70147
C	3.85902	2.41011	4.79764	H	-1.68771	-0.33644	3.11496
C	3.07279	3.60665	4.77259	H	-1.04059	-1.27168	0.97642
C	1.8889	3.67214	5.59795	H	-4.75141	-0.44162	0.76888
H	1.31462	4.60327	5.59813	H	-4.85179	-2.03625	1.53251
C	1.50309	2.61307	6.36482	H	-5.10924	-1.89113	-0.20337
H	0.61902	2.66553	7.00053	H	-1.6014	-2.72164	-0.87031
C	2.26732	1.3996	6.35279	H	-3.20003	-2.47725	-1.59409
H	1.91908	0.56287	6.96348	H	-2.98282	-3.71649	-0.35584
C	3.40102	1.30046	5.59941	H	-0.09056	1.30275	2.00921
H	3.98264	0.38258	5.60337	C	-0.94997	2.22106	3.78185
C	2.79025	-7.48924	0.61169	H	-1.19861	1.30416	4.32855
C	1.86597	-3.80757	5.15049	H	-1.68909	2.97568	4.07237
C	-4.66553	-4.9252	-2.33646	H	0.03079	2.5569	4.13557
C	-1.65888	6.35225	-0.19657	H	1.94279	0.72921	-0.09506
C	-6.21392	2.93971	-4.82408	H	0.37003	2.21352	-2.3208
H	-7.29444	2.96037	-4.62147	H	-0.15276	3.99975	-0.52644
